# Regioselective Magnesiations of Fluorinated Arenes and Heteroarenes Using Magnesium‐*bis*‐Diisopropylamide (MBDA) in Hydrocarbons

**DOI:** 10.1002/anie.202206176

**Published:** 2022-06-01

**Authors:** Andreas Hess, Nurtalya Alandini, Yusuf C. Guersoy, Paul Knochel

**Affiliations:** ^1^ Department Chemie Ludwig-Maximilians-Universität München Butenandtstrasse 5–13, Haus F 81377 München Germany

**Keywords:** Directed Metalation, Fluorine, Fluoroaromatics, N-Heterocyles, Organomagnesium Reagents

## Abstract

We report a convenient preparation of a new and storable magnesium amide (*i*Pr_2_N)_2_Mg (magnesium‐*bis*‐diisopropylamide; MBDA) which proved to be especially suitable for the non‐cryogenic magnesiation of fluoro‐substituted arenes and heteroarenes providing arylmagnesium amides (ArMgDA) or *bis*‐heteroaryl magnesiums (HetAr)_2_Mg in hydrocarbons. Further reactions with electrophiles (aldehydes, ketones, allylic bromides, aryl halides (Negishi cross‐coupling)) furnished a range of polyfunctional fluoro‐substituted unsaturated building blocks. Several postfunctionalizations were described as well as NMR‐studies confirming the dimeric structure of the base.

## Graphical abstract

Various fluoro‐substituted aromatics and heterocycles were magnesiated with the hydrocarbon‐soluble organomagnesium amide (*i*Pr_2_N)_2_Mg (magnesium‐*bis*‐diisopropylamide; MBDA) at non‐cryogenic temperatures providing thermally stable arylmagnesium amides (or diheteroarylmagnesium amides in the case of heterocyclic substrates). Trapping reactions with typical electrophiles furnished polyfunctional fluoro‐substituted building blocks.

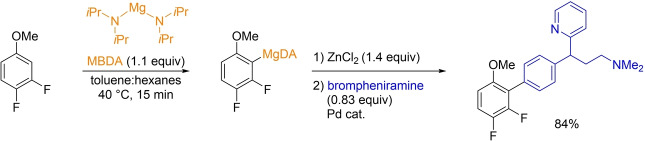

Fluorinated aromatics are important scaffolds present in numerous pharmaceuticals and agrochemicals.^[1]^ The special nature of fluorine imparts a range of useful properties, including enhanced binding interactions, metabolic stability, changes in physical properties^[2]^ and selective reactivities.^[3]^ The regioselective metalation of such aromatics using lithium bases may be complicated by the formation of aryne side‐products requiring cryogenic temperatures for such lithiations.^[4]^ Due to the increasing importance of polyfunctionalized fluorinated aromatics, we have envisioned to develop a convenient magnesiation of fluorinated unsaturated substrates since we anticipated that magnesiated fluoroaromatics should be significantly more stable and easy to handle.^[5]^ A range of magnesium amides in THF suitable for metalations have been reported.^[6]^ Among them, the mixed lithium magnesium amides TMPMgCl⋅LiCl **1** (TMP=2,2,6,6‐tetramethylpiperidyl),^[7]^ TMP_2_Mg⋅2 LiCl **2** ^[8]^ and [*t*Bu(*i*Pr)N]_2_Mg⋅2 LiCl **3** ^[9]^ have recently found many applications. The TMP group in combination with LiCl proved to be important for providing a monomeric, highly soluble base with remarkable reactivity.^[10]^ However, due to the high cost of TMPH compared to DA (diisopropylamine),^[11]^ we envisioned the preparation of a new DA‐based magnesium amide in hexanes. Previous reports of Kondo and Sakamoto have already described the magnesiation of indoles in THF with (*i*Pr_2_N)_2_Mg **4** and related bases.^[12a]^ Also, Lessène and Bordeau reported the regio‐ and stereo‐selective generation of silyl enol ethers with magnesium‐*bis*‐diisopropylamide (MBDA) **4**.^[12b]^ The use of an apolar, industrially friendly^[13]^ solvent compared to THF should suppress any aryne formation and allow magnesiations at non‐cryogenic temperatures. Thus, treating *i*Pr_2_NH (DA) with commercially available Bu_2_Mg^[14]^ in hexanes (25 °C, 4 h) produced a light‐yellow ca. 0.8 M solution of magnesium‐*bis*‐diisopropylamide **4** (MBDA) in quantitative yield. This solution was storable at ambient temperature for more than three months without decomposition or loss of activity. Herein, we wish to report that this base allowed the magnesiation of various fluorinated aromatics and heterocycles of type **5** and **6** in a convenient temperature range (−20 °C to 70 °C) leading to the corresponding organomagnesium species **7** or **8** (depending on the stoichiometry of base **4** used).^[15]^ After quenching with typical electrophiles such as aldehydes, ketones, allylic bromides, disulfides or aryl halides, a range of polyfunctionalized fluorinated aromatics and heterocycles of type **9** and **10** were obtained in 52–96 % yield (Scheme 1).

**Scheme 1:**
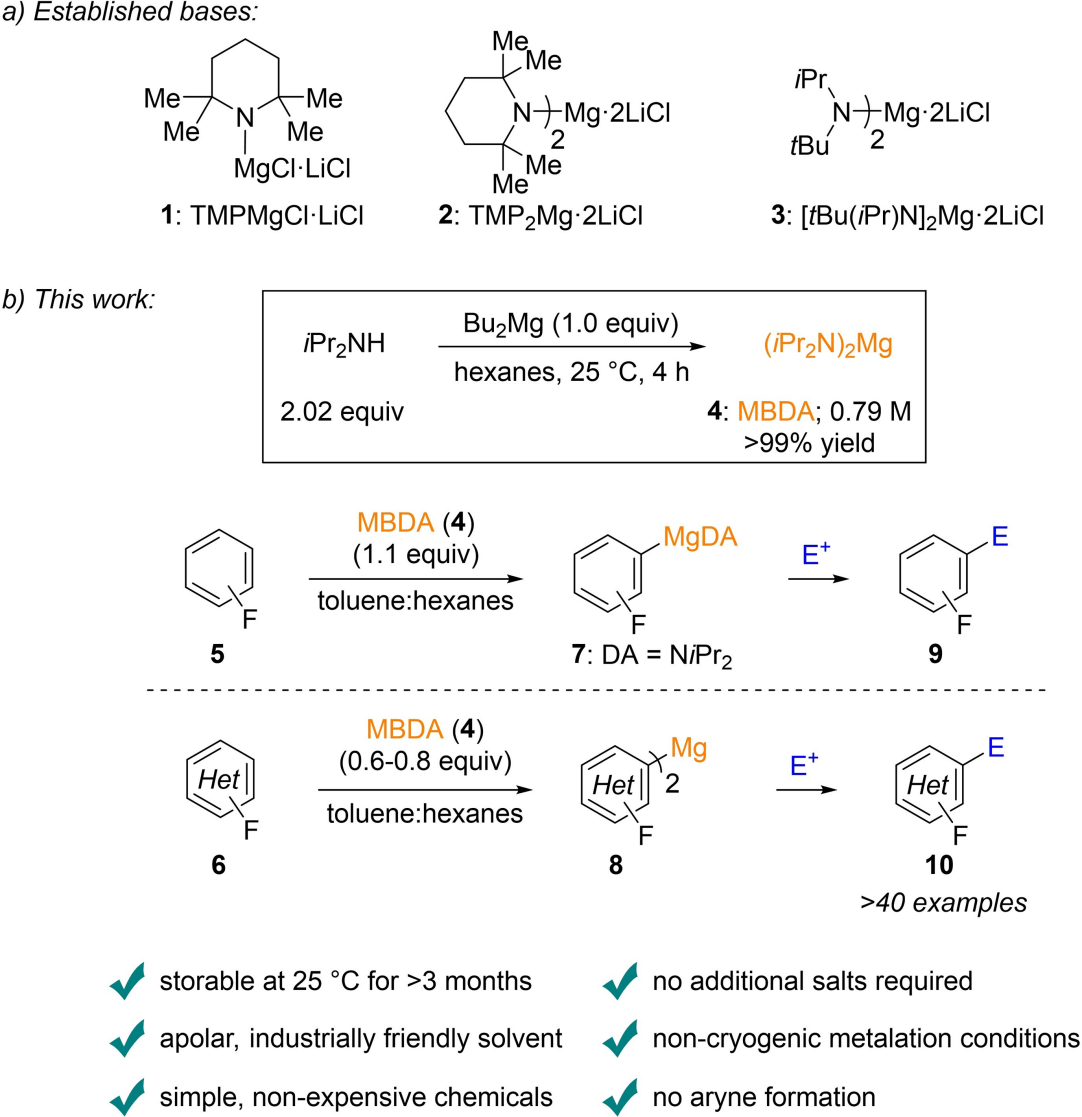
a) Valuable magnesium amide bases. b) Preparation of MBDA **4** and its reaction with fluoroarenes **5** or heteroarenes **6**.

Thus, various halogenated fluoroaromatics such as pentafluorobenzene (**5 a**), 5‐bromo‐1,2,3‐trifluorobenzene (**5 b**), 1,2,4‐trifluorobenzene (**5 c**), 1,2‐dibromo‐4,5‐difluorobenzene (**5 d**), 1,4‐dibromo‐2,5‐difluorobenzene (**5 e**), 1,3‐dibromo‐2‐chloro‐5‐fluorobenzene (**5 f**), and 1,2‐dibromo‐5‐chloro‐3‐fluorobenzene (**5 g**) were all readily magnesiated with MBDA (**4**, 1.1 equiv) in toluene:hexanes at 25 °C within 5–45 min as indicated by iodolysis of reaction aliquots. The resulting arylmagnesium amides (**7 a**–**7 g**) were quenched with several electrophiles (1.2–1.4 equiv) such as iodine, aldehydes, aryl iodides (Negishi cross‐coupling)^[16]^ and allylic bromides leading to the desired products **9 a**–**h** in 52–84 % yield. The organomagnesium amides **7 a**–**g** proved to be thermally stable and for example the reagent **7 e** was stable in hexanes at 40 °C for four days without significant decomposition. In no cases aryne‐derived side products were observed. Various electron‐rich substitutents such as an iodide, methoxy, TBS‐O or 1,3‐dioxolane in aromatic substrates **5 h**–**l** were similarly metalated with MBDA **4**. However, due to the increased electronic density of these ring systems, higher magnesiation temperatures and longer reaction times were required (25–70 °C, 15 min–1 h; see Scheme 2). After quenching with various electrophiles the desired functionalized aromatics **9 m**–**p** were obtained in 60–94 % yield. Electron‐withdrawing substituents such as *t*‐butyl esters were compatible with a metalation using MBDA **4**. Thus, the *tert*‐butyl benzoates **5 m**, **5 n** and **5 o** were readily magnesiated at 25 °C within 15–20 min. Interestingly, in the case of *t*‐butyl 3‐fluorobenzoate (**5 o**) a metalation with MBDA **4** in toluene:hexanes was complicated by a competitive reaction with the ester function. This side reaction was widely suppressed by the addition of 3 equivalents THF accelerating this magnesiation.^[20]^ Quenching with typical electrophiles gave the fluorobenzoates **9 r**–**t** in 62–75 % yield. Although fluorobenzonitriles were not magnesiated with MBDA due to extensive reaction of the cyano group, the corresponding *N*,*N*‐diisopropylamido derivatives **5 p** and **5 q** were magnesiated at 25 °C and reacted well in various trapping reactions affording the polyfunctional amides **9 u**–**x** in 63–80 % yield. Also, the fluorinated aryl oxazoline **5 r** was successfully magnesiated at 60 °C (0.5 h) providing, after a Negishi cross‐coupling, the polyfunctional biphenyl **9 y** in 96 % yield. Finally, the triazene **5 s** was smoothly magnesiated with MBDA at 0 °C (1 h) and trapping with furfural or cross‐coupling gave the poly‐substituted triazenes **9 z‐aa** in 60–74 % yield.^[21]^

**Scheme 2:**
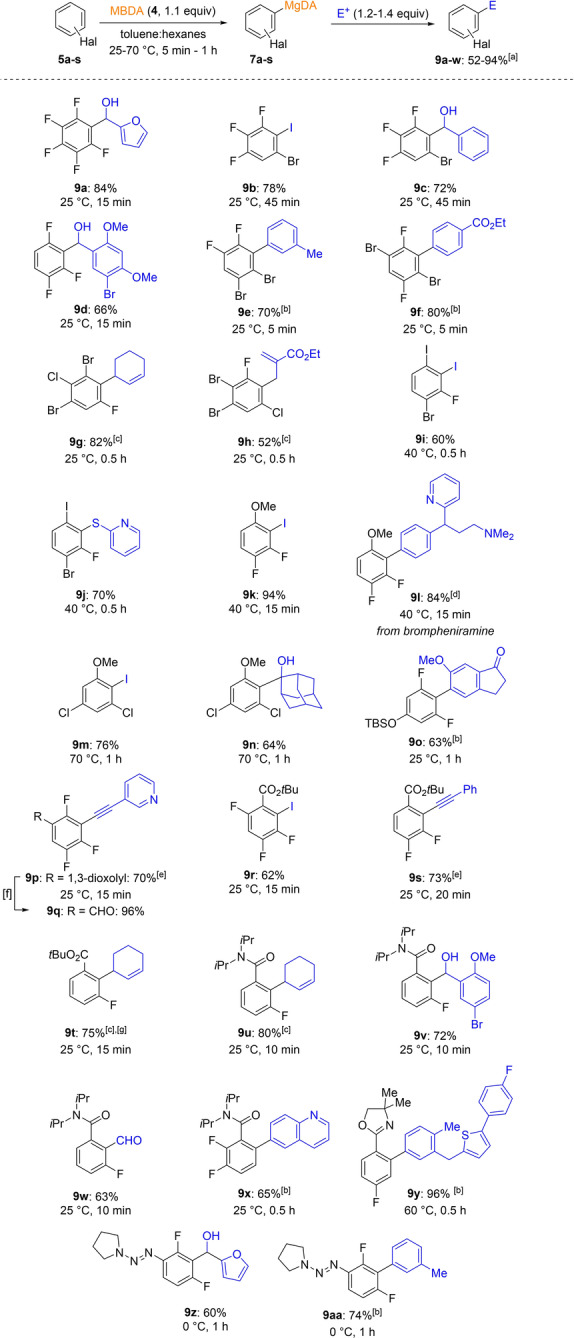
Regioselective magnesiation of fluorinated arenes **5 a**–**s** with MBDA **4** leading to arylmagnesium species **7 a**–**s** and after electrophile trapping to functionalized arenes **9 a**–**aa**. a) All yields refer to isolated compounds. b) Obtained after transmetalation with ZnCl_2_ (1.4 equiv) and a palladium‐catalyzed cross‐coupling with an aryl iodide (0.83 equiv) using Pd(dba)_2_ (3 mol %, dba=dibenzylideneacetone), tfp (6 mol %, tfp=tri‐(2‐furyl)‐phosphine).^[17]^ c) The reaction was catalyzed by CuCN⋅2 LiCl (20 mol %).^[18]^ d) Obtained after transmetalation with ZnCl_2_ (1.4 equiv) and a palladium‐catalyzed cross‐coupling with an aryl bromide (0.83 equiv) using [PdCl_2_(dppf)] (5 mol %). e) Obtained after transmetalation with ZnCl_2_ (1.4 equiv), subsequent iodine quench (1.1 equiv) and Sonogashira cross‐coupling with an alkyne (1.3 equiv) using CuI (4 mol %), Pd(dba)_2_ (3 mol %), tfp(6 mol %).^[19]^ f) Reaction conditions: conc. HCl, THF:H_2_O, 25 °C, 0.5 h. g) 3 equiv of THF were added.

MBDA was an excellent base for the metalation of heterocycles. The formation of a *bis*‐heteroaryl magnesium intermediate of type **8** was performed in most cases using 0.6–0.8 equivalents of MBDA **4**. Various trapping reactions with iodine, allylic bromides, aryl iodides (Negishi cross‐coupling), ketones, aldehydes and alkynes (Sonogashira cross‐coupling) provided a range of fluorinated or halogenated heterocycles **10 a**–**r** in 60–96 % yield (Scheme 3). Thus, fluoropyridines **6 a**–**d**, polyfluorinated quinoline **6 e**, 2‐chloropyrazine **6 f**, 2,6‐dichloropyrazine **6 g** as well as thiomethylpyrazine **6 h** were magnesiated between −25 and 25 °C within a few minutes.

**Scheme 3:**
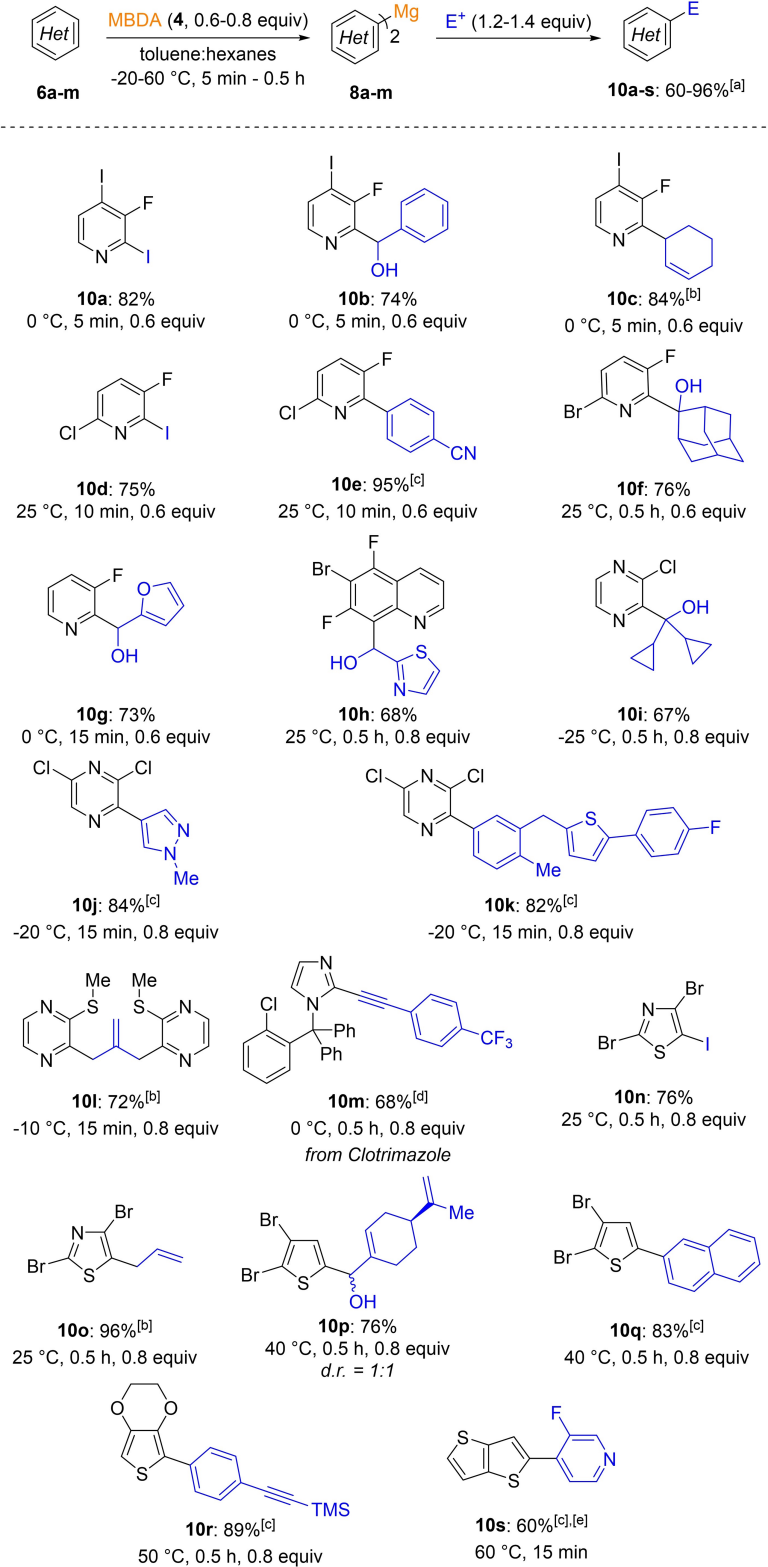
Regioselective magnesiation of heteroarenes **6 a**–**m** with MBDA **4** leading to diheteroarylmagnesium species **8 a**–**m** and after electrophile trapping to functionalized arenes **10 a**–**s**. a) All yields refer to isolated compounds. b) The reaction was catalyzed by CuCN⋅2 LiCl (20 mol %). c) Obtained after transmetalation with ZnCl_2_ (1.4 equiv) and a palladium‐catalyzed cross‐coupling with an aryl iodide (0.83 equiv) using Pd(dba)_2_ (3 mol %), tfp (6 mol %). d) Obtained after transmetalation with ZnCl_2_ (1.4 equiv), subsequent iodine quench (1.1 equiv) and Sonogashira cross‐coupling with an alkyne (1.3 equiv) using CuI (4 mol %), Pd(dba)_2_ (3 mol %), tfp (6 mol %). e) 1.1 equiv of MBDA **4** were used.

Quenching with typical electrophiles afforded the expected products in 67–96 % yield. Five‐membered heterocycles such as the antifungal drug clotrimazole **6 i**, 2,4‐dibromothiazole **6 j**, 2,3‐dibromothiophene **6 k** or 3,4‐ethylenedioxythiophene **6 l** were magnesiated between 25 °C and 50 °C giving the expected diheteroarylmagnesium derivatives of type **8** which after electrophile quench provided the heterocycles **10 m**–**r** in 68–96 % yield. Finally, in the case of thieno[3,2‐*b*]thiophene **6 m** the magnesiation required 1.1 equivalents of MBDA (**4**). Pd‐catalyzed cross‐coupling with 4‐iodo‐3‐fluoropyridine **6 a** afforded the product **10 s** in 60 % yield.

Some products of type **9** (Scheme 2) were readily post‐functionalized furnishing more complex fluorinated molecules (Scheme 4). Thus, the benzoate **9 s** underwent a ring closure with ICl leading to the fluorinated isocoumarine **11 a** in 94 % yield.^[22]^ Also, the aryl oxazoline **9 y** was converted to the corresponding fluoronitrile **11 b** under Vilsmeier–Haack conditions in 95 % yield.^[23]^ Finally, the triazene^[24]^ **9 aa** gave the key aryl azide **11 c** by treatment with BF_3_⋅OEt_2_, trifluoroacetic acid (TFA) and sodium azide which by click‐reaction with trimethylsilylacetylene afforded the triazole **11 d** in 95 % yield.^[25]^ Reduction of **11 c** with SnCl_2_ furnished the difluoroaniline **11 e** in 95 % yield.^[26]^

**Scheme 4:**
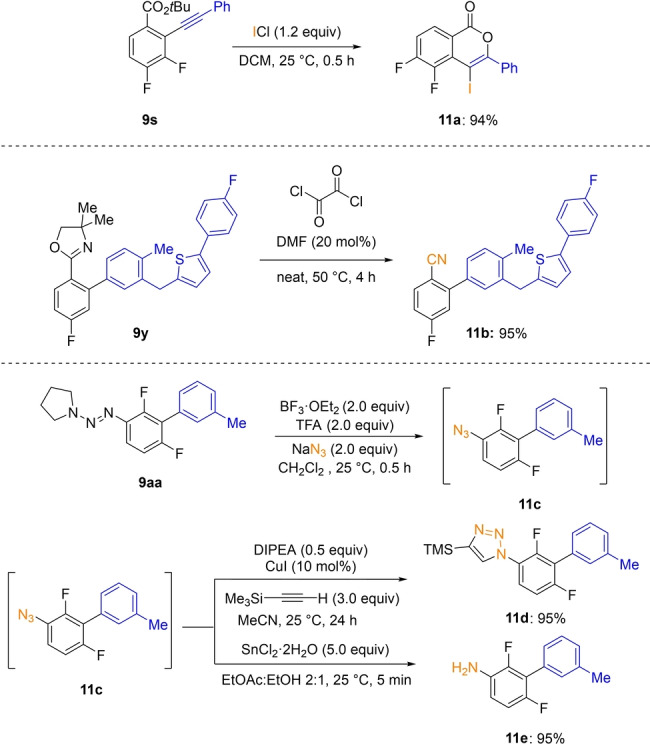
Postfunctionalizations of fluoroarenes **9 s**, **9 y** and **9 aa** providing highly functionalized fluoroarenes.

Furthermore, ^1^H‐ and ^13^C NMR studies revealed a dimeric structure of MBDA (**4**) in toluene‐*d_8_* as shown by a typical pattern showing two sets of signals (Scheme 5).^[27]^ Also, ^1^H‐ and ^19^F NMR spectra of ArMgDA confirmed the expected stoichiometry for arenes by addition of 1.1 equivalents of MBDA (**4**).^[28]^

**Scheme 5:**
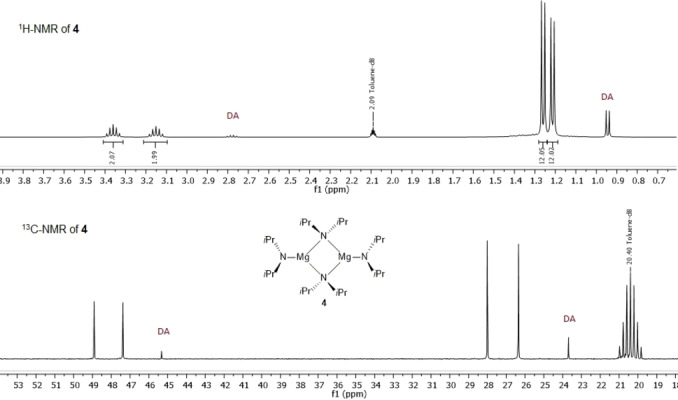
^1^H and ^13^C NMR spectra of MBDA (**4**) in toluene‐*d*_*8*._

In summary, we have reported a new hydrocarbon soluble base MBDA (**4**) that allowed a non‐cryogenic magnesiation of various fluoroarenes (**5**) and heterocyclic fluoro‐derivatives **6**. The resulting organomagnesium intermediates of type ArMgDA **7** or Het_2_Mg **8** reacted with various electrophiles. Further studies on the chemical behavior of these organomagnesium species in hydrocarbon solvents are currently underway.

## Conflict of interest

The authors declare no conflict of interest.

## Supporting information

As a service to our authors and readers, this journal provides supporting information supplied by the authors. Such materials are peer reviewed and may be re‐organized for online delivery, but are not copy‐edited or typeset. Technical support issues arising from supporting information (other than missing files) should be addressed to the authors.

Supporting Information

## Data Availability

The data that support the findings of this study are available in the Supporting Information of this article.
