# Supplementary material for: Regioselective Magnesiations of Fluorinated Arenes and Heteroarenes Using Magnesium‐bis‐Diisopropylamide (MBDA) in Hydrocarbons
Source: Angew Chem Int Ed Engl. 2022 Jun 1;61(29):e202206176. doi: 10.1002/anie.202206176 (PMC9401567; doi:10.1002/anie.202206176)
Supplement: Supplementary file 1 — Supporting Information [file ANIE-61-0-s001.pdf]

Angewandte Chemie
Eine Zeitschrift der Gesellschaft Deutscher Chemiker

Supporting Information

Regioselective Magnesiations of Fluorinated Arenes and Heteroarenes Using Magnesium-*bis*-Diisopropylamide (MBDA) in Hydrocarbons

*A. Hess, N. Alandini, Y. C. Guersoy, P. Knochel**

Supporting Information

Table of Content

1. General Information 2
2. Reagents 4
3. NMR Studies 5
4. Typical Procedures 8
5. Starting Materials 9
6. Preparation of Compounds 19
7. Postfunctionalizations 65
8. X-Ray Crystal Structure Data 69
9. NMR Spectra 71

1. General Information

All reactions were carried out under argon or nitrogen atmosphere in glassware dried with a heat gun (650 °C) under high vacuum (<1 mbar). Syringes which were used to transfer anhydrous solvents or reagents were purged thrice with argon or nitrogen prior to use. Indicated yields are isolated yields of compounds estimated to be >95% pure as determined by ^{1}H-NMR (25 °C) and capillary GC. Unless otherwise indicated, all reagents were obtained from commercial sources.

Solvents

Toluene was continuously refluxed and freshly distilled from sodium under nitrogen and stored over molecular sieves. THF was continuously refluxed and freshly distilled from sodium benzophenone ketyl under nitrogen and stored over molecular sieves. Solvents for flash column chromatography were distilled prior to use.

Chromatography

Flash column chromatography was performed using SiO_2 60 (0.040-0.063 mm, 230-400 mesh ASTM) from Merck. Thin layer chromatography (TLC) was performed using aluminum plates covered with SiO_2 (Merck 60, F-254). Spots were visualized under UV light.

Analytical Data

^{1}H-NMR and **^{13}C-NMR** spectra were recorded on VARIAN Mercury 200, BRUKER ARX 300, VARIAN VXR 400 S and BRUKER AMX 600 instruments. Chemical shifts are reported as values in ppm relative to tetramethylsilane. $CDCl_3$ peaks were set to 7.26 ppm in ^{1}H NMR and 77.16 ppm in ^{13}C NMR experiments. The following abbreviations were used to characterize signal multiplicities: s (singlet), d (doublet), dd (doublet of doublets),t (triplet),q (quartet), hept (heptett)as well as m (multiplet).

Mass spectroscopy: High resolution (HRMS) and low resolution (MS) spectra were recorded on a FINNIGAN MAT 95Q instrument. Electron impact ionization (EI) was conducted with an ionization energy of 70 eV. For coupled gas chromatography/mass spectrometry, a HEWLETT-PACKARD HP 6890/MSD 5973 GC/MS system was used. Molecular fragments are reported starting at a relative intensity of 10-20%.

2. Reagents

All reagents were obtained from commercial sources and used without further purification unless otherwise stated.

Bu_2Mg solution in hexanes was purchased from Albemarle and the concentration was determined by titration using benzoic acid and 4-(phenylazo)diphenylamine as indicator.

Magnesium bis-diisopropylamide 4 (MBDA): A dry and argon-flushed Schlenk-flask equipped with a stirring bar and a septum, was charged with Bu_2Mg (67.1 mL, 50 mmol, 0.75 M in hexanes, 1.00 equiv). Then diisopropylamine (14.3 mL, 101 mmol, 2.02 equiv) was added dropwise at 0 °C. The reaction mixture was allowed to warm to room temperature and was stirred for 4 h. The slightly yellow solution was titrated (benzoic acid and 4-(phenylazo)diphenylamine as indicator, 0.79 M, >99% yield) prior use.

CuCN·2LiCl: A CuCN·2LiCl solution (1.00 M) was prepared by drying CuCN (80.0 mmol, 7.17 g) and LiCl (160 mmol, 6.77 g) in a Schlenk-flask under vacuum at 140 °C for 12 h. After cooling, dry THF (80 mL) was added and stirring was continued until the salts were dissolved.[1]

$ZnCl_2$: A $ZnCl_2$ solution (1.00 M) was prepared by drying $ZnCl_2$ (200 mmol, 27.3 g) in a Schlenk-flask under vacuum at 140 °C for 5 h. After cooling, dry THF (200 mL) was added and stirring continued until the salt was dissolved.

[1] P. Knochel, M. C. P. Yeh, S. C. Berk, J. Talbert, *J. Org. Chem.* **1988**, *53*, 2390-2392.

3. NMR studies

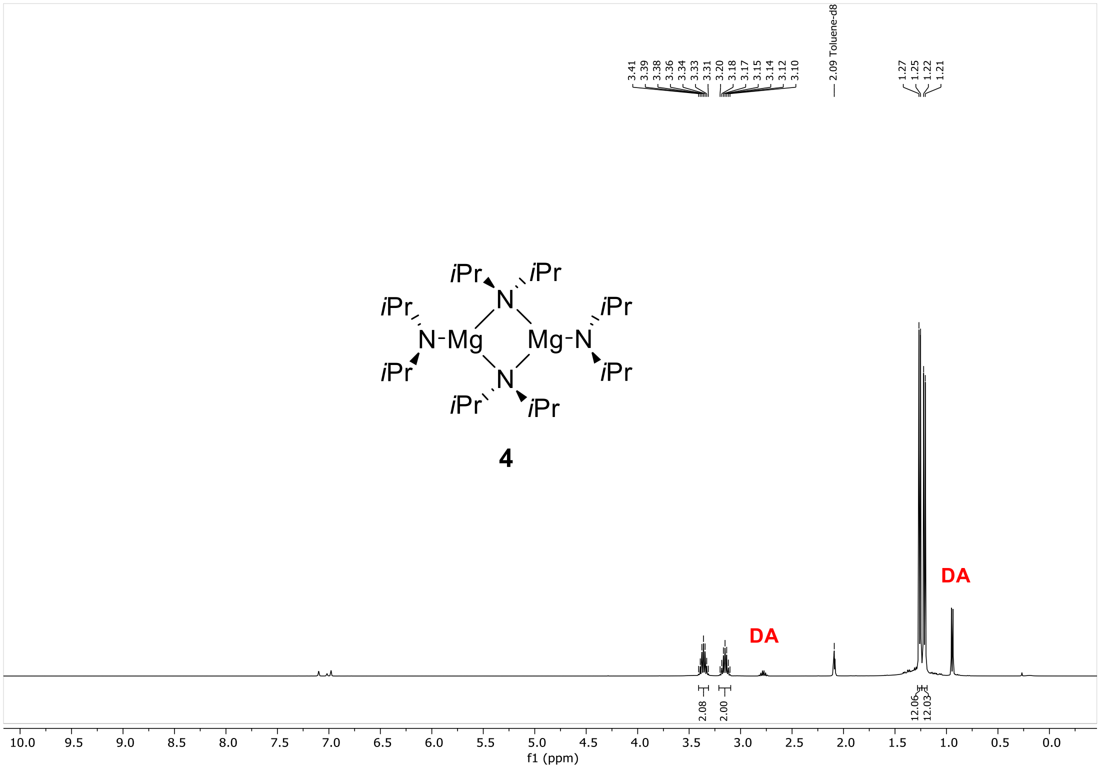

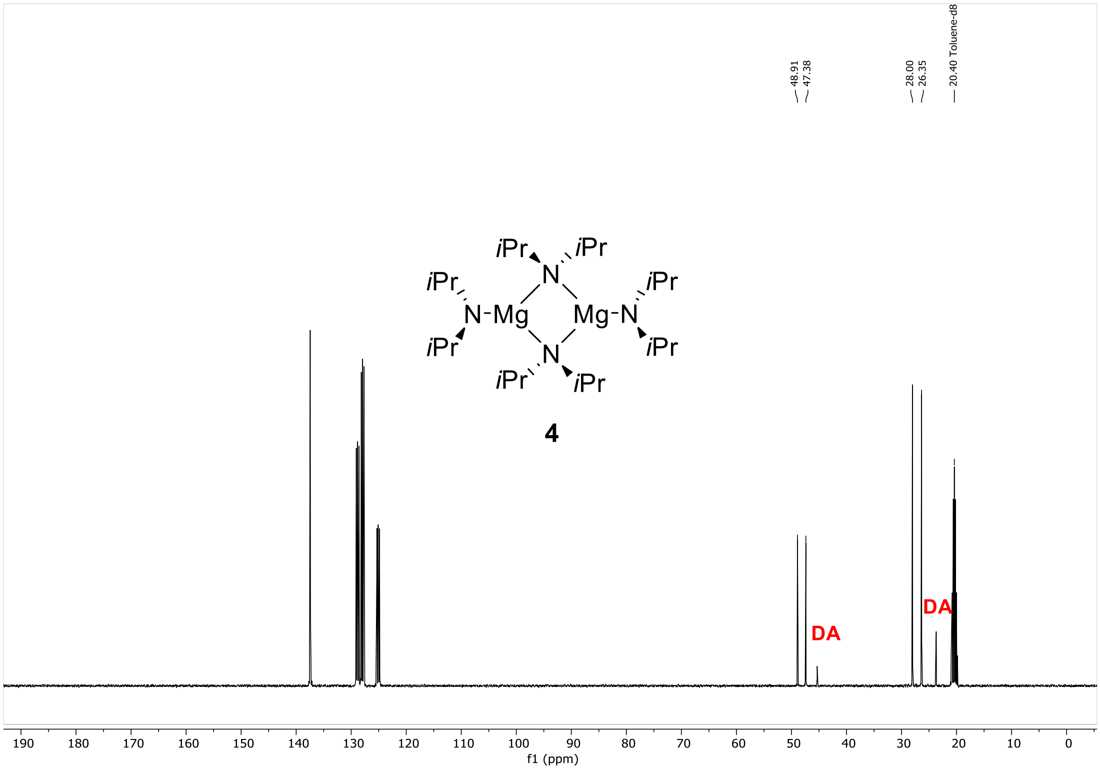

^{1}H NMR (400 MHz, tol-d$_8$): δ (ppm) = 3.36 (hept, J = 6.3 Hz, 2H), 3.15 (hept, J = 6.4 Hz, 2H), 1.26 (d, J = 6.3 Hz, 12H), 1.21 (d, J = 6.4 Hz, 12H).

^{13}C NMR (101 MHz, tol-d$_8$): δ (ppm) = 48.9, 47.4, 28.0, 26.3.

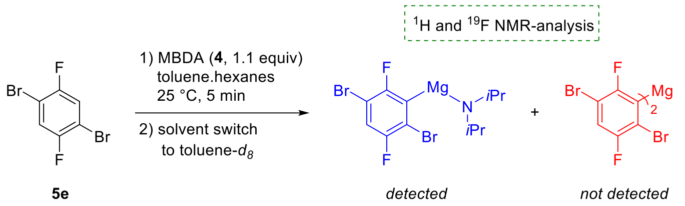

^{1}H NMR-spectra:

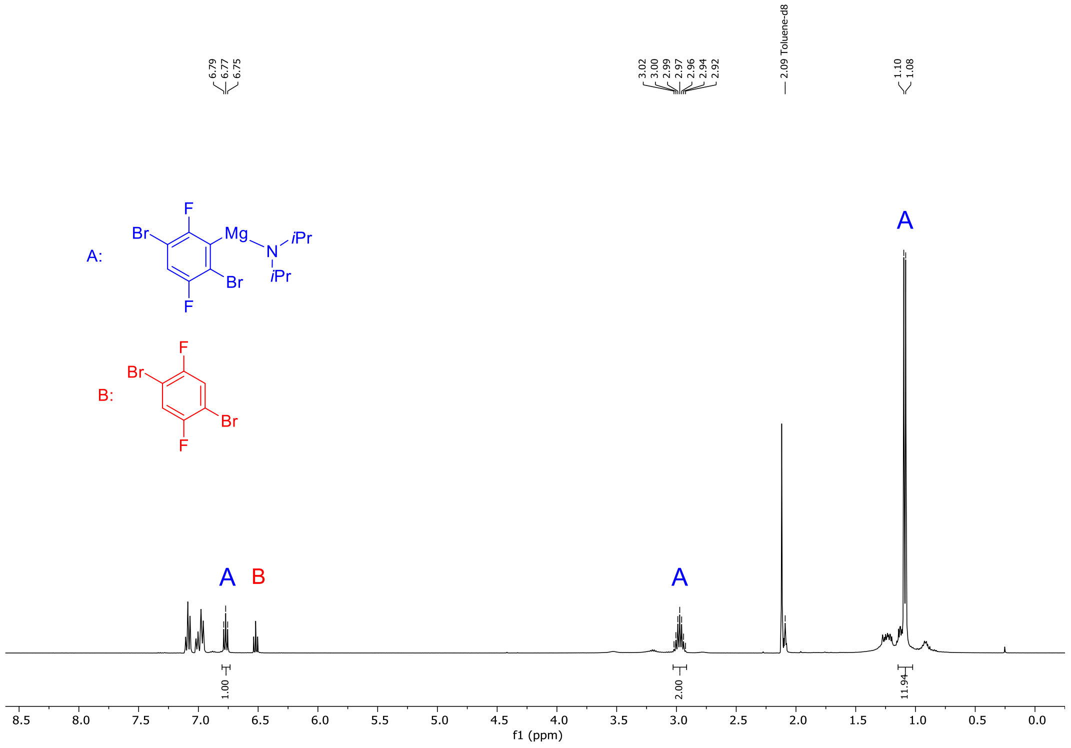

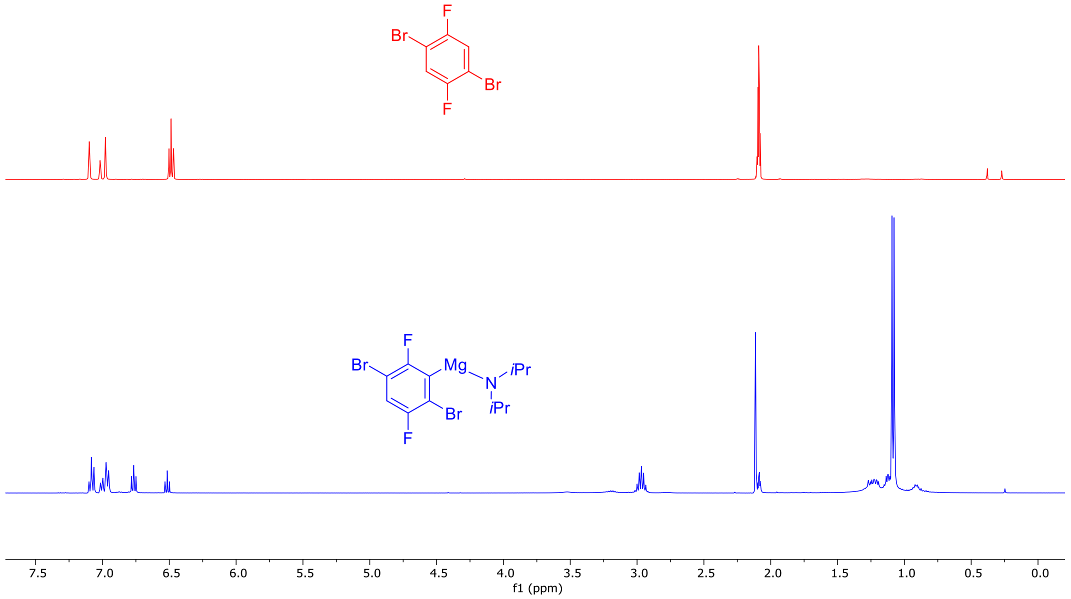

^{19}F NMR-spectra:

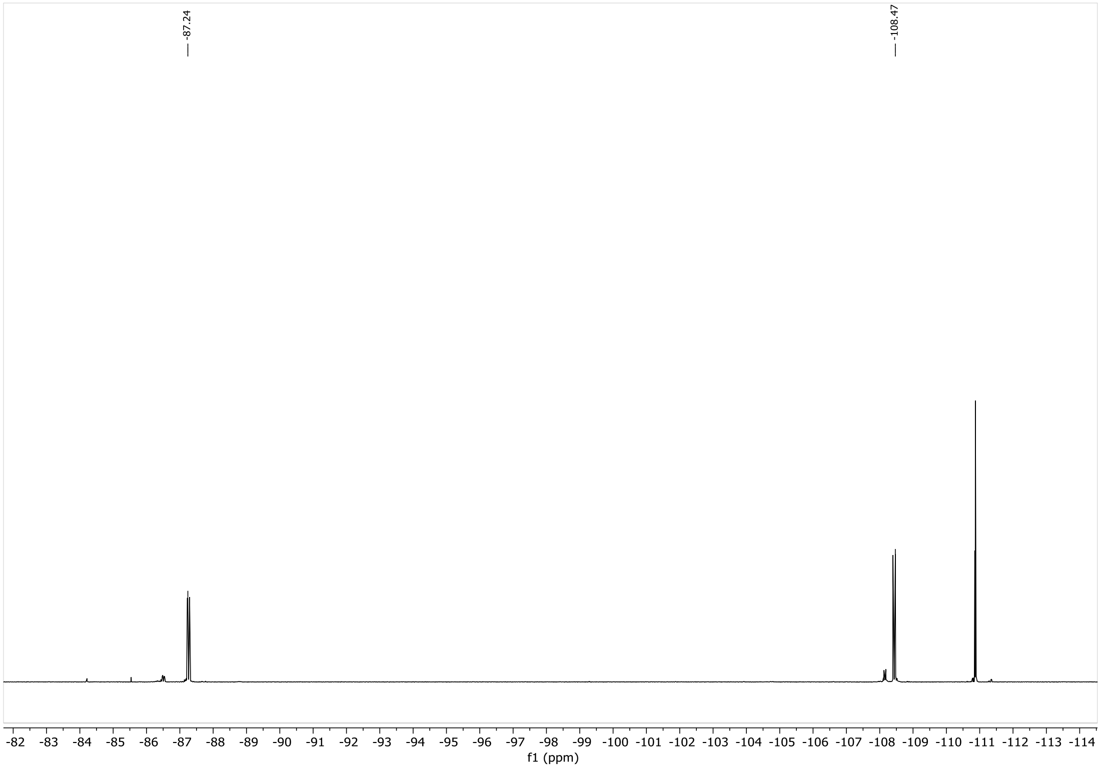

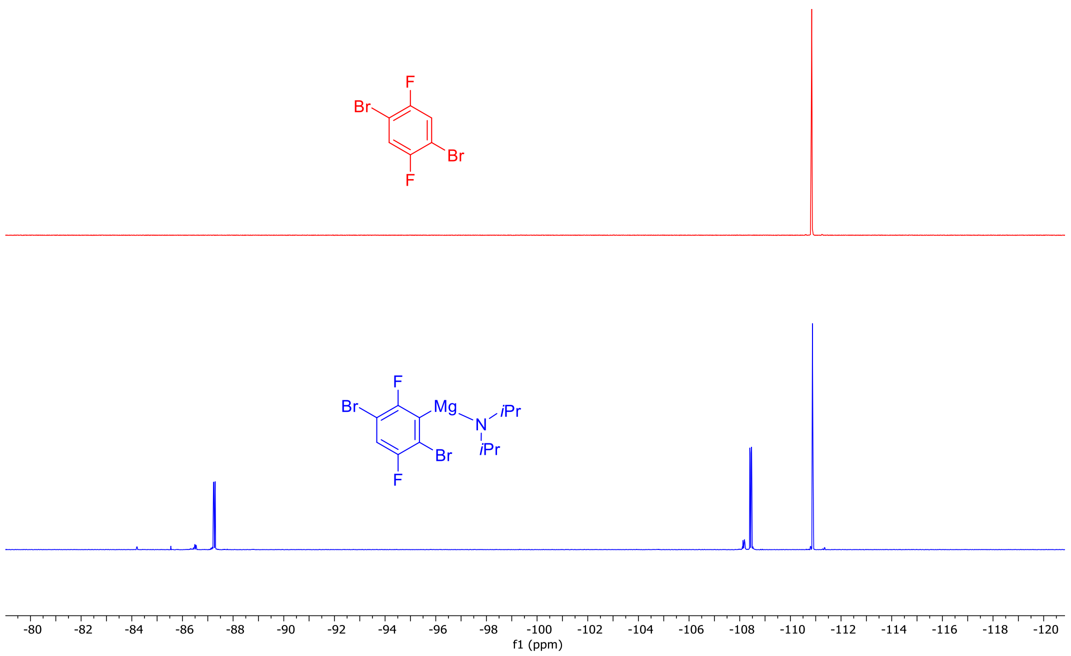

4. Typical Procedures

Typical Procedure 1 (TP1): Regioselective metalation and functionalization of fluorinated arenes and heteroarenes using MBDA

A dry and argon flushed Schlenk-tube, equipped with a magnetic stirring bar and a septum, was charged with the corresponding starting material (1.0 equiv) in dry toluene (0.5 M solution). The resulting solution was stirred at indicated temperature and MBDA (0.60-1.10 equiv) was added dropwise. The completion of the metalation was checked by GC-analysis of reaction aliquots quenched with iodine, using undecane as internal standard. Subsequent reactions with electrophiles (1.2-1.4 equiv) were carried out under the indicated conditions. After complete conversion, the mixture was quenched with sat. aq. NH_4Cl solution and extracted with ethyl acetate (3 x 10 mL). The combined organic extracts were dried over $MgSO_4$, filtered and concentrated. Purification of the crude product by flash column chromatography using an indicated eluent afforded the corresponding title compounds.

5. Starting Materials

Overview

Arenes:

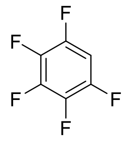

5a

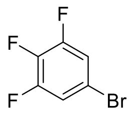

5b

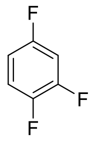

5c

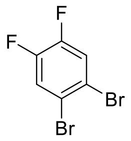

5d

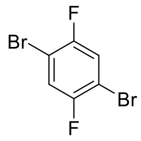

5e

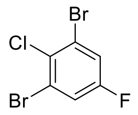

5f

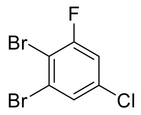

5g

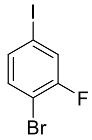

5h

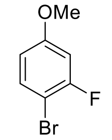

5i

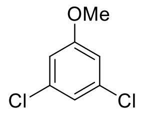

5j

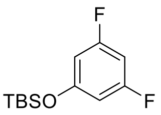

5k

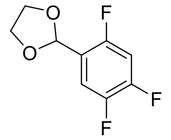

5l

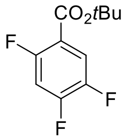

5m

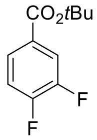

5n

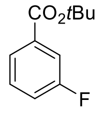

5o

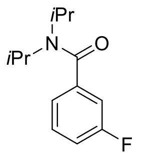

5p

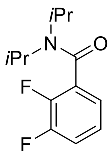

5q

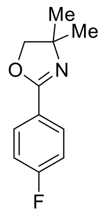

5r

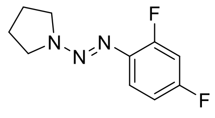

5s

Heteroarenes:

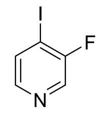

6a

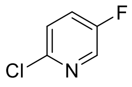

6b

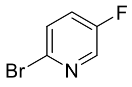

6c

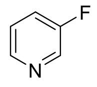

6d

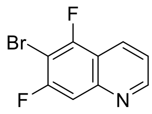

6e

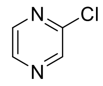

6f

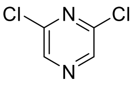

6g

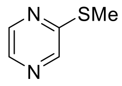

6h

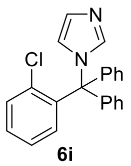

6i

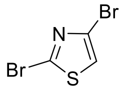

6j

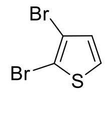

6k

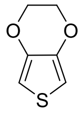

6l

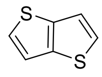

6m

Preparation of Starting Materials:

***Tert-butyl*(3,5-difluorophenoxy)dimethylsilane (5k)**

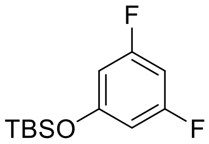

3,5-difluorophenol (4.00 g, 30.70 mmol, 1.0 equiv) and imidazole (4.18 g, 61.40 mmol, 2.2 equiv) were dissolved in DMF (40 mL). The reaction mixture was cooled to 0 °C and TBS-Cl (5.09 g, 33.80 mmol, 1.1 equiv) was added in portions. The reaction mixture was allowed to warm to room temperature and stirred overnight. The reaction mixture was quenched by addition of water and extracted with DCM (3 x 30 mL). The combined organic layers were washed with water (2 x 20 mL) and brine (20 mL), dried over $MgSO_4$ and the solvent was removed *in vacuo*. Purification of the crude product by flash column chromatography (silica gel, *i*hexane/EtOAc = 199:1) afforded the title compound as a colorless oil (**5k**, 6.00 g, 24.56 mmol, 80% yield).

^{1}H NMR (400 MHz, $CDCl_3$): δ (ppm) = 6.43 (tt, *J* = 9.1, 2.3 Hz, 1H), 6.39–6.32 (m, 2H), 0.97 (s, 9H), 0.22 (s, 6H).

^{13}C NMR (101 MHz, $CDCl_3$): δ (ppm) = 163.6 (dd, *J* = 246.4, 16.1 Hz), 157.8 (t, *J* = 14.1 Hz), 104.1–103.7 (m), 97.3 (t, *J* = 25.9 Hz), 25.7, 18.3, -4.4.

^{19}F NMR (376 MHz, $CDCl_3$): δ (ppm) = -110.2.

MS (EI, 70 eV): m/z (%) = 205 (21), 187 (100), 80 (11).

HRMS (EI): for $C_{12}H_{18}F_2OSi$: calc. [M+]: 244.1095; found: 244.1088.

2-(2,4,5-Trifluorophenyl)-1,3-dioxolane (5l)

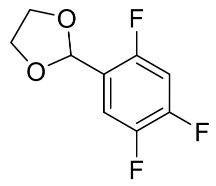

2,4,5-trifluorobenzaldehyde (3.16 mL, 28.00 mmol, 1.0 equiv), ethylene glycol (1.87 mL, 33.60 mmol, 1.2 equiv) and TsOH·H_2O (107 mg, 2 mol%) were dissolved in toluene (40 mL) and the reaction mixture was refluxed overnight. After cooling to room temperature, water (20 mL) was added and the reaction mixture was extracted with DCM (3 x 30 mL). The combined organic layers were dried over $MgSO_4$ and the solvent was removed *in vacuo*. Purification of the crude product by flash column chromatography (silica gel, *i*hexane/EtOAc = 96:4) afforded the title compound as a white solid (**5l**, 3.474 g, 17.0 mmol, 61% yield).

^{1}H NMR (400 MHz, $CDCl_3$): δ (ppm) = 7.37 (ddd, *J* = 10.4, 8.9, 6.4 Hz, 1H), 6.94 (td, *J* = 9.7, 6.3 Hz, 1H), 6.01 (d, *J* = 1.3 Hz, 1H), 4.18–4.09 (m, 2H), 4.09–4.00 (m, 2H).
^{13}C NMR (101 MHz, $CDCl_3$): δ (ppm) = 156.2 (ddd, *J* = 248.6, 9.7, 2.7 Hz), 150.7 (ddd, *J* = 252.7, 14.6, 12.4 Hz), 146.9 (ddd, *J* = 245.2, 12.6, 3.7 Hz), 122.0 (dt, *J* = 14.7, 4.5 Hz), 115.9 (ddd, *J* = 20.4, 5.4, 1.7 Hz), 105.9 (dd, *J* = 27.2, 21.1 Hz), 97.8 (d, *J* = 3.1 Hz), 65.6.
^{19}F NMR (376 MHz, $CDCl_3$): δ (ppm) = -121.1, -132.3, -142.3.
MS (EI, 70 eV): m/z (%) = 203 (80), 185 (100), 159 (97), 144 (46), 125 (19).
HRMS (EI): for $C_9H_7F_3O_2$: calc. [M+]: 204.0398; found: 204.0391.

***Tert-butyl* 2,4,5-trifluorobenzoate (5m)**

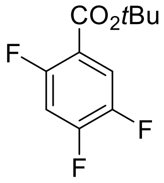

2,4,5-trifluorobenzoyl chloride (2.94 mL, 23.10 mmol, 1.0 equiv) was dissolved in dry DCM (40 mL). The reaction mixture was cooled to 0 °C and KO*t*Bu (2.851 g, 25.41 mmol, 1.1 equiv) was added in portions. The mixture was stirred for 1 h and then allowed to warm to room temperature and was stirred overnight. The reaction mixture was quenched by addition of water (20 mL) and was extracted with DCM (3 x 30 mL). The combined organic layers were dried over $MgSO_4$ and the solvent was removed *in vacuo*. Purification of the crude product by flash column chromatography (silica gel, *i*hexane/EtOAc = 94:6) afforded the title compound as a colorless oil (**5m**, 4.364 g, 18.79 mmol, 81% yield).
The analytical data were consistent with literature values.[2]

^{1}H NMR (400 MHz, $CDCl_3$): δ (ppm) = 7.71 (ddd, *J* = 10.5, 8.9, 6.6 Hz, 1H), 6.96 (td, *J* = 9.8, 6.2 Hz, 1H), 1.58 (s, 9H).
^{13}C NMR (101 MHz, $CDCl_3$): δ (ppm) = 161.7 (d, *J* = 4.5 Hz), 157.8 (ddd, *J* = 259.4, 9.9, 2.7 Hz), 152.8 (ddd, *J* = 257.9, 14.4, 12.0 Hz), 146.4 (ddd, *J* = 246.3, 12.7, 3.9 Hz), 120.0 (dt, *J* = 20.3, 2.3 Hz), 117.0 (dt, *J* = 11.6, 4.5 Hz), 107.0 (dd, *J* = 28.7, 20.8 Hz), 82.9, 28.2.

[2] M. Al-Masum, A. Hira, S. Chrisman, N. T. Nguyen, *Tetrahedron Lett.* **2019**, *60*, art. no. 150936.

***Tert-butyl* 3,4-difluorobenzoate (5n)**

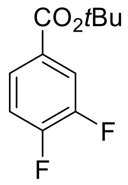

3,4-difluorobenzoyl chloride (2.30 mL, 20.00 mmol, 1.0 equiv) was dissolved in dry DCM (40 mL). The reaction mixture was cooled to 0 °C and KO*t*Bu (2.360 g, 22.00 mmol, 1.1 equiv) was added in portions. The mixture was stirred for 1 h and then allowed to warm to room temperature and was stirred overnight. The reaction mixture was quenched by addition of water (20 mL) and was extracted with DCM (3 x 30 mL). The combined organic layers were dried over $MgSO_4$ and the solvent was removed *in vacuo*. Purification of the crude product by flash column chromatography (silica gel, *i*hexane/EtOAc = 98:2) afforded the title compound as a colorless oil (**5n**, 3.724 g, 17.40 mmol, 87% yield).

^{1}H NMR (400 MHz, $CDCl_3$): δ (ppm) = 7.83–7.73 (m, 2H), 7.23–7.15 (m, 1H), 1.59 (s, 9H).

^{13}C NMR (101 MHz, $CDCl_3$): δ (ppm) = 163.9, 153.4 (dd, *J* = 255.0, 12.7 Hz), 150.1 (dd, *J* = 249.3, 13.0 Hz), 129.2 (dd, *J* = 5.4, 3.6 Hz), 126.4 (dd, *J* = 7.3, 3.7 Hz), 118.9 (dd, *J* = 18.4, 1.6 Hz), 117.2 (d, *J* = 17.8 Hz), 82.0, 28.3.

^{19}F NMR (376 MHz, $CDCl_3$): δ (ppm) = -131.4, -137.1.

MS (EI, 70 eV): m/z (%) = 159 (12), 141 (100), 63 (16).

HRMS (EI): for $C_{11}H_{12}F_2O_2$: calc. [M+]: 214.0805; found: 214.0815.

***Tert-butyl* 3-fluorobenzoate (5o)**

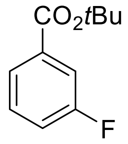

3-fluorobenzoyl chloride (4.20 mL, 35.00 mmol, 1.0 equiv) was dissolved in dry DCM (50 mL). The reaction mixture was cooled to 0 °C and KO*t*Bu (4.000 g, 38.50 mmol, 1.1 equiv) was added in portions. The mixture was stirred for 1 h and then allowed to warm to room temperature and was stirred overnight. The reaction mixture was quenched by addition of water (20 mL) and was extracted with DCM (3 x 30 mL). The combined organic layers were dried over $MgSO_4$ and the solvent was removed *in vacuo*. Purification of the crude product by flash column chromatography (silica gel, *i*hexane/EtOAc = 94:6) afforded the title compound as a colorless oil (**5o**, 5.235 g, 26.68 mmol, 76% yield).
The analytical data were consistent with literature values.[3]

^{1}H NMR (400 MHz, $CDCl_3$): δ (ppm) = 7.78 (dt, *J* = 7.7, 1.3 Hz, 1H), 7.66 (ddd, *J* = 9.5, 2.7, 1.5 Hz, 1H), 7.38 (td, *J* = 8.0, 5.6 Hz, 1H), 7.21 (tdd, *J* = 8.3, 2.7, 1.1 Hz, 1H), 1.59 (s, 9H).
^{13}C NMR (101 MHz, $CDCl_3$): δ (ppm) = 164.7 (d, *J* = 2.9 Hz), 162.6 (d, *J* = 246.5 Hz), 134.4 (d, *J* = 7.3 Hz), 129.9 (d, *J* = 7.7 Hz), 125.2 (d, *J* = 3.0 Hz), 119.6 (d, *J* = 21.3 Hz), 116.4 (d, *J* = 22.9 Hz), 81.7, 28.3.

[3] S. W. Wright, D. L. Hagemann, A. S. Wright, L. D. McClure, *Tetrahedron Lett.* **1997**, *38*, 7345-7348.

3-Fluoro-*N*,*N*-diisopropylbenzamide (5p)

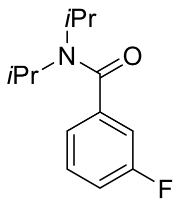

3-fluorobenzoyl chloride (2.40 mL, 20.00 mmol, 1.0 equiv) and Et_3N (3.90 mL, 28.00 mmol, 1.4 equiv) were dissolved in dry DCM (40 mL). The reaction mixture was cooled to 0 °C and diisopropylamine (3.96 mL, 28.00 mmol, 1.4 equiv) was added dropwise. The mixture was stirred for 1 h and then allowed to warm to room temperature and was stirred overnight. The reaction mixture was quenched by addition of water (20 mL) and was extracted with DCM (3 x 30 mL). The combined organic layers were dried over $MgSO_4$ and the solvent was removed *in vacuo*. Purification of the crude product by flash column chromatography (silica gel, *i*hexane/EtOAc = 9:1) afforded the title compound as a colorless oil (**5p**, 4.437 g, 19.90 mmol, quant. yield).

The analytical data were consistent with literature values.[4]

^{1}H NMR (400 MHz, $CDCl_3$): δ (ppm) = 7.35 (td, *J* = 7.9, 5.6 Hz, 1H), 7.12–6.97 (m, 3H), 3.65 (d, *J* = 92.0 Hz, 2H), 1.83–0.90 (m, 12H).

^{13}C NMR (101 MHz, $CDCl_3$): δ (ppm) = 169.6 (d, *J* = 2.3 Hz), 162.7 (d, *J* = 247.8 Hz), 141.0 (d, *J* = 6.6 Hz), 130.4 (d, *J* = 8.1 Hz), 121.4 (d, *J* = 3.2 Hz), 115.8 (d, *J* = 21.1 Hz), 113.1 (d, *J* = 22.5 Hz), 20.8.

[4] L. Wang, L. Ackermann, *Chem. Comm.* **2014**, *50*, 1083-1085.

2,3-Difluoro-*N*,*N*-diisopropylbenzamide (5q)

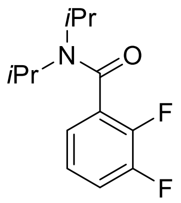

2,3-difluorobenzoyl chloride (2.48 mL, 20.00 mmol, 1.0 equiv) and Et_3N (3.90 mL, 28.00 mmol, 1.4 equiv) were dissolved in dry DCM (40 mL). The reaction mixture was cooled to 0 °C and diisopropylamine (3.96 mL, 28.00 mmol, 1.4 equiv) was added dropwise. The mixture was stirred for 1 h and then allowed to warm to room temperature and was stirred overnight. The reaction mixture was quenched by addition of water (20 mL) and was extracted with DCM (3 x 30 mL). The combined organic layers were dried over $MgSO_4$ and the solvent was removed *in vacuo*. Purification of the crude product by flash column chromatography (silica gel, *i*hexane/EtOAc = 9:1) afforded the title compound as a colorless oil (**5q**, 4.236 g, 17.56 mmol, 88% yield).

^{1}H NMR (400 MHz, $CDCl_3$): δ (ppm) = 7.19–7.07 (m, 2H), 7.01 (ddt, *J* = 8.6, 5.3, 1.7 Hz, 1H), 3.71 (pd, *J* = 6.7, 1.3 Hz, 1H), 3.54 (hept, *J* = 6.8 Hz, 1H), 1.55 (d, *J* = 6.8 Hz, 6H), 1.29–1.02 (m, 6H).
^{13}C NMR (101 MHz, $CDCl_3$): δ (ppm) = 164.4, 150.5 (dd, *J* = 238.4, 13.0 Hz), 129.1 (dd, *J* = 235.7, 14.8 Hz), 129.1 (d, *J* = 16.1 Hz), 125.1 (dd, *J* = 6.6, 4.4 Hz), 122.6 (t, *J* = 3.5 Hz), 117.5 (d, *J* = 17.1 Hz), 51.4, 46.4, 20.7 (t, *J* = 29.1 Hz).
^{19}F NMR (376 MHz, $CDCl_3$): δ (ppm) = -136.9, -141.5.
MS (EI, 70 eV): m/z (%) = 241 (11), 226 (11), 198 (19), 184 (16), 141 (100).
HRMS (EI): for $C_{13}H_{17}F_2NO$: calc. [M^+]: 241.1278; found: 241.1274.

2-(4-Fluorophenyl)-4,4-dimethyl-4,5-dihydrooxazole (5r)

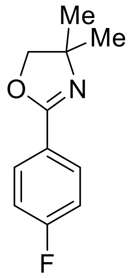

4-fluorobenzaldehyde (2.60 mL, 25.00 mmol, 1.0 equiv) and 2-amino-2-methylpropan-1-ol (2.80 mL, 30.00 mmol, 1.2 equiv) were dissolved in dry CH_2Cl_2 (40 mL), to which molecular sieves 4 A (2 g) were added. The mixture was stirred for 23 h at room temperature. The flask was cooled to 0 °C and NBS (9.08 g, 45.4 mmol, 1.8 equiv) was added in portions. The mixture was stirred for 10 min at 0 °C, the cooling bath was removed, and stirring was continued for 3 h at 25 °C. The mixture was filtered and the solid residue was washed with EtOAc (150 mL) and sat. NaHCO3 (100 mL) solution. The phases were separated and the aqueous layer was extracted with EtOAc (3 x 50 mL). The combined organic layers were dried over MgSO4, filtered and the solvent was removed in vacuo. Purification of the crude product by flash column chromatography (silica gel, ihexane/EtOAc = 9:1) afforded the title compound as a colorless oil (**5r**, 3.965 g, 20.52 mmol, 82% yield).
The analytical data were consistent with literature values.[5]

^{1}H NMR (400 MHz, $CDCl_3$): δ (ppm) = 7.75–7.67 (m, 1H), 7.62 (ddt, *J* = 10.2, 5.7, 2.7 Hz, 1H), 7.35 (dtt, *J* = 8.3, 5.7, 3.1 Hz, 1H), 7.13 (tdt, *J* = 8.5, 5.3, 2.7 Hz, 1H), 4.14 (s, 2H), 1.36 (s, 6H).
^{13}C NMR (101 MHz, $CDCl_3$): δ (ppm) = 162.6 (d, *J* = 246.0 Hz), 161.1 (d, *J* = 3.2 Hz), 130.3 (d, *J* = 8.3 Hz), 130.0 (d, *J* = 8.0 Hz), 124.0 (d, *J* = 3.1 Hz), 118.2 (d, *J* = 21.2 Hz), 115.3 (d, *J* = 23.5 Hz), 79.4, 67.9, 28.5.

[5] D. T. Witiak, S. Goswami, G. E. Milo, *J. Org. Chem.* **1988**, *53*, 345-352.

(*E*)-1-((2,4-Difluorophenyl)diazenyl)pyrrolidine (5s)

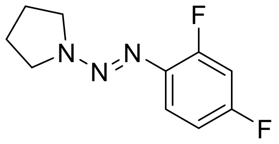

The compound was prepared according to a literature procedure.[6] A solution of 2,4-difluoroaniline (2.582 g, 20.00 mmol, 1.0 equiv) in 4 mL of conc. HCl was cooled in an ice bath while a solution of $NaNO_2$ (1.38 g, 20.00 mmol, 1.0 equiv) in 10 mL of cold water was added dropwise. The resulting solution of the diazonium salt was stirred at 0 °C for 0.5 h and then added at once to a solution of pyrrolidine (1.81 mL, 22.00 mmol, 1.1 equiv) in 1 M KOH (20 mL). The reaction mixture was stirred for 0.5 h at 0 °C and was extracted with DCM (3 x 30 mL). The combined organic layers were dried over $MgSO_4$ and the solvent was removed *in vacuo*. Purification of the crude product by flash column chromatography (silica gel, *i*hexane/EtOAc = 9:1) afforded the title compound as an orange solid (**5s**, 3.259 g, 15.43 mmol, 77% yield).

^{1}H NMR (400 MHz, CDCl$_3$): δ (ppm) = 7.40 (td, *J* = 9.0, 6.2 Hz, 1H), 6.89–6.75 (m, 2H), 3.80 (d, *J* = 75.3 Hz, 4H), 2.12–1.95 (m, 5H).
^{13}C NMR (101 MHz, CDCl$_3$): δ (ppm) = 160.1 (dd, *J* = 245.5, 11.3 Hz), 156.0 (dd, *J* = 251.5, 11.9 Hz), 136.3 (dd, *J* = 8.0, 3.8 Hz), 119.8 (dd, *J* = 9.4, 3.2 Hz), 111.1 (dd, *J* = 21.9, 3.8 Hz), 104.4 (dd, *J* = 25.9, 24.1 Hz), 51.3, 46.6, 23.9.
^{19}F NMR (376 MHz, CDCl$_3$): δ (ppm) = -115.7, -124.2.
MS (EI, 70 eV): m/z (%) = 141 (100), 113 (10), 63 (6).
HRMS (EI): for $C_{10}H_{11}F_2N_3$: calc. [M+]: 211.0921; found: 211.0913.

[6] C.-Y. Liu, P. Knochel, *Org. Lett.* **2005**, *13,* 2543-2546.

6. Preparation of Compounds

Furan-2-yl(perfluorophenyl)methanol (9a)

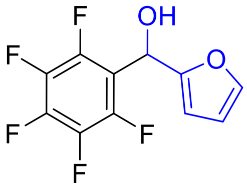

According to **TP 1**, to a mixture of pentafluorobenzene (**5a**, 56 µL, 0.50 mmol, 1.0 equiv) in toluene (1 mL) was added MBDA (0.40 mmol, 0.8 equiv) at 25 °C. After 15 min, the reaction mixture was cooled to 0 °C and furfural (58 µL, 0.70 mmol, 1.4 equiv) was added dropwise and the reaction mixture was stirred for 0.5 h at 0 °C and then allowed to warm to room temperature and stirred for 1 h. Purification of the crude product by flash column chromatography (silica gel, *i*hexane/EtOAc = 9:1) afforded the title compound as a yellow oil (**9a**, 110 mg, 0.42 mmol, 84% yield).

^{1}H NMR (400 MHz, $CDCl_3$): δ (ppm) = 7.40 (dd, *J* = 1.9, 0.8 Hz, 1H), 6.37 (dd, *J* = 3.4, 1.8 Hz, 1H), 6.31 (d, *J* = 3.4 Hz, 1H), 6.16 (d, *J* = 8.3 Hz, 1H), 2.83 (d, *J* = 8.4 Hz, 1H).

^{13}C NMR (101 MHz, $CDCl_3$): δ (ppm) = 152.3, 146.7–143.5 (m), 143.2, 142.7–139.7 (m), 139.3–136.2 (m), 114.6 (t, *J* = 16.4 Hz), 110.8, 107.8 (d, *J* = 1.2 Hz), 63.1–60.8 (m).

^{19}F NMR (376 MHz, $CDCl_3$): δ (ppm) = -143.0, -154.0, -161.4.

MS (EI, 70 eV): m/z (%) = 264 (28), 247 (33), 236 (82), 194 (100), 187 (29), 169 (60), 97 (23).

HRMS (EI): for $C_{11}H_5F_5O_2$: calc. [M+]: 264.0210; found: 264.0217.

1-Bromo-3,4,5-trifluoro-2-iodobenzene (9b)

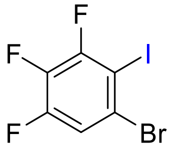

According to **TP 1**, to a mixture of 5-bromo-1,2,3-trifluorobenzene (**5b**, 60 µL, 0.50 mmol, 1.0 equiv) in toluene (1 mL) was added MBDA (0.55 mmol, 1.1 equiv) at 25 °C. After 45 min, the reaction mixture was cooled to 0 °C and iodine (152 mg, 0.60 mmol, 1.2 equiv) dissolved in THF (1 mL) was added dropwise and the reaction mixture was stirred for 1 h. Purification of the crude product by flash column chromatography (silica gel, pentane) afforded the title compound as a colorless liquid (**9b**, 132 mg, 0.39 mmol, 78% yield).

^{1}H NMR (400 MHz, $CDCl_3$): δ (ppm) = 7.42 (ddd, *J* = 9.2, 6.7, 2.3 Hz, 1H).
^{13}C NMR (101 MHz, $CDCl_3$): δ (ppm) = 152.9 (ddd, *J* = 70.2, 10.9, 4.2 Hz), 150.4 (ddd, *J* = 75.7, 11.0, 4.3 Hz), 138.6 (ddd, *J* = 257.1, 18.1, 15.6 Hz), 123.3 (ddd, *J* = 9.2, 5.1, 1.4 Hz), 116.6 (dd, *J* = 20.8, 3.6 Hz), 85.8 (ddd, *J* = 24.6, 4.5, 1.9 Hz).
^{19}F NMR (377 MHz, $CDCl_3$): δ (ppm) = -101.9, -132.1, -156.6.
MS (EI, 70 eV): m/z (%) = 130 (100), 126 (10), 99 (12), 80 (33).
HRMS (EI): for C_6HBrF_3I: calc. [M^+]: 335.8258; found: 335.8248.

(6-Bromo-2,3,4-trifluorophenyl)(phenyl)methanol (9c)

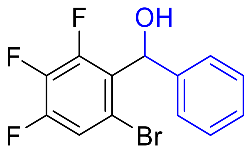

According to **TP 1**, to a mixture of 5-bromo-1,2,3-trifluorobenzene (**5b**, 60 µL, 0.50 mmol, 1.0 equiv) in toluene (1 mL) was added MBDA (0.55 mmol, 1.1 equiv) at 25 °C. After 45 min, the reaction mixture was cooled to 0 °C and benzaldehyde (78 µL, 0.70 mmol, 1.4 equiv) was added dropwise and the reaction mixture was stirred for 0.5 h at 0 °C and then allowed to warm to room temperature and stirred for 1 h. Purification of the crude product by flash column chromatography (silica gel, *i*hexane/EtOAc = 9:1) afforded the title compound as a pale yellow oil (**9c**, 114 mg, 0.36 mmol, 72% yield).

^{1}H NMR (400 MHz, $CDCl_3$): δ (ppm) = 7.40–7.27 (m, 7H), 6.34 (d, *J* = 8.0 Hz, 1H), 2.94–2.81 (m, 1H).
^{13}C NMR (101 MHz, $CDCl_3$): δ (ppm) = 151.9 (ddd, *J* = 34.4, 10.7, 4.6 Hz), 149.4 (ddd, *J* = 33.6, 10.8, 4.5 Hz), 141.6–141.1 (m), 140.9, 139.2–138.5 (m), 128.7, 128.5–128.3 (m), 128.0, 125.6 (d, *J* = 1.4 Hz), 117.2 (dd, *J* = 19.9, 4.0 Hz), 116.3–115.8 (m), 73.3 (d, *J* = 1.8 Hz).
^{19}F NMR (377 MHz, $CDCl_3$): δ (ppm) = -133.0, -158.4.
MS (EI, 70 eV): m/z (%) = 317 (44), 315 (46), 238 (31), 219 (40), 79 (67), 42 (100).
HRMS (EI): for $C_{13}H_8BrF_3O$: calc. [M+]: 315.9711; found: 315.9697.

(5-Bromo-2,4-dimethoxyphenyl)(2,3,6-trifluorophenyl)methanol (9d)

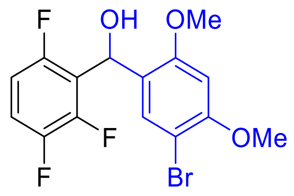

According to **TP 1**, to a mixture of 1,2,4-trifluorobenzene (**5c**, 52 µL, 0.50 mmol, 1.0 equiv) in toluene (1 mL) was added MBDA (0.40 mmol, 0.8 equiv) at 25 °C. After 15 min, the reaction mixture was cooled to 0 °C and 5-bromo-2,4-dimethoxybenzaldehyde (172 mg, 0.70 mmol, 1.4 equiv) dissolved in toluene (1 mL) was added dropwise and the reaction mixture was stirred for 0.5 h at 0 °C and then allowed to warm to room temperature and stirred for 1 h. Purification of the crude product by flash column chromatography (silica gel, *i*hexane/EtOAc = 4:1) afforded the title compound as a white solid (**9d**, 125 mg, 0.33 mmol, 66% yield).

^{1}H NMR (400 MHz, $CDCl_3$): δ (ppm) = 7.60 (q, *J* = 1.1 Hz, 1H), 7.09–7.00 (m, 1H), 6.78 (tdd, *J* = 9.4, 3.8, 2.2 Hz, 1H), 6.43 (s, 1H), 6.29 (s, 1H), 3.88 (s, 3H), 3.80 (s, 3H), 3.08 (s, 1H).
^{13}C NMR (101 MHz, $CDCl_3$): δ (ppm) = 157.7 (dd, *J* = 5.9, 2.8 Hz), 157.1, 156.8, 155.3 (dd, *J* = 5.9, 2.8 Hz), 150.3 (dd, *J* = 14.5, 8.6 Hz), 148.9 (dd, *J* = 13.6, 3.6 Hz), 147.8 (dd, *J* = 14.6, 8.6 Hz), 146.4 (dd, *J* = 13.5, 3.6 Hz), 131.4 (t, *J* = 2.3 Hz), 123.3 (d, *J* = 1.1 Hz), 120.8 (dd, *J* = 18.0, 12.8 Hz), 116.5 (ddd, *J* = 19.4, 10.4, 1.7 Hz), 111.4 (ddd, *J* = 25.1, 6.4, 4.1 Hz), 102.3, 96.8, 64.0 (td, *J* = 3.0, 1.9 Hz), 56.8, 56.2.
^{19}F NMR (376 MHz, $CDCl_3$): δ (ppm) = -118.8, -137.1, -142.3.
MS (EI, 70 eV): m/z (%) = 377 (43), 375 (46), 247 (29), 245 (31), 158 (32), 137 (28), 70 (13), 42 (100).
HRMS (EI): for $C_{15}H_{12}BrF_3O_3$: calc. [M^+]: 375.9922; found: 375.9920.

2,3-dibromo-5,6-difluoro-3'-methyl-1,1'-biphenyl (9e)

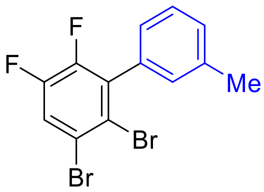

According to **TP 1**, to a mixture of 1,2-dibromo-4,5-difluorobenzene (**5d**, 136 mg, 0.50 mmol, 1.0 equiv) in toluene (1 mL) was added MBDA (0.55 mmol, 1.1 equiv) at 25 °C. After 5 min, the resulting diarylmagnesium was transmetalated with a $ZnCl_2$ solution (0.70 mL, 1.00 M in THF, 1.4 equiv) at 0 °C for 30 min. A dry and argon-flushed Schlenk-tube, equipped with a magnetic stirring bar and a septum was charged with $Pd(dba)_2$ (8 mg, 3 mol%), tfp (7 mg, 6 mol%) and 3-iodotoluene (54 µl, 0.42 mmol, 0.83 equiv).The freshly prepared arylzinc reagent was added and the reaction mixture was placed in an oil bath at 55 °C for 12 h. Purification of the crude product by flash column chromatography (silica gel, pentane) afforded the title compound as a colorless oil (**9e**, 104 mg, 0.29 mmol, 70% yield).

^{1}H NMR (400 MHz, $CDCl_3$): δ (ppm) = 7.55 (dd, *J* = 9.3, 7.5 Hz, 1H), 7.42–7.35 (m, 1H), 7.28 (d, *J* = 7.6 Hz, 1H), 7.11–7.06 (m, 2H), 2.43 (s, 3H).

^{13}C NMR (101 MHz, $CDCl_3$): δ (ppm) = 148.7 (dd, *J* = 218.1, 13.8 Hz), 147.2 (dd, *J* = 214.3, 13.8 Hz),138.3, 134.8 (d, *J* = 15.9 Hz), 134.3 (d, *J* = 2.2 Hz), 130.2 (d, *J* = 1.3 Hz), 129.8, 128.5, 126.6 (d, *J* = 1.3 Hz), 121.7 (d, *J* = 4.0 Hz), 120.9 (d, *J* = 20.6 Hz), 119.5 (dd, *J* = 7.8, 4.7 Hz), 21.6.

^{19}F NMR (377 MHz, $CDCl_3$): δ (ppm) = -133.4, -135.5.

MS (EI, 70 eV): m/z (%) = 363 (32), 361 (76), 359 (39), 201 (73), 43 (41), 42 (100).

HRMS (EI): for $C_{13}H_8Br_2F_2$: calc. [M+]: 359.8961; found: 359.8951.

ethyl 2',5'-dibromo-3',6'-difluoro-[1,1'-biphenyl]-4-carboxylate (9f)

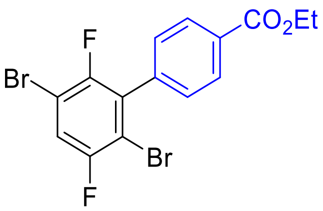

According to **TP 1**, to a mixture of 1,4-dibromo-2,5-difluorobenzene (**5e**, 136 mg, 0.50 mmol, 1.0 equiv) in toluene (1 mL) was added MBDA (0.55 mmol, 1.1 equiv) at 25 °C. After 5 min, the resulting diarylmagnesium was transmetalated with a $ZnCl_2$ solution (0.70 mL, 1.00 M in THF, 1.4 equiv) at 0 °C for 30 min. A dry and argon-flushed Schlenk-tube, equipped with a magnetic stirring bar and a septum was charged with $Pd(dba)_2$ (8 mg, 3 mol%), tfp (7 mg, 6 mol%) and 4-iodo ethylbenzoate (72 µl, 0.42 mmol, 0.83 equiv).The freshly prepared arylzinc reagent was added and the reaction mixture was placed in an oil bath at 55 °C for 12 h. Purification of the crude product by flash column chromatography (silica gel, *i*hexane/EtOAc = 98:2) afforded the title compound as a yellow solid (**9f**, 137 mg, 0.33 mmol, 80% yield).

^{1}H NMR (400 MHz, $CDCl_3$): δ (ppm) = 8.20–8.14 (m, 2H), 7.46–7.36 (m, 3H), 4.42 (q, *J* = 7.1 Hz, 2H), 1.42 (t, *J* = 7.1 Hz, 3H).

^{13}C NMR (101 MHz, $CDCl_3$): δ (ppm) = 166.2, 156.7 (d, *J* = 3.7 Hz), 154.2 (dd, *J* = 17.3, 3.5 Hz), 151.7 (d, *J* = 3.3 Hz), 137.5 (d, *J* = 1.9 Hz), 132.1 (d, *J* = 21.2 Hz), 131.1, 130.0 (d, *J* = 1.4 Hz), 129.8, 119.9 (d, *J* = 27.7 Hz), 110.5 (dd, *J* = 22.7, 2.2 Hz), 108.7 (dd, *J* = 25.0, 9.6 Hz), 61.4, 14.5.

^{19}F NMR (377 MHz, $CDCl_3$): δ (ppm) = -106.9, -108.2.

MS (EI, 70 eV): m/z (%) = 419 (39), 391 (54), 376 (51), 374 (100), 372 (54), 268 (48), 187 (50).

HRMS (EI): for $C_{15}H_{10}Br_2F_2O_2$: calc. [M^+]: 417.9016; found: 417.9009.

2',4'-dibromo-3'-chloro-6'-fluoro-1,2,3,4-tetrahydro-1,1'-biphenyl (9g)

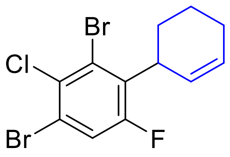

According to **TP 1**, to a mixture of 1,3-dibromo-2-chloro-5-fluorobenzene (**5f**, 144 mg, 0.50 mmol, 1.0 equiv) in toluene (1 mL) was added MBDA (0.55 mmol, 1.1 equiv) at 25 °C. After 0.5 h, the reaction mixture was cooled to -25 °C and CuCN·2LiCl (0.1 mL, 20 mol%, 1M in THF) and 3-bromocyclohexen (70 µL, 0.6 mmol, 1.2 equiv) were added and the mixture was stirred for 0.5 h at -25 °C. Purification of the crude product by flash column chromatography (silica gel,pentane) afforded the title compound as a white solid (**9g**, 152 mg, 0.41 mmol, 82% yield).

^{1}H NMR (400 MHz, CDCl$_3$): δ (ppm) = 7.33 (d, *J* = 9.9 Hz, 1H), 5.83–5.76 (m, 1H), 5.59 (dt, *J* = 10.1, 2.5 Hz, 1H), 4.05 (tdd, *J* = 9.2, 7.7, 4.8, 2.4 Hz, 1H), 2.20–2.02 (m, 2H), 1.99–1.86 (m, 2H), 1.79–1.63 (m, 2H).

^{13}C NMR (101 MHz, CDCl$_3$): δ (ppm) = 160.7, 158.2, 134.7 (d, *J* = 14.8 Hz), 131.3, 128.1 (d, *J* = 2.0 Hz), 127.1, 126.2, 120.9–120.4 (m), 27.7, 24.6, 22.8.

^{19}F NMR (377 MHz, CDCl$_3$): δ (ppm) = -109.9.

MS (EI, 70 eV): m/z (%) = 369 (38), 367 (54), 313 (46), 182 (36), 180 (100), 67 (31), 43 (36).

HRMS (EI): for $C_{12}H_{10}Br_2ClF$: calc. [M+]: 365.8822; found: 365.8824.

ethyl 2-(3,4-dibromo-6-chloro-2-fluorobenzyl)acrylate (9h)

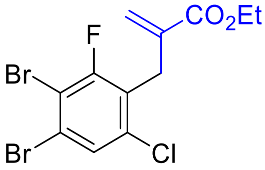

According to **TP 1**, to a mixture of 1,2-dibromo-5-chloro-3-fluorobenzene (**5g**, 144 mg, 0.50 mmol, 1.0 equiv) in toluene (1 mL) was added MBDA (0.55 mmol, 1.1 equiv) at 25 °C. After 0.5 h, the reaction mixture was cooled to -25 °C and CuCN·2LiCl (0.1 mL, 20 mol%, 1M in THF) and ethyl 2-(bromomethyl)acrylate (97 µL, 0.6 mmol, 1.2 equiv) were added and the mixture was stirred for 0.5 h at -25 °C. Purification of the crude product by flash column chromatography (silica gel,pentane) afforded the title compound as a colorless oil (**9h**, 104 mg, 0.26 mmol, 52% yield).

^{1}H NMR (400 MHz, CDCl$_3$): δ (ppm) = 7.53 (d, *J* = 1.9 Hz, 1H), 6.24 (d, *J* = 1.6 Hz, 1H), 5.17 (d, *J* = 2.0 Hz, 1H), 4.24 (q, *J* = 7.1 Hz, 2H), 3.78 (q, *J* = 1.9 Hz, 2H), 1.31 (t, *J* = 7.1 Hz, 3H).
^{13}C NMR (101 MHz, CDCl$_3$): δ (ppm) = 166.3, 158.5 (d, *J* = 251.9 Hz), 136.1, 135.4 (d, *J* = 6.4 Hz), 129.3 (d, *J* = 3.9 Hz), 125.6, 125.1 (d, *J* = 20.3 Hz), 124.1 (d, *J* = 1.5 Hz), 111.9 (d, *J* = 24.6 Hz), 61.2, 29.1 (d, *J* = 2.6 Hz), 14.3.
^{19}F NMR (376 MHz, CDCl$_3$): δ (ppm) = -96.9.
MS (EI, 70 eV): m/z (%) = 364 (23), 219 (15), 167 (100).
HRMS (EI): for $C_{12}H_{10}Br_2ClFO_2$: calc. [M+]: 397.8720; found: 397.8726.

1-bromo-2-fluoro-3,4-diiodobenzene (9i)

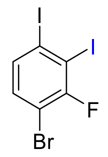

According to **TP 1**, to a mixture of 1-bromo-2-fluoro-4-iodobenzene (**5h**, 150 mg, 0.50 mmol, 1.0 equiv) in toluene (1 mL) was added MBDA (0.40 mmol, 0.8 equiv) at 40 °C. After 0.5 h, the reaction mixture was cooled to 0 °C and iodine (152 mg, 0.60 mmol, 1.2 equiv) dissolved in THF (1 mL) was added dropwise and the reaction mixture was stirred for 1 h. Purification of the crude product by flash column chromatography (silica gel, pentane) afforded the title compound as a white solid (**9i**, 129 mg, 0.30 mmol, 60% yield).

^{1}H NMR (400 MHz, $CDCl_3$): δ (ppm) = 7.55 (dd, *J* = 8.4, 1.3 Hz, 1H), 7.32–7.26 (m, 1H).
^{13}C NMR (101 MHz, $CDCl_3$): δ (ppm) = 158.3 (d, *J* = 247.7 Hz), 135.5 (d, *J* = 4.2 Hz), 134.7, 108.4, 107.6, 97.7 (d, *J* = 27.9 Hz).
^{19}F NMR (377 MHz, $CDCl_3$): δ (ppm) = -70.4.
MS (EI, 70 eV): m/z (%) = 427 (91), 425 (100), 126 (52).
HRMS (EI): for $C_6H_2BrFI_2$: calc. [M^+]: 425.7413; found: 425.7397.

2-((3-bromo-2-fluoro-6-iodophenyl)thio)pyridine (9j)

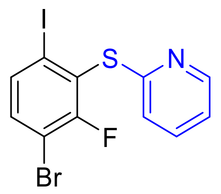

According to **TP 1**, to a mixture of 1-bromo-2-fluoro-4-iodobenzene (**5h**, 150 mg, 0.50 mmol, 1.0 equiv) in toluene (1 mL) was added MBDA (0.40 mmol, 0.8 equiv) at 40 °C. After 0.5 h, the reaction mixture was cooled to 0 °C and 2,2′-dipyridyl disulfide (132 mg, 0.60 mmol, 1.2 equiv) dissolved in toluene (1 mL) was added dropwise and the reaction mixture was stirred for 1 h. Purification of the crude product by flash column chromatography (silica gel, *i*hexane/EtOAc = 94:6) afforded the title compound as a white solid (**9j**, 145 mg, 0.35 mmol, 70% yield).

^{1}H NMR (400 MHz, $CDCl_3$): δ (ppm) = 8.38 (ddd, *J* = 4.9, 1.9, 0.9 Hz, 1H), 7.68 (dd, *J* = 8.5, 1.4 Hz, 1H), 7.53 (ddd, *J* = 8.0, 7.4, 1.9 Hz, 1H), 7.35 (dd, *J* = 8.5, 6.6 Hz, 1H), 7.05 (ddd, *J* = 7.5, 4.9, 1.0 Hz, 1H), 6.99 (dt, *J* = 8.0, 1.0 Hz, 1H).

^{13}C NMR (101 MHz, $CDCl_3$): δ (ppm) = 159.4 (d, *J* = 251.8 Hz), 157.5, 150.1, 137.0, 136.2 (d, *J* = 4.5 Hz), 136.0 (d, *J* = 1.4 Hz), 126.1 (d, *J* = 20.6 Hz), 121.2, 120.7, 110.4 (d, *J* = 24.4 Hz), 108.3 (d, *J* = 2.3 Hz).

^{19}F NMR (376 MHz, $CDCl_3$): δ (ppm) = -86.9.

MS (EI, 70 eV): m/z (%) = 283 (33), 281 (33), 203 (22), 61 (15) 50 (14), 43 (100).

HRMS (EI): for $C_{11}H_6BrFINS$: calc. [M^+]: 408.8433; found: 408.8432.

1,2-difluoro-3-iodo-4-methoxybenzene (9k)

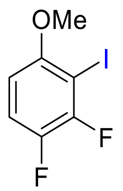

According to **TP 1**, to a mixture of 1,2-difluoro-4-methoxybenzene (**5i**, 58 µL, 0.50 mmol, 1.0 equiv) in toluene (1 mL) was added MBDA (0.55 mmol, 1.1 equiv) at 40 °C. After 15 min, the reaction mixture was cooled to 0 °C and iodine (152 mg, 0.60 mmol, 1.2 equiv) dissolved in THF (1 mL) was added dropwise and the reaction mixture was stirred for 1 h. Purification of the crude product by flash column chromatography (silica gel, *i*hexane/EtOAc = 94:6) afforded the title compound as a yellow solid (**9k**, 128 mg, 0.47 mmol, 94% yield).

^{1}H NMR (400 MHz, $CDCl_3$): δ (ppm) = 7.13 (q, *J* = 9.2 Hz, 1H), 6.54 (ddd, *J* = 9.2, 3.7, 2.1 Hz, 1H), 3.86 (s, 3H).
^{13}C NMR (101 MHz, $CDCl_3$): δ (ppm) = 155.4 (dd, *J* = 4.0, 2.3 Hz), 150.9 (dd, *J* = 244.7, 14.9 Hz), 144.9 (dd, *J* = 244.5, 15.2 Hz), 116.8 (dd, *J* = 18.6, 1.9 Hz), 105.4 (dd, *J* = 6.0, 3.3 Hz), 75.6 (d, *J* = 23.6 Hz), 57.1.
^{19}F NMR (376 MHz, $CDCl_3$): δ (ppm) = -113.2, -144.5.
MS (EI, 70 eV): m/z (%) = 269 (100), 254 (44), 226 (24), 42 (75).
HRMS (EI): for $C_7H_5F_2IO$: calc. [M+]: 269.9353; found: 269.9346.

3-(2',3'-difluoro-6'-methoxy-[1,1'-biphenyl]-4-yl)-N,N-dimethyl-3-(pyridin-2-yl)propan-1-amine (9l)

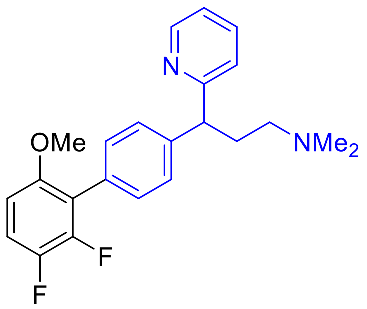

1,2-difluoro-4-methoxybenzene (**5i**, 58 µL, 0.50 mmol, 1.0 equiv) in toluene (1 mL) was added MBDA (0.55 mmol, 1.1 equiv) at 40 °C. After 15 min, the resulting diarylmagnesium was transmetalated with a $ZnCl_2$ solution (0.70 mL, 1.00 M in THF, 1.4 equiv) at 0 °C for 30 min. A dry and argon-flushed Schlenk-tube, equipped with a magnetic stirring bar and a septum was charged with $PdCl_2$(dppf) (18 mg, 5 mol%) and brompheniramine (132 mg, 0.42 mmol, 0.83 equiv).The freshly prepared arylzinc reagent was added and the reaction mixture was placed in an oil bath at 55 °C for 16 h. Purification of the crude product by flash column chromatography (silica gel, DCM/MeOH = 96:4) afforded the title compound as a brown oil (**9l**, 135 mg, 0.35 mmol, 84% yield).

^{1}H NMR (400 MHz, $CDCl_3$): δ (ppm) = 8.59–8.53 (m, 1H), 7.57 (tdd, J = 7.7, 1.8, 1.0 Hz, 1H), 7.41–7.29 (m, 4H), 7.20 (dd, J = 7.9, 1.2 Hz, 1H), 7.17–7.00 (m, 2H), 6.61 (ddd, J = 9.3, 3.8, 1.9 Hz, 1H), 4.21 (t, J = 7.6 Hz, 1H), 3.69 (d, J = 1.1 Hz, 3H), 2.81 (dtd, J = 25.4, 9.6, 8.3, 5.1 Hz, 3H), 2.64 (s, 6H), 2.59–2.45 (m, 1H).
^{13}C NMR (101 MHz, $CDCl_3$): δ (ppm) = 161.4, 153.1 (dd, *J* = 4.9, 2.2 Hz), 149.4, 149.3, 149.3, 146.9 (d, *J* = 14.0 Hz), 144.5 (d, *J* = 14.2 Hz), 141.9, 137.0, 131.0 (d, *J* = 1.8 Hz), 129.3 (d, *J* = 2.2 Hz), 127.6, 123.8, 122.0, 120.1 (d, *J* = 14.0 Hz), 115.1 (d, *J* = 1.9 Hz), 105.9 (dd, *J* = 6.5, 3.6 Hz), 56.8, 56.3, 50.4, 43.3, 29.7.
^{19}F NMR (376 MHz, $CDCl_3$): δ (ppm) = -139.0, -147.3.
MS (EI, 70 eV): m/z (%) = 336 (22), 335 (100), 320 (57), 207 (11), 159 (15).
HRMS (EI): for $C_{23}H_{24}F_2N_2O$: calc. [M+]: 382.1857; found: 382.1851.

1,5-dichloro-2-iodo-3-methoxybenzene (9m)

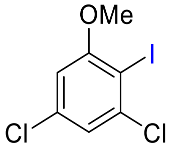

According to **TP 1**, to a mixture of 3,5-dichloroanisole (**5j**, 88 mg, 0.50 mmol, 1.0 equiv) in toluene (1 mL) was added MBDA (0.55 mmol, 1.1 equiv) at 70 °C. After 1 h, the reaction mixture was cooled to 0 °C and iodine (152 mg, 0.60 mmol, 1.2 equiv) dissolved in THF (1 mL) was added dropwise and the reaction mixture was stirred for 1 h. Purification of the crude product by flash column chromatography (silica gel, *i*hexane/EtOAc = 99:1) afforded the title compound as a white solid (**9m**, 116 mg, 0.38 mmol, 76% yield).

^{1}H NMR (400 MHz, $CDCl_3$): δ (ppm) = 7.12 (d, *J* = 2.1 Hz, 1H), 6.67 (d, *J* = 2.1 Hz, 1H), 3.88 (s, 3H).

^{13}C NMR (101 MHz, $CDCl_3$): δ (ppm) = 160.3, 140.4, 135.6, 121.7, 109.5, 89.2, 57.1.

MS (EI, 70 eV): m/z (%) = 303 (18), 301 (28), 88 (18), 60 (10), 46 (100).

HRMS (EI): for $C_7H_5Cl_2IO$: calc. [M+]: 301.8762; found: 301.8768.

(1r,5R,7S)-2-(2,4-dichloro-6-methoxyphenyl)adamantan-2-ol (9n)

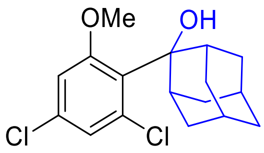

According to **TP 1**, to a mixture of 3,5-dichloroanisole (**5j**, 88 mg, 0.50 mmol, 1.0 equiv) in toluene (1 mL) was added MBDA (0.55 mmol, 1.1 equiv) at 70 °C. After 1 h, the reaction mixture was cooled to 0 °C and adamantan-2-one (105 mg, 0.70 mmol, 1.4 equiv) dissolved in toluene (1 mL) was added dropwise and the reaction mixture was stirred for 1 h. Purification of the crude product by flash column chromatography (silica gel, *i*hexane/EtOAc = 98:2) afforded the title compound as a white solid (**9n**, 103 mg, 0.32 mmol, 64% yield).

^{1}H NMR (400 MHz, $CDCl_3$): δ (ppm) = 7.01 (d, J = 2.2 Hz, 1H), 6.80 (d, J = 2.2 Hz, 1H), 3.81 (s, 3H), 3.31 (s, 2H), 2.96 (q, J = 3.1 Hz, 1H), 2.58–2.52 (m, 1H), 2.42 (dt, J = 12.6, 3.1 Hz, 2H), 2.13–1.91 (m, 5H), 1.80–1.69 (m, 2H), 1.64–1.53 (m, 2H).
^{13}C NMR (101 MHz, $CDCl_3$): δ (ppm) = 160.7, 135.1, 133.3, 130.5, 125.5, 112.1, 80.7, 56.3, 47.4, 39.7, 37.9, 37.2, 36.7, 36.6, 36.5, 35.5, 34.0, 33.6, 27.9, 27.0, 26.8.
MS (EI, 70 eV): m/z (%) = 202 (12), 70 (12), 61 (15), 46 (13), 45 (100).
HRMS (EI): for $C_{17}H_{20}Cl_2O_2$: calc. [M+]: 326.0840; found: 326.0832.

5-(4-((tert-butyldimethylsilyl)oxy)-2,6-difluorophenyl)-6-methoxy-2,3-dihydro-1H-inden-1-one (9o)

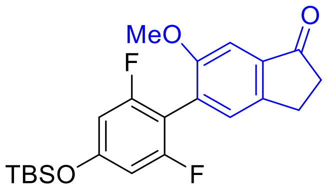

According to **TP 1**, to a mixture of tert-butyl(3,5-difluorophenoxy)dimethylsilane (**5k**, 122 mg, 0.50 mmol, 1.0 equiv) in toluene (1 mL) was added MBDA (0.55 mmol, 1.1 equiv) at 25 °C. After 1 h, the resulting diarylmagnesium was transmetalated with a $ZnCl_2$ solution (0.70 mL, 1.00 M in THF, 1.4 equiv) at 0 °C for 30 min. A dry and argon-flushed Schlenk-tube, equipped with a magnetic stirring bar and a septum was charged with $Pd(dba)_2$ (8 mg, 3 mol%), tfp (7 mg, 6 mol%) and 5-iodo-6-methoxy-2,3-dihydro-1H-inden-1-one (120 mg, 0.42 mmol, 0.83 equiv).The freshly prepared arylzinc reagent was added and the reaction mixture was placed in an oil bath at 55 °C for 12 h. Purification of the crude product by flash column chromatography (silica gel, *i*hexane/EtOAc = 9:1) afforded the title compound as a yellow solid (**9o**, 104 mg, 0.26 mmol, 63% yield).

^{1}H NMR (400 MHz, $CDCl_3$): δ (ppm) = 7.37 (s, 1H), 7.30 (s, 1H), 6.50–6.44 (m, 2H), 3.83 (s, 3H), 3.15–3.09 (m, 2H), 2.77–2.71 (m, 2H), 1.00 (s, 9H), 0.26 (s, 6H).
^{13}C NMR (101 MHz, $CDCl_3$): δ (ppm) = 207.0, 160.6 (dd, *J* = 239.6, 11.0 Hz), 157.5, 157.1 147.3, 138.4, 130.2, 126.4, 107.8, 104.4, 103.8 (d, *J* = 27.9 Hz), 56.2, 37.1, 34.3, 29.9, 25.7, 25.2, 22.5, 18.3, 14.2, -4.3.
^{19}F NMR (376 MHz, $CDCl_3$): δ (ppm) = -112.1.
MS (EI, 70 eV): m/z (%) = 404 (37), 348 (48), 347 (100), 42 (27).
HRMS (EI): for $C_{22}H_{26}F_2O_3Si$: calc. [M-C_4H_9]: 347.0931; found: 347.0939.

3-((3-(1,3-dioxolan-2-yl)-2,5,6-trifluorophenyl)ethynyl)pyridine (9p)

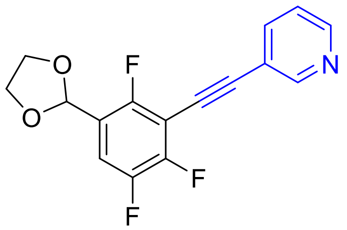

According to **TP 1**, to a mixture of 2-(2,4,5-trifluorophenyl)-1,3-dioxolane (**5l**, 102 mg, 0.50 mmol, 1.0 equiv) in toluene (1 mL) was added MBDA (0.55 mmol, 1.1 equiv) at 25 °C. After 15 min, the resulting diarylmagnesium was transmetalated with a $ZnCl_2$ solution (0.70 mL, 1.00 M in THF, 1.4 equiv) at 0 °C for 0.5 h. Iodine (152 mg, 0.60 mmol, 1.2 equiv) dissolved in THF (1 mL) was added dropwise and the reaction mixture was stirred for 1 h. . A dry and argon-flushed Schlenk-tube, equipped with a magnetic stirring bar and a septum was charged with $Pd(dba)_2$ (8 mg, 3 mol%), tfp (7 mg, 6 mol%), CuI (4 mg, 4 mol%)and Et_3N (3 mL). The freshly prepared arylzinc reagent followed by 3-ethynyl pyridine (73 mg, 0.7 mmol, 1.4 equiv) were added and the reaction mixture was placed in an oil bath at 55 °C for 2 h. Purification of the crude product by flash column chromatography (silica gel, *i*hexane/EtOAc = 2:1) afforded the title compound as a yellow solid (**9p**, 108 mg, 0.35 mmol, 70% yield).

^{1}H NMR (400 MHz, $CDCl_3$): δ (ppm) = 8.80 (dd, *J* = 2.1, 0.9 Hz, 1H), 8.60 (dd, *J* = 4.9, 1.7 Hz, 1H), 7.85 (dt, *J* = 7.9, 1.9 Hz, 1H), 7.41–7.28 (m, 2H), 6.03 (d, *J* = 1.1 Hz, 1H), 4.17–4.01 (m, 4H).
^{13}C NMR (101 MHz, $CDCl_3$): δ (ppm) = 156.5 (dt, *J* = 254.4, 2.9 Hz), 152.5, 152.2 (dd, *J* = 15.1, 5.3 Hz), 149.7, 146.8 (ddd, *J* = 246.3, 12.1, 3.7 Hz), 138.9, 123.2, 122.0 (dt, *J* = 14.4, 4.9 Hz), 119.4, 115.8 (ddd, *J* = 20.6, 5.3, 1.6 Hz), 103.8 (dt, *J* = 14.3, 1.4 Hz), 97.7 (d, *J* = 3.0 Hz), 97.0 (t, *J* = 3.5 Hz), 78.4 (d, *J* = 3.7 Hz), 65.6.
^{19}F NMR (376 MHz, $CDCl_3$): δ (ppm) = -115.1, -118.6, -128.8.
MS (EI, 70 eV): m/z (%) = 302 (27), 301 (93), 284 (100), 129 (39), 55 (45), 42 (90).
HRMS (EI): for $C_{16}H_{10}F_3NO_2$: calc. [M+]: 305.0664; found: 305.0658.

2,4,5-trifluoro-3-(pyridin-3-ylethynyl)benzaldehyde (9q)

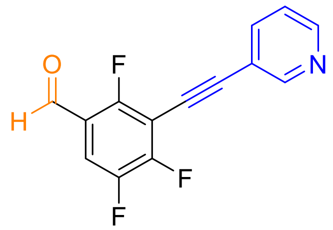

3-((3-(1,3-dioxolan-2-yl)-2,5,6-trifluorophenyl)ethynyl)pyridine (**9p**, 31 mg, 0.10 mmol, 1.0 equiv) was dissolved in THF (1 mL). The reaction mixture was cooled to 0 C° and conc. HCl (3 drops) was added. The reaction mixture was allowed to warm to room temperature and stirred for 0.5 h. The reaction was quenched by addition of sat. $NaHCO_3$ solution (5 mL) and extracted with EtOAc (3 x 10 mL). Purification of the crude product by flash column chromatography (silica gel, ihexane/EtOAc = 2:1) afforded the title compound as a white solid (**9q**, 25 mg, 0.096 mmol, 96% yield).

^{1}H NMR (400 MHz, CDCl$_3$): δ (ppm) = 10.29 (d, *J* = 2.9 Hz, 1H), 8.83 (d, *J* = 2.1 Hz, 1H), 8.64 (dd, *J* = 4.9, 1.7 Hz, 1H), 7.89 (dt, *J* = 7.9, 2.0 Hz, 1H), 7.69 (td, *J* = 9.0, 6.2 Hz, 1H), 7.40–7.32 (m, 1H).

^{13}C NMR (101 MHz, CDCl$_3$): δ (ppm) = 184.3 (dt, *J* = 6.7, 1.5 Hz), 160.8 (d, *J* = 262.9 Hz), 156.4–153.3 (m), 152.6, 152.5, 150.1, 149.2–148.7 (m), 146.6–146.2 (m), 139.0, 123.3, 121.3–120.3 (m), 118.9, 115.5 (dt, *J* = 19.8, 2.9 Hz), 105.6–104.8 (m), 98.5 (t, *J* = 3.5 Hz), 97.8, 65.7.

^{19}F NMR (376 MHz, CDCl$_3$): δ (ppm) = -118.7, -120.6, -138.6.

MS (EI, 70 eV): m/z (%) = 262 (13), 261 (100).

HRMS (EI): for $C_{14}H_6F_3NO$: calc. [M+]: 261.0401; found: 261.0408.

tert-butyl 3,4,6-trifluoro-2-iodobenzoate (9r)

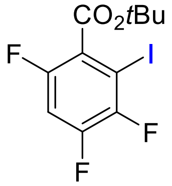

According to **TP 1**, to a mixture of tert-butyl 2,4,5-trifluorobenzoate (**5m**, 116 mg, 0.50 mmol, 1.0 equiv) in toluene (1 mL) was added MBDA (0.40 mmol, 0.8 equiv) at 25 °C. After 15 min, the reaction mixture was cooled to 0 °C and iodine (152 mg, 0.60 mmol, 1.2 equiv) dissolved in THF (1 mL) was added dropwise and the reaction mixture was stirred for 1 h. Purification of the crude product by flash column chromatography (silica gel, *i*hexane/EtOAc = 99:1) afforded the title compound as a brown solid (**9r**, 112 mg, 0.31 mmol, 62% yield).

^{1}H NMR (400 MHz, $CDCl_3$): δ (ppm) = 7.02 (ddd, *J* = 9.5, 8.4, 6.1 Hz, 1H), 1.61 (s, 9H).
^{13}C NMR (101 MHz, $CDCl_3$): δ (ppm) = 162.6, 154.6 (ddd, J = 252.6, 10.5, 3.6 Hz), 149.7 (ddd, J = 228.1, 12.2, 4.0 Hz), 147.5 (ddd, *J* = 225.1, 13.8, 4.7 Hz), 126.1 (dd, *J* = 21.6, 4.4 Hz), 106.5 (dd, *J* = 27.6, 21.5 Hz), 84.7, 82.2 (dd, *J* = 26.1, 5.2 Hz), 28.2.
^{19}F NMR (376 MHz, $CDCl_3$): δ (ppm) = -117.8, -129.3, -141.0.
MS (EI, 70 eV): m/z (%) = 306 (100), 305 (66), 260 (44), 233 (54), 73 (59), 42 (87).
HRMS (EI): for $C_{11}H_{10}F_3IO_2$: calc. [M+]: 357.9678; found: 357.9676.

tert-butyl 3,4-difluoro-2-(phenylethynyl)benzoate (9s)

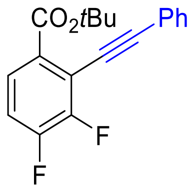

f

According to **TP 1**, to a mixture of tert-butyl 3,4-difluorobenzoate (**5n**, 107 mg, 0.50 mmol, 1.0 equiv) in toluene (1 mL) was added MBDA (0.55 mmol, 1.1 equiv) at 25 °C. After 20 min, the resulting diarylmagnesium was transmetalated with a ZnCl2 solution (0.70 mL, 1.00 M in THF, 1.4 equiv) at 0 °C for 30 min. Iodine (152 mg, 0.60 mmol, 1.2 equiv) dissolved in THF (1 mL) was added dropwise and the reaction mixture was stirred for 1 h. . A dry and argon-flushed Schlenk-tube, equipped with a magnetic stirring bar and a septum was charged with $Pd(dba)_2$ (8 mg, 3 mol%), tfp (7 mg, 6 mol%), CuI (4 mg, 4 mol%)and Et_3N (3 mL). The freshly prepared arylzinc reagent followed by phenylacetylene (77 µL, 0.7 mmol, 1.4 equiv) were added and the reaction mixture was placed in an oil bath at 55 °C for 2 h. Purification of the crude product by flash column chromatography (silica gel, *i*hexane/EtOAc = 9:1) afforded the title compound as a yellow oil (**9s**, 117 mg, 0.37 mmol, 73% yield).

^{1}H NMR (400 MHz, $CDCl_3$): δ (ppm) = 7.68 (ddd, *J* = 8.8, 5.1, 1.9 Hz, 1H), 7.62–7.56 (m, 2H), 7.38 (tt, *J* = 3.8, 2.3 Hz, 3H), 7.16 (td, *J* = 9.0, 7.4 Hz, 1H), 1.60 (s, 9H).
^{13}C NMR (101 MHz, $CDCl_3$): δ (ppm) = 164.2 (d, *J* = 2.6 Hz), 152.5 (dd, *J* = 242.1, 19.8 Hz), 151.5 (dd, *J* = 239.8, 9.8 Hz) 131.9, 130.9 (d, *J* = 3.5 Hz), 129.2, 128.6, 126.7–126.4 (m), 122.9, 116.4 (d, *J* = 17.6 Hz), 115.1–114.6 (m), 101.1 (d, *J* = 5.2 Hz), 82.5, 80.2 (d, *J* = 3.8 Hz), 28.3.
^{19}F NMR (376 MHz, $CDCl_3$): δ (ppm) = -132.0, -132.3.
MS (EI, 70 eV): m/z (%) = 259 (13), 258 (100), 240 (16), 200 (14).
HRMS (EI): for $C_{19}H_{16}F_2O_2$: calc. [M^+]: 314.1118; found: 314.1121.

tert-butyl 6-fluoro-1',2',3',4'-tetrahydro-[1,1'-biphenyl]-2-carboxylate (9t)

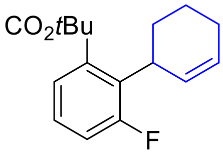

According to **TP 2**, to a mixture of 3 tert-butyl 3-fluorobenzoate (**5o**, 392 mg, 2.00 mmol, 1.0 equiv) in toluene (4 mL) and THF (0.49 mL, 3.0 equiv) was added MBDA (2.20 mmol, 1.1 equiv) at 25 °C. After 15 min, the reaction mixture was cooled to -25 °C and CuCN·2LiCl (0.4 mL, 20 mol%, 1M in THF) and 3-bromocyclohexen (0.32 mL, 0.6 mmol, 1.2 equiv) were added and the mixture was stirred for 0.5 h at -25 °C. Purification of the crude product by flash column chromatography (silica gel, *i*hexane/EtOAc = 99:1) afforded the title compound as a colorless oil (**9t**, 414 mg, 1.50 mmol, 75% yield).

^{1}H NMR (400 MHz, $CDCl_3$): δ (ppm) = 7.33 (dd, *J* = 7.7, 1.4 Hz, 1H), 7.19 (td, *J* = 7.9, 5.1 Hz, 1H), 7.07 (ddd, *J* = 11.2, 8.1, 1.4 Hz, 1H), 5.81–5.73 (m, 1H), 5.64 (ddt, *J* = 10.1, 3.3, 1.7 Hz, 1H), 3.96 (ddq, *J* = 9.9, 5.1, 2.5 Hz, 1H), 2.23–2.00 (m, 3H), 1.96–1.81 (m, 2H), 1.76–1.64 (m, 1H), 1.58 (s, 9H).
^{13}C NMR (101 MHz, $CDCl_3$): δ (ppm) = 167.7 (d, *J* = 3.7 Hz), 162.2 (d, *J* = 249.0 Hz), 136.0 (d, *J* = 5.1 Hz), 132.4 (d, *J* = 13.1 Hz), 129.8 (d, *J* = 1.3 Hz), 127.4 (d, *J* = 9.0 Hz), 126.6 (d, *J* = 2.7 Hz), 124.7 (d, *J* = 3.3 Hz), 118.4 (d, *J* = 23.0 Hz), 82.3, 36.9, 29.1 (d, *J* = 2.0 Hz), 28.3, 24.8, 23.2.
^{19}F NMR (376 MHz, $CDCl_3$): δ (ppm) = -112.9.
MS (EI, 70 eV): m/z (%) = 202 (100), 201 (62), 184 (57), 173 (19), 146 (27).
HRMS (EI): for $C_{17}H_{21}FO_2$: calc. [M+]: 276.1526; found: 276.1534.

6-fluoro-N,N-diisopropyl-1',2',3',4'-tetrahydro-[1,1'-biphenyl]-2-carboxamide (9u)

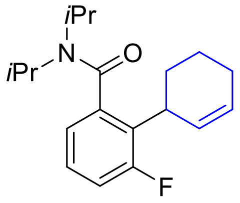

According to **TP 1**, to a mixture of 3-fluoro-*N*,*N*-diisopropylbenzamide (**5p**, 112 mg, 0.50 mmol, 1.0 equiv) in toluene (1 mL) was added MBDA (0.55 mmol, 1.1 equiv) at 25 °C. After 10 min, the reaction mixture was cooled to -25 °C and CuCN·2LiCl (0.1 mL, 20 mol%, 1M in THF) and 3-bromocyclohexen (70 µL, 0.6 mmol, 1.2 equiv) were added and the mixture was stirred for 0.5 h at -25 °C. Purification of the crude product by flash column chromatography (silica gel, *i*hexane/EtOAc = 92:8) afforded the title compound as a white solid (**9u**, 120 mg, 0.40 mmol, 80% yield).

^{1}H NMR (400 MHz, CDCl$_3$): δ (ppm) = 7.17 (tt, *J* = 7.9, 5.2 Hz, 1H), 6.96 (dddd, *J* = 11.1, 8.3, 2.6, 1.3 Hz, 1H), 6.86 (ddd, *J* = 7.6, 4.2, 1.3 Hz, 1H), 5.77 (dddd, *J* = 14.7, 10.0, 4.5, 2.6 Hz, 1H), 5.62 (dddd, *J* = 11.9, 10.1, 3.9, 1.8 Hz, 1H), 3.77–3.34 (m, 3H), 2.22–1.99 (m, 3H), 1.94–1.74 (m, 2H), 1.67-1.51 (m, 7H), 1.18–1.05 (m, 6H).

^{13}C NMR (101 MHz, CDCl$_3$): δ (ppm) = 169.2 (dd, *J* = 10.0, 3.0 Hz), 162.2 (d, *J* = 250.1 Hz), 140.6 (dd, *J* = 13.9, 4.9 Hz), 129.8 (d, *J* = 13.6 Hz), 129.5–129.2 (m), 127.9 (dd, *J* = 33.7, 9.0 Hz), 127.3 (dd, *J* = 4.5, 2.1 Hz), 121.0 (d, *J* = 3.5 Hz), 120.4 (d, *J* = 3.5 Hz), 116.0 (dd, *J* = 22.6, 17.4 Hz), 50.9 (d, *J* = 9.8 Hz), 45.9 (d, *J* = 4.5 Hz), 38.1, 37.0, 29.1 (dd, *J* = 24.2, 2.3 Hz), 24.8 (d, *J* = 8.8 Hz), 23.1 (d, *J* = 5.4 Hz), 20.9 – 20.3 (m).

^{19}F NMR (376 MHz, CDCl$_3$): δ (ppm) = -112.2.

MS (EI, 70 eV): m/z (%) = 202 (100), 201 (75), 185 (55), 165 (35), 146 (34), 133 (36).

HRMS (EI): for $C_{19}H_{26}FNO$: calc. [M+]: 303.1998; found: 303.1994.

2-((5-bromo-2-methoxyphenyl)(hydroxy)methyl)-3-fluoro-N,N-diisopropylbenzamide (9v)

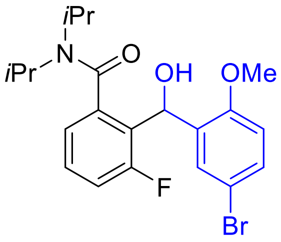

According to **TP 1**, to a mixture of 3-fluoro-*N*,*N*-diisopropylbenzamide (**5p**, 112 mg, 0.50 mmol, 1.0 equiv) in toluene (1 mL) was added MBDA (0.55 mmol, 1.1 equiv) at 25 °C. After 10 min, the reaction mixture was cooled to 0 °C and 5-bromo-2-methoxybenzaldehyde (151 mg, 0.70 mmol, 1.4 equiv) was added dropwise and the reaction mixture was stirred for 0.5 h at 0 °C and then allowed to warm to room temperature and stirred for 1 h. Purification of the crude product by flash column chromatography (silica gel, *i*hexane/EtOAc = 6:1) afforded the title compound as a white solid (**9v**, 157 mg, 0.36 mmol, 72% yield).

^{1}H NMR (400 MHz, $CDCl_3$): δ (ppm) = 7.79 (dd, *J* = 2.6, 1.1 Hz, 1H), 7.30 (ddd, *J* = 8.6, 2.6, 0.7 Hz, 1H), 7.24 – 7.19 (m, 1H), 7.13 (ddd, *J* = 9.7, 8.2, 1.3 Hz, 1H), 6.89 – 6.85 (m, 1H), 6.60 (d, *J* = 8.6 Hz, 1H), 6.22 (dq, *J* = 11.4, 1.2 Hz, 1H), 6.08 (d, *J* = 11.2 Hz, 1H), 3.57 (s, 3H), 3.30 (dp, *J* = 25.2, 6.7 Hz, 2H), 1.46 (d, *J* = 6.9 Hz, 3H), 1.40 (d, *J* = 6.7 Hz, 3H), 1.06 (d, *J* = 6.6 Hz, 3H), 0.29 (d, *J* = 6.7 Hz, 3H).

^{13}C NMR (101 MHz, $CDCl_3$): δ (ppm) = 171.6 (d, *J* = 3.1 Hz), 161.0 (d, *J* = 249.0 Hz), 155.4, 137.8 (d, *J* = 2.2 Hz), 134.0 (d, *J* = 1.8 Hz), 131.0, 130.9, 130.5, 130.4, 128.3 (d, *J* = 9.3 Hz), 122.5 (d, *J* = 3.7 Hz), 116.5 (d, *J* = 24.9 Hz), 113.5, 112.0, 64.0 (d, *J* = 8.7 Hz), 60.5, 55.7, 51.5, 46.5, 21.3, 20.2, 20.2, 20.0.

^{19}F NMR (376 MHz, $CDCl_3$): δ (ppm) = -114.1.

MS (EI, 70 eV): m/z (%) = 404 (36), 241 (59), 220 (40), 183 (100), 170 (81).

HRMS (EI): for $C_{21}H_{25}BrFNO_3$: calc. [M+]: 437.1002; found: 437.0993.

3-fluoro-2-formyl-N,N-diisopropylbenzamide (9w)

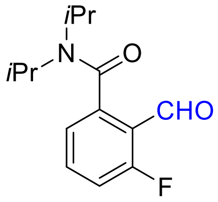

According to **TP 1**, to a mixture of 3-fluoro-*N*,*N*-diisopropylbenzamide (**5p**, 670 mg, 3.00 mmol, 1.0 equiv) in toluene (6 mL) was added MBDA (3.30 mmol, 1.1 equiv) at 25 °C. After 10 min, the reaction mixture was cooled to 0 °C and DMF (0.69 mL, 9.00 mmol, 3.0 equiv) was added dropwise and the reaction mixture was stirred for 1 h at room temperature. Purification of the crude product by flash column chromatography (silica gel, *i*hexane/EtOAc = 2:1) afforded the title compound as a white solid (**9w**, 477 mg, 1.90 mmol, 63% yield).

^{1}H NMR (400 MHz, $CDCl_3$): δ (ppm) = 10.37 (s, 1H), 7.58 (ddd, *J* = 8.4, 7.6, 5.3 Hz, 1H), 7.16 (ddd, *J* = 10.5, 8.4, 1.0 Hz, 1H), 7.03 (d, *J* = 7.5 Hz, 1H), 3.52 (dp, *J* = 9.6, 6.8 Hz, 2H), 1.60 (d, *J* = 6.9 Hz, 7H), 1.10 (d, *J* = 6.7 Hz, 7H).
^{13}C NMR (101 MHz, $CDCl_3$): δ (ppm) = 186.8, 167.9, 164.8 (d, *J* = 260.2 Hz), 141.0, 136.1, 122.3 (d, *J* = 3.8 Hz), 120.9 (d, *J* = 8.6 Hz), 116.4 (d, *J* = 20.6 Hz), 51.4 (d, *J* = 7.4 Hz), 46.1 (d, *J* = 7.5 Hz), 20.5.
^{19}F NMR (376 MHz, $CDCl_3$): δ (ppm) = -119.0.
MS (EI, 70 eV): m/z (%) = 208 (13), 166 (23), 151 (100), 123 (31), 100 (10), 75 (10).
HRMS (EI): for $C_{14}H_{18}FNO_2$: calc. [M+]: 251.1322; found: 251.1324.

2,3-difluoro-N,N-diisopropyl-6-(quinolin-6-yl)benzamide (9x)

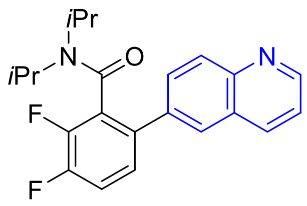

According to **TP 1**, to a mixture of 2,3-difluoro-N,N-diisopropylbenzamide (**5q**, 121 mg, 0.50 mmol, 1.0 equiv) in toluene (1 mL) was added MBDA (0.55 mmol, 1.1 equiv) at 25 °C. After 0.5 h, the resulting diarylmagnesium was transmetalated with a $ZnCl_2$ solution (0.70 mL, 1.00 M in THF, 1.4 equiv) at 0 °C for 30 min. A dry and argon-flushed Schlenk-tube, equipped with a magnetic stirring bar and a septum was charged with $Pd(dba)_2$ (8 mg, 3 mol%), tfp (7 mg, 6 mol%) and 6-iodoquinoline (106 mg, 0.42 mmol, 0.83 equiv).The freshly prepared arylzinc reagent was added and the reaction mixture was placed in an oil bath at 55 °C for 12 h. Purification of the crude product by flash column chromatography (silica gel, *i*hexane/EtOAc = 1:1) afforded the title compound as a yellow solid (**9x**, 99 mg, 0.27 mmol, 65% yield).

^{1}H NMR (400 MHz, $CDCl_3$): δ (ppm) = 8.96 (dd, *J* = 4.2, 1.7 Hz, 1H), 8.22–8.13 (m, 2H), 8.10 (d, *J* = 2.1 Hz, 1H), 7.85 (dd, *J* = 8.7, 2.1 Hz, 1H), 7.46 (dd, *J* = 8.3, 4.3 Hz, 1H), 7.32–7.23 (m, 2H), 3.52 (hept, *J* = 6.6 Hz, 1H), 3.23 (hept, *J* = 6.8 Hz, 1H), 1.54 (d, *J* = 6.8 Hz, 3H), 1.14 (d, *J* = 6.8 Hz, 3H), 1.03 (d, *J* = 6.7 Hz, 3H), 0.33 (d, *J* = 6.6 Hz, 3H).

^{13}C NMR (101 MHz, $CDCl_3$): δ (ppm) = 163.5 (d, *J* = 2.5 Hz), 151.1, 150.7 (dd, *J* = 249.4, 13.7 Hz), 147.7, 146.9 (dd, *J* = 249.4, 14.0 Hz), 136.6, 136.2 (d, *J* = 1.9 Hz), 134.5–134.4 (m), 130.8, 129.8, 128.5, 128.3, 128.0, 125.8 (dd, *J* = 6.3, 3.7 Hz), 121.9, 117.1 (d, *J* = 17.2 Hz), 51.3, 46.2, 20.9, 20.5, 20.0, 19.8.

^{19}F NMR (376 MHz, $CDCl_3$): δ (ppm) = -137.5, -141.0.

MS (EI, 70 eV): m/z (%) = 333 (12), 269 (15), 268 (100), 240 (14).

HRMS (EI): for $C_{22}H_{22}F_2N_2O$: calc. [M+]: 368.1700; found: 368.1695.

2-(5-fluoro-3'-((5-(4-fluorophenyl)thiophen-2-yl)methyl)-4'-methyl-[1,1'-biphenyl]-2-yl)-4,4-dimethyl-4,5-dihydrooxazole (9y)

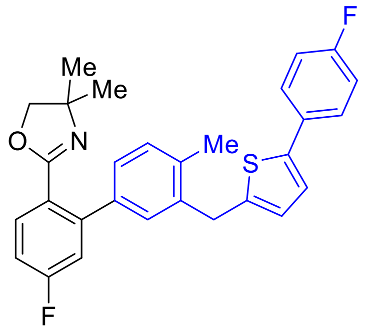

According to **TP 1**, to a mixture of 2-(4-fluorophenyl)-4,4-dimethyl-4,5-dihydrooxazole (**5r**, 163 mg, 0.84 mmol, 1.0 equiv) in toluene (1.70 mL) was added MBDA (0.92 mmol, 1.1 equiv) at 60 °C. After 0.5 h, the resulting diarylmagnesium was transmetalated with a $ZnCl_2$ solution (1.0 mL, 1.00 M in THF, 1.4 equiv) at 0 °C for 30 min. A dry and argon-flushed Schlenk-tube, equipped with a magnetic stirring bar and a septum was charged with $Pd(dba)_2$ (15 mg, 3 mol%), tfp (12 mg, 6 mol%) and 2-(4-fluorophenyl)-5-(5-iodo-2-methylbenzyl)thiophene (280 mg, 0.69 mmol, 0.83 equiv). The freshly prepared arylzinc reagent was added and the reaction mixture was placed in an oil bath at 55 °C for 12 h. Purification of the crude product by flash column chromatography (silica gel, *i*hexane/EtOAc = 9:1 to 1:1) afforded the title compound as a yellow oil (**9y**, 313 mg, 0.66 mmol, 96% yield).

^{1}H NMR (400 MHz, $CDCl_3$): δ (ppm) = 7.74 (dd, *J* = 8.6, 5.8 Hz, 1H), 7.50–7.45 (m, 2H), 7.27 (t, *J* = 1.2 Hz, 1H), 7.23 (d, *J* = 1.3 Hz, 2H), 7.09 (dd, *J* = 9.5, 2.6 Hz, 1H), 7.07–7.00 (m, 4H), 6.67 (dd, *J* = 3.6, 1.2 Hz, 1H), 4.15 (d, *J* = 1.3 Hz, 2H), 3.77 (s, 2H), 2.38 (s, 3H), 1.26 (s, 6H).
^{13}C NMR (101 MHz, $CDCl_3$): δ (ppm) = 163.8 (d, *J* = 239.9 Hz),163.3, 162.1 (d, *J* = 236.2 Hz), 144.0 (d, *J* = 8.4 Hz), 143.3 (d, *J* = 1.0 Hz), 141.7, 138.2 (d, *J* = 1.7 Hz), 138.0, 136.1, 132.7 (d, *J* = 9.0 Hz), 130.9 (d, *J* = 3.4 Hz), 130.5, 129.5, 127.2 (d, *J* = 8.0 Hz), 126.8, 126.0, 124.0 (d, *J* = 3.0 Hz), 122.7 (d, *J* = 1.2 Hz), 117.3 (d, *J* = 22.0 Hz), 115.8 (d, *J* = 21.7 Hz), 114.0 (d, *J* = 21.5 Hz), 79.6, 67.4, 34.3, 28.1, 19.4.
^{19}F NMR (376 MHz, $CDCl_3$): δ (ppm) = -109.9, -115.0.
MS (EI, 70 eV): m/z (%) = 240 (19), 239 (16), 222 (13), 196 (29), 191 (100), 178 (16), 139 (22).
HRMS (EI): for $C_{29}H_{25}F_2NOS$: calc. [M-H$^+$]: 472.1547; found: 472.1539.

(E)-(2,6-difluoro-3-(pyrrolidin-1-yldiazenyl)phenyl)(furan-2-yl)methanol (9z)

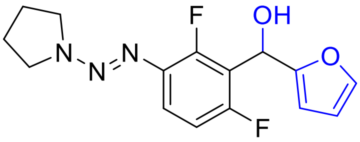

According to TP 1, to a mixture of (E)-1-((2,4-difluorophenyl)diazenyl)pyrrolidine (**5s**, 211 mg, 1.00 mmol, 1.0 equiv) in toluene (2 mL) was added MBDA (1.10 mmol, 1.1 equiv) at 0 °C. After 1 h, furfural (0.12 mL, 1.40 mmol, 1.4 equiv) was added dropwise and the reaction mixture was stirred for 0.5 h at 0 °C and then allowed to warm to room temperature and stirred for 1 h. Purification of the crude product by flash column chromatography (silica gel, ihexane/EtOAc = 8:1 to 4:1) afforded the title compound as a brown oil (**9z**, 173 mg, 0.60 mmol, 60% yield).

^{1}H NMR (400 MHz, CDCl$_3$): δ (ppm) = 7.44–7.37 (m, 2H), 6.86 (td, *J* = 9.4, 1.7 Hz, 1H), 6.32 (dd, *J* = 3.3, 1.8 Hz, 1H), 6.23 (d, *J* = 3.3 Hz, 1H), 6.19 (s, 1H), 4.06–3.52 (m, 4H), 2.13–1.97 (m, 4H).
^{13}C NMR (101 MHz, CDCl$_3$): δ (ppm) = 157.9 (dd, *J* = 239.0, 5.9 Hz), 154.1, 154.1 (dd, *J* = 243.3, 7.9 Hz), 142.7, 119.1 (dd, *J* = 10.0, 3.6 Hz), 117.4–116.9 (m), 111.5 (dd, *J* = 23.2, 3.9 Hz), 110.5, 107.3, 63.0–62.0 (m).
^{19}F NMR (376 MHz, CDCl$_3$): δ (ppm) = -120.3, -129.1.
MS (EI, 70 eV): m/z (%) = 239 (27), 140 (100), 94 (26), 84 (25), 70 (27), 40 (34).
HRMS (EI): for $C_{15}H_{15}F_2N_3O_2$: calc. [M+]: 307.1132; found: 307.1141.

(E)-1-((2,6-difluoro-3'-methyl-[1,1'-biphenyl]-3-yl)diazenyl)pyrrolidine (9aa)

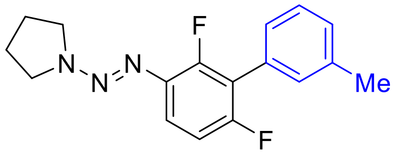

According to **TP 1**, to a mixture of (E)-1-((2,4-difluorophenyl)diazenyl)pyrrolidine (**5s**, 845 mg, 4.00 mmol, 1.0 equiv) in toluene (8.0 mL) was added MBDA (4.40 mmol, 1.1 equiv) at 0 °C. After 1 h, the resulting diarylmagnesium was transmetalated with a $ZnCl_2$ solution (5.6 mL, 1.00 M in THF, 1.4 equiv) at 0 °C for 30 min. A dry and argon-flushed Schlenk-tube, equipped with a magnetic stirring bar and a septum was charged with $Pd(dba)_2$ (64 mg, 3 mol%), tfp (56 mg, 6 mol%) and 3-iodotoluene (0.43 mL, 3.32 mmol, 0.83 equiv). The freshly prepared arylzinc reagent was added and the reaction mixture was placed in an oil bath at 55 °C for 12 h. Purification of the crude product by flash column chromatography (silica gel, *i*hexane/EtOAc = 98:2) afforded the title compound as an orange solid (**9aa**, 741 mg, 2.46 mmol, 74% yield).

^{1}H NMR (400 MHz, $CDCl_3$): δ (ppm) = 7.43–7.28 (m, 4H), 7.21 (ddt, J = 7.5, 2.2, 1.0 Hz, 1H), 6.91 (td, J = 9.0, 1.8 Hz, 1H), 3.82 (d, J = 70.6 Hz, 4H), 2.41 (s, 3H), 2.11–1.98 (m, 4H).
^{13}C NMR (101 MHz, $CDCl_3$): δ (ppm) = 157.4 (dd, *J* = 245.5, 6.2 Hz), 153.3 (dd, *J* = 251.5, 7.0 Hz), 137.9, 136.6 (dd, *J* = 9.1, 3.8 Hz), 131.2 (t, *J* = 1.8 Hz), 129.7, 129.0, 128.2, 127.5 (t, *J* = 1.9 Hz), 119.0 (dd, *J* = 19.6, 18.0 Hz), 118.1 (dd, *J* = 9.6, 3.3 Hz), 111.1 (dd, *J* = 23.7, 4.0 Hz), 23.9, 21.6.
^{19}F NMR (376 MHz, $CDCl_3$): δ (ppm) = -119.6, -128.4.
MS (EI, 70 eV): m/z (%) = 231 (26), 203 (100), 201 (14), 188 (48), 183 (21).
HRMS (EI): for $C_{17}H_{17}F_2N_3$: calc. [M+]: 301.1391; found: 301.1377.

3-fluoro-2,4-diiodopyridine (10a)

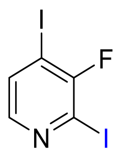

According to **TP 1**, to a mixture of 3-fluoro-4-iodopyridine (**6a**, 112 mg, 0.50 mmol, 1.0 equiv) in toluene (1 mL) was added MBDA (0.30 mmol, 0.6 equiv) at 0 °C. After 5 min, the reaction mixture was cooled to 0 °C and iodine (152 mg, 0.60 mmol, 1.2 equiv) dissolved in THF (1 mL) was added dropwise and the reaction mixture was stirred for 1 h. Purification of the crude product by flash column chromatography (silica gel, *i*hexane/EtOAc = 99:1) afforded the title compound as a brown solid (**10a**, 142 mg, 0.41 mmol, 82% yield).

^{1}H NMR (400 MHz, $CDCl_3$): δ (ppm) = 7.84 (d, *J* = 4.9 Hz, 1H), 7.65 (t, *J* = 4.7 Hz, 1H).
^{13}C NMR (101 MHz, $CDCl_3$): δ (ppm) = 158.7 (d, *J* = 255.4 Hz), 147.0 (d, *J* = 6.2 Hz), 134.1, 105.4 (d, *J* = 31.8 Hz), 91.1 (d, *J* = 26.2 Hz).
^{19}F NMR (376 MHz, $CDCl_3$): δ (ppm) = -81.9.
MS (EI, 70 eV): m/z (%) = 70 (11), 61 (13), 45 (14), 44 (14), 43 (100).
HRMS (EI): for $C_5H_2FI_2N$: calc. [M+]: 348.8261; found: 348.8255.

(3-fluoro-4-iodopyridin-2-yl)(phenyl)methanol (10b)

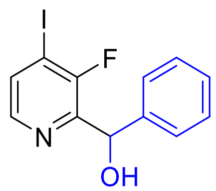

According to TP 1, to a mixture of 3-fluoro-4-iodopyridine (**6a**, 112 mg, 0.50 mmol, 1.0 equiv) in toluene (1 mL) was added MBDA (0.30 mmol, 0.6 equiv) at 0 °C. After 5 min, the reaction mixture was cooled to 0 °C and benzaldehyde (78 µL, 0.70 mmol, 1.4 equiv) was added dropwise and the reaction mixture was stirred for 0.5 h at 0 °C and then allowed to warm to room temperature and stirred for 1 h. Purification of the crude product by flash column chromatography (silica gel, ihexane/EtOAc = 9:1) afforded the title compound as a white solid (**10b**, 120 mg, 0.37 mmol, 74% yield).

^{1}H NMR (400 MHz, $CDCl_3$): δ (ppm) = 8.04 (d, *J* = 5.0 Hz, 1H), 7.66 (t, *J* = 4.8 Hz, 1H), 7.42–7.26 (m, 6H), 5.97 (d, *J* = 2.2 Hz, 1H).

^{13}C NMR (101 MHz, $CDCl_3$): δ (ppm) = 156.3 (d, *J* = 256.3 Hz), 149.2 (d, *J* = 19.0 Hz), 144.4 (d, *J* = 6.2 Hz), 141.7, 133.9, 128.7, 128.2, 127.8, 127.1, 126.9 (d, *J* = 1.7 Hz), 93.7 (d, *J* = 23.3 Hz), 70.5 (d, *J* = 3.0 Hz).

^{19}F NMR (376 MHz, $CDCl_3$): δ (ppm) = -106.9.

MS (EI, 70 eV): m/z (%) = 328 (16), 61 (11), 45 (10), 44 (100).

HRMS (EI): for $C_{12}H_9FINO$: calc. [M+]: 328.9713; found: 328.9692.

2-(cyclohex-2-en-1-yl)-3-fluoro-4-iodopyridine (10c)

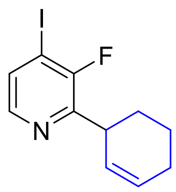

According to **TP 1**, to a mixture of 3-fluoro-4-iodopyridine (**6a**, 112 mg, 0.50 mmol, 1.0 equiv) in toluene (1 mL) was added MBDA (0.30 mmol, 0.6 equiv) at 0 °C. After 5 min, the reaction mixture was cooled to -25 °C and CuCN·2LiCl (0.1 mL, 20 mol%, 1M in THF) and 3-bromocyclohexen (70 µL, 0.6 mmol, 1.2 equiv) were added and the mixture was stirred for 0.5 h at -25 °C. Purification of the crude product by flash column chromatography (silica gel, ihexane/EtOAc = 97:3) afforded the title compound as a colorless oil (**10c**, 127 mg, 0.42 mmol, 84% yield).

^{1}H NMR (400 MHz, $CDCl_3$): δ (ppm) = 8.00 (d, *J* = 5.0 Hz, 1H), 7.54 (t, *J* = 4.7 Hz, 1H), 5.97 (ddt, *J* = 10.1, 5.0, 2.7 Hz, 1H), 5.75–5.66 (m, 1H), 3.91 (qp, *J* = 5.1, 2.5 Hz, 1H), 2.24–1.98 (m, 3H), 1.90–1.82 (m, 1H), 1.77–1.64 (m, 2H).
^{13}C NMR (101 MHz, $CDCl_3$): δ (ppm) = 157.3 (d, *J* = 255.9 Hz), 153.5 (d, *J* = 16.5 Hz), 145.6 (d, *J* = 6.6 Hz), 132.3, 129.3, 127.3 (d, *J* = 1.3 Hz), 92.7 (d, *J* = 24.2 Hz), 37.9 (d, *J* = 2.2 Hz), 28.9, 24.8, 21.7.
^{19}F NMR (376 MHz, $CDCl_3$): δ (ppm) = -107.7.
MS (EI, 70 eV): m/z (%) = 207 (6), 70 (11), 63 (18), 61 (13), 44 (12), 41 (100).
HRMS (EI): for $C_{11}H_{11}FIN$: calc. [M^{+}]: 302.9920; found: 302.9908.

6-chloro-3-fluoro-2-iodopyridine (10d)

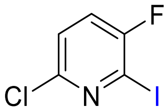

According to **TP 1**, to a mixture of 2-chloro-5-fluoropyridine (**6b**, 49 µL, 0.50 mmol, 1.0 equiv) in toluene (1 mL) was added MBDA (0.40 mmol, 0.8 equiv) at 25 °C. After 10 min, the reaction mixture was cooled to 0 °C and iodine (152 mg, 0.60 mmol, 1.2 equiv) dissolved in THF (1 mL) was added dropwise and the reaction mixture was stirred for 1 h. Purification of the crude product by flash column chromatography (silica gel, *i*hexane/EtOAc = 99:1) afforded the title compound as a colorless oil (**10d**, 96 mg, 0.38 mmol, 75% yield).

^{1}H NMR (400 MHz, $CDCl_3$): δ (ppm) = 7.25–7.19 (m, 2H).
^{13}C NMR (101 MHz, $CDCl_3$): δ (ppm) = 158.9 (d, *J* = 257.2 Hz), 145.6 (d, *J* = 3.0 Hz), 125.0, 124.8 (d, *J* = 3.2 Hz), 124.7, 104.6 (d, *J* = 31.2 Hz).
^{19}F NMR (376 MHz, $CDCl_3$): δ (ppm) = -105.1.
MS (EI, 70 eV): m/z (%) = 256 (27), 131 (32), 129 (100), 109 (35), 94 (12).
HRMS (EI): for C_5H_2ClFIN: calc. [M^+]: 256.8904; found: 256.8897.

4-(6-chloro-3-fluoropyridin-2-yl)benzonitrile (10e)

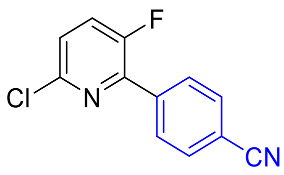

According to **TP 1**, to a mixture of 2-chloro-5-fluoropyridine (**6b**, 49 µL, 0.50 mmol, 1.0 equiv) in toluene (1 mL) was added MBDA (0.40 mmol, 0.8 equiv) at 25 °C. After 10 min, the resulting diarylmagnesium was transmetalated with a $ZnCl_2$ solution (0.70 mL, 1.00 M in THF, 1.4 equiv) at 0 °C for 30 min. A dry and argon-flushed Schlenk-tube, equipped with a magnetic stirring bar and a septum was charged with $Pd(dba)_2$ (8 mg, 3 mol%), tfp (7 mg, 6 mol%) and 4-iodobenzonitrile (120 mg, 0.42 mmol, 0.83 equiv).The freshly prepared arylzinc reagent was added and the reaction mixture was placed in an oil bath at 55 °C for 12 h. Purification of the crude product by flash column chromatography (silica gel, *i*hexane/EtOAc = 9:1) afforded the title compound as a white solid (**10e**, 96 mg, 0.41 mmol, 95% yield).

^{1}H NMR (400 MHz, $CDCl_3$): δ (ppm) = 8.18–8.10 (m, 2H), 7.81–7.74 (m, 2H), 7.53 (dd, J = 10.0, 8.6 Hz, 1H), 7.35 (dd, J = 8.6, 3.1 Hz, 1H).

^{13}C NMR (101 MHz, $CDCl_3$): δ (ppm) = 157.0 (d, *J* = 261.3 Hz), 146.0 (d, *J* = 2.7 Hz), 143.6 (d, *J* = 12.4 Hz), 138.1 (d, *J* = 5.8 Hz), 132.4, 129.5 (d, *J* = 7.1 Hz), 128.0 (d, *J* = 23.1 Hz), 125.6 (d, *J* = 5.1 Hz), 118.6, 113.4.

^{19}F NMR (376 MHz, $CDCl_3$): δ (ppm) = -125.3.

MS (EI, 70 eV): m/z (%) = 234 (24), 232 (81), 205 (13), 198 (12), 197 (100), 177 (21), 170 (28), 150 (26).

HRMS (EI): for $C_{12}H_6ClFN_2$: calc. [M+]: 232.0204; found: 232.0196.

(1r,5R,7S)-2-(6-bromo-3-fluoropyridin-2-yl)adamantan-2-ol (10f)

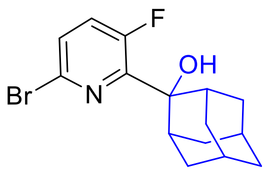

According to **TP 1**, to a mixture of 2-bromo-5-fluoropyridine (**6c**, 88 mg, 0.50 mmol, 1.0 equiv) in toluene (1 mL) was added MBDA (0.40 mmol, 0.8 equiv) at 25 °C. After 0.5 h, the reaction mixture was cooled to 0 °C and adamantan-2-one (105 mg, 0.70 mmol, 1.4 equiv) dissolved in toluene (1 mL) was added dropwise and the reaction mixture was stirred for 1 h. Purification of the crude product by flash column chromatography (silica gel, *i*hexane/EtOAc = 94:6) afforded the title compound as a white solid (**10f**, 122 mg, 0.38 mmol, 76% yield).

^{1}H NMR (400 MHz, $CDCl_3$): δ (ppm) = 7.40 (dd, *J* = 8.4, 3.1 Hz, 1H), 7.32–7.27 (m, 1H), 2.76 (s, 2H), 2.38 (dd, *J* = 12.8, 3.1 Hz, 3H), 2.22 (s, 1H), 1.94–1.68 (m, 10H).
^{13}C NMR (101 MHz, $CDCl_3$): δ (ppm) = 158.0 (d, *J* = 261.1 Hz), 153.0 (d, *J* = 11.5 Hz), 134.1 (d, *J* = 2.5 Hz), 128.2 (d, *J* = 4.9 Hz), 127.8 (d, *J* = 24.0 Hz), 78.2 (d, *J* = 4.6 Hz), 37.7, 35.1, 35.0, 34.9, 32.8, 27.0, 26.9.
^{19}F NMR (376 MHz, $CDCl_3$): δ (ppm) = -121.8.
MS (EI, 70 eV): m/z (%) = 61 (12), 44 (10), 42 (100).
HRMS (EI): for $C_{15}H_{17}BrFNO$: calc. [M^{+}]: 325.0478; found: 325.0464.

(3-fluoropyridin-2-yl)(furan-2-yl)methanol (10g)

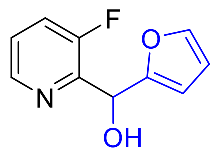

According to **TP 1**, to a mixture of 3-fluoropyridine (**6d**, 43 µL, 0.50 mmol, 1.0 equiv) in toluene (1 mL) was added MBDA (0.30 mmol, 0.6 equiv) at 0 °C. After 15 min, the reaction mixture was cooled to 0 °C and furfural (58 µL, 0.70 mmol, 1.4 equiv) was added dropwise and the reaction mixture was stirred for 0.5 h at 0 °C and then allowed to warm to room temperature and stirred for 1 h. Purification of the crude product by flash column chromatography (silica gel, ihexane/EtOAc = 9:1) afforded the title compound as a white solid (**10g**, 71 mg, 0.37 mmol, 74% yield).

^{1}H NMR (400 MHz, $CDCl_3$): δ (ppm) = 8.45 (d, J = 4.7 Hz, 1H), 7.47–7.29 (m, 3H), 6.37–6.28 (m, 1H), 6.20 (d, J = 3.3 Hz, 1H), 6.09–6.01 (m, 1H), 5.58–4.84 (m, 1H).
^{13}C NMR (101 MHz, $CDCl_3$): δ (ppm) = 156.5 (d, *J* = 258.3 Hz), 154.1, 146.4 (d, *J* = 16.1 Hz), 144.1 (d, *J* = 5.2 Hz), 142.8, 124.7 (d, *J* = 3.7 Hz), 123.8 (d, *J* = 18.2 Hz), 110.4, 107.6, 64.0 (d, *J* = 1.8 Hz).
^{19}F NMR (376 MHz, $CDCl_3$): δ (ppm) = -125.7.
MS (EI, 70 eV): m/z (%) = 193 (46), 164 (100), 136 (55), 97 (56), 81 (36) 68 (41).
HRMS (EI): for $C_{10}H_8FNO_2$: calc. [M+]: 193.0539; found: 193.0532.

(6-bromo-5,7-difluoroquinolin-8-yl)(thiazol-2-yl)methanol (10h)

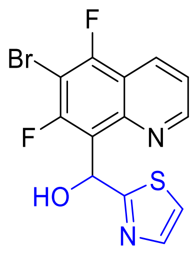

According to **TP 1**, to a mixture of 6-bromo-5,7-difluoroquinoline (**6e**, 122 mg, 0.50 mmol, 1.0 equiv) in toluene (1 mL) was added MBDA (0.40 mmol, 0.8 equiv) at 25 °C. After 0.5 h, the reaction mixture was cooled to 0 °C and thiazole-2-carbaldehyde (70 µL, 0.70 mmol, 1.4 equiv) was added dropwise and the reaction mixture was stirred for 0.5 h at 0 °C and then allowed to warm to room temperature and stirred for 1 h. Purification of the crude product by flash column chromatography (silica gel, ihexane/EtOAc = 3:1) afforded the title compound as a yellow solid (**10h**, 121 mg, 0.34 mmol, 68% yield).

^{1}H NMR (400 MHz, CDCl$_3$): δ (ppm) = 8.83 (dd, *J* = 4.4, 1.8 Hz, 1H), 8.43 (dd, *J* = 8.6, 1.8 Hz, 1H), 7.70 (d, *J* = 11.1 Hz, 1H), 7.61 (d, *J* = 3.3 Hz, 1H), 7.48 (dd, *J* = 8.5, 4.4 Hz, 1H), 6.70 (d, *J* = 11.1 Hz, 1H).

^{13}C NMR (101 MHz, CDCl$_3$): δ (ppm) = 175.3 (d, *J* = 2.4 Hz), 155.7 (ddd, *J* =255.6,183.2, 57.4, 6.8 Hz), 150.7, 145.6 (dd, *J* = 8.6, 4.2 Hz), 142.8, 130.5 (dd, *J* = 4.0, 2.1 Hz), 121.4 (t, *J* = 2.6 Hz), 119.6, 119.0 (dd, *J* = 16.6, 5.4 Hz), 117.2 (dd, *J* = 17.2, 1.8 Hz), 97.7 (d, *J* = 23.5 Hz), 97.4 (d, *J* = 23.5 Hz), 69.2 (d, *J* = 4.5 Hz).

^{19}F NMR (376 MHz, CDCl$_3$): δ (ppm) = -105.6, -111.7.

MS (EI, 70 eV): m/z (%) = 271 (15), 244 (10), 242 (12), 163 (11), 86 (26), 70 (11), 61 (14), 45 (14), 43 (100).

HRMS (EI): for $C_{13}H_7BrF_2N_2OS$: calc. [M+]: 355.9431; found: 355.9421.

(3-chloropyrazin-2-yl)dicyclopropylmethanol (10i)

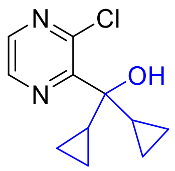

According to **TP 1**, to a mixture of 2-chloropyrazine (**6f**, 45 µL, 0.50 mmol, 1.0 equiv) in toluene (1 mL) was added MBDA (0.40 mmol, 0.8 equiv) at -25 °C. After 0.5 h, the reaction mixture dicyclopropylketone (79 µL, 0.70 mmol, 1.4 equiv) was added dropwise and the reaction mixture was stirred for 0.5 h at 0 °C and then allowed to warm to room temperature and stirred for 1 h. Purification of the crude product by flash column chromatography (silica gel, ihexane/EtOAc = 9:1) afforded the title compound as a yellow oil (**10i**, 75 mg, 0.34 mmol, 68% yield).

^{1}H NMR (400 MHz, $CDCl_3$): δ (ppm) = 8.37 (s, 2H), 4.84 (s, 1H), 1.89 (tt, *J* = 8.3, 5.2 Hz, 2H), 0.79 (dtd, *J* = 9.5, 5.6, 4.1 Hz, 2H), 0.48 (tdd, *J* = 8.7, 6.0, 4.0 Hz, 2H), 0.20 – 0.05 (m, 4H).
^{13}C NMR (101 MHz, $CDCl_3$): δ (ppm) = 158.5, 147.3, 142.5, 139.1, 71.6, 17.2, 1.5, -0.7.
MS (EI, 70 eV): m/z (%) = 185 (26), 183 (82), 181 (23), 155 (21), 142 (29), 140 (90), 128 (64), 112 (69), 111 (100), 69 (74).
HRMS (EI): for $C_{11}H_{13}ClN_2O$: calc. [M+]: 224.0716; found: 224.0711.

3,5-dichloro-2-(1-methyl-1H-pyrazol-4-yl)pyrazine (10j)

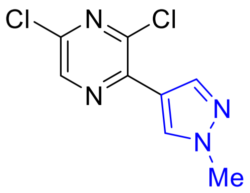

According to **TP 1**, to a mixture of 2,6-dichloropyrazine (**6g**, 75 mg, 0.50 mmol, 1.0 equiv) in toluene (1 mL) was added MBDA (0.40 mmol, 0.8 equiv) at -20 °C. After 15 min, the resulting diarylmagnesium was transmetalated with a $ZnCl_2$ solution (0.70 mL, 1.00 M in THF, 1.4 equiv) at 0 °C for 30 min. A dry and argon-flushed Schlenk-tube, equipped with a magnetic stirring bar and a septum was charged with $Pd(dba)_2$ (8 mg, 3 mol%), tfp (7 mg, 6 mol%) and 4-iodo-1-methyl-1H-pyrazole (86 mg, 0.42 mmol, 0.83 equiv).The freshly prepared arylzinc reagent was added and the reaction mixture was placed in an oil bath at 55 °C for 12 h. Purification of the crude product by flash column chromatography (silica gel, *i*hexane/EtOAc = 3:1) afforded the title compound as a yellow solid (**10j**, 79 mg, 0.35 mmol, 84% yield).

^{1}H NMR (400 MHz, $CDCl_3$): δ (ppm) = 8.45 (s, 1H), 8.24 (s, 1H), 8.19 (s, 1H), 3.99 (s, 3H).
^{13}C NMR (101 MHz, $CDCl_3$): δ (ppm) = 144.8, 143.0, 142.7, 141.9, 140.5, 131.8, 118.0, 39.5.
MS (EI, 70 eV): m/z (%) = 229 (63), 227 (100), 195 (29), 193 (91), 168 (14), 166 (43).
HRMS (EI): for $C_8H_6Cl_2N_4$: calc. [M^+]: 227.9970; found: 227.9963.

3,5-dichloro-2-(3-((5-(4-fluorophenyl)thiophen-2-yl)methyl)-4-methylphenyl)pyrazine (10k)

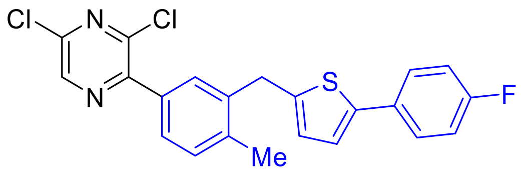

According to **TP 1**, to a mixture of 2,6-dichloropyrazine (**6g**, 75 mg, 0.50 mmol, 1.0 equiv) in toluene (1 mL) was added MBDA (0.40 mmol, 0.8 equiv) at -20 °C. After 15 min, the resulting diarylmagnesium was transmetalated with a $ZnCl_2$ solution (0.70 mL, 1.00 M in THF, 1.4 equiv) at 0 °C for 30 min. A dry and argon-flushed Schlenk-tube, equipped with a magnetic stirring bar and a septum was charged with $Pd(dba)_2$ (8 mg, 3 mol%), tfp (7 mg, 6 mol%) and 2-(4-fluorophenyl)-5-(5-iodo-2-methylbenzyl)thiophene (169 mg, 0.42 mmol, 0.83 equiv).The freshly prepared arylzinc reagent was added and the reaction mixture was placed in an oil bath at 55 °C for 12 h. Purification of the crude product by flash column chromatography (silica gel, *i*hexane/EtOAc = 99:1) afforded the title compound as a yellow oil (**10k**, 146 mg, 0.34 mmol, 82% yield).

^{1}H NMR (400 MHz, $CDCl_3$): δ (ppm) = 8.57 (s, 1H), 7.67 (d, *J* = 2.0 Hz, 1H), 7.64 (dd, *J* = 7.8, 2.0 Hz, 1H), 7.51–7.45 (m, 2H), 7.32 (d, *J* = 7.8 Hz, 1H), 7.07–6.99 (m, 3H), 6.74–6.69 (m, 1H), 4.20 (s, 2H), 2.41 (s, 3H).

^{13}C NMR (101 MHz, $CDCl_3$): δ (ppm) = 162.1 (d, *J* = 246.8 Hz), 151.1, 145.4, 145.0, 142.6, 141.9, 141.8, 138.6 (d, *J* = 36.6 Hz), 133.0, 130.8 (d, *J* = 3.4 Hz), 130.5 (d, *J* = 13.6 Hz), 128.0, 127.2 (d, *J* = 8.0 Hz), 126.3, 122.8 (d, *J* = 1.2 Hz), 115.9, 115.6, 34.1, 19.5.

^{19}F NMR (376 MHz, $CDCl_3$): δ (ppm) = -114.9.

MS (EI, 70 eV): m/z (%) = 430 (62), 429 (29), 428 (90), 413 (20), 252 (64), 250 (100), 191 (53), 178 (20).

HRMS (EI): for $C_{22}H_{15}Cl_2FN_2S$: calc. [M+]: 428.0317; found: 428.0312.

3,3'-(2-methylenepropane-1,3-diyl)bis(2-(methylthio)pyrazine) (10l)

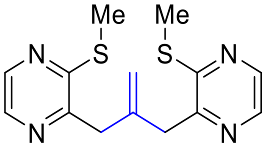

According to **TP 1**, to a mixture of 2-(methylthio)pyrazine (**6h**, 63 mg, 0.50 mmol, 1.0 equiv) in toluene (1 mL) was added MBDA (0.40 mmol, 0.8 equiv) at -10 °C. After 15 min, the reaction mixture was cooled to -25 °C and CuCN·2LiCl (0.1 mL, 20 mol%, 1M in THF) and 3-bromo-2-(bromomethyl)prop-1-ene (35 µL, 0.3 mmol, 0.6 equiv) were added and the mixture was stirred for 0.5 h at -25 °C. Purification of the crude product by flash column chromatography (silica gel, ihexane/EtOAc = 2:1) afforded the title compound as a brown solid (**10l**, 56 mg, 0.18 mmol, 72% yield).

^{1}H NMR (400 MHz, $CDCl_3$): δ (ppm) = 8.25 (d, *J* = 2.7 Hz, 2H), 8.16 (d, *J* = 2.7 Hz, 2H), 4.93 (t, *J* = 1.2 Hz, 2H), 3.64 (d, *J* = 1.3 Hz, 4H), 2.51 (s, 6H).
^{13}C NMR (101 MHz, $CDCl_3$): δ (ppm) = 156.7, 152.4, 141.6, 140.9, 138.3, 116.1, 41.3, 13.0.
MS (EI, 70 eV): m/z (%) = 289 (23), 257 (42), 179 (13), 165 (100), 150 (20), 131 (12).
HRMS (EI): for $C_{14}H_{16}N_4S_2$: calc. [M+]: 304.0816; found: 304.0811.

1-((2-chlorophenyl)diphenylmethyl)-2-((4-(trifluoromethyl)phenyl)ethynyl)-1H-imidazole (10m)

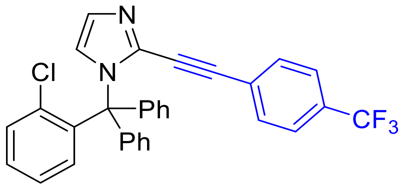

According to **TP 1**, to a mixture of clotrimazole (**6i**, 173 mg, 0.50 mmol, 1.0 equiv) in toluene (1 mL) was added MBDA (0.40 mmol, 0.8 equiv) at 0 °C. After 0.5 h, the resulting diarylmagnesium was transmetalated with a $ZnCl_2$ solution (0.70 mL, 1.00 M in THF, 1.4 equiv) at 0 °C for 0.5 h. Iodine (152 mg, 0.60 mmol, 1.2 equiv) dissolved in THF (1 mL) was added dropwise and the reaction mixture was stirred for 1 h. A dry and argon-flushed Schlenk-tube, equipped with a magnetic stirring bar and a septum was charged with $Pd(dba)_2$ (8 mg, 3 mol%), tfp (7 mg, 6 mol%), CuI (4 mg, 4 mol%)and Et_3N (3 mL). The freshly prepared arylzinc reagent followed by 1-ethynyl-4-(trifluoromethyl)benzene (0.11 mL, 0.7 mmol, 1.4 equiv) were added and the reaction mixture was placed in an oil bath at 55 °C for 2 h. Purification of the crude product by flash column chromatography (silica gel, DCM/MeOH = 98:2) afforded the title compound as a brown oil (**10m**, 175 mg, 0.34 mmol, 68% yield).

^{1}H NMR (400 MHz, $CDCl_3$): δ (ppm) = 7.46–7.40 (m, 3H), 7.31 (qd, J = 4.2, 3.6, 2.0 Hz, 8H), 7.24–7.17 (m, 5H), 7.11–7.06 (m, 2H), 7.04–6.98 (m, 2H).
^{13}C NMR (101 MHz, $CDCl_3$): δ (ppm) = 140.3, 139.6, 135.9, 133.0, 132.6, 131.9, 131.6, 130.8, 129.9, 128.2, 128.1, 127.9, 126.6, 126.0, 125.3, 125.2, 125.1 (q, J = 3.8 Hz), 125.1, 125.0, 123.9 (q, J = 271.6 Hz), 122.6, 92.5, 83.4, 76.4.
^{19}F NMR (376 MHz, $CDCl_3$): δ (ppm) = -62.9.
MS (EI, 70 eV): m/z (%) = 278 (36), 276 (100), 238 (31), 235 (36), 165 (62).
HRMS (EI): for $C_{31}H_{20}ClF_3N_2$: calc. [M^+]: 512.1267; found: 512.1262.

2,4-dibromo-5-iodothiazole (10n)

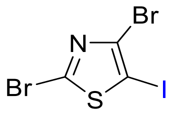

According to **TP 1**, to a mixture of 2,4-dibromothiazole (**6j**, 121 mg, 0.50 mmol, 1.0 equiv) in toluene (1 mL) was added MBDA (0.40 mmol, 0.8 equiv) at 25 °C. After 0.5 h, the reaction mixture was cooled to 0 °C and iodine (152 mg, 0.60 mmol, 1.2 equiv) dissolved in THF (1 mL) was added dropwise and the reaction mixture was stirred for 1 h. Purification of the crude product by flash column chromatography (silica gel, *i*hexane/EtOAc = 199:1) afforded the title compound as a brown solid (**10n**, 141 mg, 0.38 mmol, 76% yield).

^{1}H NMR (400 MHz, $CDCl_3$): δ (ppm) = no proton signals existing.
^{13}C NMR (101 MHz, $CDCl_3$): δ (ppm) = 140.3, 134.0, 74.7.
MS (EI, 70 eV): m/z (%) = 368 (12), 243 (48), 241 (100), 134 (77), 81 (22).
HRMS (EI): for C_3Br_2INS: calc. [M+]: 366.7163; found: 366.7158.

5-allyl-2,4-dibromothiazole (10o)

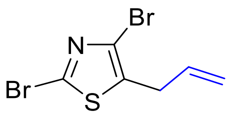

According to **TP 1**, to a mixture of 2,4-dibromothiazole (**6j**, 121 mg, 0.50 mmol, 1.0 equiv) in toluene (1 mL) was added MBDA (0.40 mmol, 0.8 equiv) at 25 °C. After 0.5 h, the reaction mixture was cooled to -25 °C and CuCN·2LiCl (0.1 mL, 20 mol%, 1M in THF) and allyl bromide (69 µL, 0.6 mmol, 1.2 equiv) were added and the mixture was stirred for 0.5 h at -25 °C. Purification of the crude product by flash column chromatography (silica gel, *i*hexane/EtOAc = 199:1) afforded the title compound as a yellow oil (**10o**, 135 mg, 0.48 mmol, 96% yield).

^{1}H NMR (400 MHz, CDCl$_3$): δ (ppm) = 5.94–5.82 (m, 1H), 5.23–5.19 (m, 1H), 5.17 (d, *J* = 1.4 Hz, 1H), 3.53–3.45 (m, 2H).

^{13}C NMR (101 MHz, CDCl$_3$): δ (ppm) = 136.6, 134.0, 133.4, 122.6, 118.4, 32.0.

MS (EI, 70 eV): m/z (%) = 282 (16), 203 (18), 201 (19), 124 (15), 123 (100).

HRMS (EI): for $C_6H_5Br_2NS$: calc. [M+]: 280.8509; found: 280.8504.

(4,5-dibromothiophen-2-yl)((S)-4-(prop-1-en-2-yl)cyclohex-1-en-1-yl)methanol (10p)

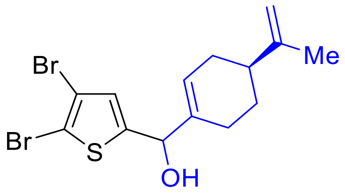

According to **TP 1**, to a mixture of 2,3-dibromothiophene (**6k**, 57 µL, 0.50 mmol, 1.0 equiv) in toluene (1 mL) was added MBDA (0.40 mmol, 0.8 equiv) at 40 °C. After 0.5 h, the reaction mixture was cooled to 0 °C and (*S*)-(-)-perillaldehyde (0.11 mL, 0.70 mmol, 1.4 equiv) was added dropwise and the reaction mixture was stirred for 0.5 h at 0 °C and then allowed to warm to room temperature and stirred for 1 h. Purification of the crude product by flash column chromatography (silica gel, ihexane/EtOAc = 9:1) afforded the title compound as a yellow oil (**10p**, 150 mg, 0.38 mmol, 76% yield).

^{1}H NMR (400 MHz, CDCl$_3$): δ (ppm) = 6.75 (dd, *J* = 6.0, 1.1 Hz, 1H), 5.91 (td, *J* = 4.8, 4.2, 1.6 Hz, 1H), 5.23 (t, *J* = 3.8 Hz, 1H), 4.77–4.70 (m, 2H), 2.27–1.92 (m, 5H), 1.84 (ddq, *J* = 12.7, 5.0, 2.0 Hz, 1H), 1.74 (s, 3H), 1.45 (tdd, *J* = 12.7, 10.3, 4.7 Hz, 1H).

^{13}C NMR (101 MHz, CDCl$_3$): δ (ppm) = 149.5, 149.4, 148.6, 148.3, 138.3, 137.9, 126.8, 126.8, 125.2, 125.0, 113.2, 110.4, 110.4, 109.1, 74.6, 74.3, 41.1, 41.0, 30.6, 30.5, 27.4, 24.2, 24.0, 20.9.

MS (EI, 70 eV): m/z (%) = 268 (55), 254 (31), 171 (65), 115 (41), 91 (100).

HRMS (EI): for $C_{14}H_{16}Br_2OS$: calc. [M-2H$^+$]: 387.9132; found: 387.9126.

2,3-dibromo-5-(naphthalen-2-yl)thiophene (10q)

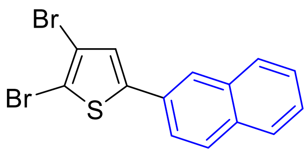

According to **TP 1**, to a mixture of 2,3-dibromothiophene (**6k**, 57 µL, 0.50 mmol, 1.0 equiv) in toluene (1 mL) was added MBDA (0.40 mmol, 0.8 equiv) at 40 °C. After 0.5 h, the resulting diarylmagnesium was transmetalated with a $ZnCl_2$ solution (0.70 mL, 1.00 M in THF, 1.4 equiv) at 0 °C for 30 min. A dry and argon-flushed Schlenk-tube, equipped with a magnetic stirring bar and a septum was charged with $Pd(dba)_2$ (8 mg, 3 mol%), tfp (7 mg, 6 mol%) and 2-iodonaphthalene (105 mg, 0.42 mmol, 0.83 equiv).The freshly prepared arylzinc reagent was added and the reaction mixture was placed in an oil bath at 55 °C for 12 h. Purification of the crude product by flash column chromatography (silica gel, pentane) afforded the title compound as a white solid (**10q**, 127 mg, 0.35 mmol, 83% yield).

^{1}H NMR (400 MHz, $CDCl_3$): δ (ppm) = 8.23–8.13 (m, 1H), 7.96–7.86 (m, 2H), 7.60–7.46 (m, 4H), 7.07 (s, 1H).

^{13}C NMR (101 MHz, $CDCl_3$): δ (ppm) = 143.4, 133.9, 131.5, 130.6, 129.9, 129.6, 128.6, 128.3, 127.1, 126.5, 125.3, 125.2, 114.0, 111.0.

MS (EI, 70 eV): m/z (%) = 209 (13), 208 (100), 164 (65), 163 (88), 104 (11).

HRMS (EI): for $C_{14}H_8Br_2S$: calc. [M^+]: 365.8713; found: 365.8705.

((4-(2,3-dihydrothieno[3,4-b][1,4]dioxin-5-yl)phenyl)ethynyl)trimethylsilane (10r)

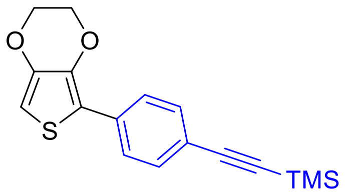

According to **TP 1**, to a mixture of 2,3-dihydrothieno[3,4-b][1,4]dioxine (**6l**, 53 µL, 0.50 mmol, 1.0 equiv) in toluene (1 mL) was added MBDA (0.40 mmol, 0.8 equiv) at 50 °C. After 0.5 h, the resulting diarylmagnesium was transmetalated with a $ZnCl_2$ solution (0.70 mL, 1.00 M in THF, 1.4 equiv) at 0 °C for 30 min. A dry and argon-flushed Schlenk-tube, equipped with a magnetic stirring bar and a septum was charged with $Pd(dba)_2$ (8 mg, 3 mol%), tfp (7 mg, 6 mol%) and ((4-iodophenyl)ethynyl)trimethylsilane (130 mg, 0.42 mmol, 0.83 equiv).The freshly prepared arylzinc reagent was added and the reaction mixture was placed in an oil bath at 55 °C for 12 h. Purification of the crude product by flash column chromatography (silica gel, ihexane/EtOAc = 9:1) afforded the title compound as a yellow solid (**10r**, 111 mg, 0.37 mmol, 89% yield).

^{1}H NMR (400 MHz, $CDCl_3$): δ (ppm) = 7.71–7.63 (m, 2H), 7.48–7.41 (m, 2H), 6.33 (s, 1H), 4.34–4.31 (m, 2H), 4.27–4.22 (m, 2H), 0.26 (s, 9H).
^{13}C NMR (101 MHz, $CDCl_3$): δ (ppm) = 142.4, 138.9, 133.5, 132.3, 125.7, 125.5, 120.9, 117.0, 105.4, 98.5, 94.7, 64.9, 64.5.
MS (EI, 70 eV): m/z (%) = 317 (17), 314 (81), 299 (100), 217 (12).
HRMS (EI): for $C_{17}H_{18}O_2SSi$: calc. [M^+]: 314.0797; found: 314.0790.

3-fluoro-4-(thieno[3,2-b]thiophen-2-yl)pyridine (10s)

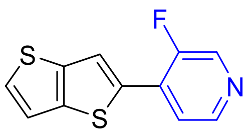

According to **TP 1**, to a mixture of thieno[3,2-b]thiophene (**6m**, 70 mg, 0.50 mmol, 1.0 equiv) in toluene (1 mL) was added MBDA (0.55 mmol, 1.1 equiv) at 70 °C. After 15 min, the resulting diarylmagnesium was transmetalated with a $ZnCl_2$ solution (0.70 mL, 1.00 M in THF, 1.4 equiv) at 0 °C for 30 min. A dry and argon-flushed Schlenk-tube, equipped with a magnetic stirring bar and a septum was charged with $Pd(dba)_2$ (8 mg, 3 mol%), tfp (7 mg, 6 mol%) and 3-fluoro-4-iodopyridine (93 mg, 0.42 mmol, 0.83 equiv).The freshly prepared arylzinc reagent was added and the reaction mixture was placed in an oil bath at 55 °C for 12 h. Purification of the crude product by flash column chromatography (silica gel, *i*hexane/EtOAc = 4:1) afforded the title compound as a brown solid (**10s**, 58 mg, 0.25 mmol, 60% yield).

^{1}H NMR (400 MHz, $CDCl_3$): δ (ppm) = 8.53 (d, *J* = 3.2 Hz, 1H), 8.41 (dd, *J* = 5.1, 1.0 Hz, 1H), 7.89 (t, *J* = 0.8 Hz, 1H), 7.57–7.48 (m, 2H), 7.30 (d, *J* = 5.3 Hz, 1H).
^{13}C NMR (101 MHz, $CDCl_3$): δ (ppm) = 155.5 (d, *J* = 259.3 Hz), 146.2 (d, *J* = 5.0 Hz), 140.8 (d, *J* = 3.7 Hz), 140.3 (d, *J* = 1.7 Hz), 139.3 (d, *J* = 25.4 Hz), 135.9 (d, *J* = 4.0 Hz), 129.7, 129.6, 121.8 (d, *J* = 9.6 Hz), 121.3, 119.6.
^{19}F NMR (376 MHz, $CDCl_3$): δ (ppm) = -128.9.
MS (EI, 70 eV): m/z (%) = 236 (10), 235 (16), 234 (100), 63 (15), 61 (10), 41 (63).
HRMS (EI): for $C_{11}H_6FNS_2$: calc. [M^+]: 234.9926; found: 234.9914.

7. Postfunctionalizations

5,6-difluoro-4-iodo-3-phenyl-1H-isochromen-1-one (11a)

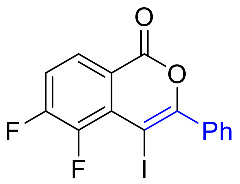

tert-butyl 3,4-difluoro-2-(phenylethynyl)benzoate (**9s**, 31 mg, 0.10 mmol, 1.0 equiv) was dissolved in dry DCM (1 mL). The reaction mixture was cooled to 0 C° and iodine monochloride (7 µL, 0.12 mmol, 1.2 equiv)) was added. The reaction mixture was allowed to warm to room temperature and stirred for 0.5 h. The reaction was quenched by addition of sat. $Na_2S_2O_3$ solution (5 mL) and extracted with EtOAc (3 x 10 mL). Purification of the crude product by flash column chromatography (silica gel, ihexane/EtOAc = 96:4) afforded the title compound as a white solid (**11a**, 36 mg, 0.094 mmol, 94% yield).

^{1}H NMR (400 MHz, $CDCl_3$): δ (ppm) = 8.20 (ddd, *J* = 8.9, 5.2, 1.9 Hz, 1H), 7.84–7.60 (m, 2H), 7.52–7.47 (m, 3H), 7.42 (td, *J* = 8.9, 6.6 Hz, 1H).

^{13}C NMR (101 MHz, $CDCl_3$): δ (ppm) = 159.9, 157.2, 155.4 (dd, *J* = 258.5, 13.8 Hz), 145.4 (dd, *J* = 261.2, 14.8 Hz), 135.5, 130.8, 130.7, 130.1, 129.7, 128.7, 128.4, 126.9 (dd, *J* = 8.8, 5.1 Hz), 118.4 (d, *J* = 19.1 Hz), 118.2–117.8 (m), 58.9 (t, *J* = 4.1 Hz).

^{19}F NMR (376 MHz, $CDCl_3$): δ (ppm) = -120.6, -138.6.

MS (EI, 70 eV): m/z (%) = 384 (14), 383 (100), 355 (30), 229 (22), 201 (39), 105 (49), 77 (31).

HRMS (EI): for $C_{15}H_7F_2IO_2$: calc. [M^+]: 383.9459; found: 383.9455.

5-fluoro-3'-((5-(4-fluorophenyl)thiophen-2-yl)methyl)-4'-methyl-[1,1'-biphenyl]-2-carbonitrile (11b)

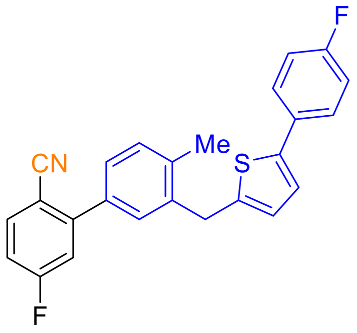

A dry and argon flushed Schlenk-tube, equipped with a magnetic stirring bar and a septum, was charged with 2-(5-fluoro-3'-((5-(4-fluorophenyl)thiophen-2-yl)methyl)-4'-methyl-[1,1'-biphenyl]-2-yl)-4,4-dimethyl-4,5-dihydrooxazole (**9z**, 47 mg, 0.10 mmol). Oxalyl chloride (0.50 mL) was added and the resulting solution was cooled to 0 °C. DMF (1 drop) was added and the reaction mixture was stirred at 50 °C for 4 h. The mixture was cooled to 0 °C and quenched with a sat. aqueous $NaHCO_3$ solution and extracted with ethyl acetate (3 x 10 mL). The combined organic extracts were dried over $MgSO_4$, filtered and concentrated. Purification of the crude product by flash column chromatography (silica gel, ihexane/EtOAc = 9:1) afforded the title compound as a white solid (**11b**, 38 mg, 0.095 mmol, 95% yield).

^{1}H NMR (400 MHz, $CDCl_3$): δ (ppm) = 7.62 (dd, *J* = 8.6, 5.5 Hz, 1H), 7.38–7.34 (m, 2H), 7.29–7.24 (m, 2H), 7.19 (d, *J* = 7.7 Hz, 1H), 7.08 (dd, *J* = 9.2, 2.6 Hz, 1H), 6.99 (ddd, *J* = 8.7, 7.8, 2.6 Hz, 1H), 6.93 (d, *J* = 3.6 Hz, 1H), 6.92–6.86 (m, 2H), 6.60 (dt, *J* = 3.7, 1.1 Hz, 1H), 4.07 (s, 2H), 2.27 (s, 3H).
^{13}C NMR (101 MHz, $CDCl_3$): δ (ppm) = 164.9 (d, *J* = 256.6 Hz), 162.2 (d, *J* = 246.7 Hz), 148.5 (d, *J* = 9.2 Hz), 142.6 (d, *J* = 1.0 Hz), 141.8, 139.0, 138.0, 136.2 (d, *J* = 9.7 Hz), 135.2 (d, *J* = 1.7 Hz), 131.2, 130.9 (d, *J* = 3.3 Hz), 129.7, 127.3 (d, *J* = 8.0 Hz), 127.2, 126.5, 122.9 (d, *J* = 1.2 Hz), 118.3, 117.4 (d, *J* = 22.7 Hz), 115.9 (d, *J* = 21.8 Hz), 115.1 (d, *J* = 22.7 Hz), 107.5 (d, *J* = 3.2 Hz), 34.2, 19.5.
^{19}F NMR (376 MHz, $CDCl_3$): δ (ppm) = -103.1, -115.1.
MS (EI, 70 eV): m/z (%) = 401 (58), 223 (100), 191 (54), 178 (21), 133 (28).
HRMS (EI): for $C_{25}H_{17}F_2NS$: calc. [M+]: 401.1050; found: 401.1047.

1-(2,6-difluoro-3'-methyl-[1,1'-biphenyl]-3-yl)-4-(trimethylsilyl)-1H-1,2,3-triazole (11d)

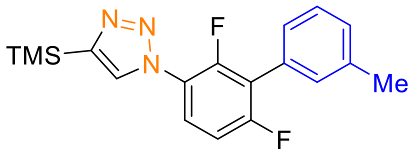

(E)-1-((2,6-difluoro-3'-methyl-[1,1'-biphenyl]-3-yl)diazenyl)pyrrolidine (**9aa**, 150 mg, 0.50 mmol, 1.0 equiv) and NaN_3 (65 mg, 1.0 mmol, 2.0 equiv) were suspended in DCM (1 mL). $BF_3{\cdot}OEt_2$ (0.12 mL, 1.0 mmol, 2.0 equiv) and CF_3COOH (80 µL, 1.0 mmol, 2.0 equiv) were added and the reaction mixture was stirred for 0.5 h at 25 °C. The reaction mixture was quenched by addition of water and was extracted with DCM (3 x 10 mL), dried over $MgSO_4$ and the solvent was removed in vacuo. The crude product was purified through a short silica plug and the obtained azide **11c** was directly used for the next step.

The crude azide **11c** was dissolved in MeCN (1 mL) and DIPEA (43 µL, 0.25 mmol, 0.5 equiv), ethynyltrimethylsilane (0.21 mL, 1.5 mmol, 3.0 equiv) followed by CuI (19 mg, 10 mol%) were added. The reaction mixture was stirred at 25 °C for 24 h, quenched by addition of water and extracted with DCM (3 x 10 mL). The combined organic extracts were dried over $MgSO_4$, filtered and concentrated. Purification of the crude product by flash column chromatography (silica gel, ihexane/EtOAc = 9:1) afforded the title compound as a pale yellow solid (**11d**, 163 mg, 0.48 mmol, 95% yield).

^{1}H NMR (400 MHz, $CDCl_3$): δ (ppm) = 7.98 (d, *J* = 3.1 Hz, 1H), 7.82 (td, *J* = 8.7, 5.5 Hz, 1H), 7.34 (t, *J* = 7.6 Hz, 1H), 7.28–7.21 (m, 3H), 7.11 (td, *J* = 8.8, 1.7 Hz, 1H), 2.38 (d, *J* = 1.3 Hz, 3H), 0.35 (s, 9H).

^{13}C NMR (101 MHz, $CDCl_3$): δ (ppm) = 159.5 (dd, *J* = 251.5, 6.2 Hz), 151.3 (dd, *J* = 252.0, 7.4 Hz), 147.1, 138.3, 130.8 (t, *J* = 1.7 Hz), 130.2 (d, *J* = 7.2 Hz), 129.7, 128.5, 127.8, 127.3 (t, *J* = 1.8 Hz), 124.6 (dd, *J* = 10.2, 1.5 Hz), 122.4 (dd, *J* = 12.2, 3.9 Hz), 120.1 (dd, *J* = 20.5, 18.1 Hz), 112.5 (dd, *J* = 24.5, 3.9 Hz), 21.5, -1.1.

^{19}F NMR (376 MHz, $CDCl_3$): δ (ppm) = -111.9, -123.0.

MS (EI, 70 eV): m/z (%) = 315 (56), 300 (99), 184 (100), 77 (76).

HRMS (EI): for C_{18}H19F_2N_3Si: calc. [M+]: 343.1310; found: 343.1300.

2,6-difluoro-3'-methyl-[1,1'-biphenyl]-3-amine (11e)

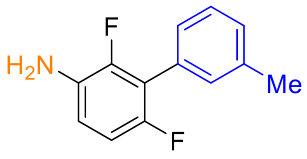

(E)-1-((2,6-difluoro-3'-methyl-[1,1'-biphenyl]-3-yl)diazenyl)pyrrolidine (**9aa**, 60 mg, 0.20 mmol, 1.0 equiv) and NaN_3 (26 mg, 0.4 mmol, 2.0 equiv) were suspended in DCM (1 mL). $BF_3{\cdot}OEt_2$ (50 µL, 0.4 mmol, 2.0 equiv) and CF_3COOH (30 µL, 0.4 mmol, 2.0 equiv) were added and the reaction mixture was stirred for 0.5 h at 25 °C. The reaction mixture was quenched by addition of water and was extracted with DCM (3 x 10 mL), dried over $MgSO_4$ and the solvent was removed in vacuo. The crude product was purified through a short silica plug and the obtained azide **11c** was directly used for the next step.

The crude azide **11c** was dissolved in EtOAc (2 mL) and EtOH (1 mL) followed by addition of $SnCl_2{\cdot}2H_2O$ (225 mg, 1.0 mmol, 5.0 equiv) and the reaction mixture was stirred at room temperature for 5 min. The reaction was quenched by addition of water (2 mL) and the aqueous phase was extracted with EtOAc (3 x 10 mL). The combined organic extracts were dried over $MgSO_4$, filtered and concentrated. Purification of the crude product by flash column chromatography (silica gel, ihexane/EtOAc = 4:1) afforded the title compound as a colorless oil (**11e**, 42 mg, 0.19 mmol, 95% yield).

^{1}H NMR (400 MHz, $CDCl_3$): δ (ppm) = 7.36 (t, *J* = 7.5 Hz, 1H), 7.31–7.25 (m, 2H), 7.22 (ddt, *J* = 7.7, 1.9, 0.9 Hz, 1H), 6.81 (td, *J* = 9.0, 1.7 Hz, 1H), 6.71 (td, *J* = 9.2, 5.4 Hz, 1H), 3.63 (s, 2H), 2.42 (s, 3H).

^{13}C NMR (101 MHz, $CDCl_3$): δ (ppm) = 152.7 (dd, *J* = 238.8, 5.9 Hz), 148.4 (dd, *J* = 241.1, 6.8 Hz), 137.9, 131.2 (d, *J* = 3.1 Hz), 131.1–131.0 (m), 129.6, 129.0, 128.2, 127.4 (t, *J* = 1.9 Hz), 118.7 (dd, *J* = 20.1, 17.0 Hz), 115.3 (dd, *J* = 9.2, 4.8 Hz), 111.1 (dd, *J* = 23.8, 4.0 Hz), 21.6.

^{19}F NMR (376 MHz, $CDCl_3$): δ (ppm) = -128.3, -135.3.

MS (EI, 70 eV): m/z (%) = 220 (14), 219 (100), 198 (37), 170 (10).

HRMS (EI): for $C_{13}H_{11}F_2N$: calc. [M^+]: 219.0860; found: 219.0854.

8. X-Ray crystal structure data

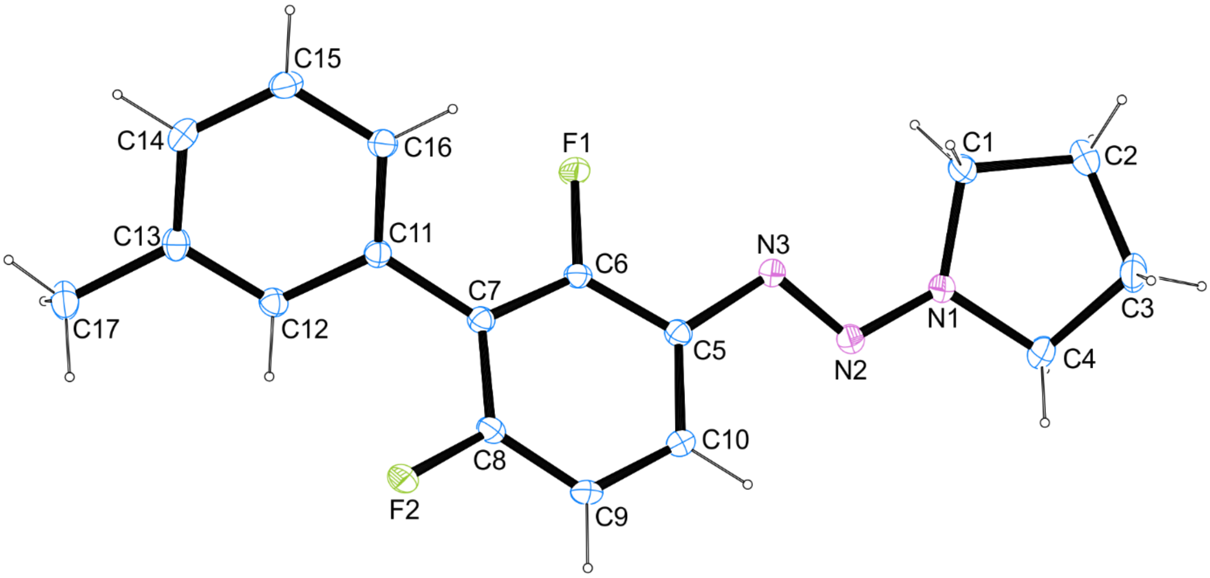

Figure 1: X-ray crystal structure of **9aa** (CCDC 2156072).

Experimental details

The X-ray intensity data of *CCDC 2156072* were measured on a Bruker D8 Venture TXS system equipped with a multilayer mirror monochromator and a Mo Kα rotating anode X-ray tube (λ = 0.71073 Å). The frames were integrated with the Bruker SAINT software package [1]. Data were corrected for absorption effects using the Multi-Scan method (SADABS) [2]. The structure was solved and refined using the Bruker SHELXTL Software Package [3]. All hydrogen atoms have been calculated in ideal geometry riding on their parent atoms. The figures have been drawn at the 25% ellipsoid probability level [4].

References

[1] Bruker (2012). SAINT. Bruker AXS Inc., Madison, Wisconsin, USA.

[2] Sheldrick, G. M. (1996). SADABS. University of Göttingen, Germany.

[3] Sheldrick, G. M. (2015). Acta Cryst. A71, 3-8.

[4] Farrugia, L. J. (2012). J. Appl. Cryst. 45, 849-854.

Table 1: Crystallographic data of **9aa** (CCDC 2156072).

	**9aa**
net formula	$C_{17}H_{17}F_2N_3$
M_r/g mol^{-1}	301.33
crystal size/mm	0.160 × 0.120 × 0.080
T/K	173.(2)
radiation	MoKα
diffractometer	'Bruker D8 Venture TXS'
crystal system	monoclinic
space group	'P 1 21/c 1'
a/Å	6.8655(2)
b/Å	7.5245(2)
c/Å	28.4353(9)
α/°	90
β/°	91.5810(10)
γ/°	90
V/Å^3	1468.39(7)
Z	4
calc. density/g cm^{-3}	1.363
μ/mm^{-1}	0.100
absorption correction	Multi-Scan
transmission factor range	0.96–0.99
refls. measured	25010
R_{int}	0.0330
mean σ(I)/I	0.0232
θ range	2.866–27.483
observed refls.	3033
x, y (weighting scheme)	0.0806, 0.5799
hydrogen refinement	constr
Flack parameter	?
refls in refinement	3358
parameters	201
restraints	0
$R(F_{obs})$	0.0610
$R_w(F^2)$	0.1570
S	1.203
shift/error$_{max}$	0.001
max electron density/e Å^{-3}	0.546
min electron density/e Å^{-3}	−0.451

9. NMR Spectra

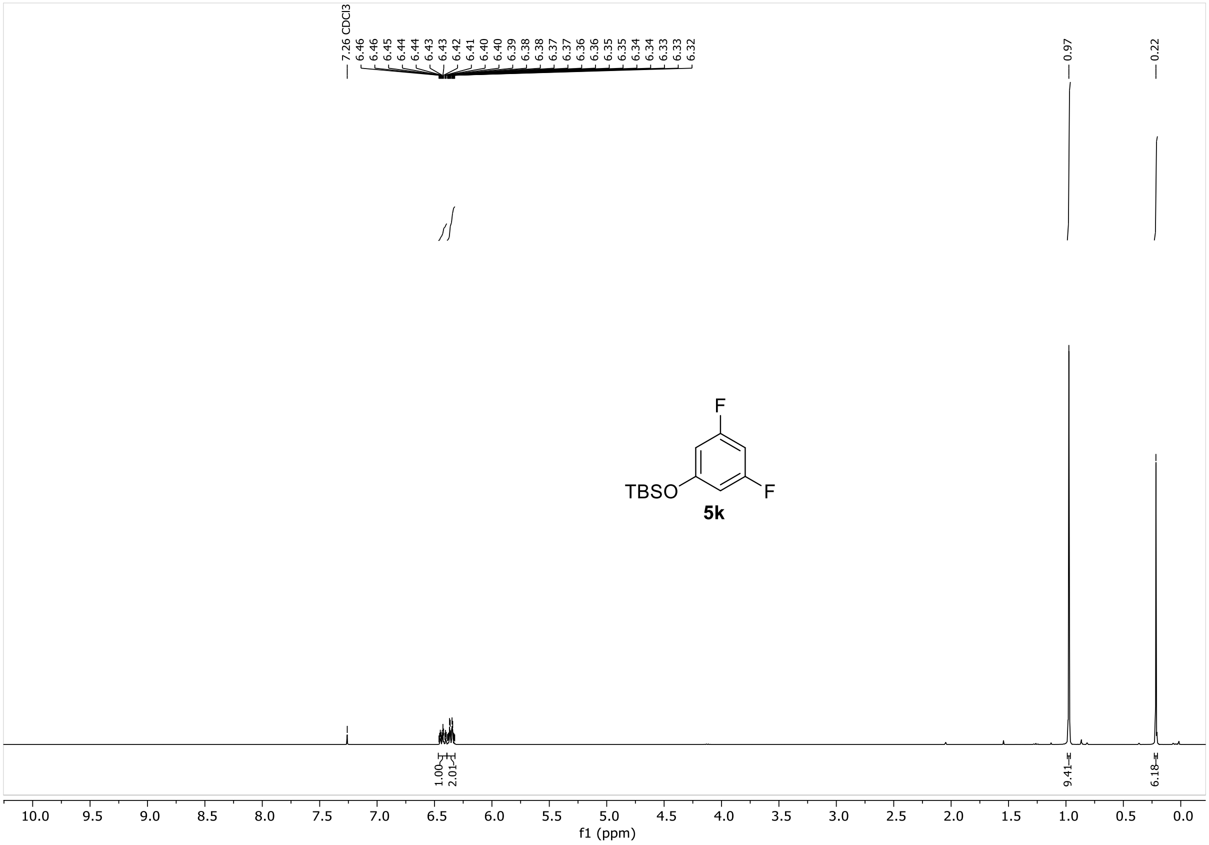

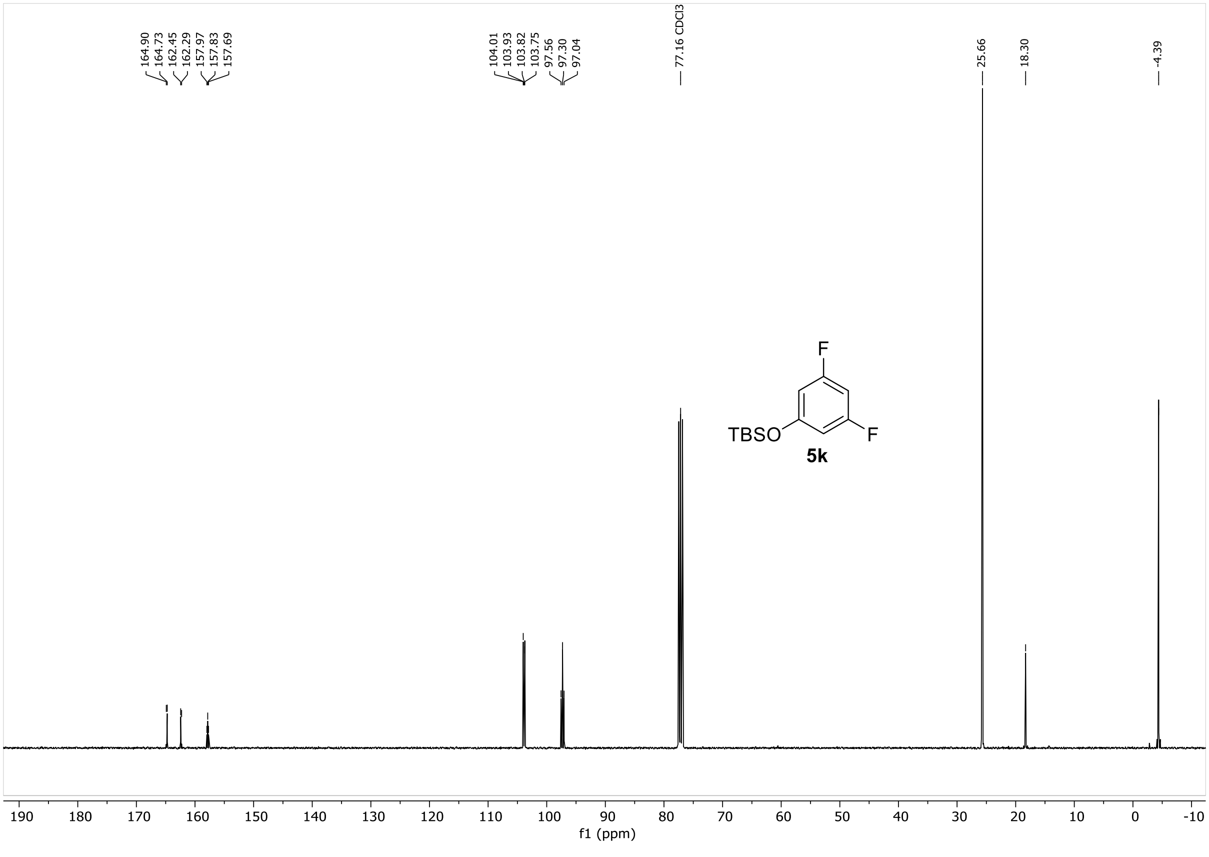

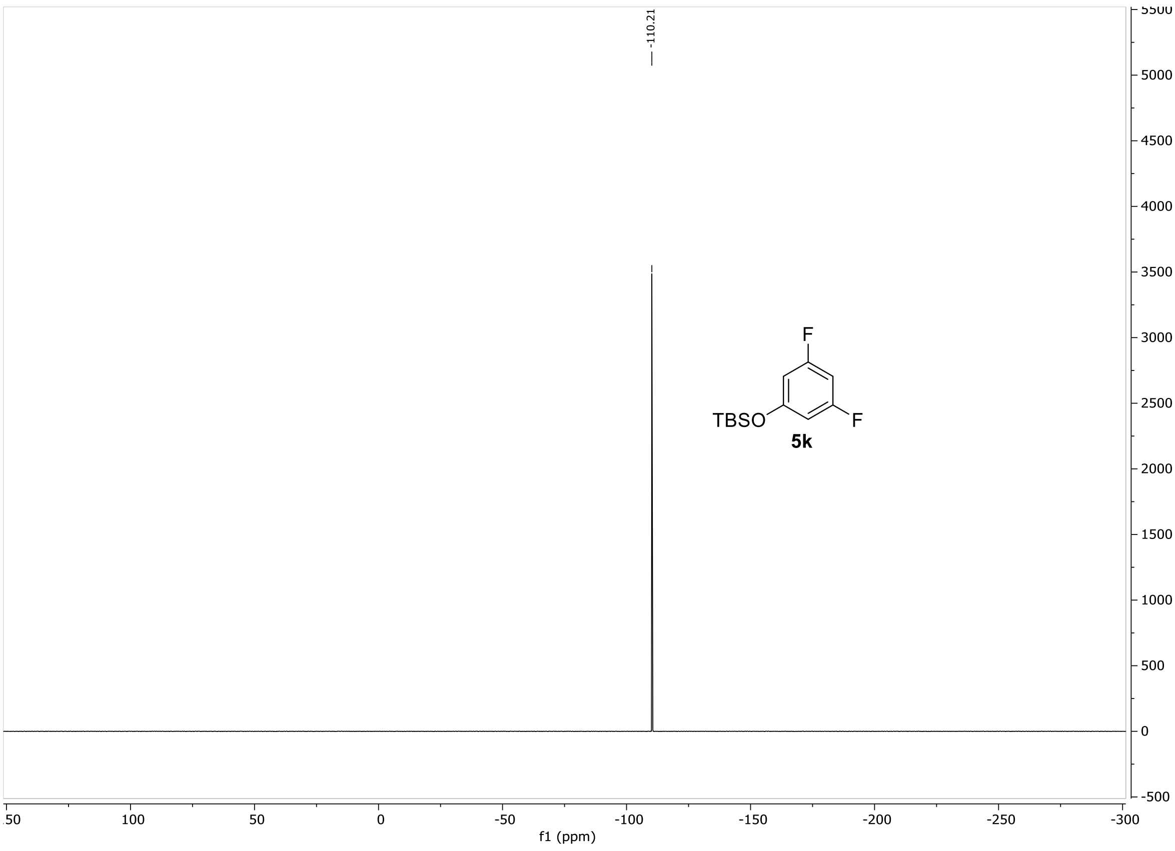
-110.21
TBSO
F
F
5k
f1 (ppm)

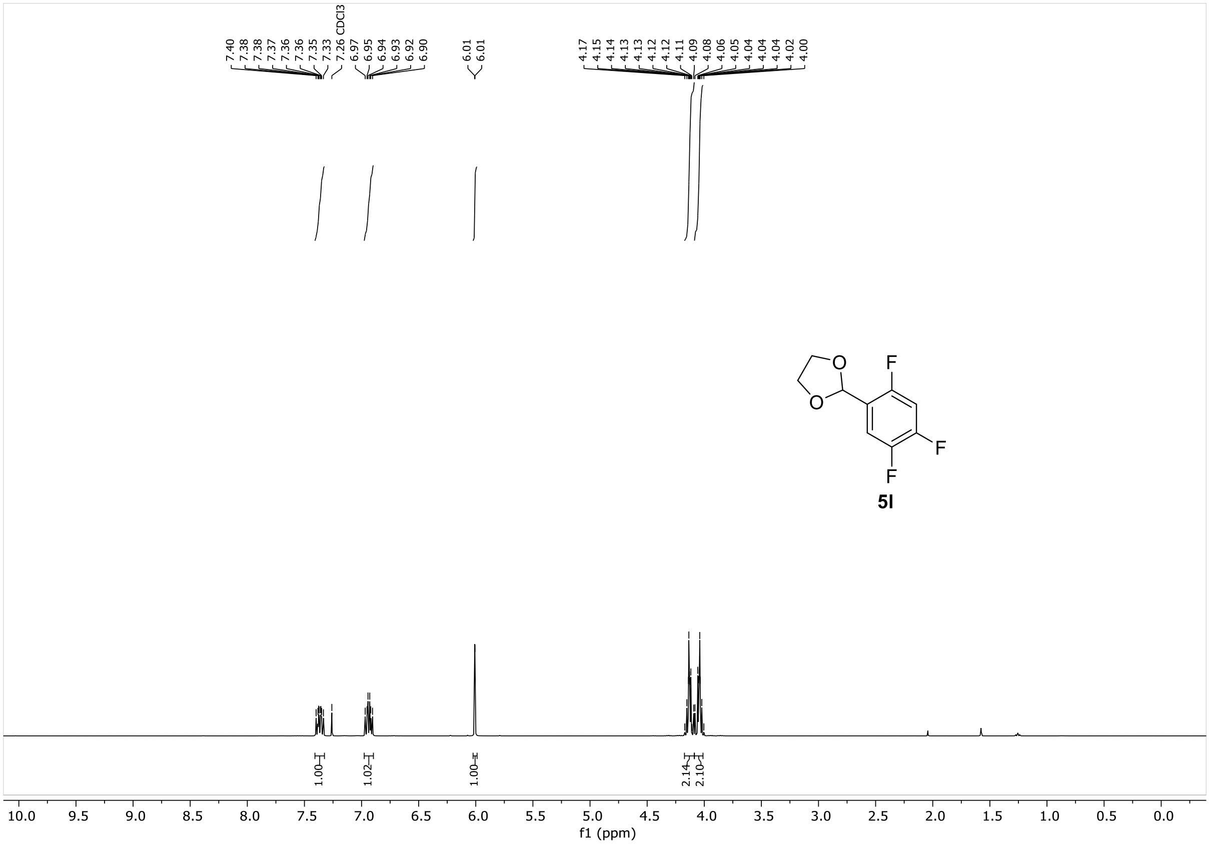
7.40
7.38
7.38
7.37
7.36
7.36
7.35
7.33
7.26 CDCl3
6.97
6.95
6.94
6.93
6.92
6.90
6.01
6.01
4.17
4.15
4.14
4.13
4.13
4.12
4.12
4.11
4.09
4.08
4.06
4.05
4.04
4.04
4.04
4.02
4.00
O
O
F
F
F
5l
1.00
1.02
1.00
2.14
2.10
f1 (ppm)

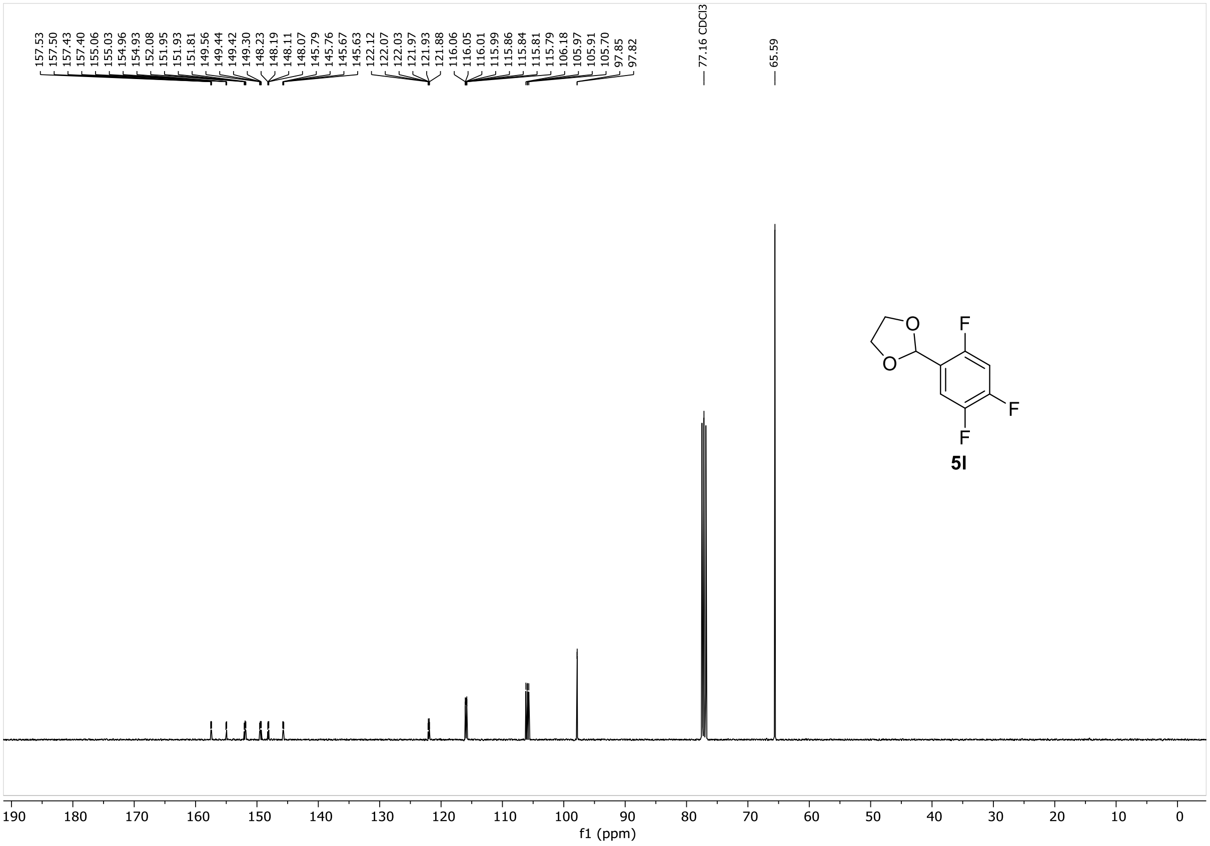
157.53
157.50
157.43
157.40
155.06
155.03
154.96
154.93
152.08
151.95
151.93
151.81
149.56
149.44
149.42
149.30
148.23
148.19
148.11
148.07
145.79
145.76
145.67
145.63
122.12
122.07
122.03
121.97
121.93
121.88
116.06
116.05
116.01
115.99
115.86
115.84
115.81
115.79
106.18
105.97
105.91
105.70
97.85
97.82
77.16 CDCl3
65.59
O
O
F
F
F
5l
190
180
170
160
150
140
130
120
110
100
90
80
70
60
50
40
30
20
10
0
f1 (ppm)

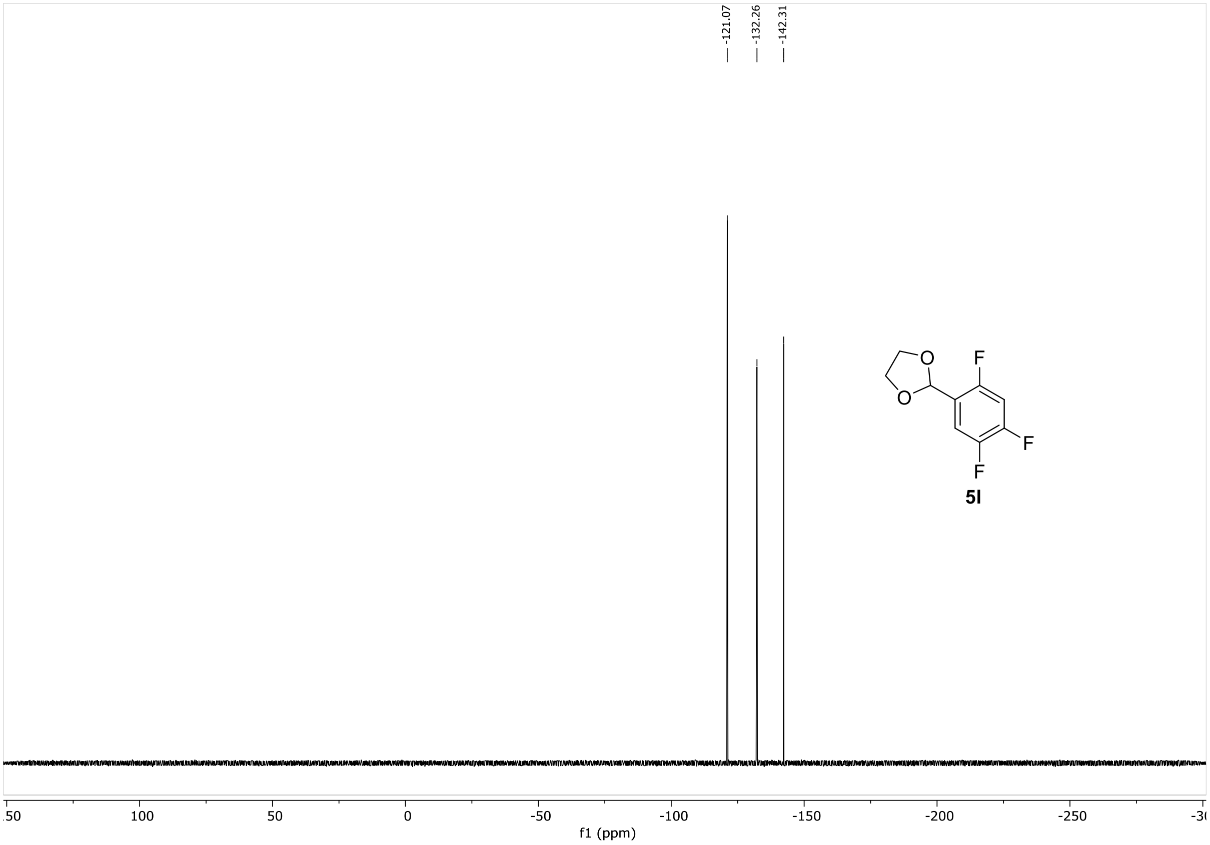
-121.07
-132.26
-142.31
O
O
F
F
F
5l
100
50
0
-50
-100
-150
-200
-250
f1 (ppm)

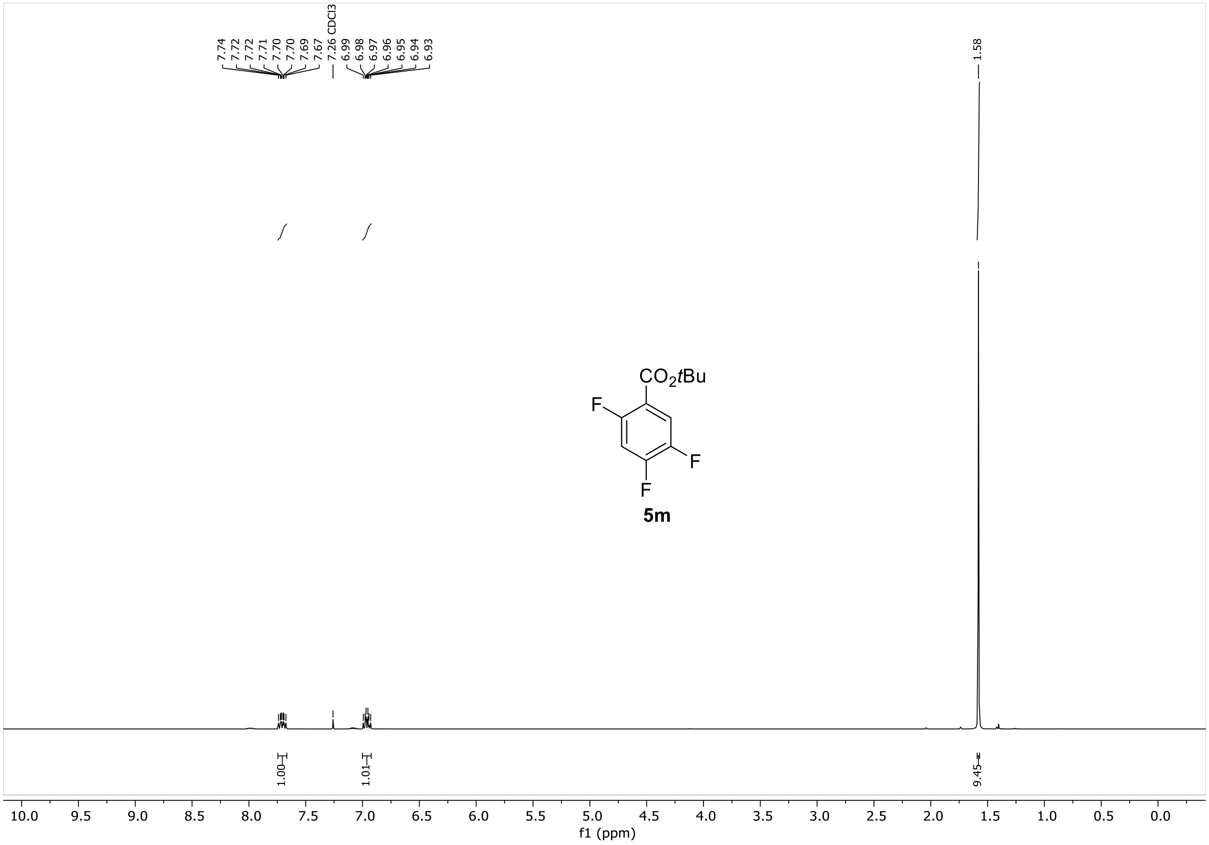

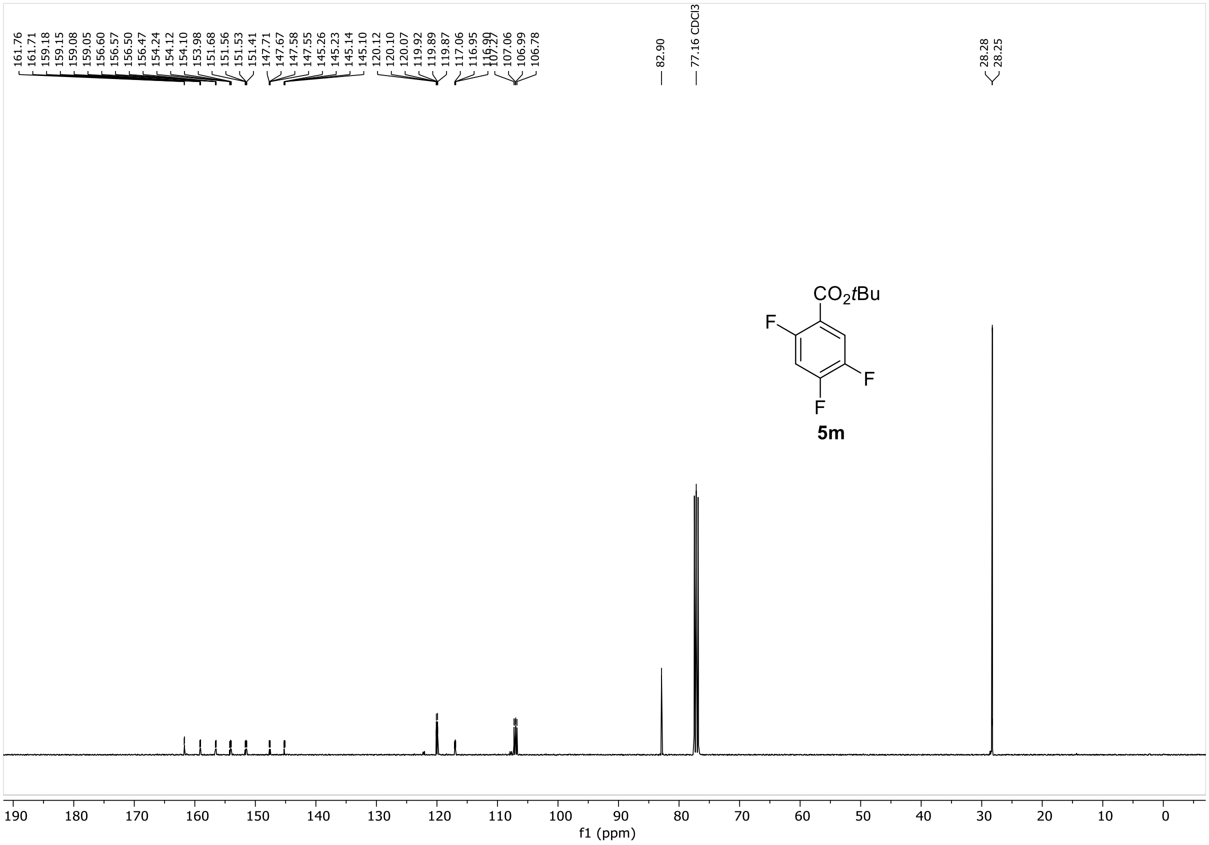

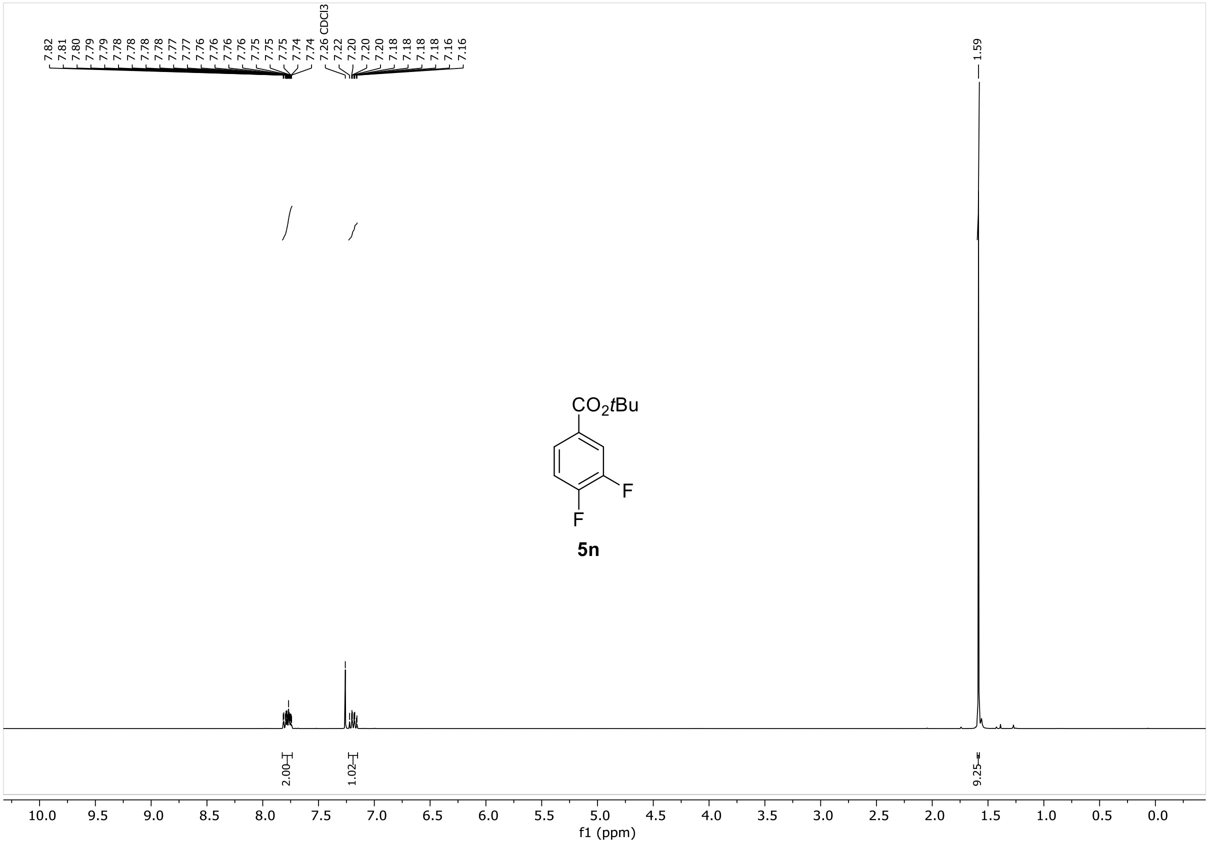
CO2tBu
F
F
5n
f1 (ppm)

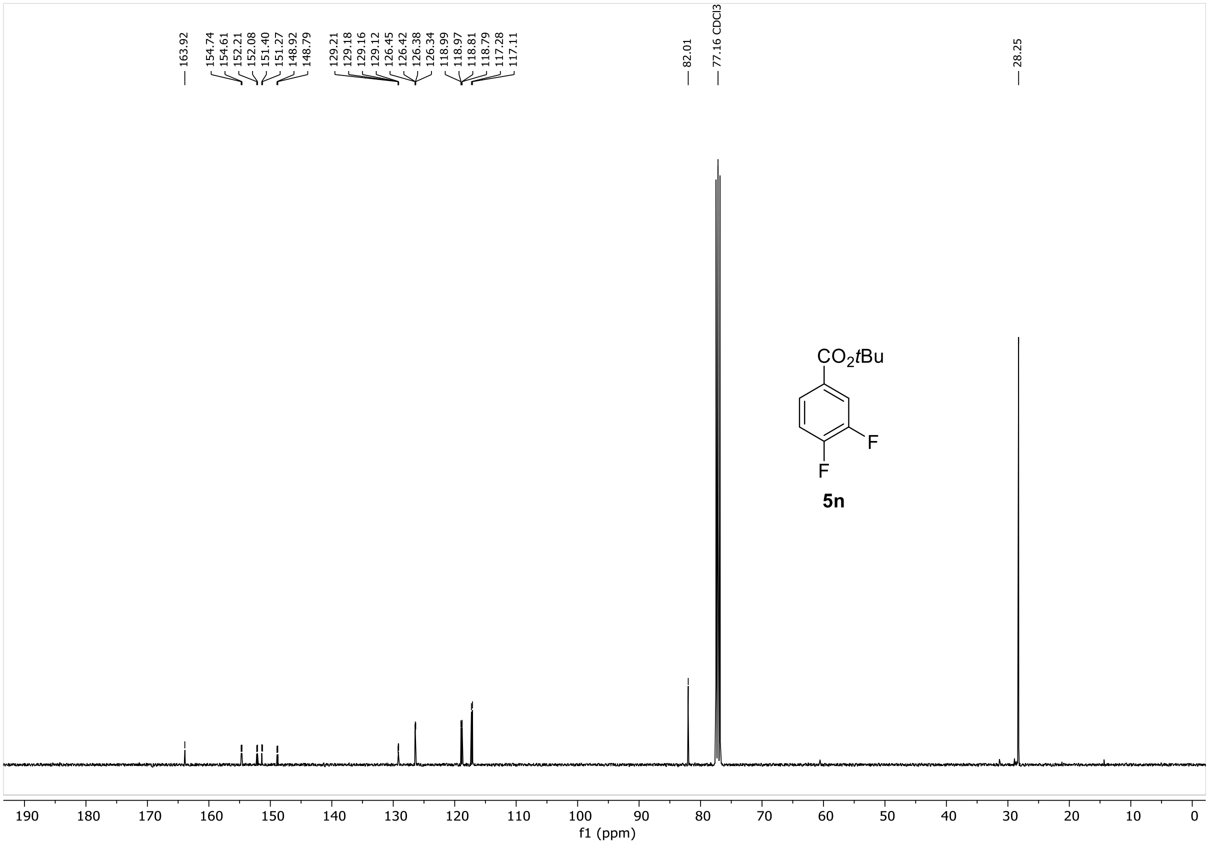
CO2tBu
F
F
5n
f1 (ppm)

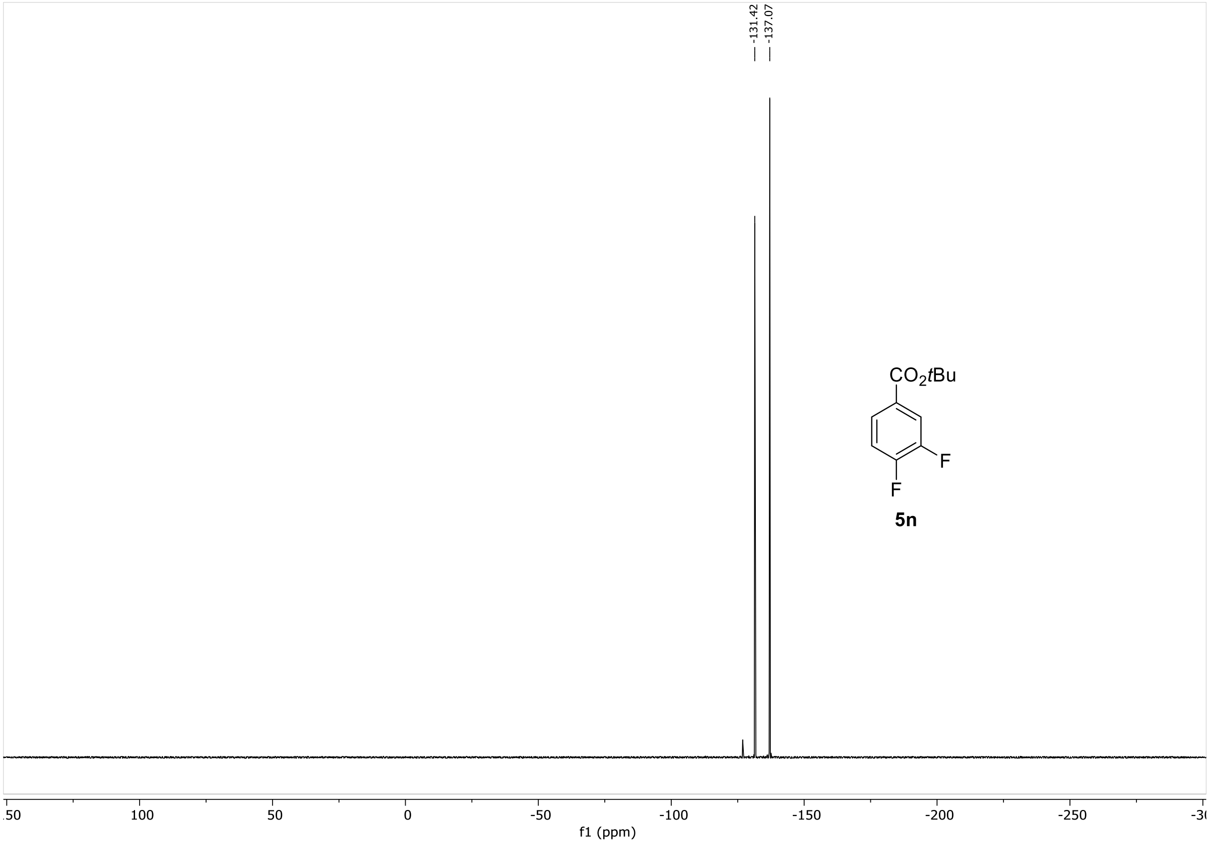
-131.42
-137.07
CO2tBu
F
F
5n
f1 (ppm)

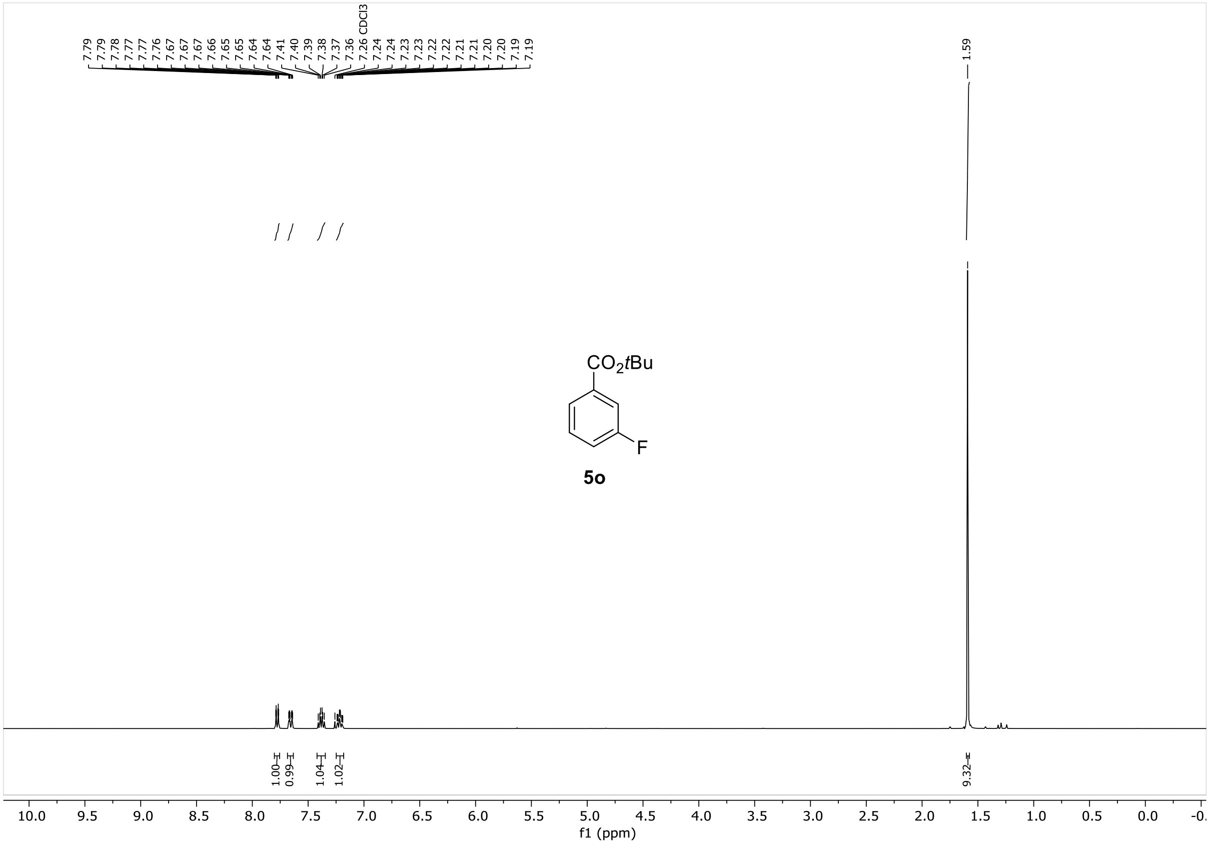
7.79 7.79 7.78 7.77 7.77 7.76 7.67 7.67 7.66 7.65 7.65 7.64 7.64 7.41 7.40 7.39 7.38 7.37 7.36 7.26 CDCl3 7.24 7.24 7.23 7.23 7.22 7.22 7.21 7.21 7.20 7.20 7.19 7.19
1.59
CO2tBu
F
5o
1.00
0.99
1.04
1.02
9.32
f1 (ppm)

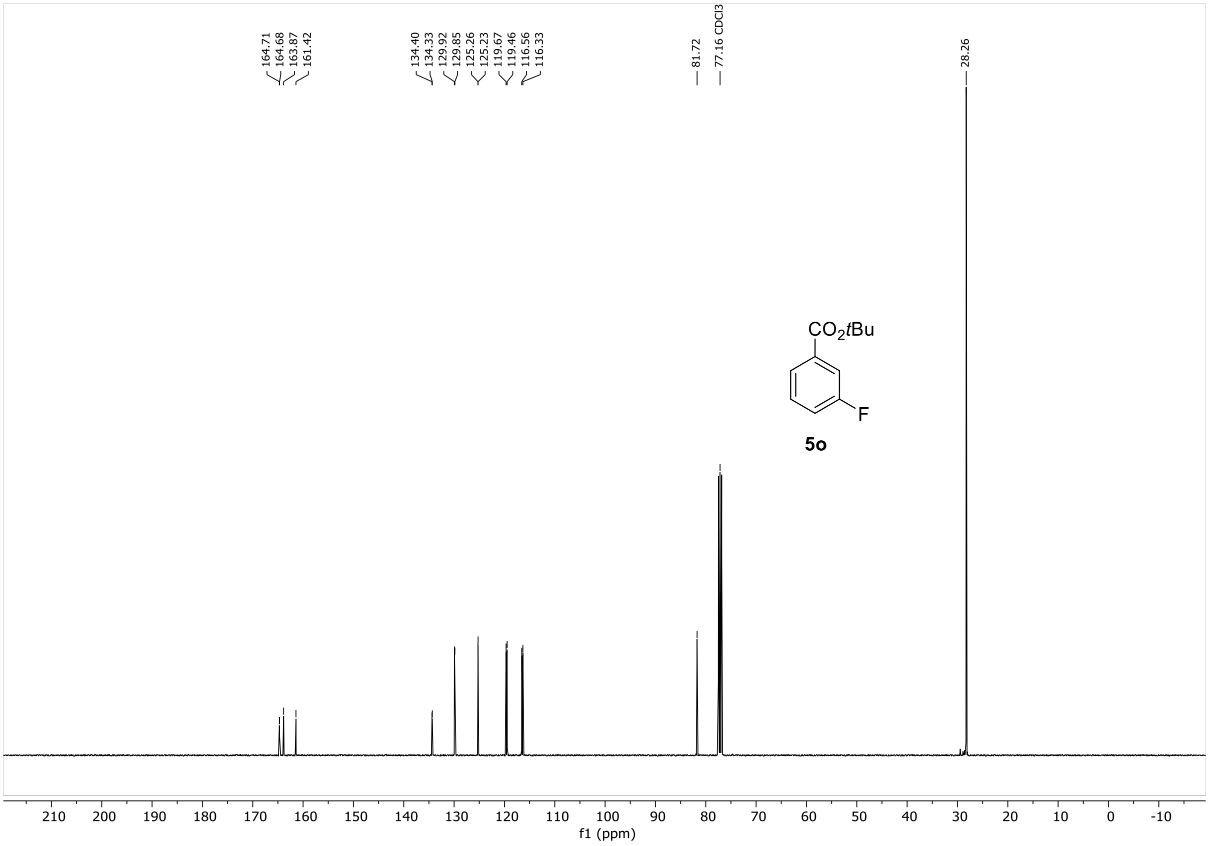
164.71
164.68
163.87
161.42
134.40
134.33
129.92
129.85
125.26
125.23
119.67
119.46
116.56
116.33
81.72
77.16 CDCl3
28.26
CO2tBu
F
5o
f1 (ppm)

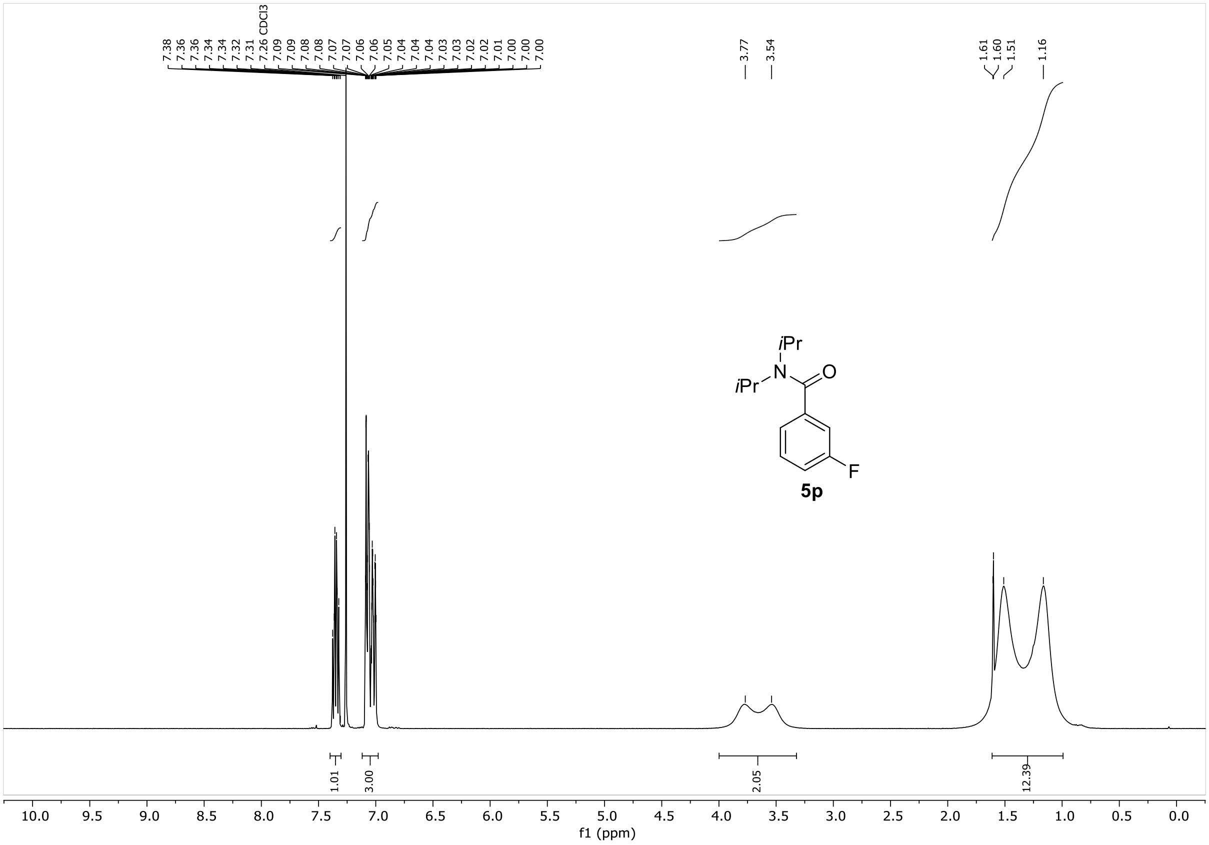
7.38
7.36
7.36
7.34
7.34
7.32
7.31
7.26 CDCl3
7.09
7.09
7.08
7.08
7.07
7.07
7.06
7.06
7.05
7.04
7.04
7.03
7.03
7.02
7.02
7.01
7.00
7.00
7.00
3.77
3.54
1.61
1.60
1.51
1.16
iPr
N
O
iPr
F
5p
1.01
3.00
2.05
12.39
f1 (ppm)

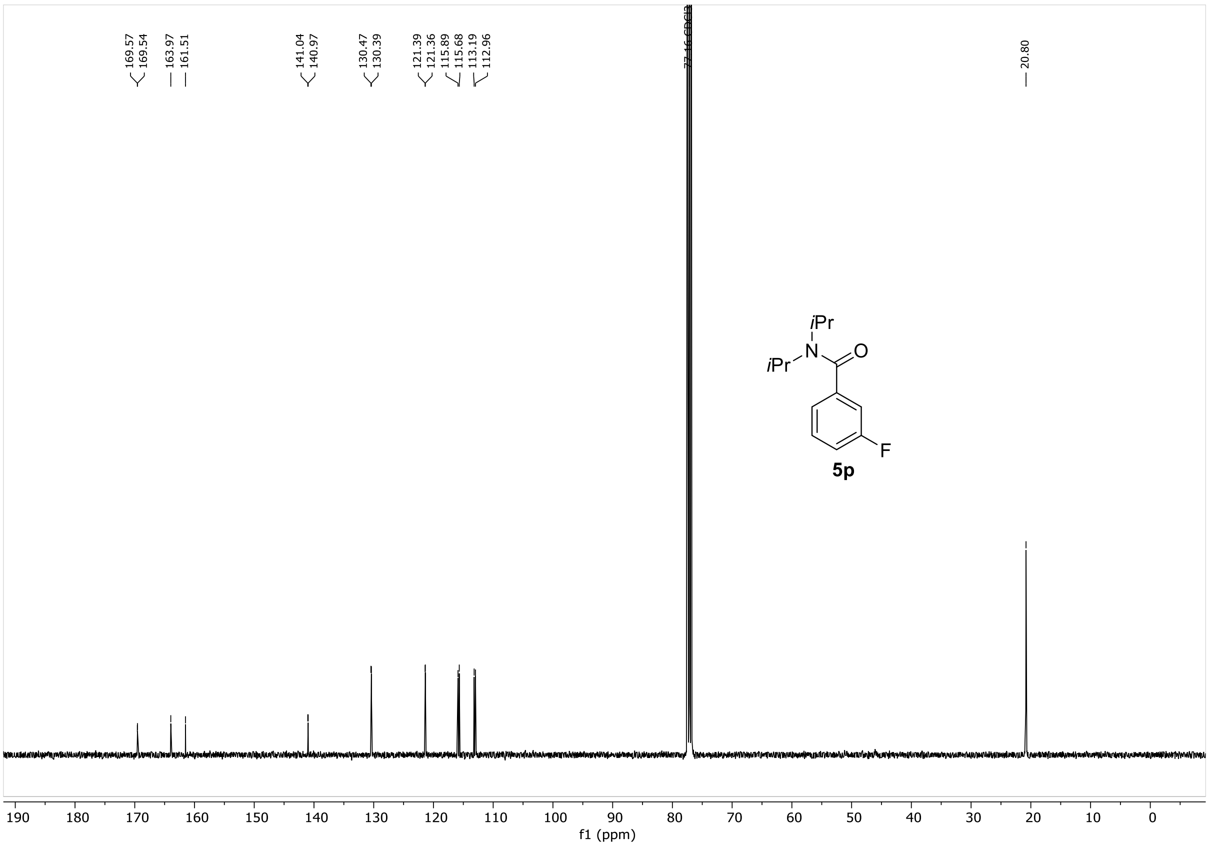
169.57
169.54
163.97
161.51
141.04
140.97
130.47
130.39
121.39
121.36
115.89
115.68
113.19
112.96
77.16 CDCl3
20.80
iPr
N
O
iPr
F
5p
190 180 170 160 150 140 130 120 110 100 90 80 70 60 50 40 30 20 10 0
f1 (ppm)

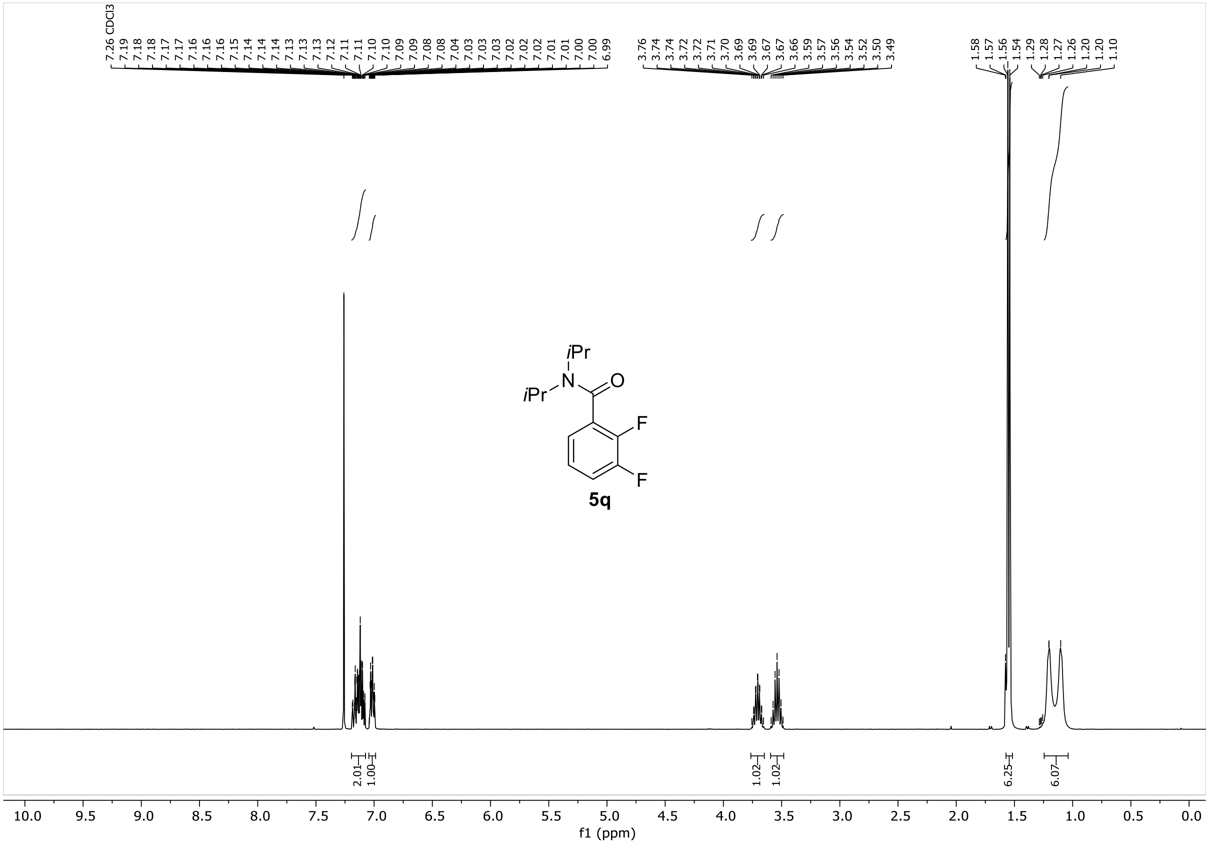
7.26 CDCl3
7.19
7.18
7.18
7.17
7.17
7.16
7.16
7.15
7.14
7.14
7.13
7.13
7.13
7.12
7.11
7.11
7.10
7.10
7.09
7.09
7.08
7.08
7.04
7.03
7.03
7.02
7.02
7.02
7.01
7.01
7.00
7.00
6.99
3.76
3.74
3.74
3.72
3.72
3.71
3.70
3.69
3.69
3.67
3.67
3.66
3.59
3.57
3.56
3.54
3.52
3.50
3.49
1.58
1.57
1.56
1.54
1.29
1.28
1.27
1.26
1.20
1.20
1.10
iPr
N
O
iPr
F
F
5q
2.01
1.00
1.02
1.02
6.25
6.07
10.0 9.5 9.0 8.5 8.0 7.5 7.0 6.5 6.0 5.5 5.0 4.5 4.0 3.5 3.0 2.5 2.0 1.5 1.0 0.5 0.0
f1 (ppm)

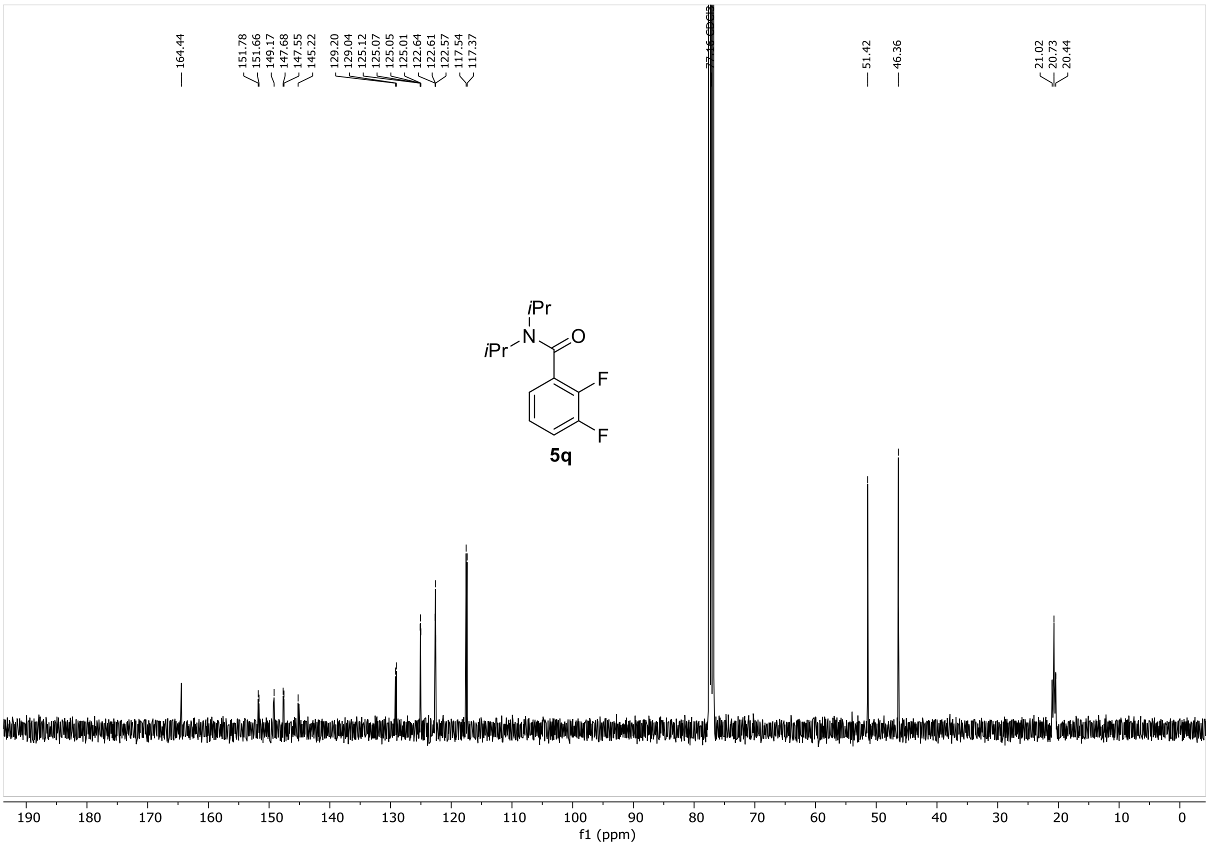
164.44
151.78
151.66
149.17
147.68
147.55
145.22
129.20
129.04
125.12
125.07
125.05
125.01
122.64
122.61
122.57
117.54
117.37
51.42
46.36
21.02
20.73
20.44
iPr
N
O
iPr
F
F
5q
f1 (ppm)

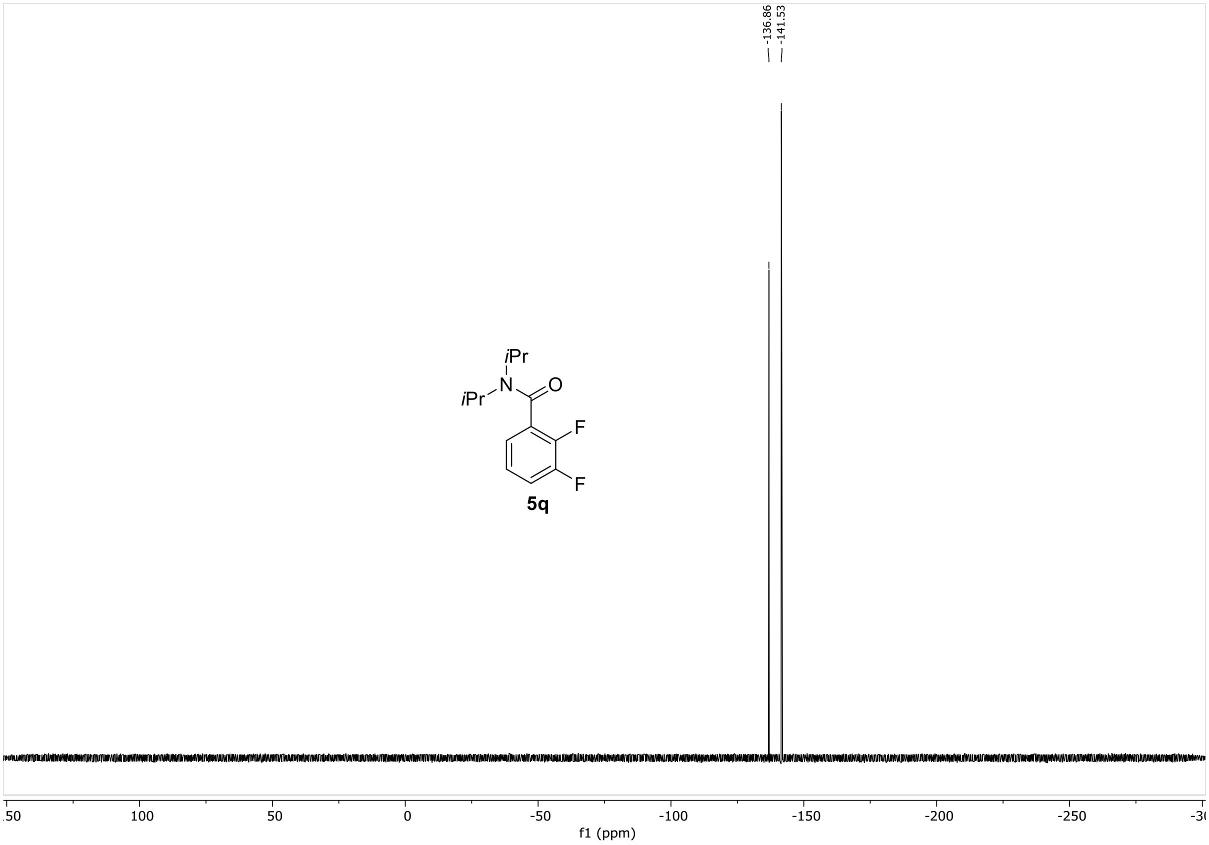
-136.86
-141.53
iPr
N
O
iPr
F
F
5q
f1 (ppm)

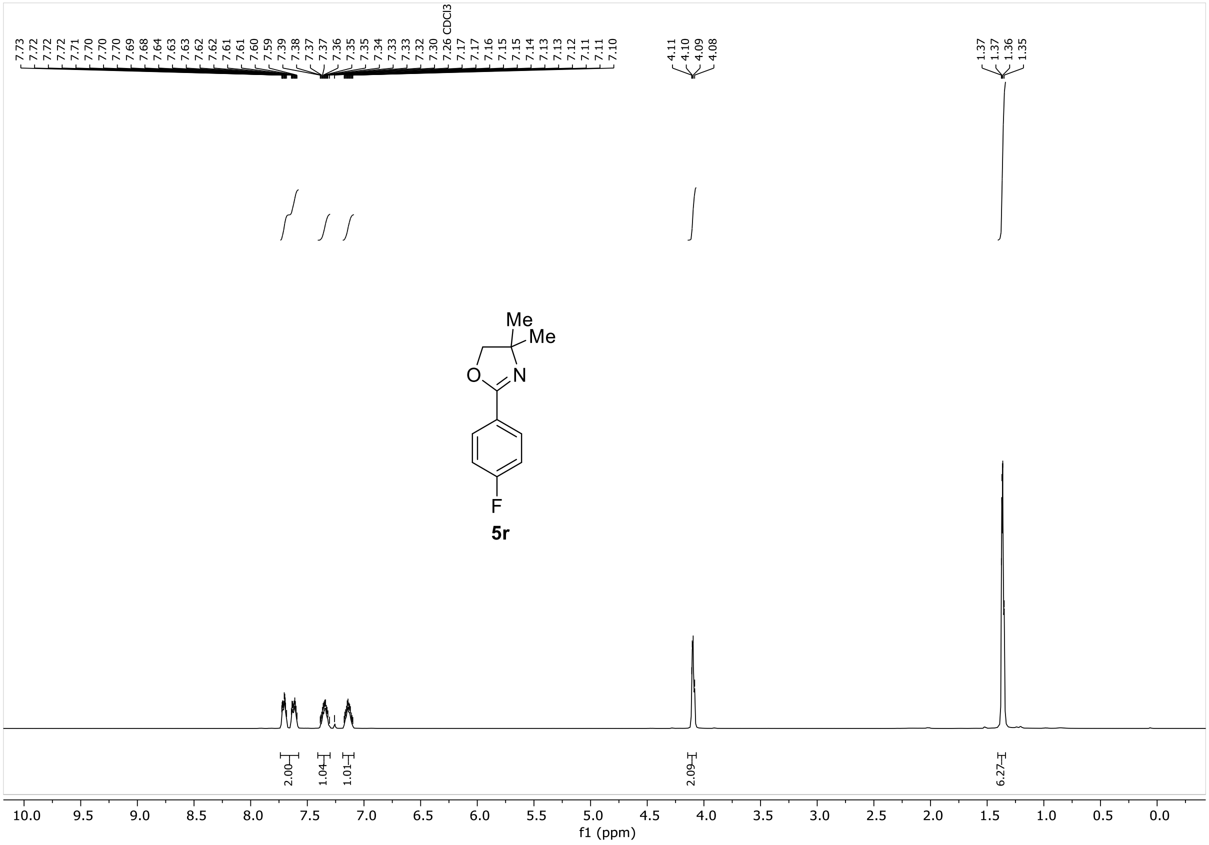
7.73 7.72 7.72 7.72 7.71 7.70 7.70 7.70 7.69 7.68 7.64 7.63 7.63 7.62 7.62 7.61 7.61 7.60 7.59 7.39 7.38 7.37 7.37 7.36 7.35 7.35 7.34 7.33 7.33 7.32 7.30 7.26 CDCl3 7.17 7.17 7.16 7.15 7.15 7.14 7.13 7.13 7.12 7.11 7.11 7.10
4.11 4.10 4.09 4.08
1.37 1.37 1.36 1.35
Me
Me
O
N
F
5r
2.00
1.04
1.01
2.09
6.27
10.0 9.5 9.0 8.5 8.0 7.5 7.0 6.5 6.0 5.5 5.0 4.5 4.0 3.5 3.0 2.5 2.0 1.5 1.0 0.5 0.0
f1 (ppm)

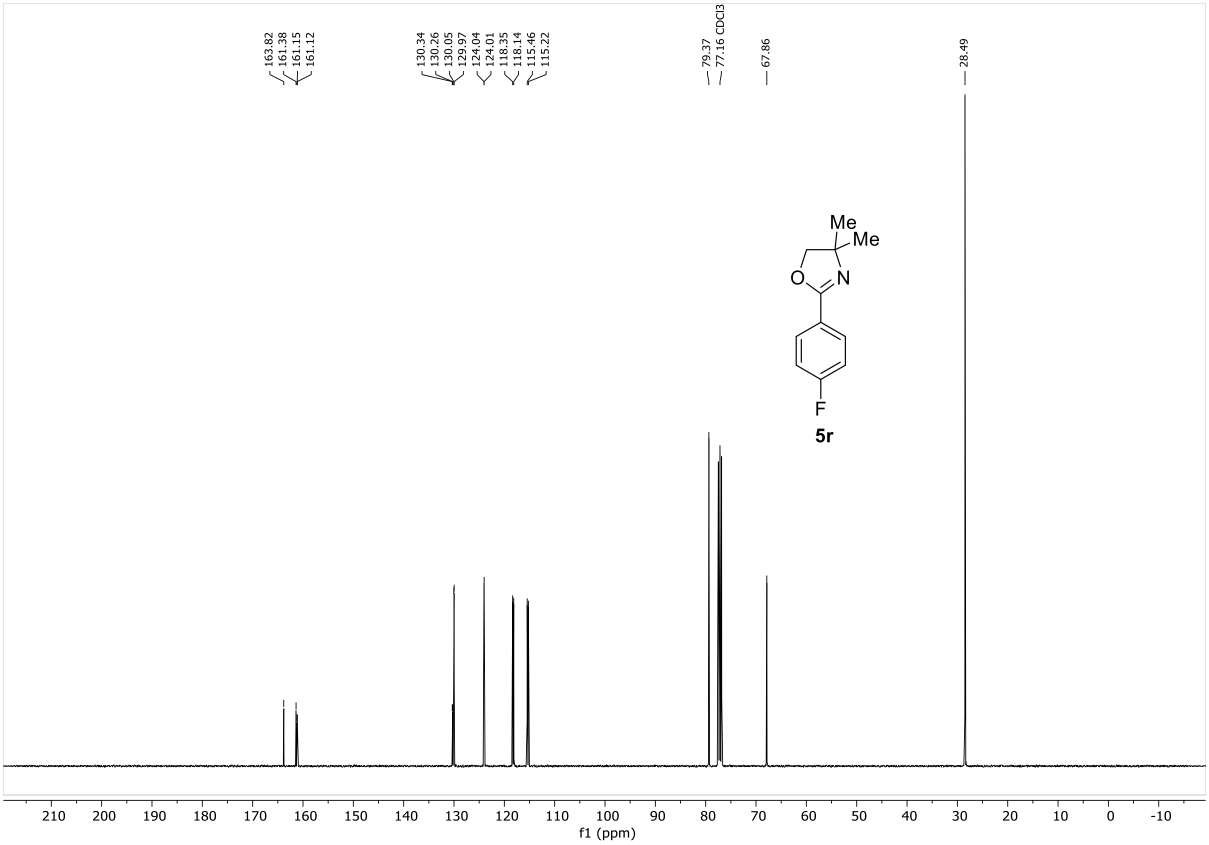
163.82 161.38 161.15 161.12
130.34 130.26 130.05 129.97 124.04 124.01 118.35 118.14 115.46 115.22
79.37 77.16 CDCl3
67.86
28.49
Me
Me
O
N
F
5r
210 200 190 180 170 160 150 140 130 120 110 100 90 80 70 60 50 40 30 20 10 0 -10
f1 (ppm)

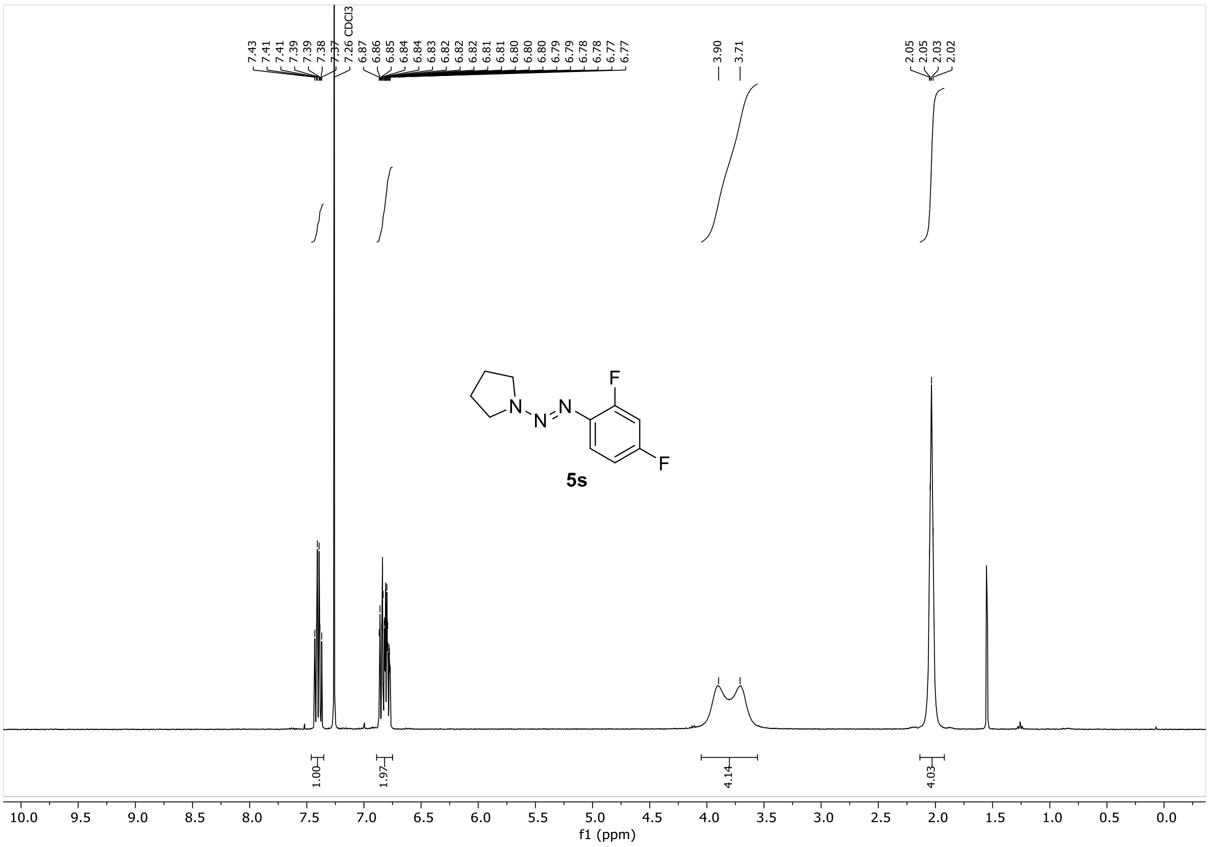

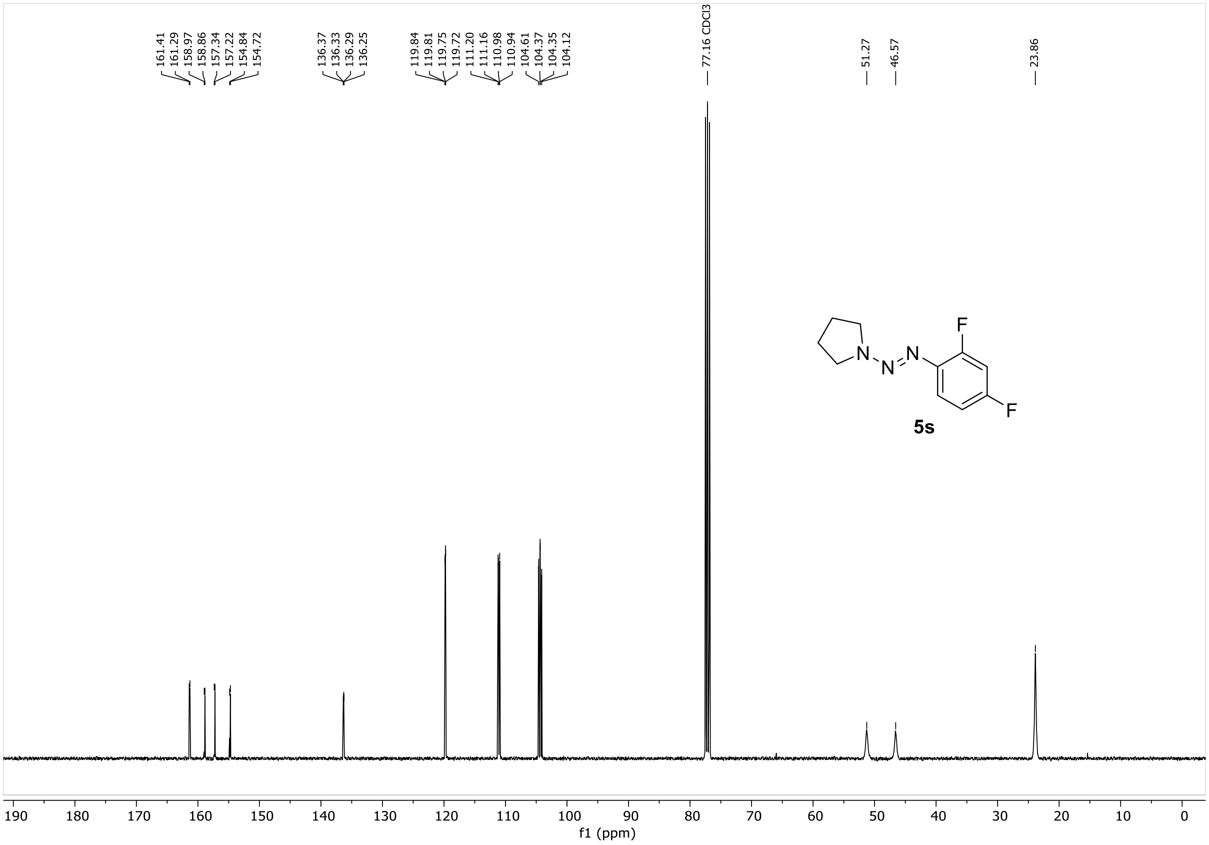

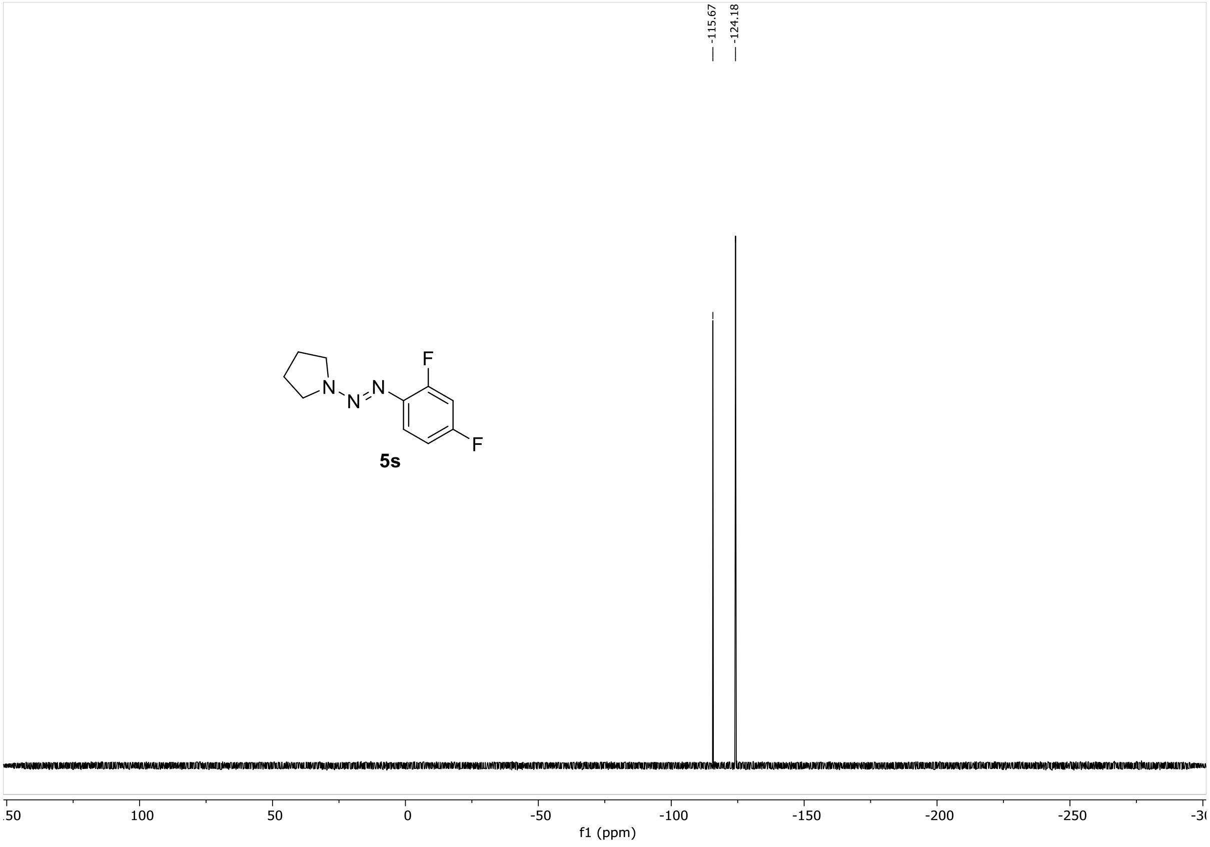
-115.67
-124.18
F
N
N
N
F
5s
150 100 50 0 -50 -100 -150 -200 -250 -300
f1 (ppm)

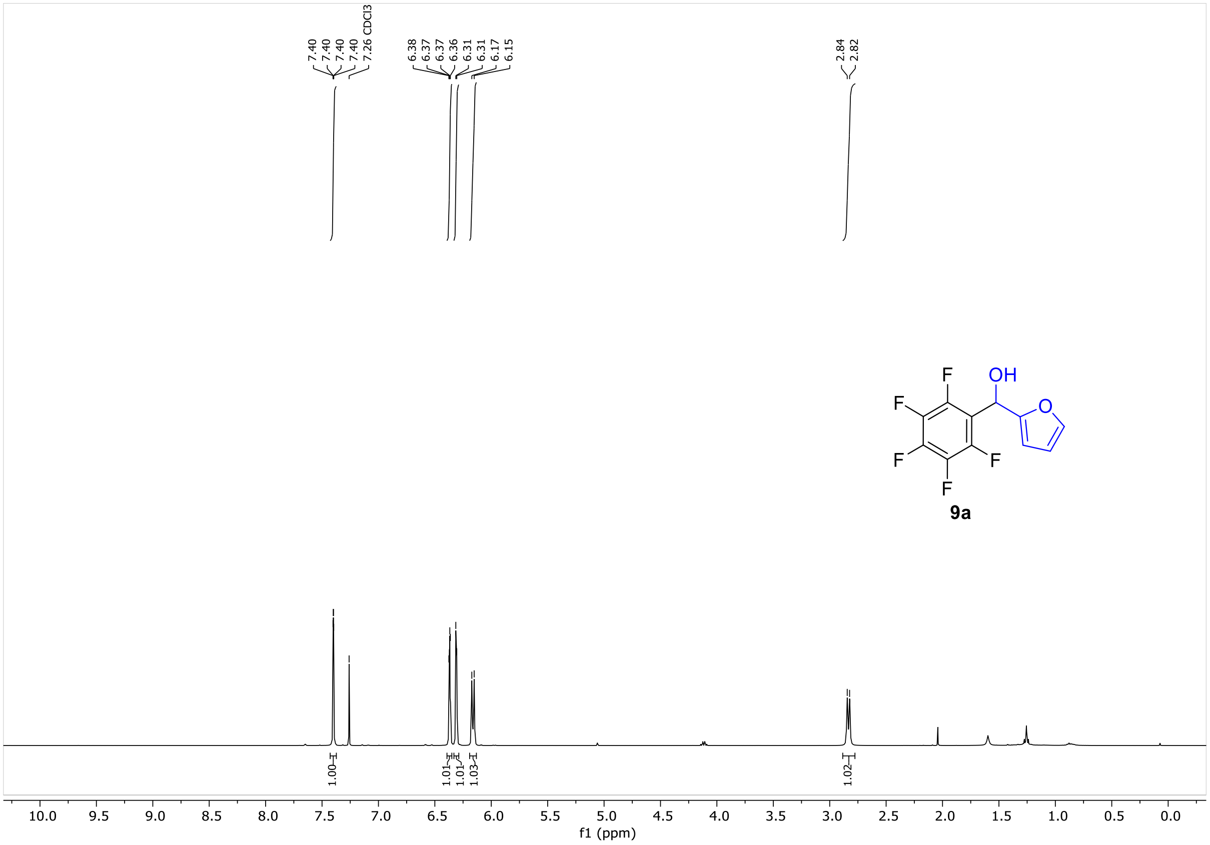
7.40
7.40
7.40
7.40
7.26 CDCl3
6.38
6.37
6.37
6.36
6.31
6.31
6.17
6.15
2.84
2.82
F
OH
F
O
F
F
F
9a
1.00
1.01
1.01
1.03
1.02
10.0 9.5 9.0 8.5 8.0 7.5 7.0 6.5 6.0 5.5 5.0 4.5 4.0 3.5 3.0 2.5 2.0 1.5 1.0 0.5 0.0
f1 (ppm)

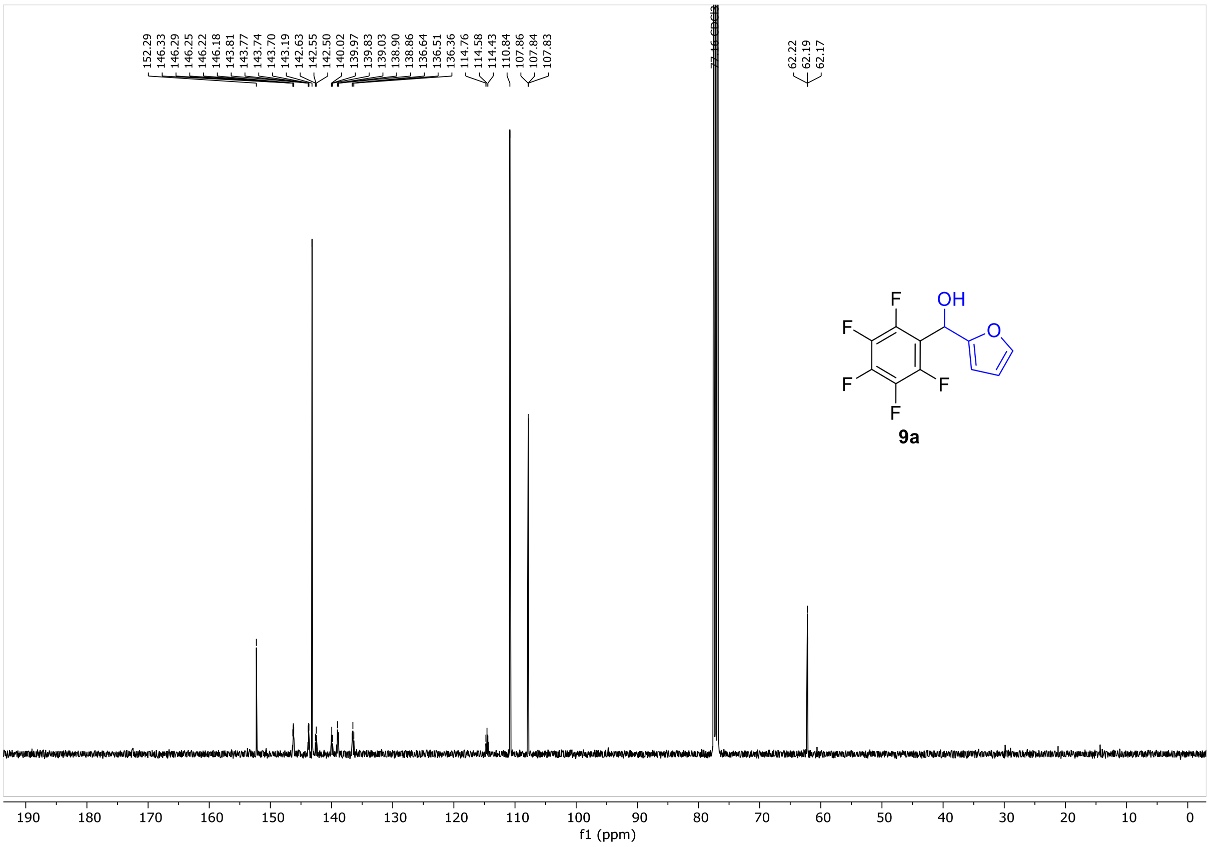
152.29
146.33
146.29
146.25
146.22
146.18
143.81
143.77
143.74
143.70
143.19
142.63
142.55
142.50
140.02
139.97
139.83
139.03
138.90
138.86
136.64
136.51
136.36
114.76
114.58
114.43
110.84
107.86
107.84
107.83
77.16 CDCl3
62.22
62.19
62.17
F
OH
O
9a
190 180 170 160 150 140 130 120 110 100 90 80 70 60 50 40 30 20 10 0
f1 (ppm)

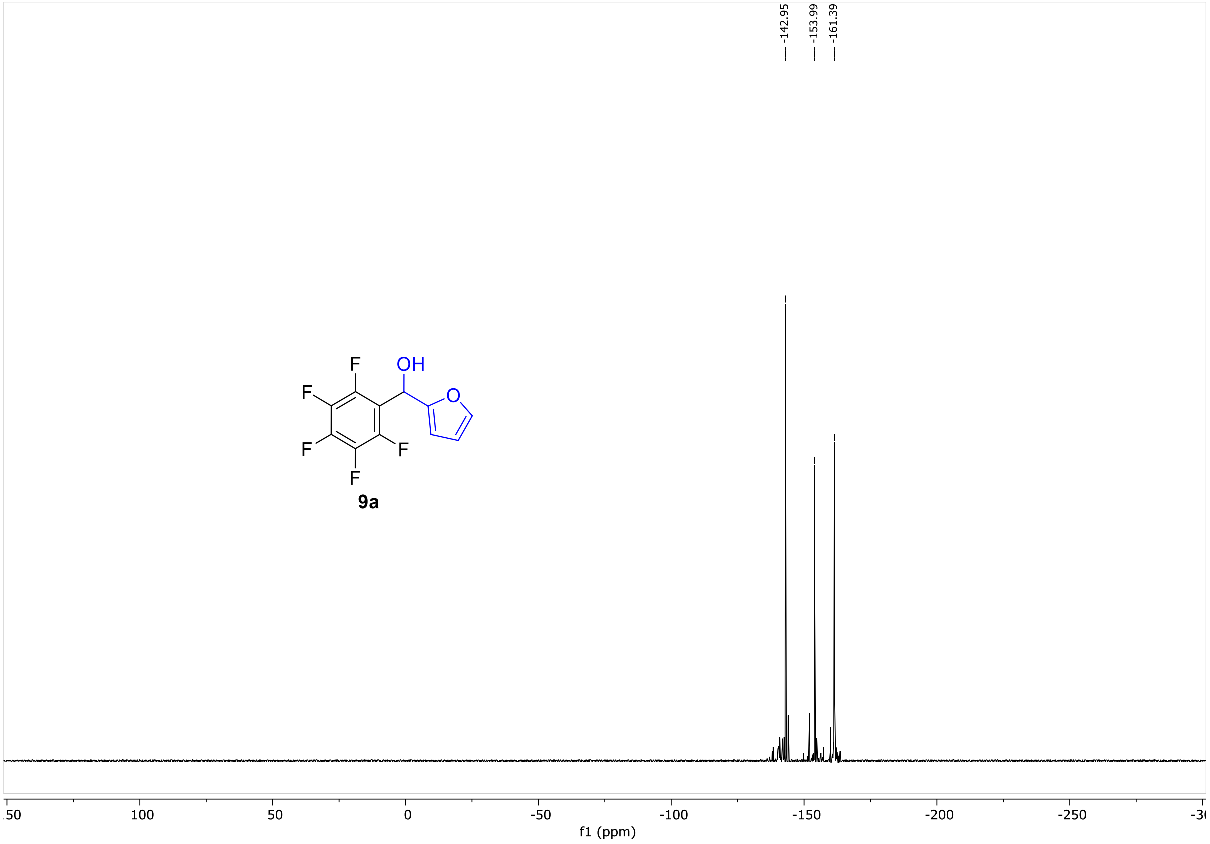
-142.95
-153.99
-161.39
F
OH
O
9a
100 50 0 -50 -100 -150 -200 -250
f1 (ppm)

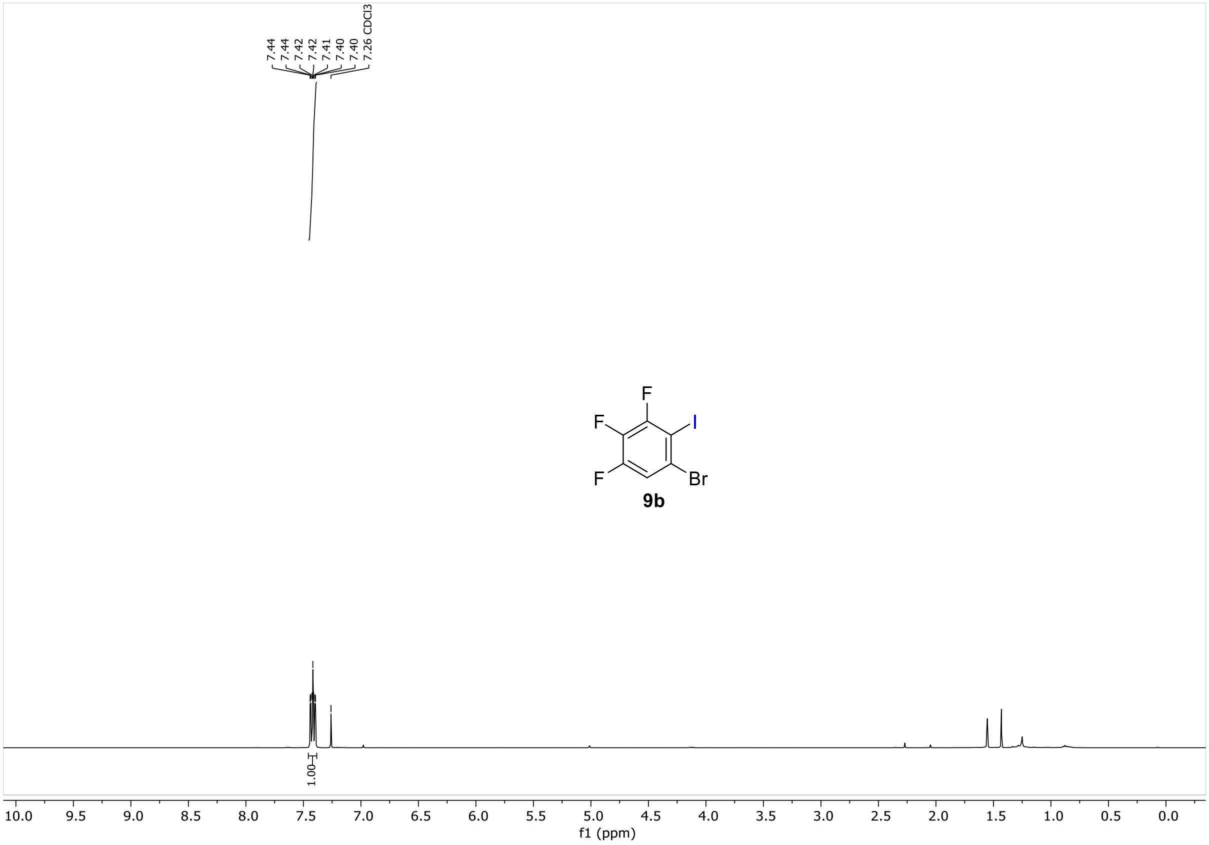
7.44
7.44
7.42
7.42
7.41
7.40
7.40
7.26 CDCl3
9b
1.00
f1 (ppm)

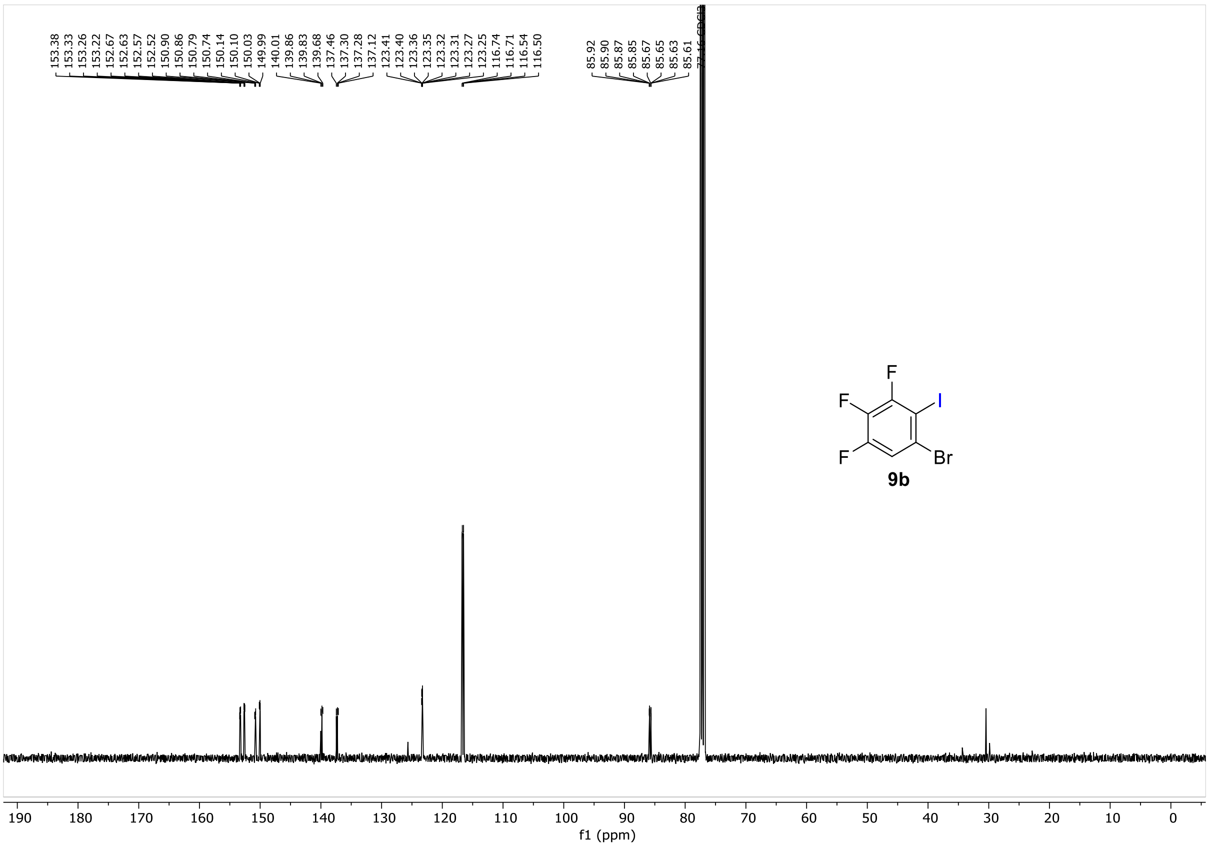
153.38
153.33
153.26
153.22
152.67
152.63
152.57
152.52
150.90
150.86
150.79
150.74
150.14
150.10
150.03
149.99
140.01
139.86
139.83
139.68
137.46
137.30
137.28
137.12
123.41
123.40
123.36
123.35
123.32
123.31
123.27
123.25
116.74
116.71
116.54
116.50
85.92
85.90
85.87
85.85
85.67
85.65
85.63
85.61
9b
f1 (ppm)

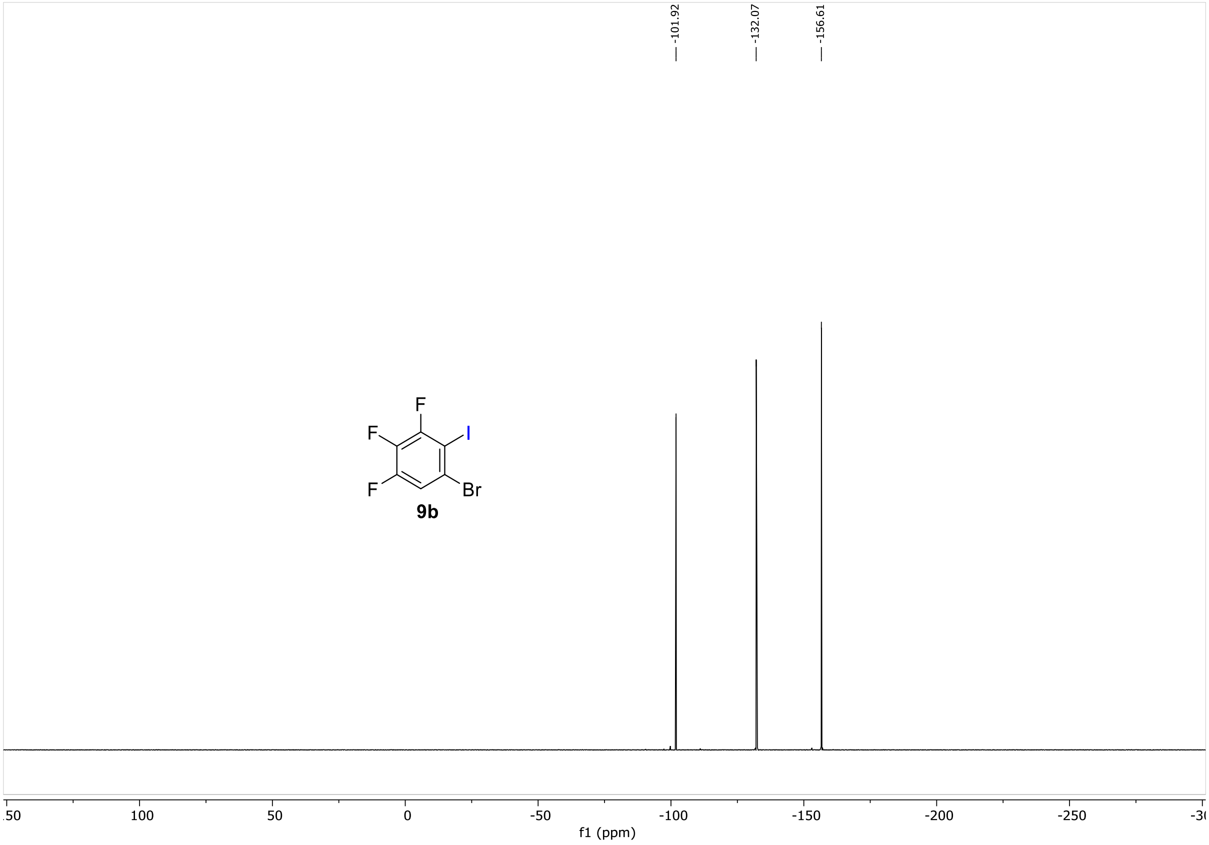
-101.92
-132.07
-156.61
F
F
I
F
Br
9b
f1 (ppm)

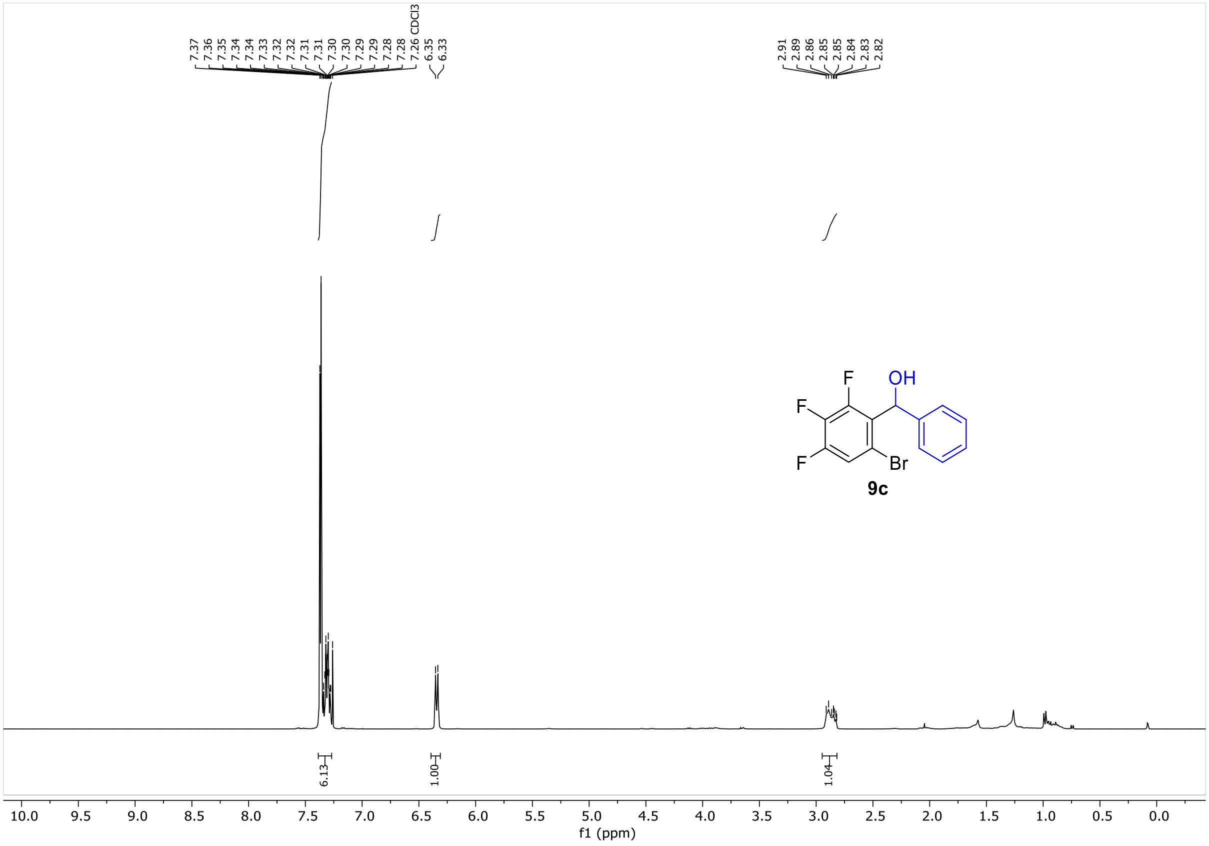
7.37 7.36 7.35 7.34 7.34 7.33 7.32 7.32 7.31 7.31 7.30 7.30 7.29 7.29 7.28 7.28 7.26 CDCl3 6.35 6.33
2.91 2.89 2.86 2.85 2.85 2.84 2.83 2.82
F
OH
F
F
Br
9c
6.13
1.00
1.04
f1 (ppm)

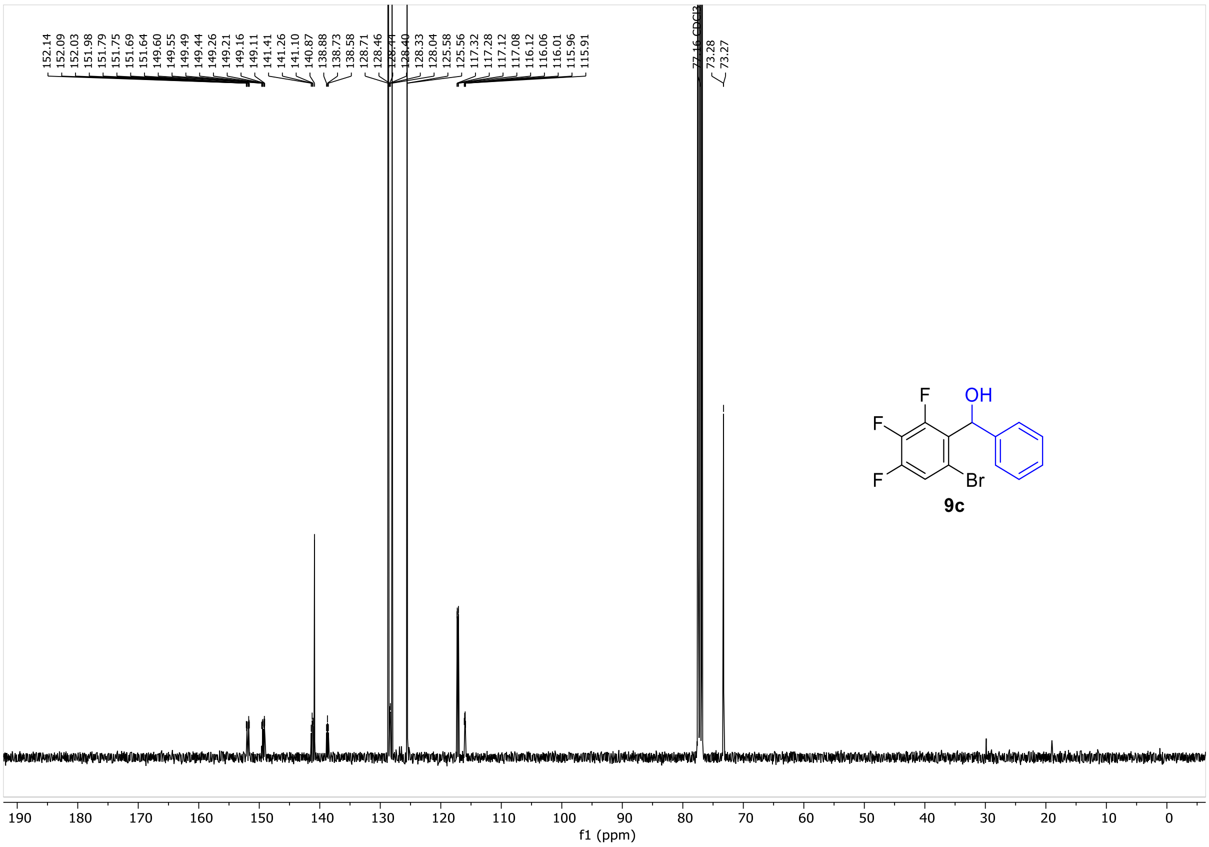
152.14
152.09
152.03
151.98
151.79
151.75
151.69
151.64
149.60
149.55
149.49
149.44
149.26
149.21
149.16
149.11
141.41
141.26
141.10
140.87
138.88
138.73
138.58
128.71
128.46
128.44
128.40
128.33
128.04
125.58
125.56
117.32
117.28
117.12
117.08
116.12
116.06
116.01
115.96
115.91
77.16 CDCl3
73.28
73.27
F
F
F
OH
Br
9c
190 180 170 160 150 140 130 120 110 100 90 80 70 60 50 40 30 20 10 0
f1 (ppm)

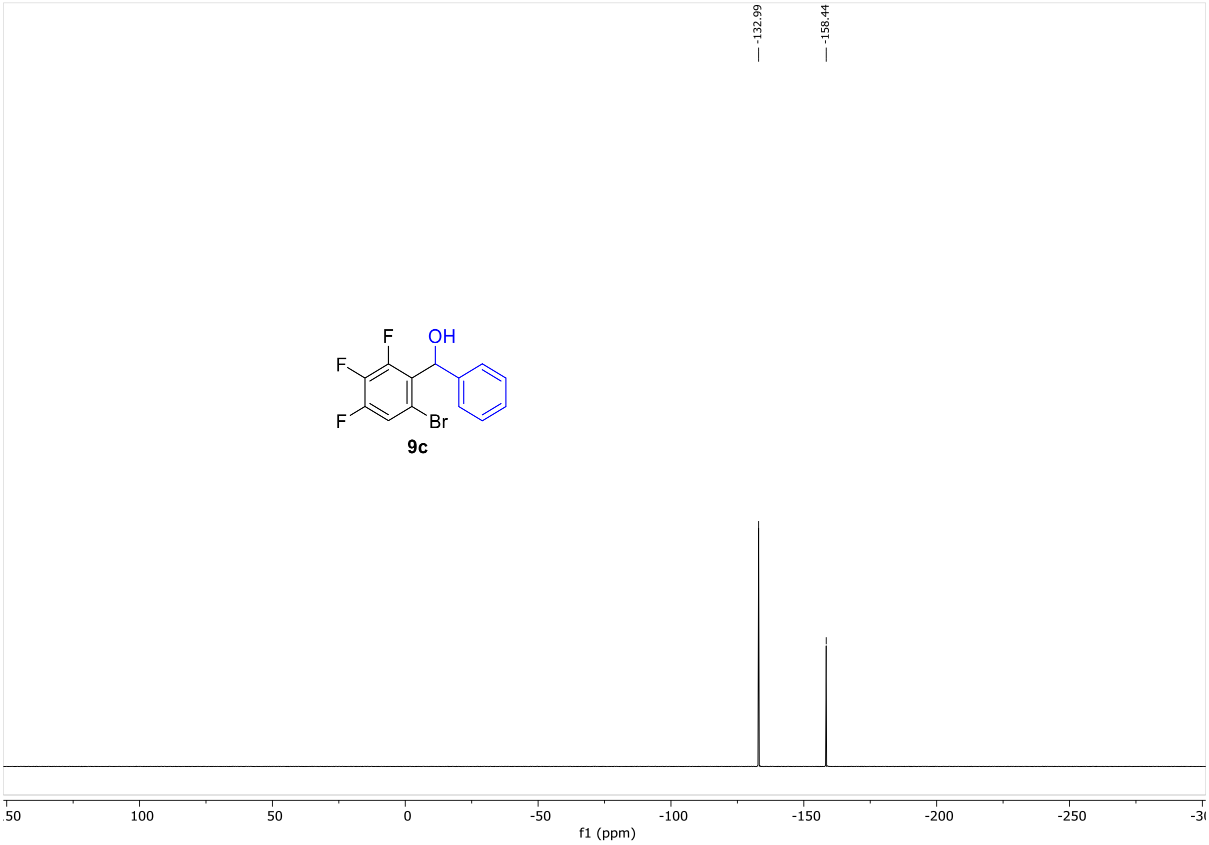
-132.99
-158.44
F
F
F
OH
Br
9c
100 50 0 -50 -100 -150 -200 -250
f1 (ppm)

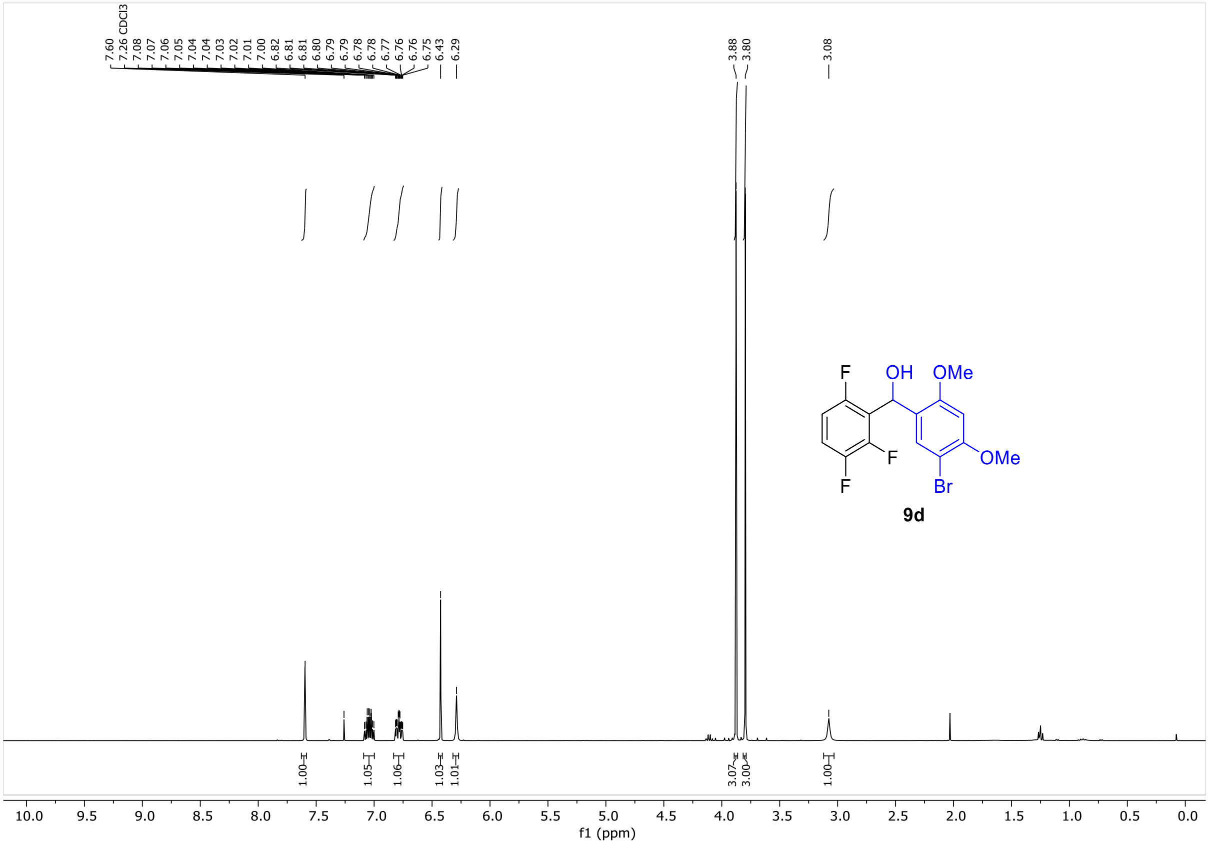
7.60
7.26 CDCl3
7.08
7.07
7.06
7.05
7.04
7.04
7.03
7.02
7.01
7.00
6.82
6.81
6.81
6.80
6.79
6.79
6.78
6.78
6.77
6.76
6.76
6.75
6.43
6.29
3.88
3.80
3.08
1.00
1.05
1.06
1.03
1.01
3.07
3.00
1.00
F
OH
OMe
F
OMe
F
Br
9d
10.0 9.5 9.0 8.5 8.0 7.5 7.0 6.5 6.0 5.5 5.0 4.5 4.0 3.5 3.0 2.5 2.0 1.5 1.0 0.5 0.0
f1 (ppm)

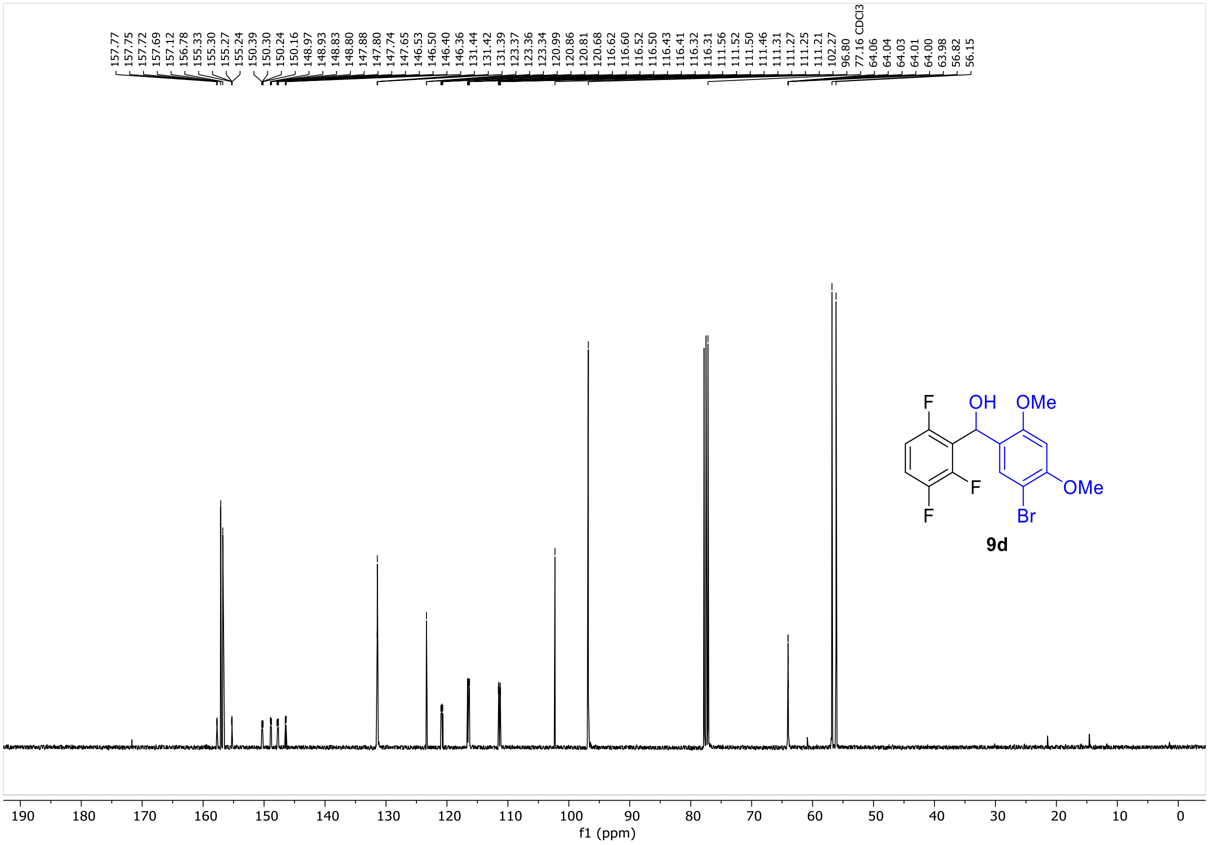
157.77
157.75
157.72
157.69
157.12
156.78
155.33
155.30
155.27
155.24
150.39
150.30
150.24
150.16
148.97
148.93
148.83
148.80
147.88
147.80
147.74
147.65
146.53
146.50
146.40
146.36
131.44
131.42
131.39
123.37
123.36
123.34
120.99
120.86
120.81
120.68
116.62
116.60
116.52
116.50
116.43
116.41
116.32
116.31
111.56
111.52
111.50
111.46
111.31
111.27
111.25
111.21
102.27
96.80
77.16 CDCl3
64.06
64.04
64.03
64.01
64.00
63.98
56.82
56.15
F
OH
OMe
F
OMe
F
Br
9d
190 180 170 160 150 140 130 120 110 100 90 80 70 60 50 40 30 20 10 0
f1 (ppm)

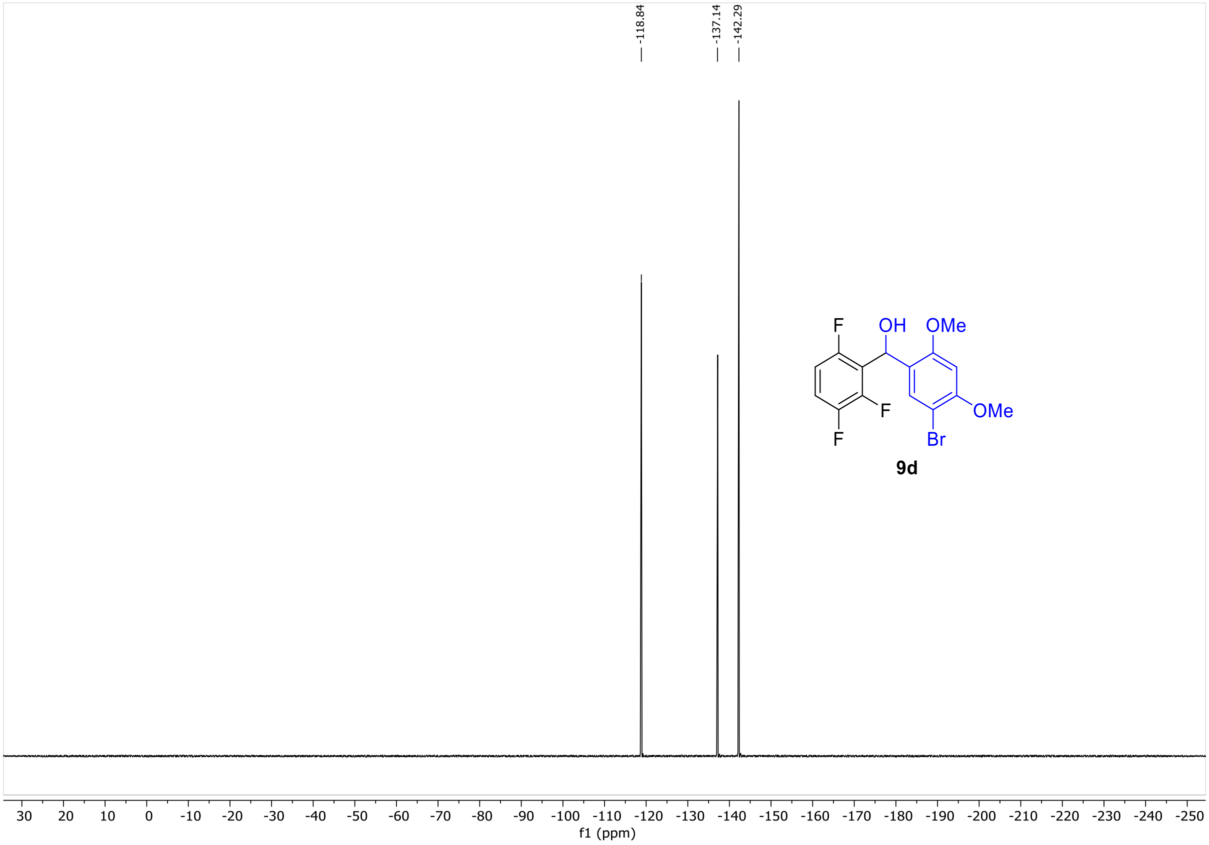
-118.84
-137.14
-142.29
F
OH
OMe
F
OMe
F
Br
9d
30 20 10 0 -10 -20 -30 -40 -50 -60 -70 -80 -90 -100 -110 -120 -130 -140 -150 -160 -170 -180 -190 -200 -210 -220 -230 -240 -250
f1 (ppm)

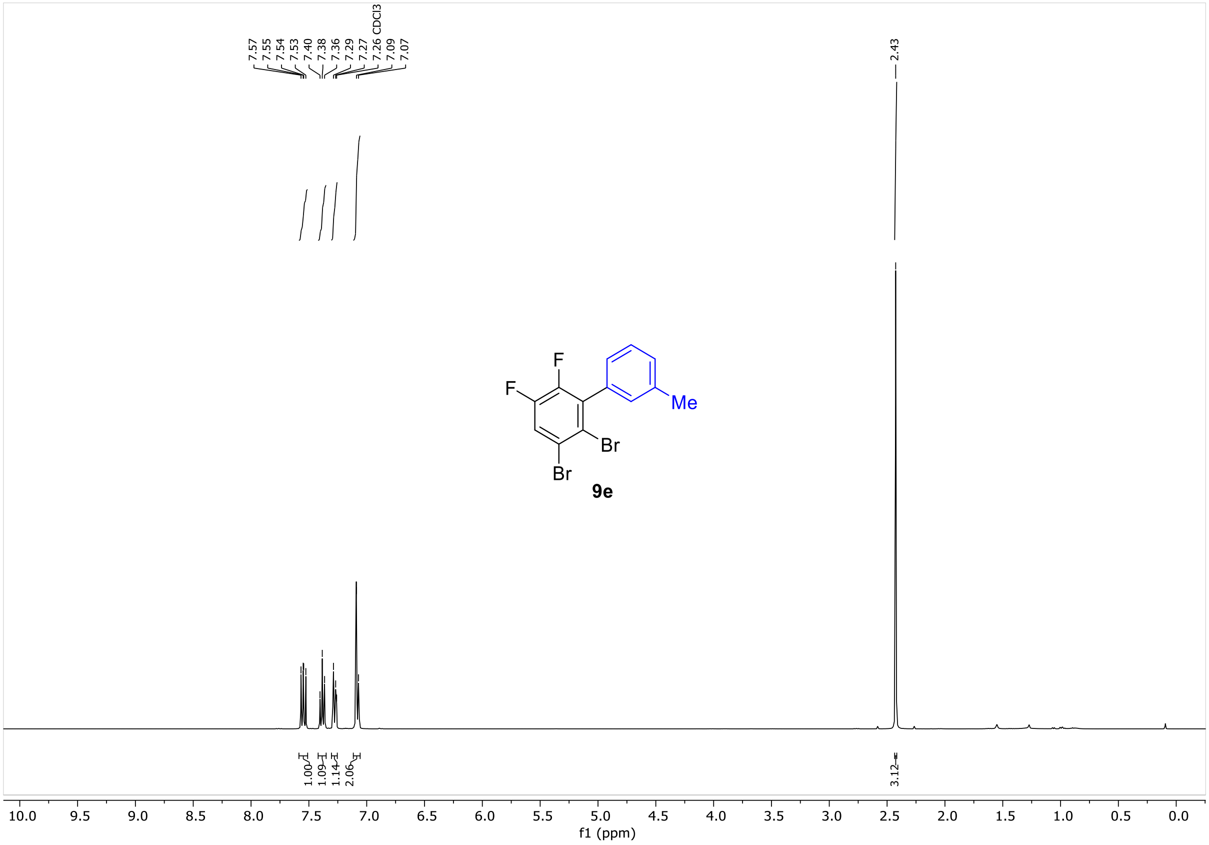
7.57
7.55
7.54
7.53
7.40
7.38
7.36
7.29
7.27
7.26 CDCl3
7.09
7.07
2.43
F
F
Me
Br
Br
9e
1.00
1.09
1.14
2.06
3.12
10.0 9.5 9.0 8.5 8.0 7.5 7.0 6.5 6.0 5.5 5.0 4.5 4.0 3.5 3.0 2.5 2.0 1.5 1.0 0.5 0.0
f1 (ppm)

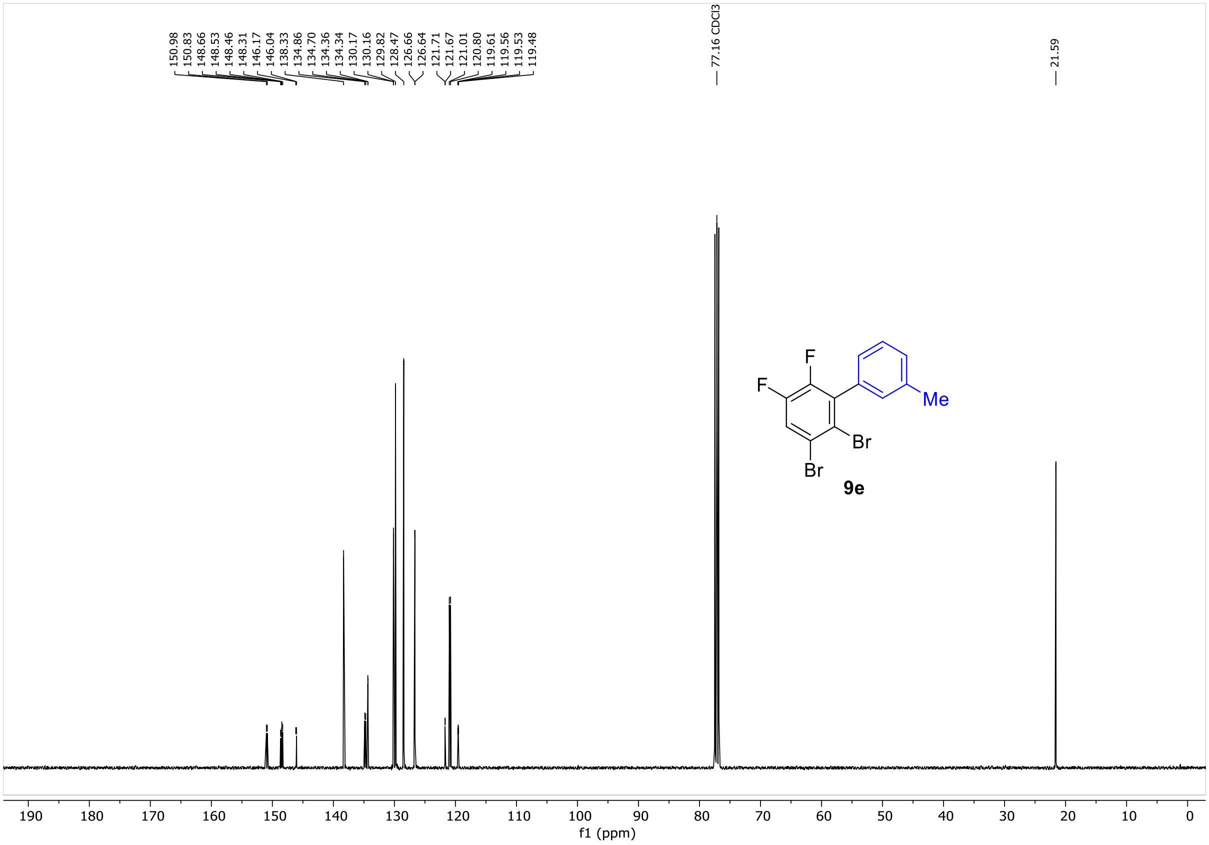
150.98
150.83
148.66
148.53
148.46
148.31
146.17
146.04
138.33
134.86
134.70
134.36
134.34
130.17
130.16
129.82
128.47
126.66
126.64
121.71
121.67
121.01
120.80
119.61
119.56
119.53
119.48
77.16 CDCl3
21.59
F
F
Me
Br
Br
9e
190 180 170 160 150 140 130 120 110 100 90 80 70 60 50 40 30 20 10 0
f1 (ppm)

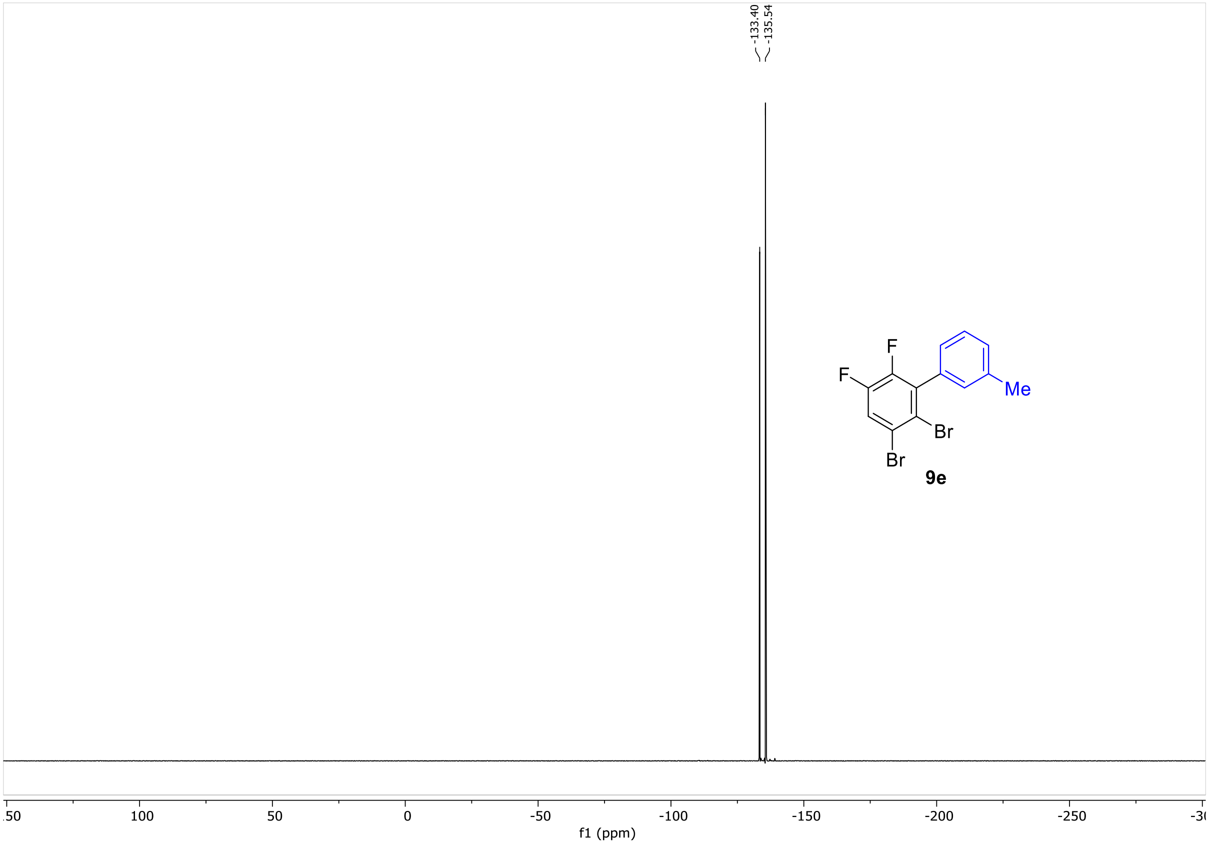
-133.40
-135.54
F
F
Me
Br
Br
9e
100 50 0 -50 -100 -150 -200 -250
f1 (ppm)

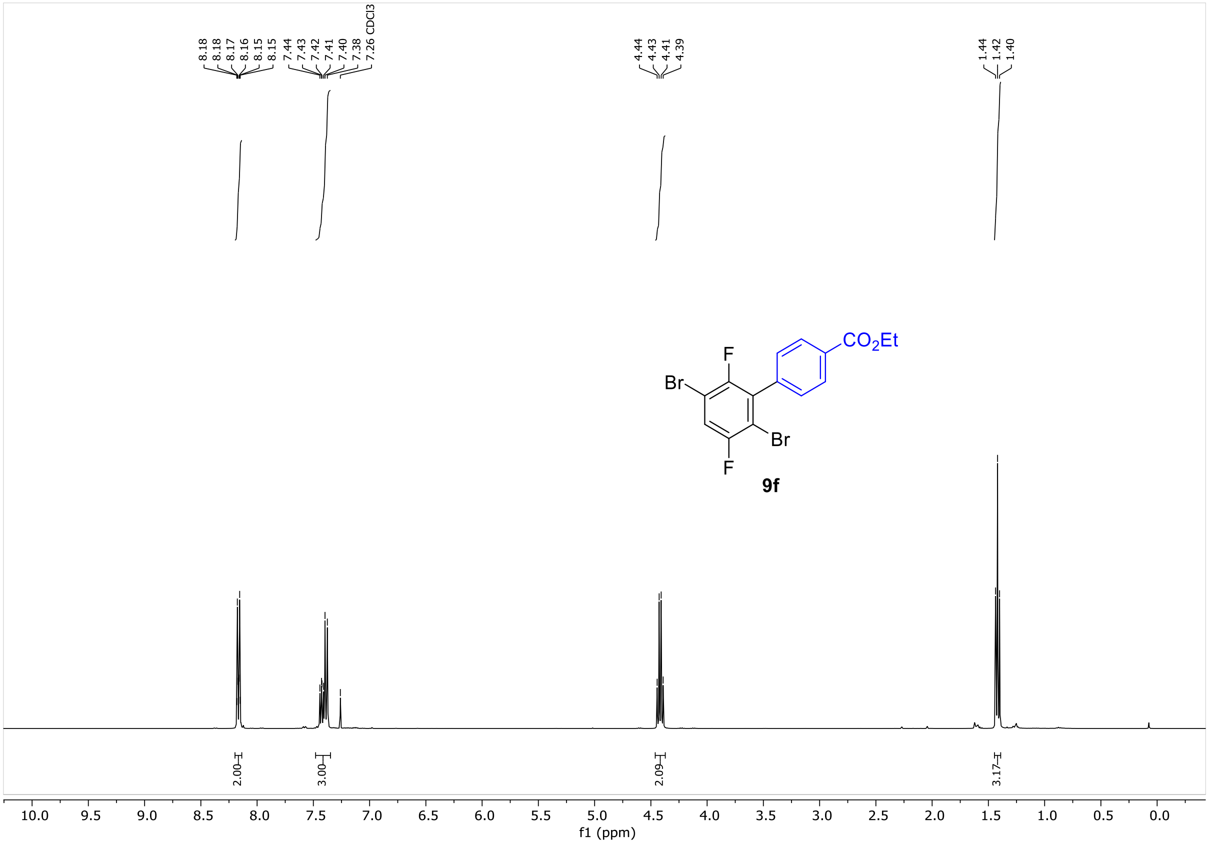
8.18
8.18
8.17
8.16
8.15
8.15
7.44
7.43
7.42
7.41
7.40
7.38
7.26 CDCl3
4.44
4.43
4.41
4.39
1.44
1.42
1.40
CO2Et
F
Br
Br
F
9f
2.00
3.00
2.09
3.17
f1 (ppm)

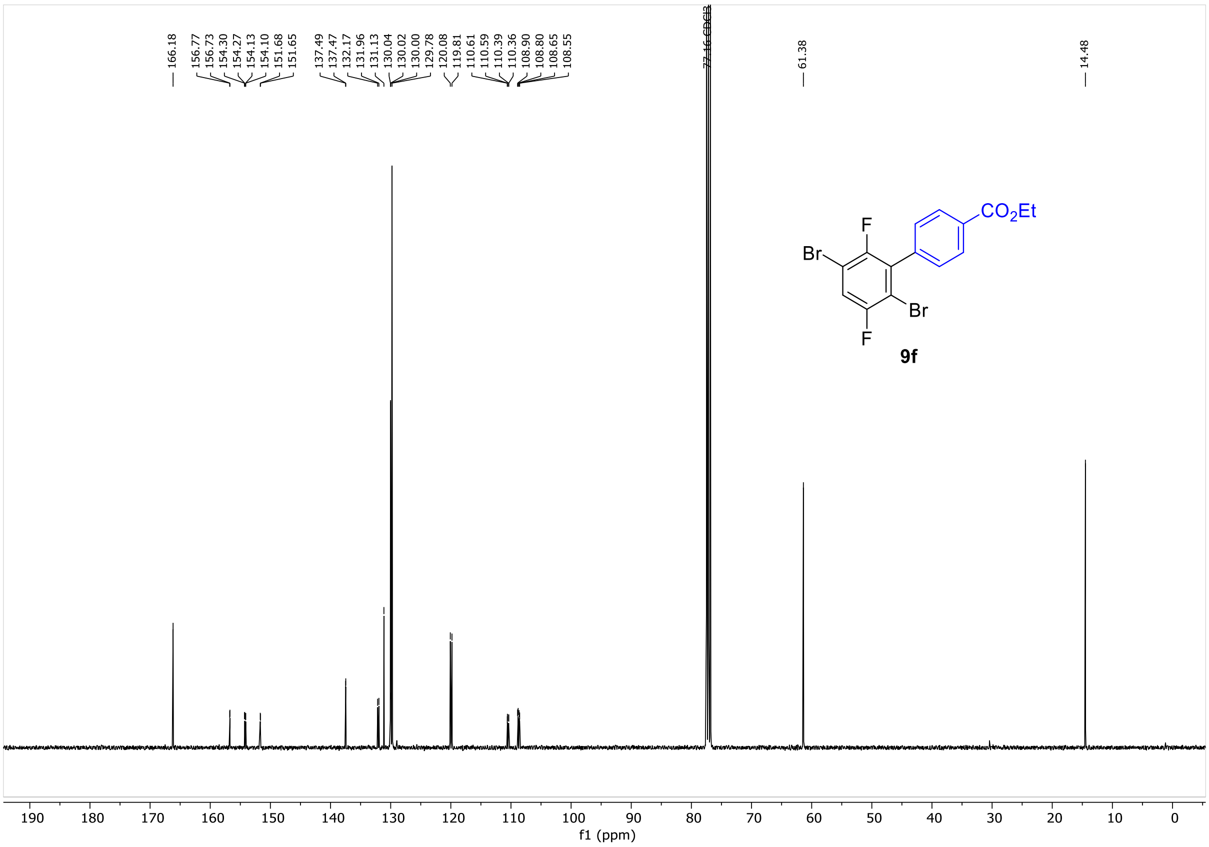
166.18
156.77
156.73
154.30
154.27
154.13
154.10
151.68
151.65
137.49
137.47
132.17
131.96
131.13
130.04
130.02
130.00
129.78
120.08
119.81
110.61
110.59
110.39
110.36
108.90
108.80
108.65
108.55
77.16 CDCl3
61.38
14.48
CO2Et
F
Br
Br
F
9f
f1 (ppm)

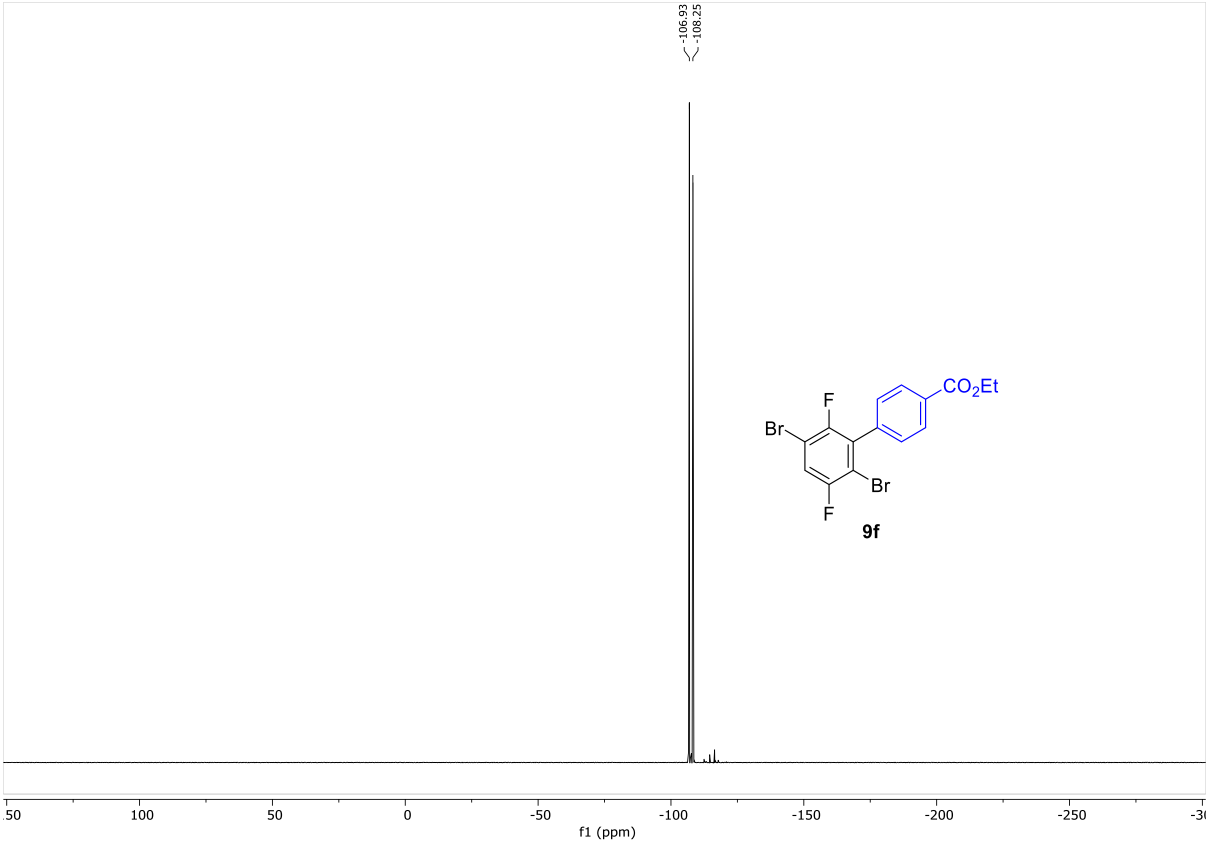
-106.93
-108.25
CO2Et
F
Br
Br
F
9f
50
100
50
0
-50
-100
-150
-200
-250
f1 (ppm)

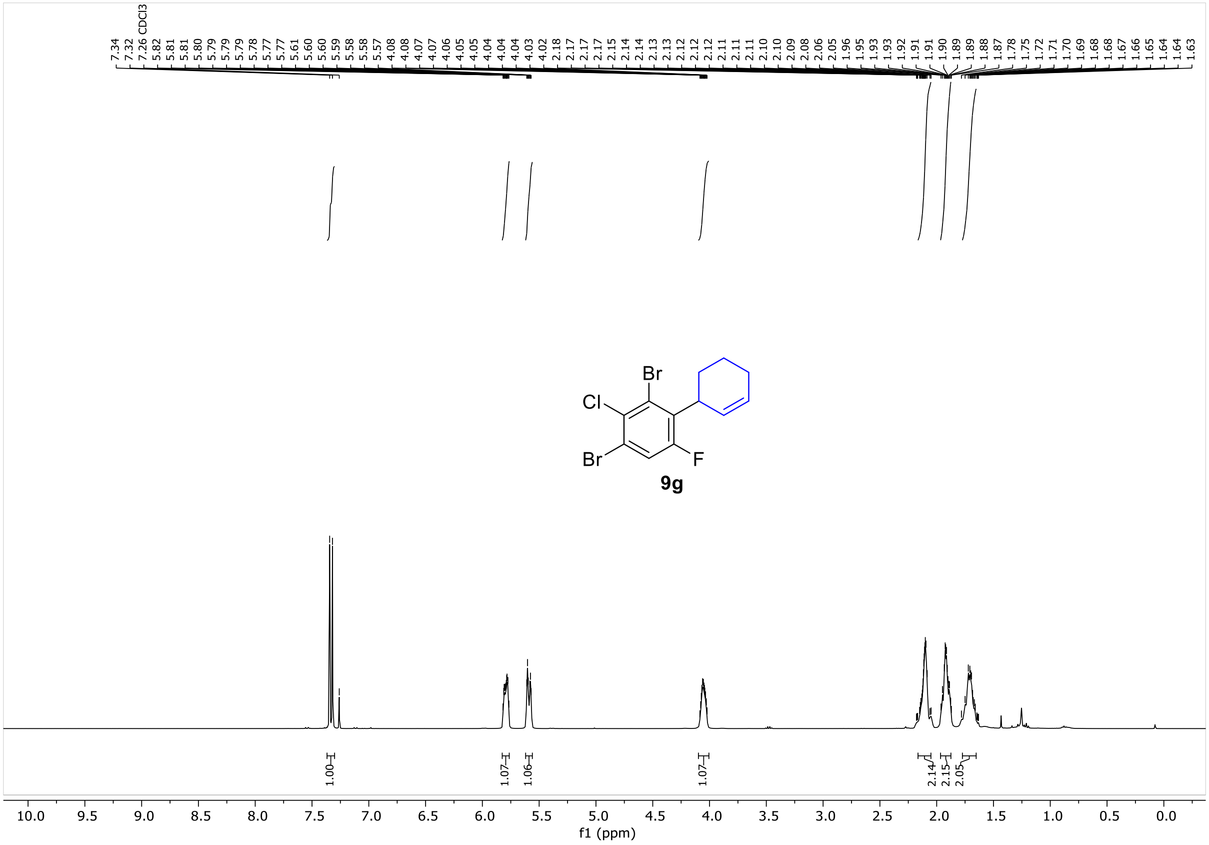
7.34 7.32 7.26 CDCl3 5.82 5.81 5.81 5.80 5.79 5.79 5.78 5.77 5.61 5.60 5.59 5.58 5.58 5.57 4.08 4.08 4.07 4.07 4.06 4.05 4.05 4.04 4.04 4.04 4.03 4.02 2.18 2.17 2.17 2.15 2.14 2.14 2.13 2.13 2.12 2.12 2.11 2.11 2.11 2.10 2.09 2.08 2.06 2.05 1.96 1.95 1.93 1.93 1.92 1.91 1.91 1.90 1.89 1.89 1.88 1.87 1.78 1.75 1.72 1.71 1.70 1.69 1.68 1.68 1.67 1.66 1.65 1.64 1.64 1.63
Br
Cl
Br
F
9g
1.00
1.07
1.06
1.07
2.14
2.15
2.05
10.0 9.5 9.0 8.5 8.0 7.5 7.0 6.5 6.0 5.5 5.0 4.5 4.0 3.5 3.0 2.5 2.0 1.5 1.0 0.5 0.0
f1 (ppm)

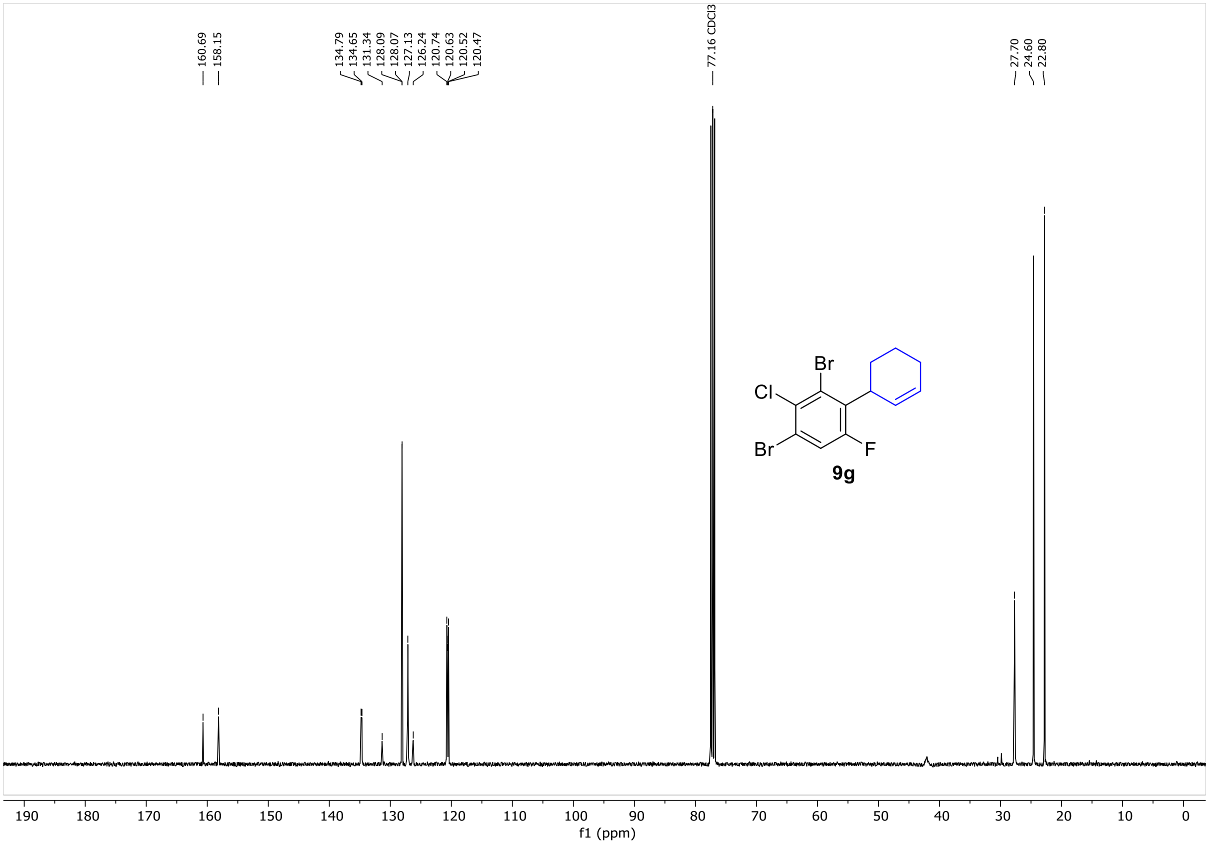
160.69
158.15
134.79
134.65
131.34
128.09
128.07
127.13
126.24
120.74
120.63
120.52
120.47
77.16 CDCl3
27.70
24.60
22.80
Br
Cl
Br
F
9g
190 180 170 160 150 140 130 120 110 100 90 80 70 60 50 40 30 20 10 0
f1 (ppm)

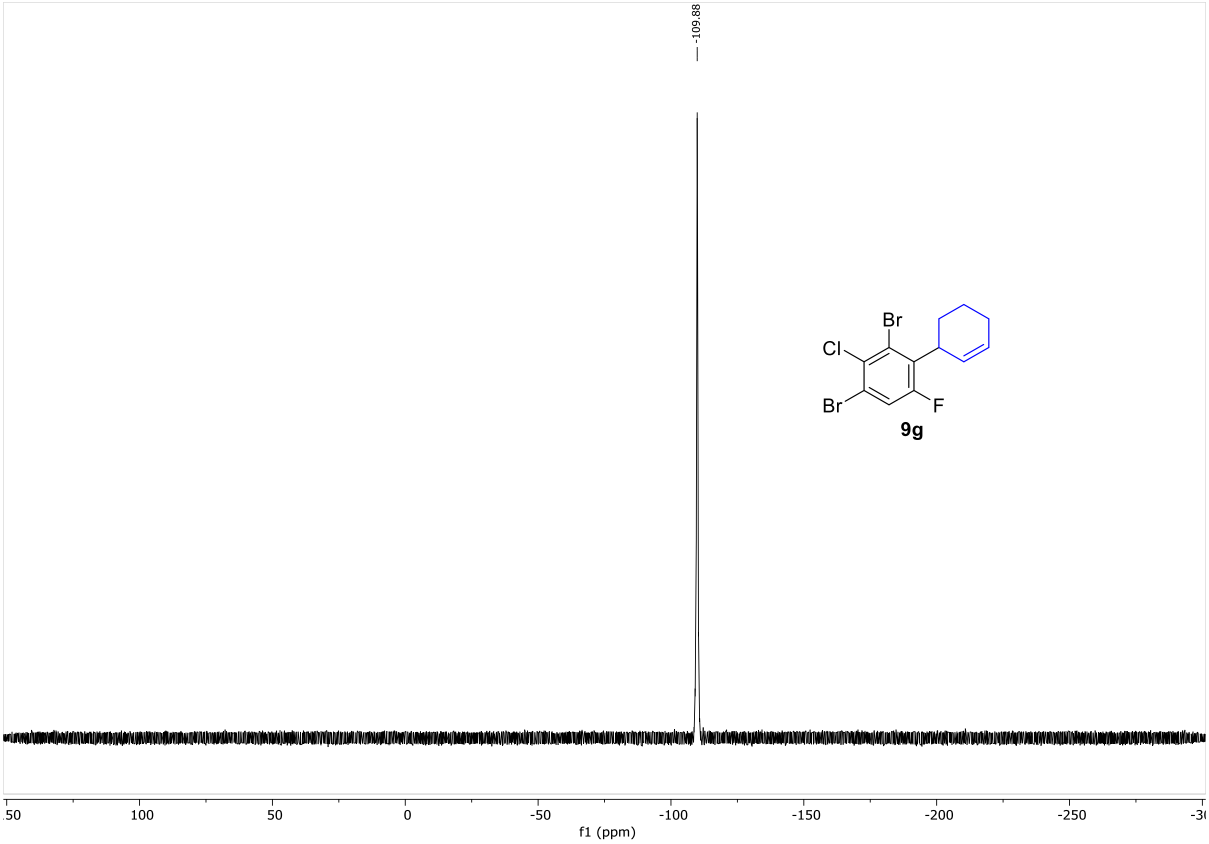
-109.88
Br
Cl
Br
F
9g
100 50 0 -50 -100 -150 -200 -250
f1 (ppm)

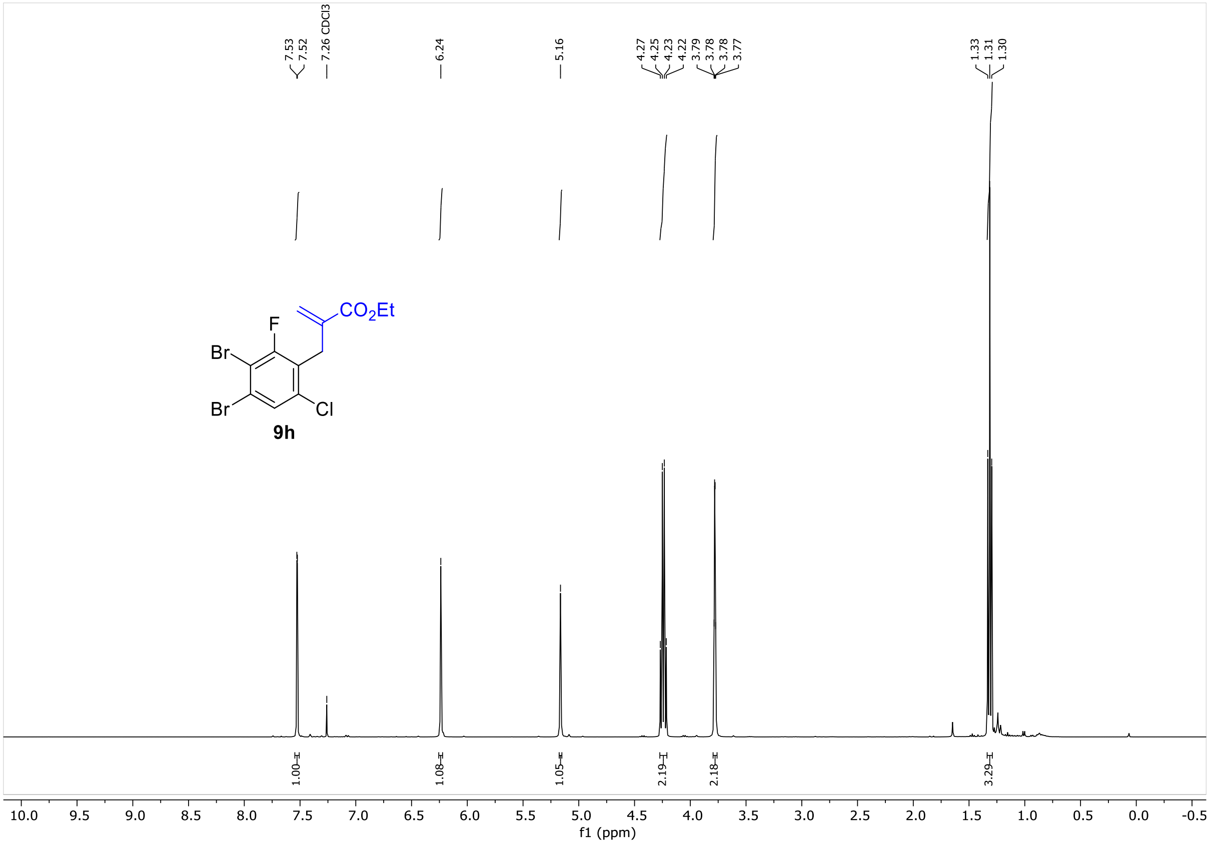
7.53
7.52
7.26 CDCl3
6.24
5.16
4.27
4.25
4.23
4.22
3.79
3.78
3.78
3.77
1.33
1.31
1.30
CO2Et
F
Br
Br
Cl
9h
1.00
1.08
1.05
2.19
2.18
3.29
f1 (ppm)

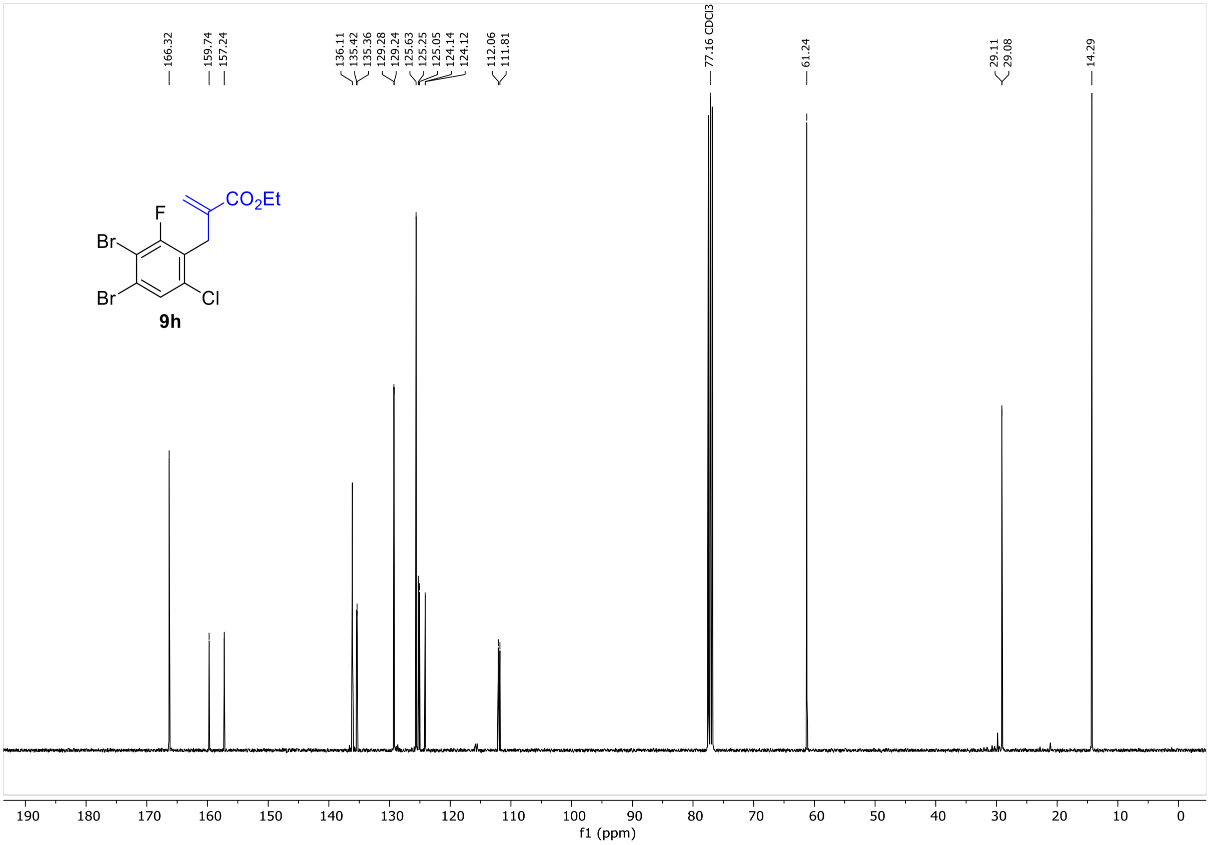
166.32
159.74
157.24
136.11
135.42
135.36
129.28
129.24
125.63
125.25
125.05
124.14
124.12
112.06
111.81
77.16 CDCl3
61.24
29.11
29.08
14.29
CO2Et
F
Br
Br
Cl
9h
f1 (ppm)

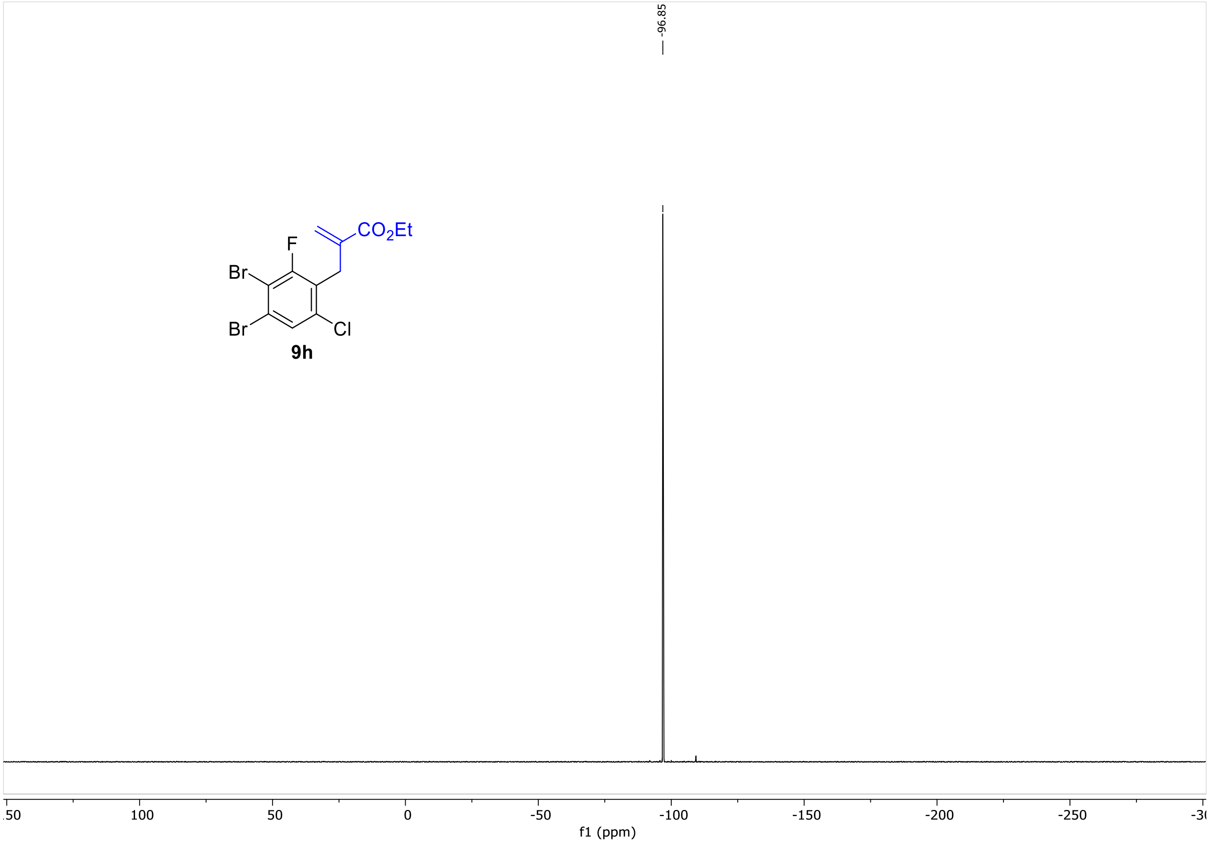
-96.85
F
CO2Et
Br
Br
Cl
9h
150 100 50 0 -50 -100 -150 -200 -250 -300
f1 (ppm)

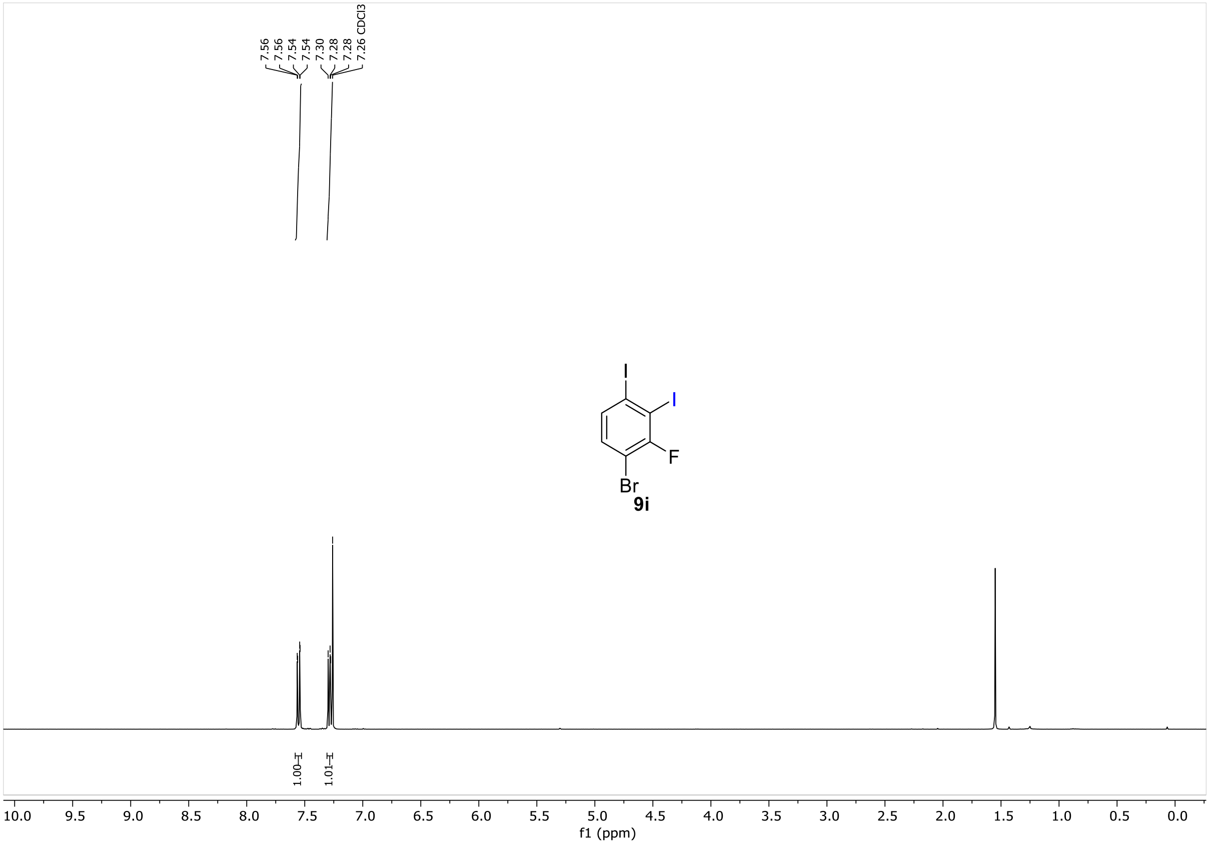
7.56
7.56
7.54
7.54
7.30
7.28
7.28
7.26 CDCl3
I
I
F
Br
9i
1.00
1.01
10.0 9.5 9.0 8.5 8.0 7.5 7.0 6.5 6.0 5.5 5.0 4.5 4.0 3.5 3.0 2.5 2.0 1.5 1.0 0.5 0.0
f1 (ppm)

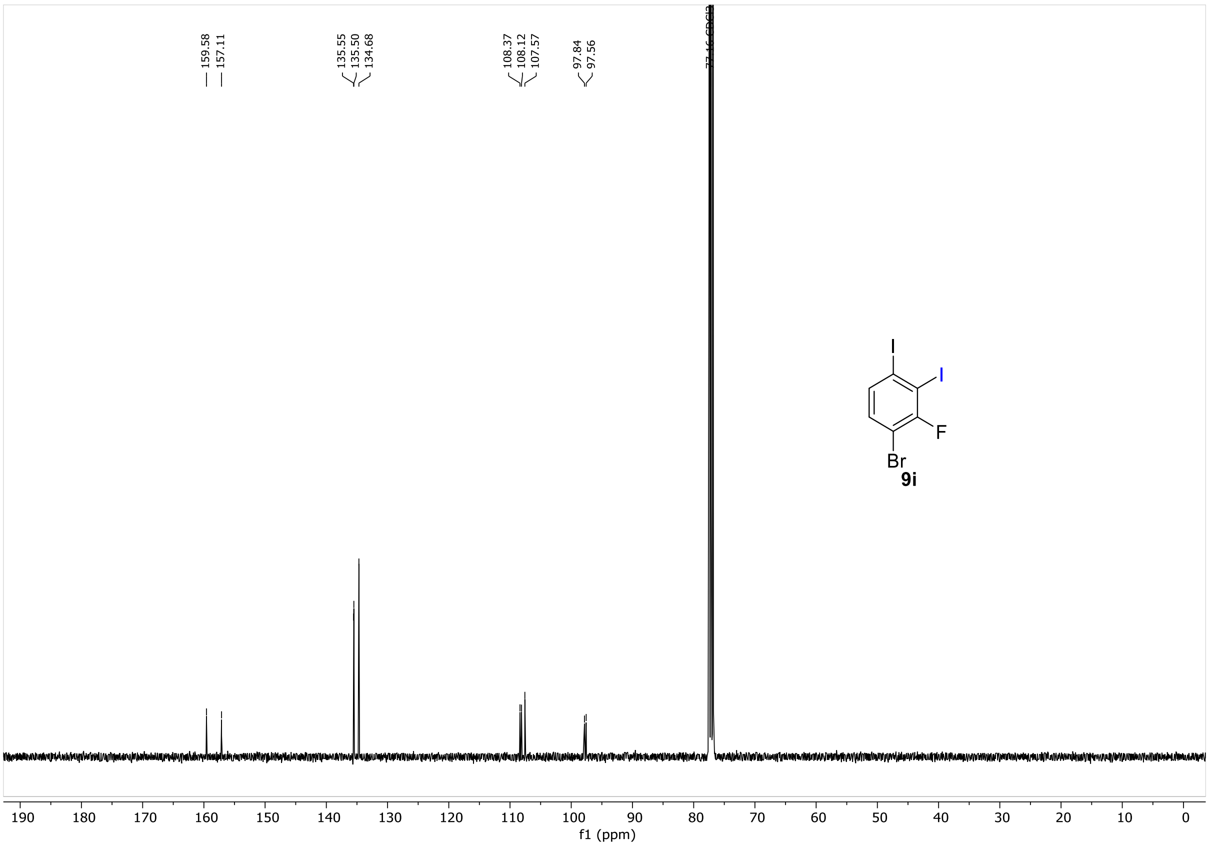
159.58
157.11
135.55
135.50
134.68
108.37
108.12
107.57
97.84
97.56
I
I
F
Br
9i
190 180 170 160 150 140 130 120 110 100 90 80 70 60 50 40 30 20 10 0
f1 (ppm)

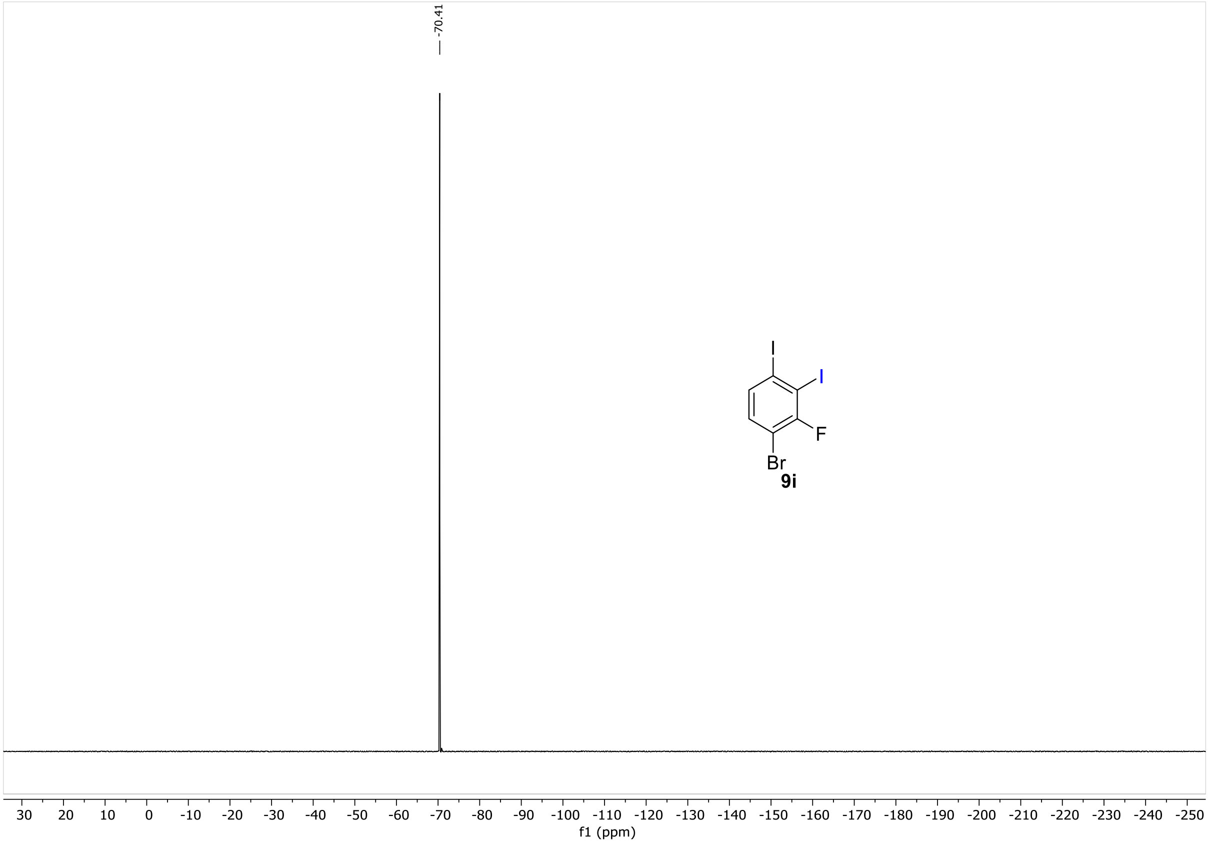
-70.41
I
I
F
Br
9i
30 20 10 0 -10 -20 -30 -40 -50 -60 -70 -80 -90 -100 -110 -120 -130 -140 -150 -160 -170 -180 -190 -200 -210 -220 -230 -240 -250
f1 (ppm)

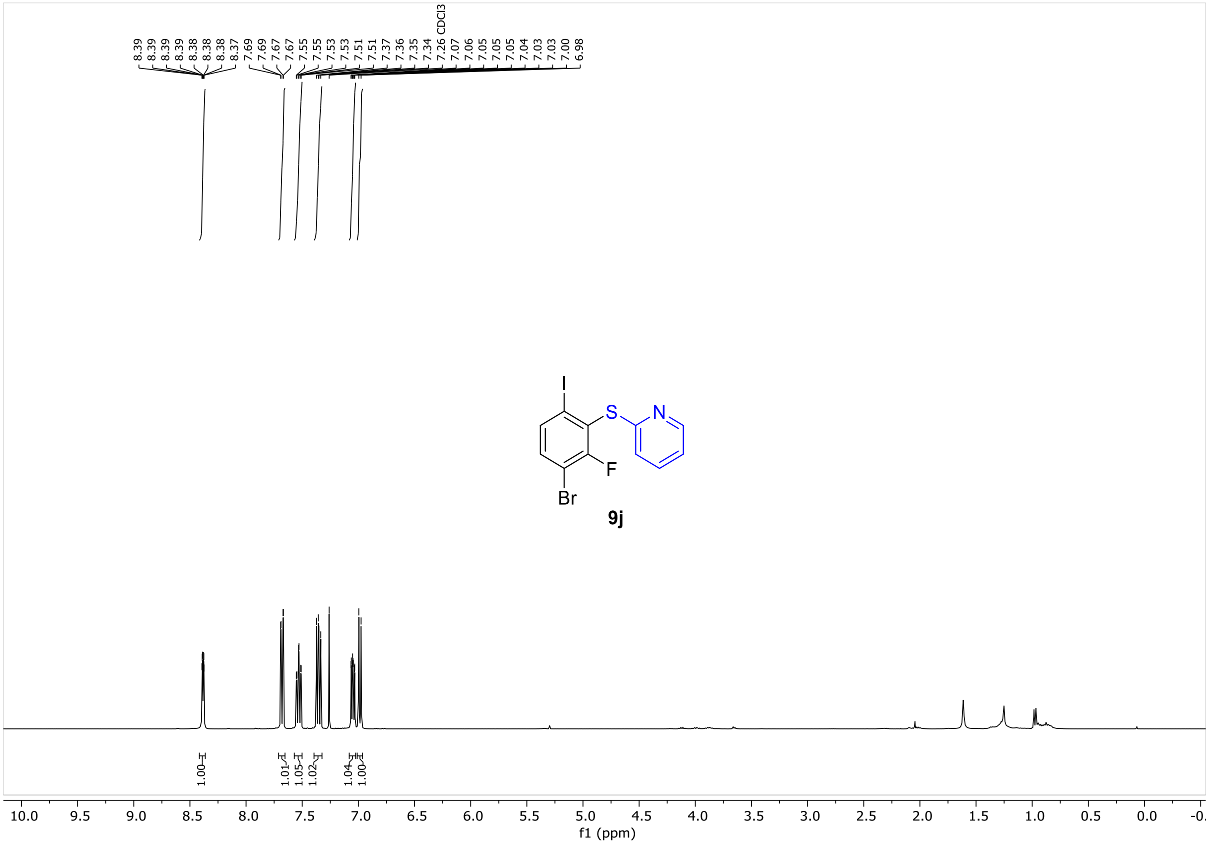
8.39
8.39
8.39
8.39
8.38
8.38
8.38
8.37
7.69
7.69
7.67
7.67
7.55
7.55
7.53
7.53
7.51
7.51
7.37
7.36
7.35
7.34
7.26 CDCl3
7.07
7.06
7.05
7.05
7.05
7.04
7.03
7.03
7.00
6.98
9j
1.00
1.01
1.05
1.02
1.04
1.00
f1 (ppm)

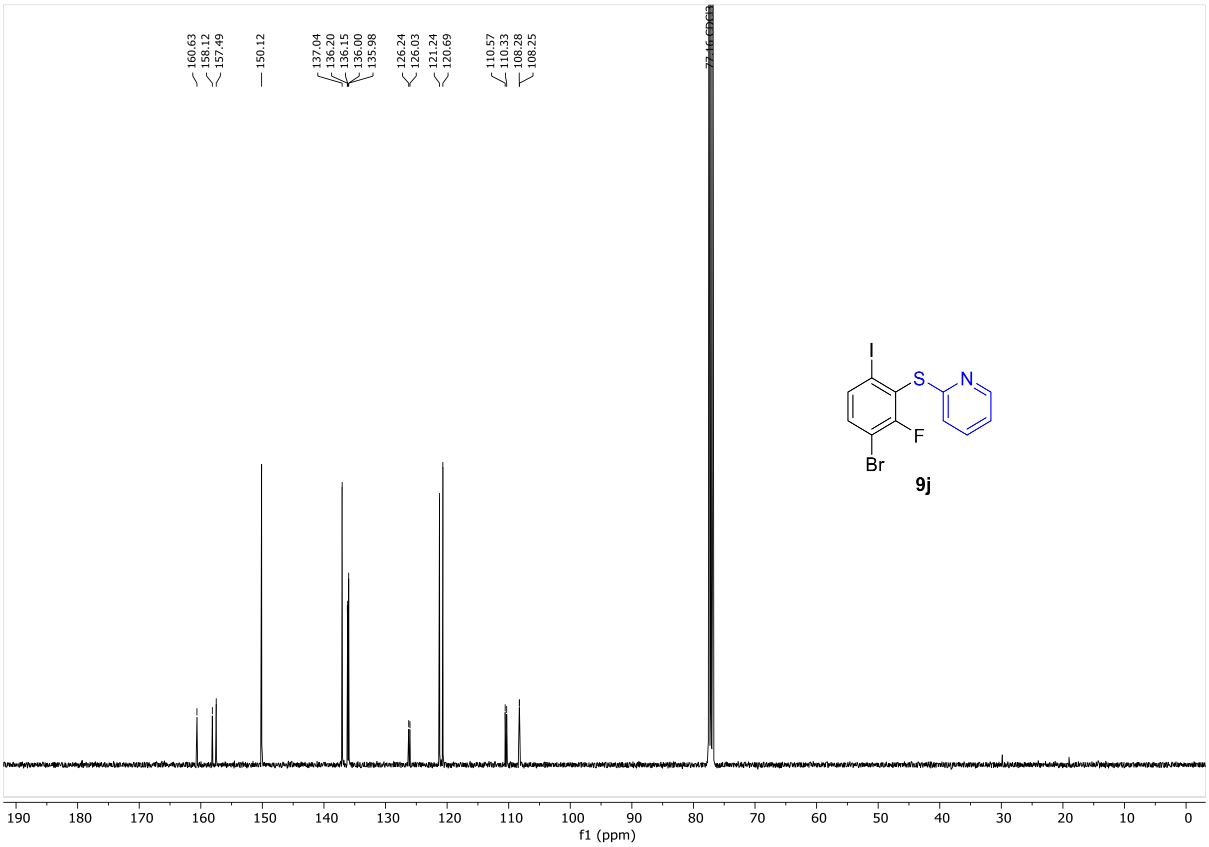
160.63
158.12
157.49
150.12
137.04
136.20
136.15
136.00
135.98
126.24
126.03
121.24
120.69
110.57
110.33
108.28
108.25
77.16 CDCl3
9j
f1 (ppm)

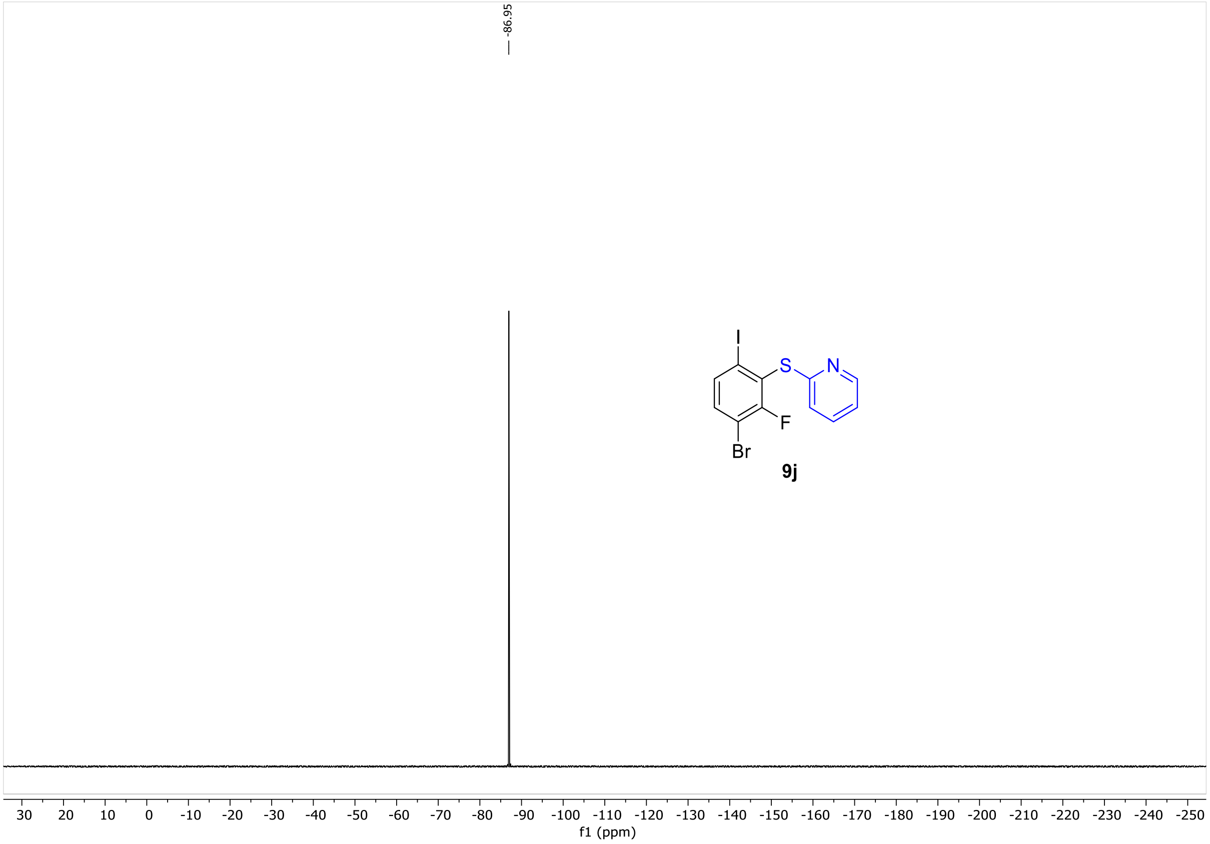
-86.95
I
S
N
F
Br
9j
30 20 10 0 -10 -20 -30 -40 -50 -60 -70 -80 -90 -100 -110 -120 -130 -140 -150 -160 -170 -180 -190 -200 -210 -220 -230 -240 -250
f1 (ppm)

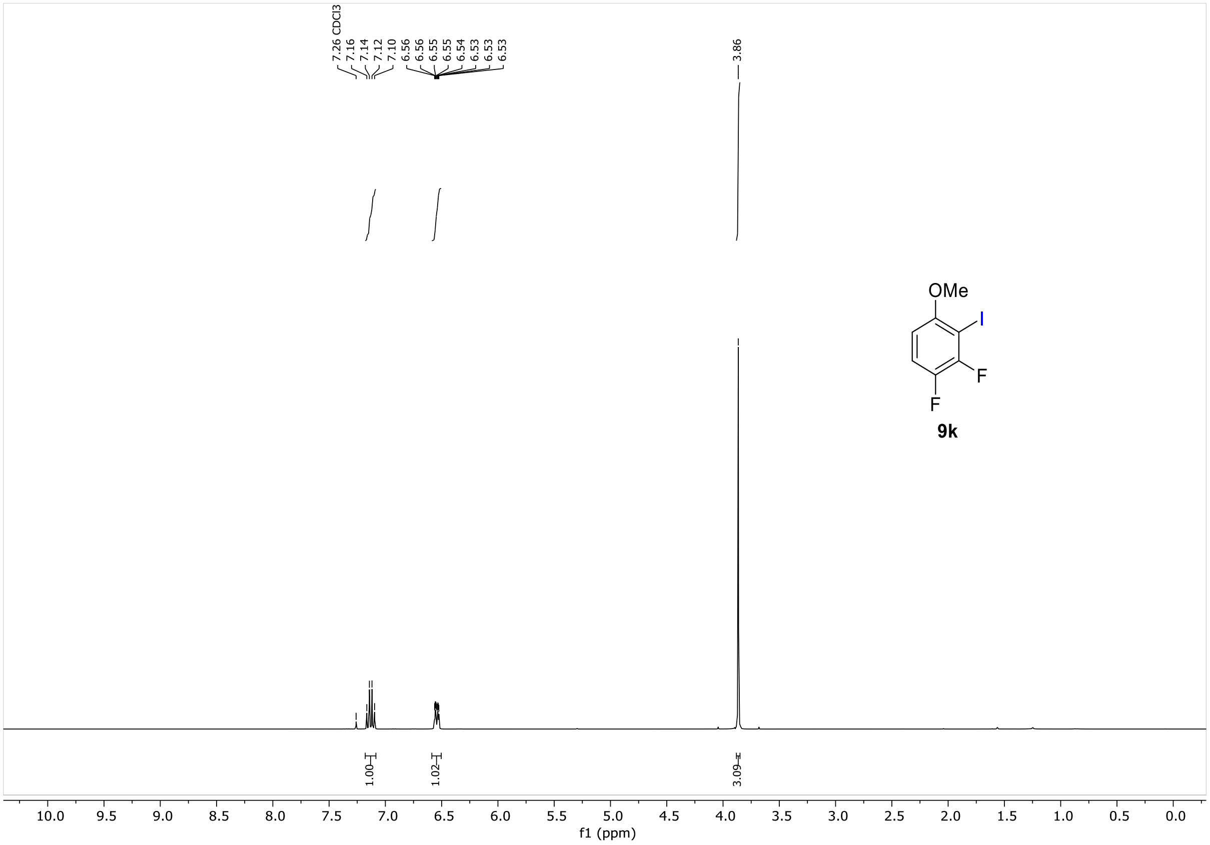
7.26 CDCl3
7.16
7.14
7.12
7.10
6.56
6.56
6.55
6.55
6.54
6.53
6.53
6.53
3.86
OMe
I
F
F
9k
1.00
1.02
3.09
10.0 9.5 9.0 8.5 8.0 7.5 7.0 6.5 6.0 5.5 5.0 4.5 4.0 3.5 3.0 2.5 2.0 1.5 1.0 0.5 0.0
f1 (ppm)

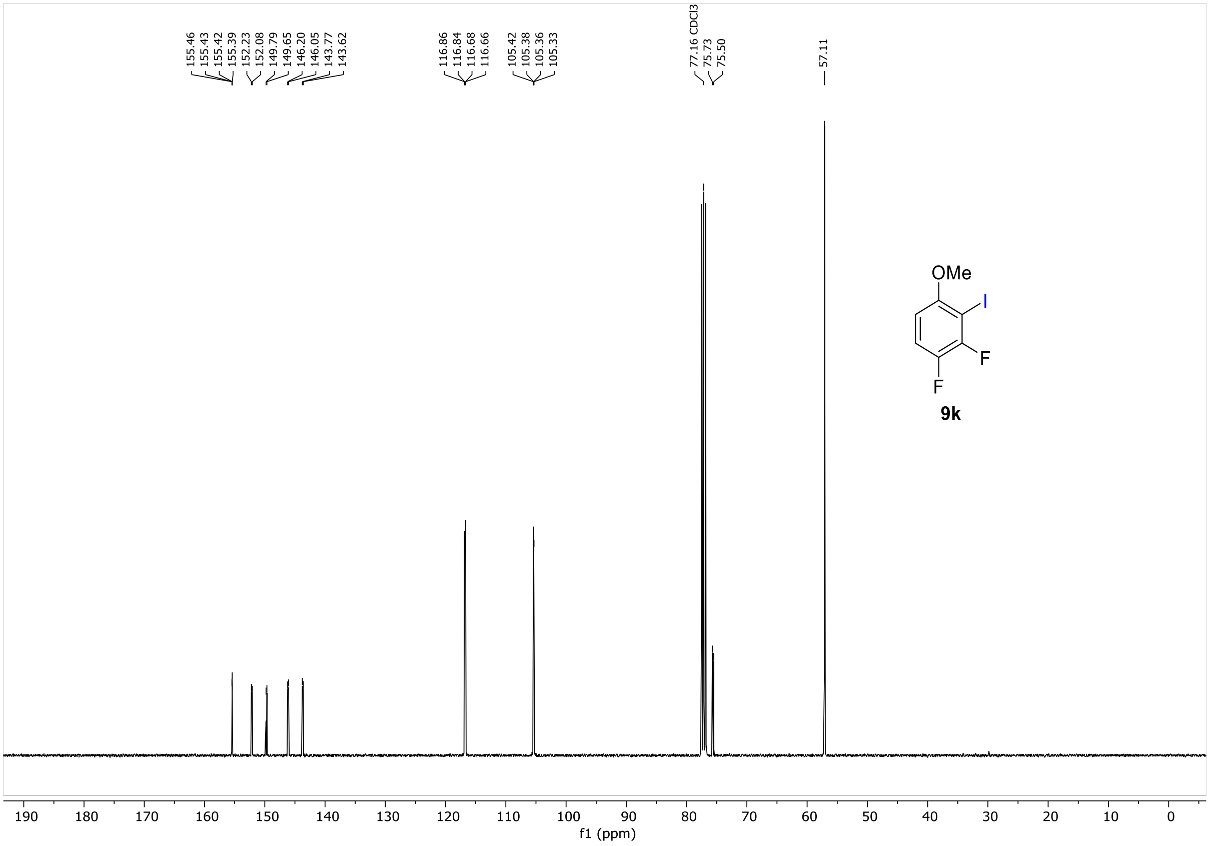
155.46
155.43
155.42
155.39
152.23
152.08
149.79
149.65
146.20
146.05
143.77
143.62
116.86
116.84
116.68
116.66
105.42
105.38
105.36
105.33
77.16 CDCl3
75.73
75.50
57.11
OMe
I
F
F
9k
f1 (ppm)

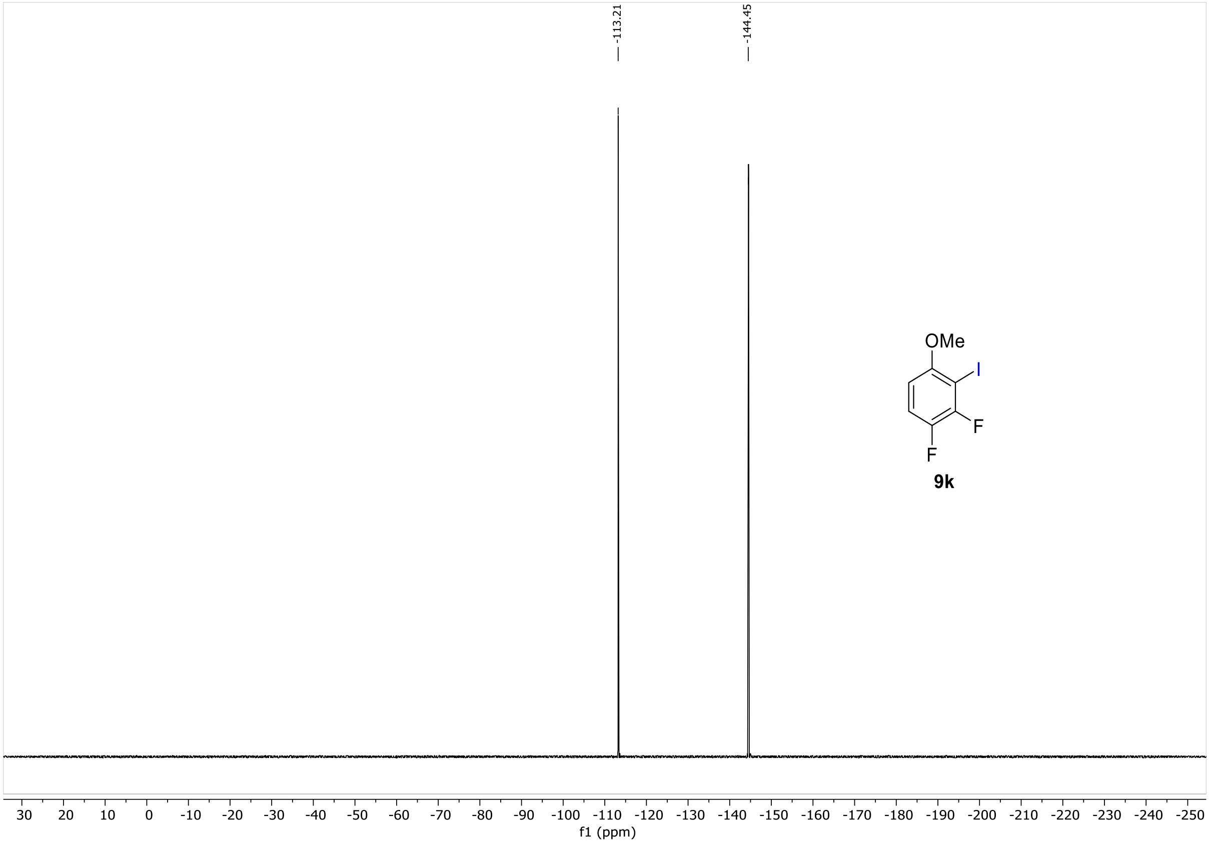
-113.21
-144.45
OMe
I
F
F
9k
f1 (ppm)

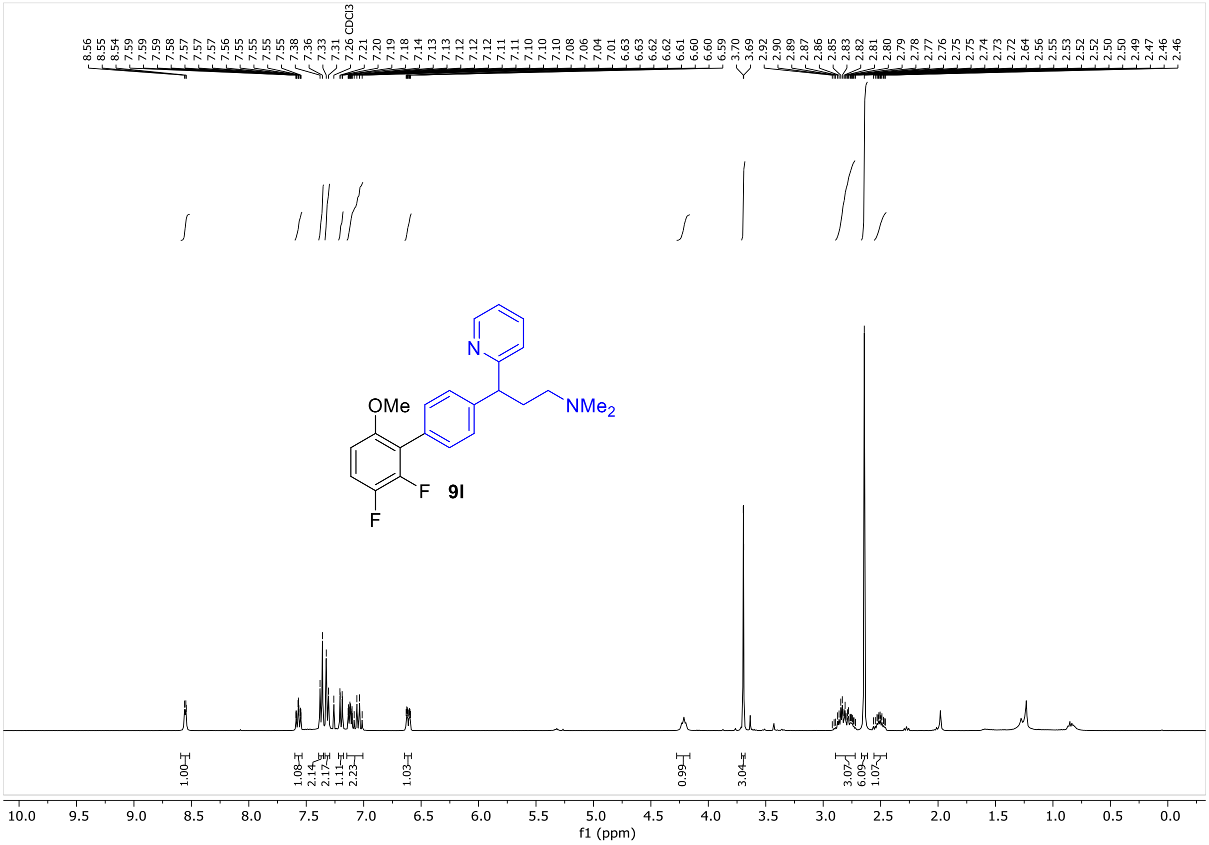
8.56 8.55 8.54 7.59 7.59 7.59 7.58 7.57 7.57 7.57 7.56 7.55 7.55 7.55 7.55 7.55 7.38 7.36 7.33 7.31 7.26 CDCl3 7.21 7.20 7.19 7.18 7.14 7.13 7.13 7.12 7.12 7.12 7.11 7.11 7.10 7.10 7.10 7.08 7.06 7.04 7.01 6.63 6.63 6.62 6.61 6.60 6.60 6.59 3.70 3.69 2.92 2.90 2.89 2.87 2.86 2.85 2.83 2.82 2.81 2.80 2.79 2.78 2.77 2.76 2.75 2.75 2.74 2.73 2.72 2.64 2.56 2.55 2.53 2.52 2.52 2.50 2.49 2.47 2.46 2.46
OMe
N
NMe2
F
F
9l
1.00 1.08 2.14 2.17 1.11 2.23 1.03 0.99 3.04 3.07 6.09 1.07
10.0 9.5 9.0 8.5 8.0 7.5 7.0 6.5 6.0 5.5 5.0 4.5 4.0 3.5 3.0 2.5 2.0 1.5 1.0 0.5 0.0
f1 (ppm)

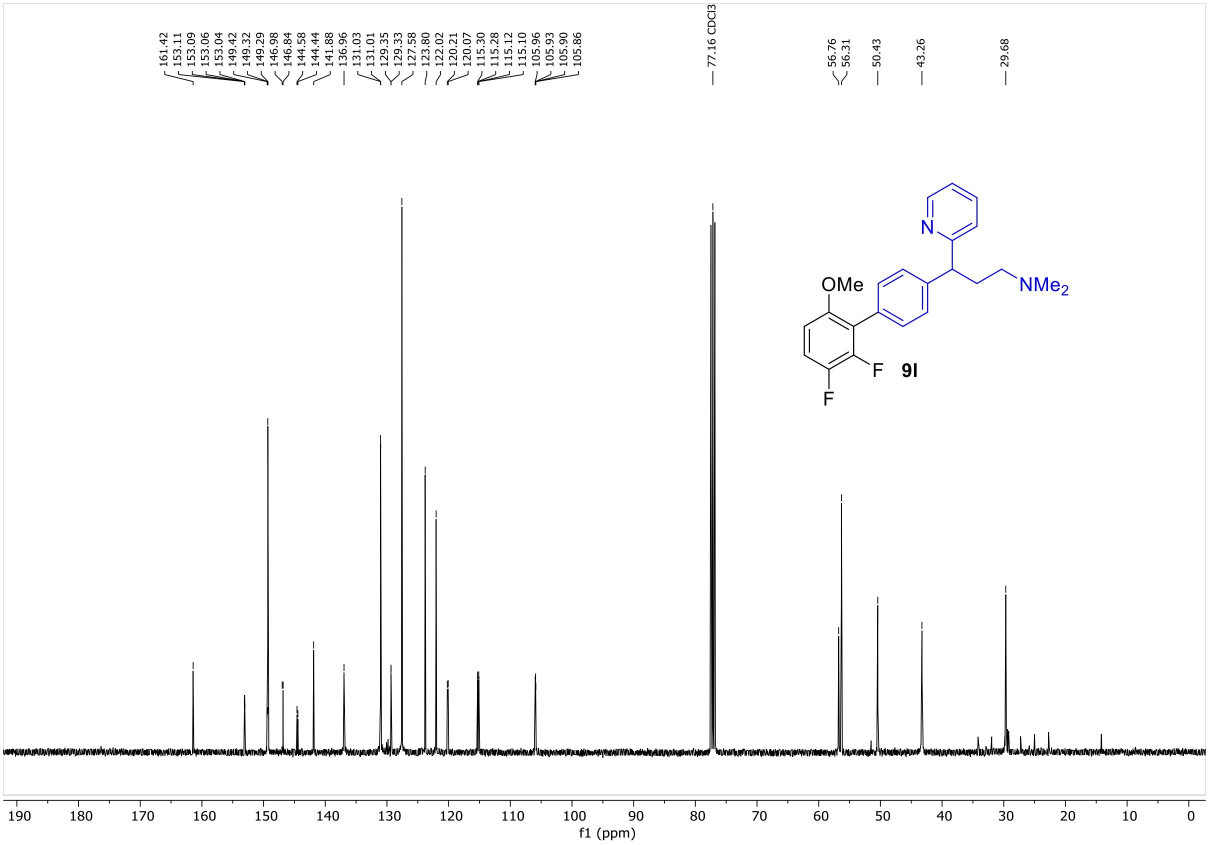
161.42 153.11 153.09 153.06 153.04 149.42 149.32 149.29 146.98 146.84 144.58 144.44 141.88 136.96 131.03 131.01 129.35 129.33 127.58 123.80 122.02 120.21 120.07 115.30 115.28 115.12 115.10 105.96 105.93 105.90 105.86
77.16 CDCl3
56.76 56.31 50.43 43.26 29.68
OMe
N
NMe2
F
F
9l
190 180 170 160 150 140 130 120 110 100 90 80 70 60 50 40 30 20 10 0
f1 (ppm)

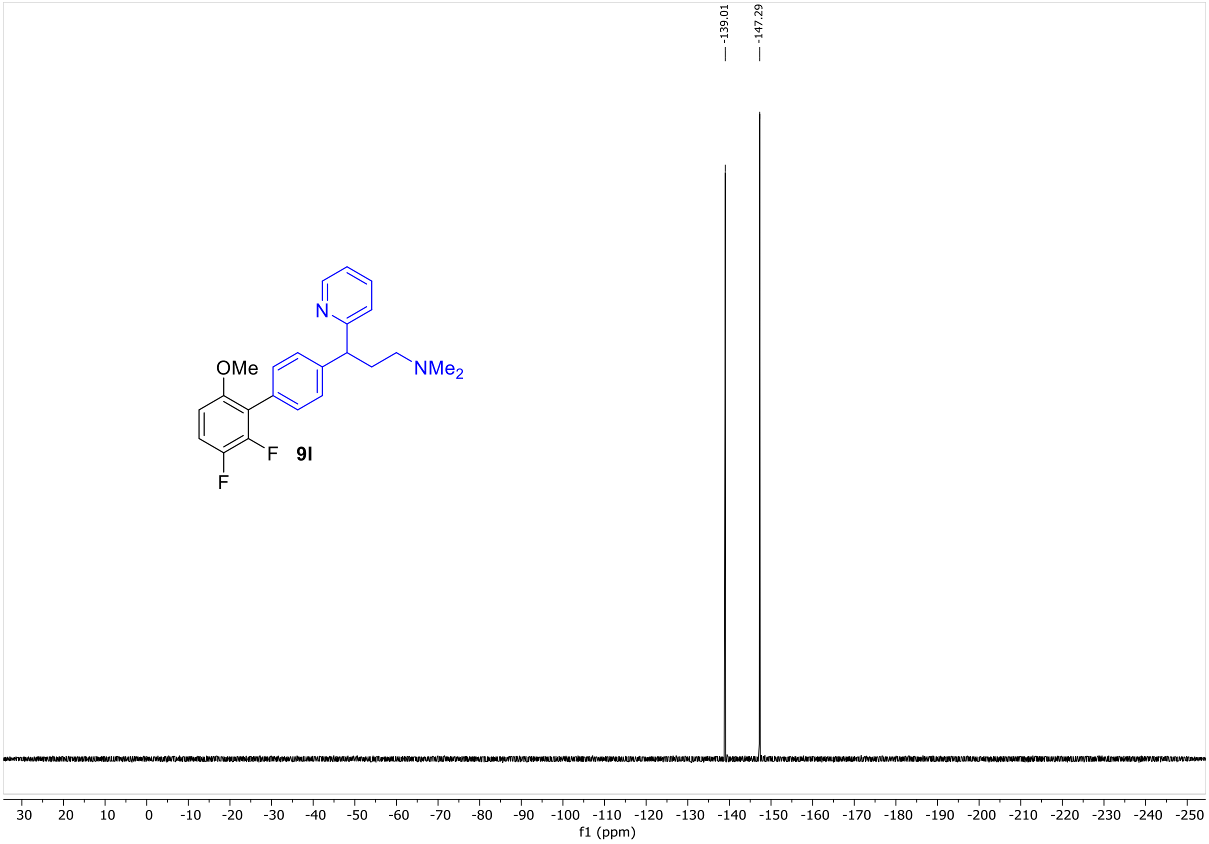
-139.01
-147.29
OMe
N
NMe2
F
F
9l
f1 (ppm)

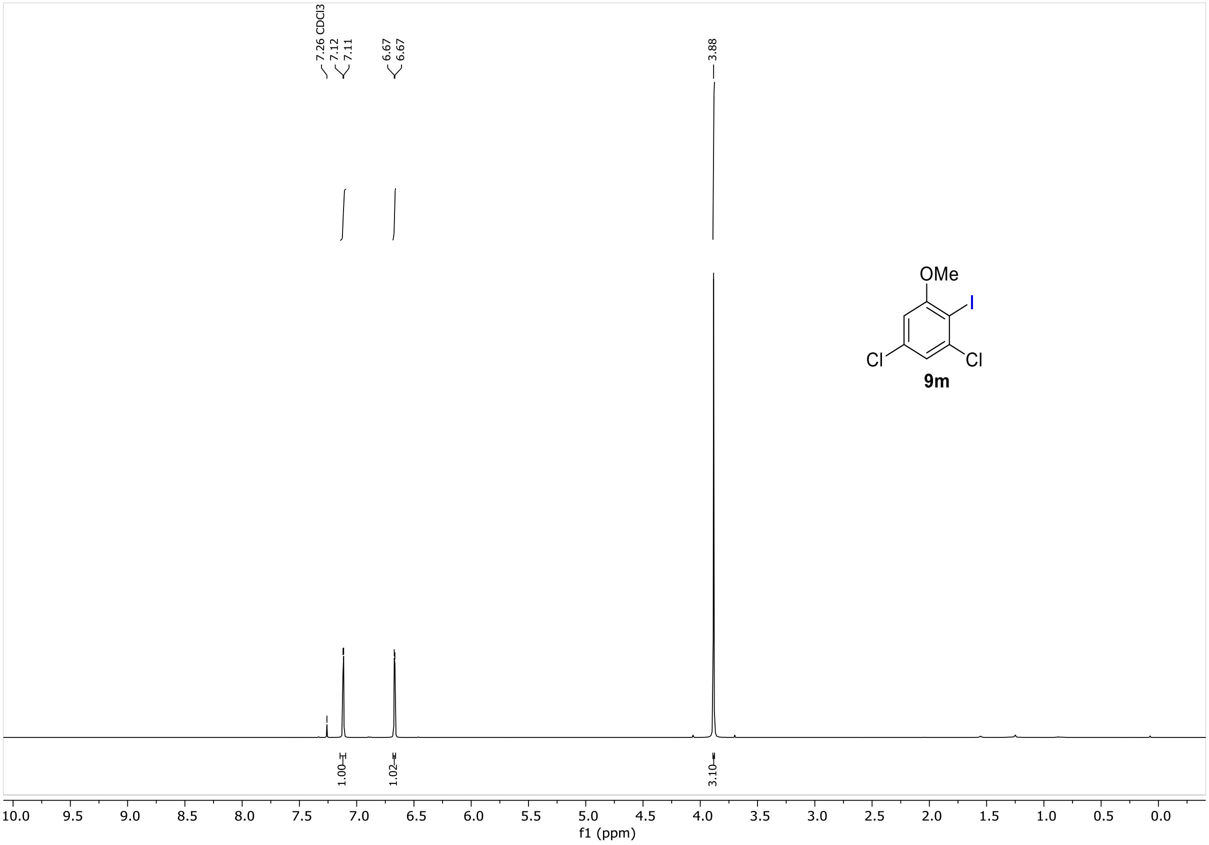
7.26 CDCl3
7.12
7.11
6.67
6.67
3.88
OMe
I
Cl
Cl
9m
1.00
1.02
3.10
f1 (ppm)

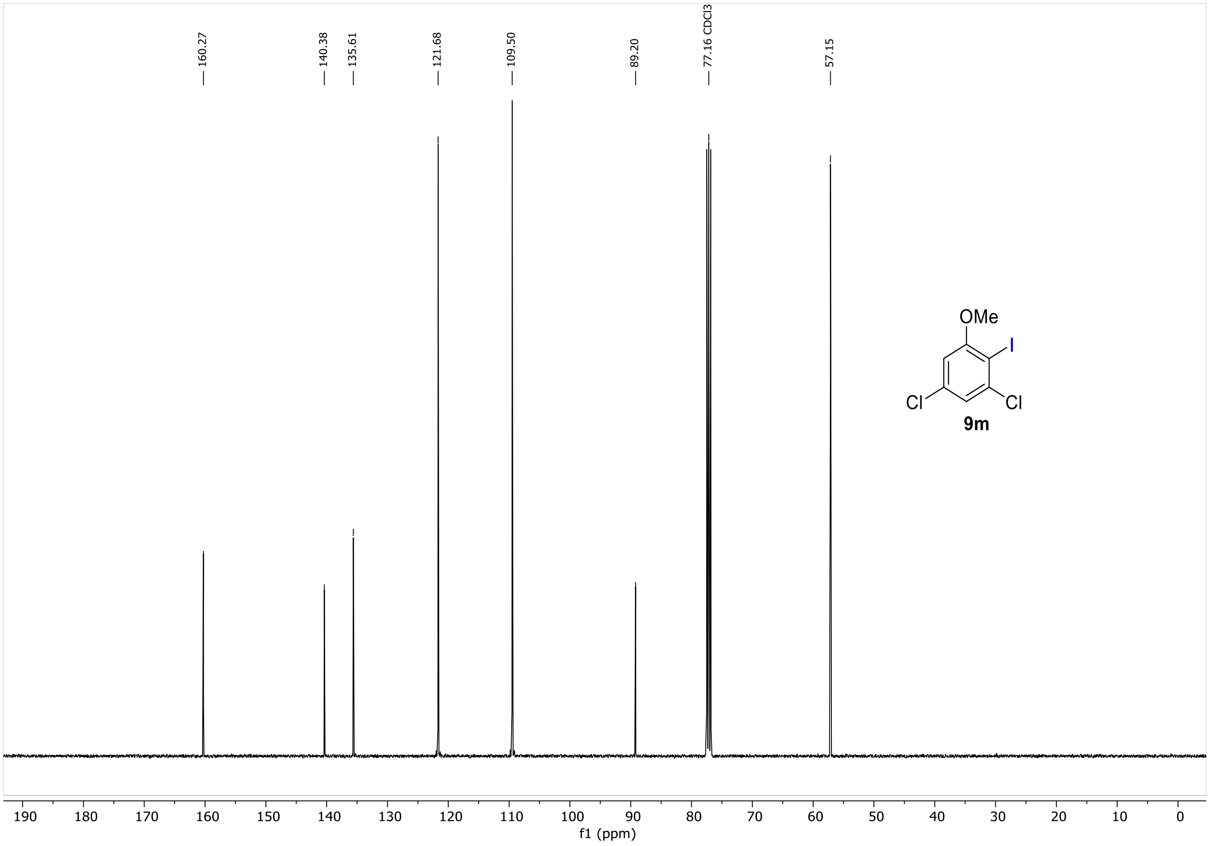
160.27
140.38
135.61
121.68
109.50
89.20
77.16 CDCl3
57.15
OMe
I
Cl
Cl
9m
f1 (ppm)

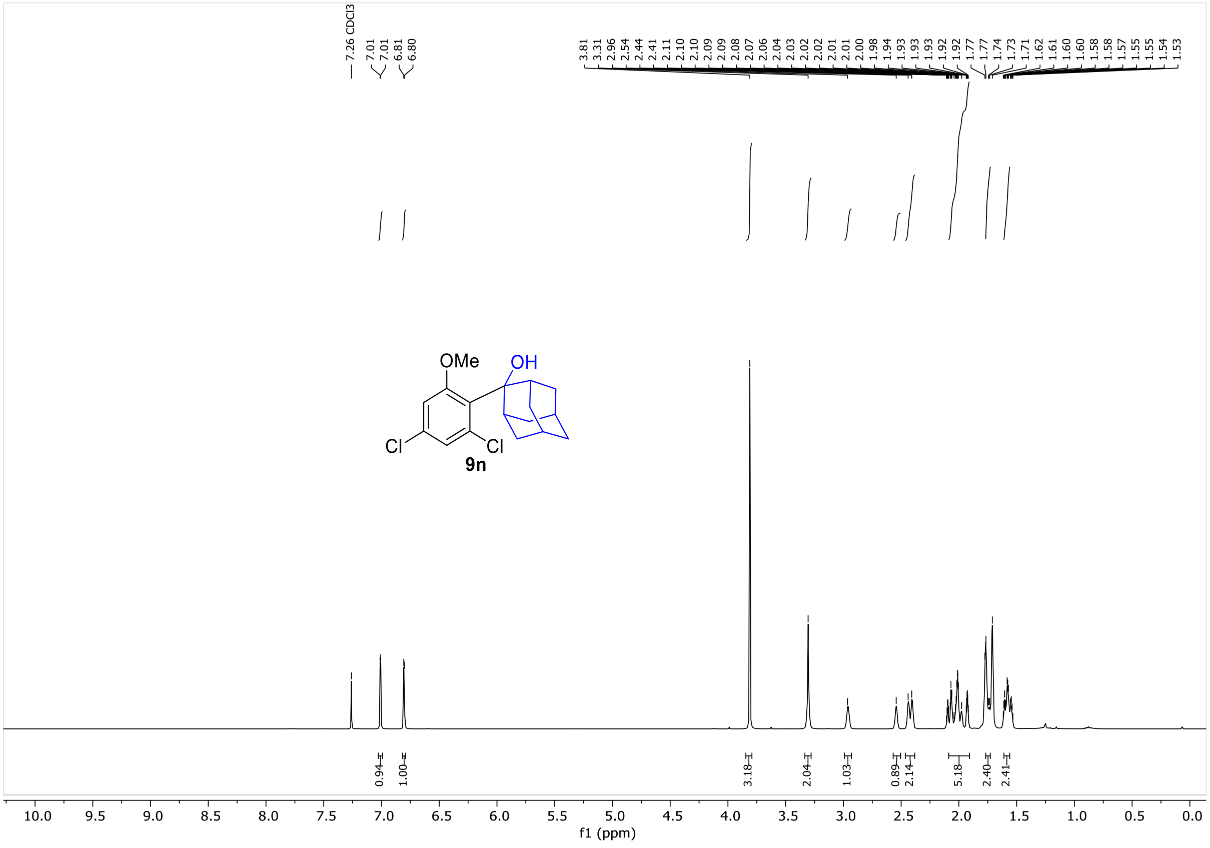
7.26 CDCl3
7.01
7.01
6.81
6.80
OMe
OH
Cl
Cl
9n
0.94
1.00
3.18
2.04
1.03
0.89
2.14
5.18
2.40
2.41
f1 (ppm)

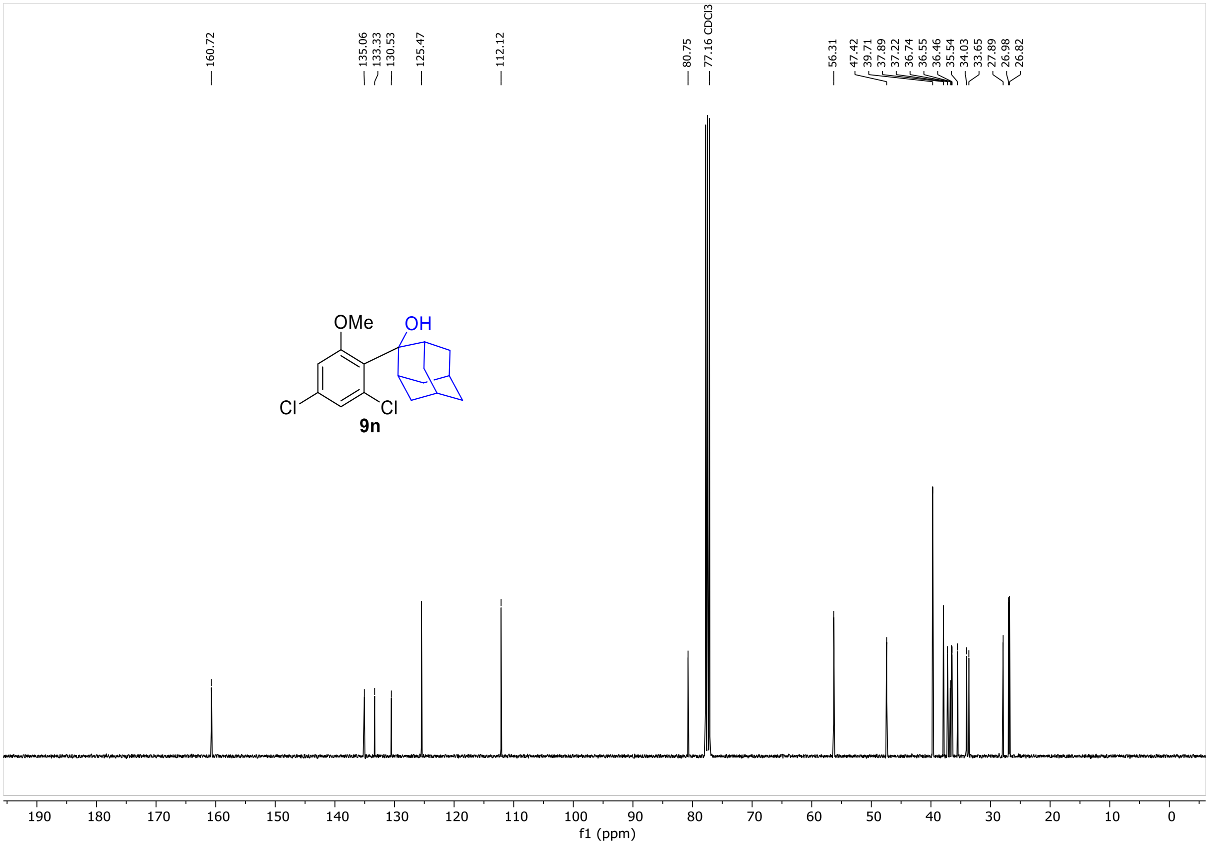
160.72
135.06
133.33
130.53
125.47
112.12
80.75
77.16 CDCl3
56.31
47.42
39.71
37.89
37.22
36.74
36.55
36.46
35.54
34.03
33.65
27.89
26.98
26.82
OMe
OH
Cl
Cl
9n
f1 (ppm)

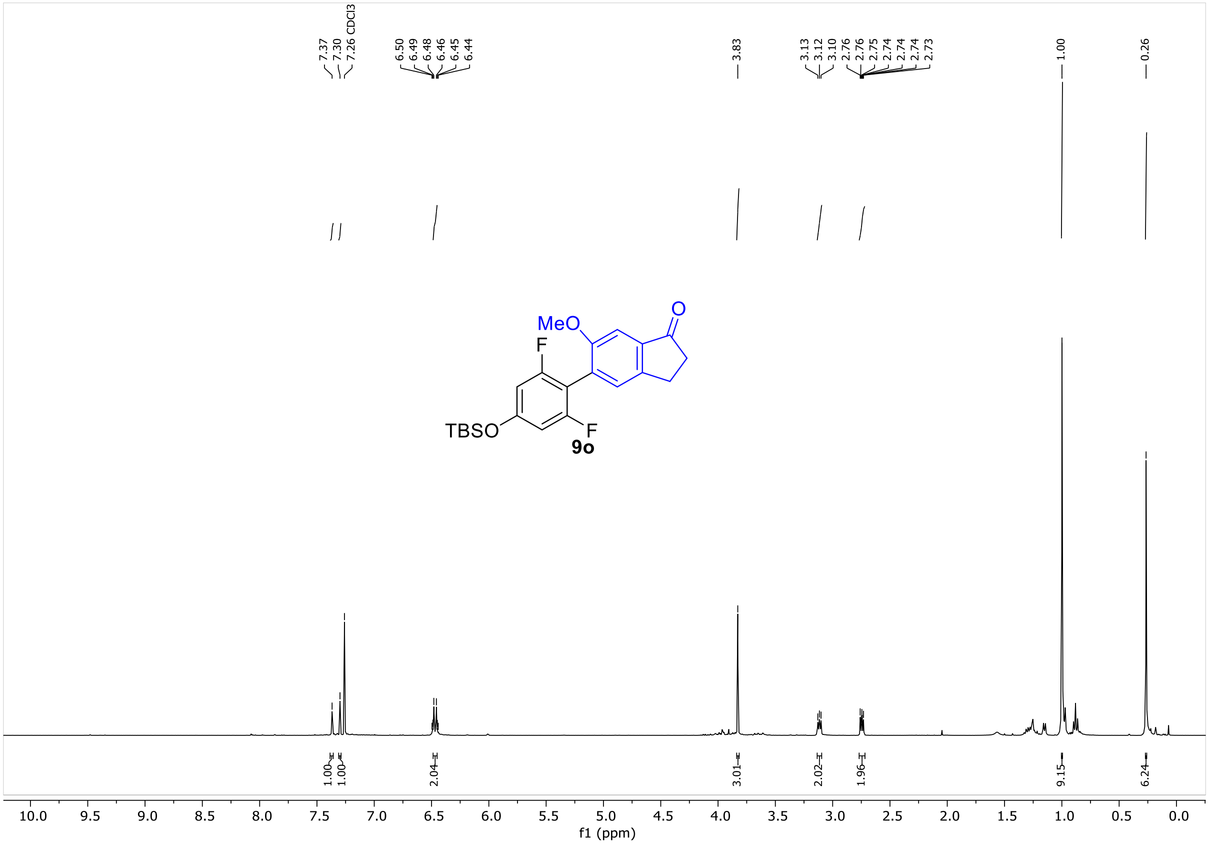
7.37
7.30
7.26 CDCl3
6.50
6.49
6.48
6.46
6.45
6.44
3.83
3.13
3.12
3.10
2.76
2.75
2.74
2.74
2.74
2.73
1.00
0.26
MeO
O
F
F
TBSO
9o
1.00
1.00
2.04
3.01
2.02
1.96
9.15
6.24
f1 (ppm)

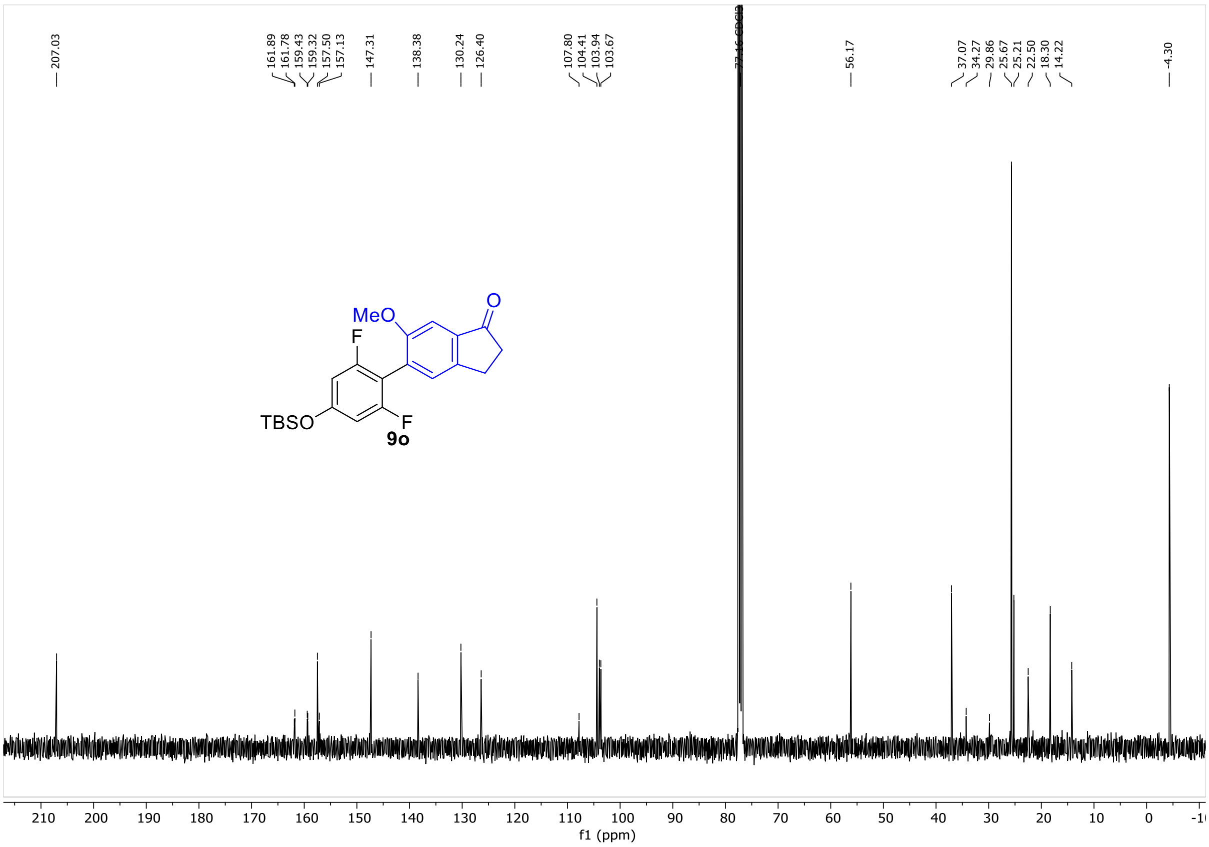
207.03
161.89
161.78
159.43
159.32
157.50
157.13
147.31
138.38
130.24
126.40
107.80
104.41
103.94
103.67
56.17
37.07
34.27
29.86
25.67
25.21
22.50
18.30
14.22
-4.30
MeO
F
TBSO
F
9o
f1 (ppm)

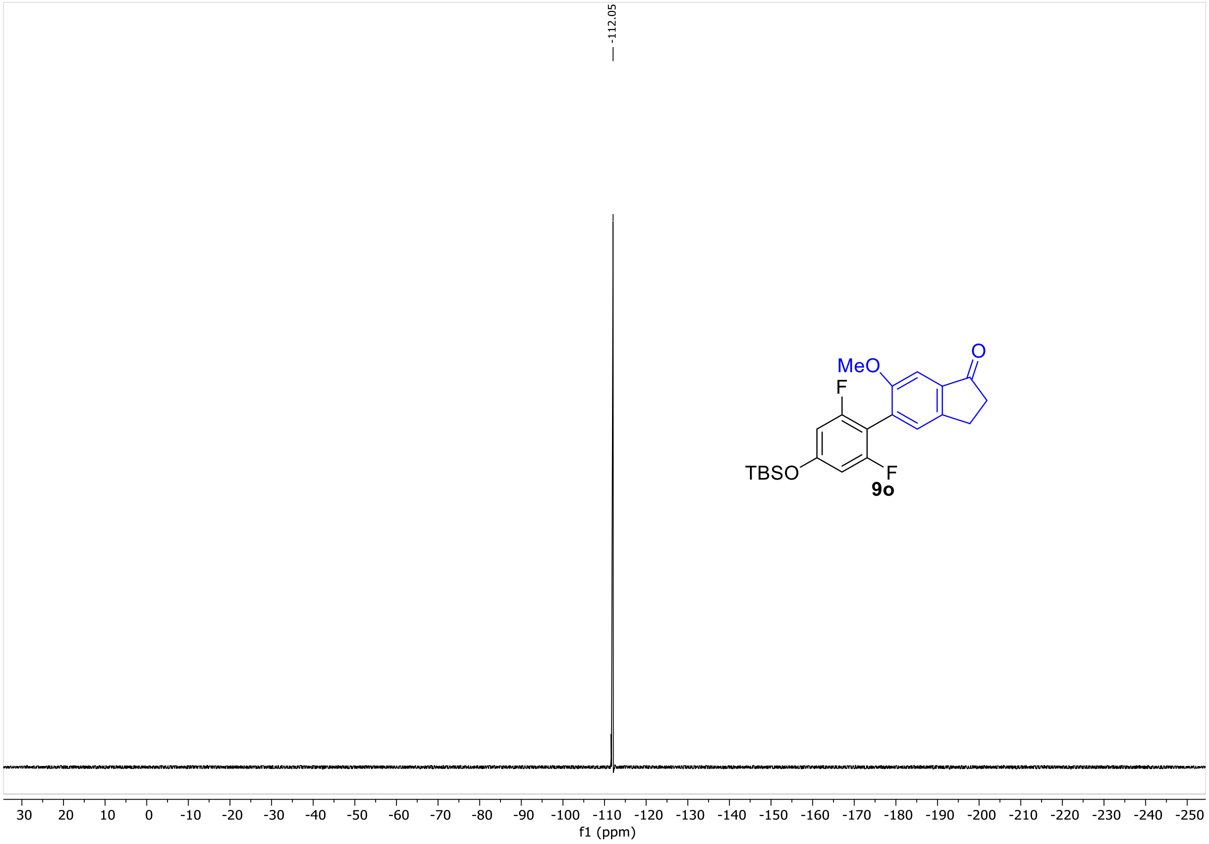
-112.05
MeO
F
TBSO
F
9o
f1 (ppm)

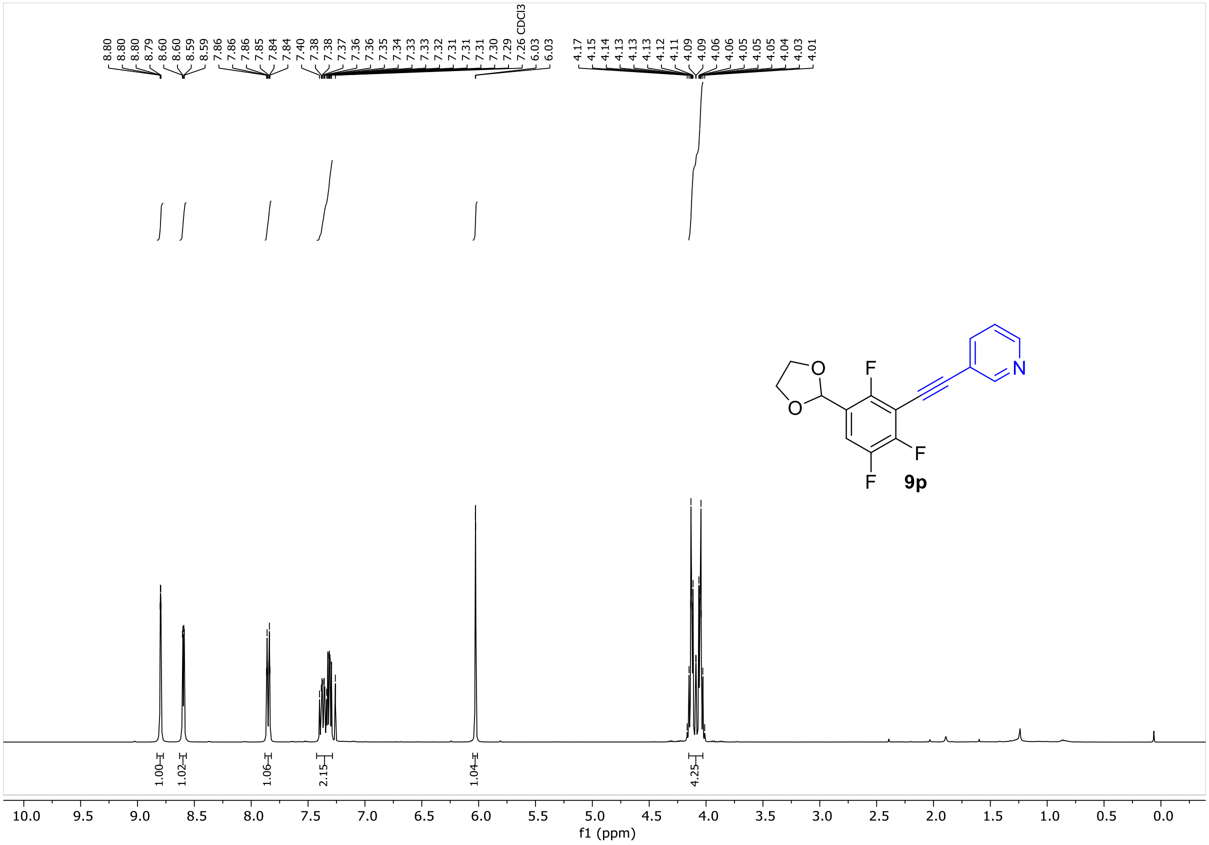
8.80 8.80 8.80 8.79 8.60 8.60 8.59 8.59 7.86 7.86 7.85 7.84 7.84 7.40 7.38 7.38 7.37 7.36 7.36 7.35 7.34 7.33 7.33 7.32 7.31 7.31 7.31 7.30 7.29 7.26 CDCl3 6.03 6.03
4.17 4.15 4.14 4.13 4.13 4.13 4.12 4.11 4.09 4.09 4.06 4.05 4.05 4.05 4.04 4.03 4.01
O
O
F
F
F
N
9p
1.00
1.02
1.06
2.15
1.04
4.25
10.0 9.5 9.0 8.5 8.0 7.5 7.0 6.5 6.0 5.5 5.0 4.5 4.0 3.5 3.0 2.5 2.0 1.5 1.0 0.5 0.0
f1 (ppm)

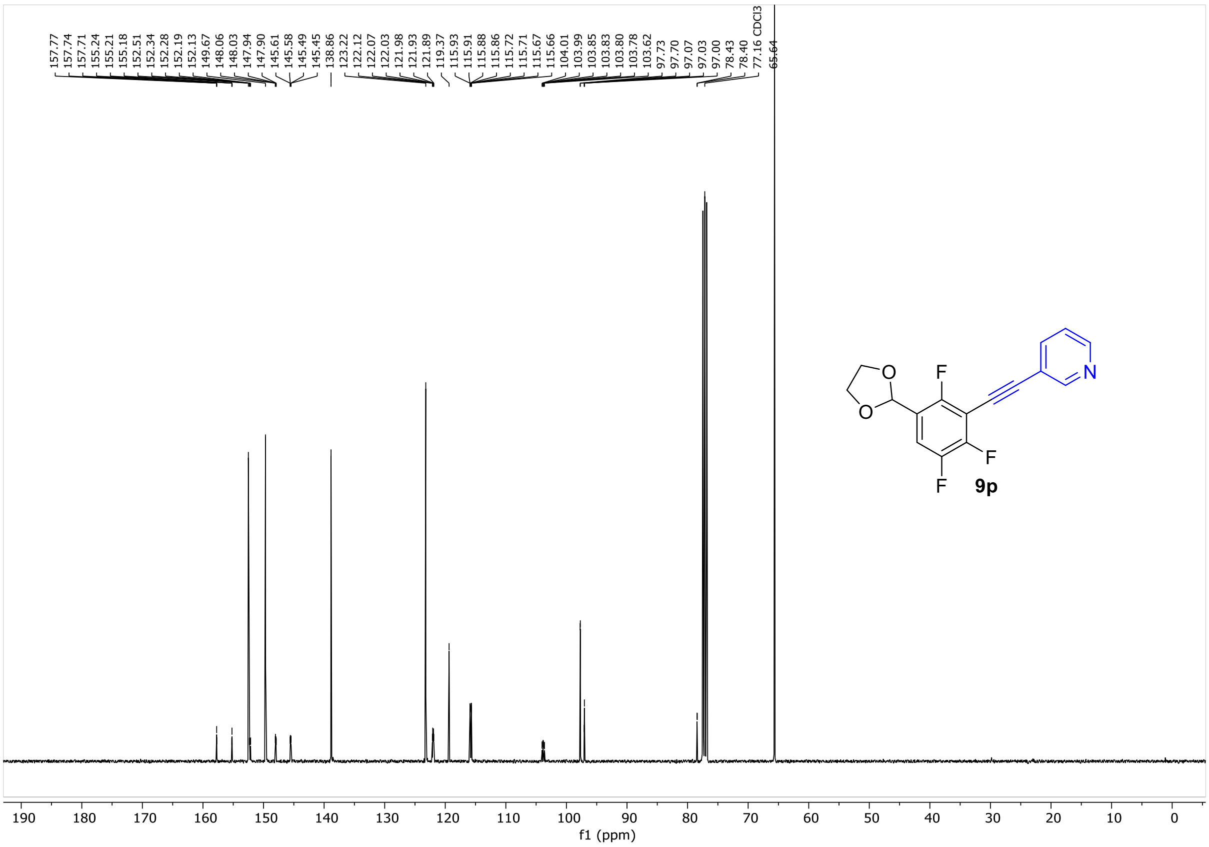
157.77 157.74 157.71 155.24 155.21 155.18 152.51 152.34 152.28 152.19 152.13 149.67 148.06 148.03 147.94 147.90 145.61 145.58 145.49 145.45 138.86 123.22 122.12 122.07 122.03 121.98 121.93 121.89 119.37 115.93 115.91 115.88 115.86 115.72 115.71 115.67 115.66 104.01 103.99 103.85 103.83 103.80 103.78 103.62 97.73 97.70 97.07 97.03 97.00 78.43 78.40 77.16 CDCl3 65.64
O
O
F
F
F
N
9p
190 180 170 160 150 140 130 120 110 100 90 80 70 60 50 40 30 20 10 0
f1 (ppm)

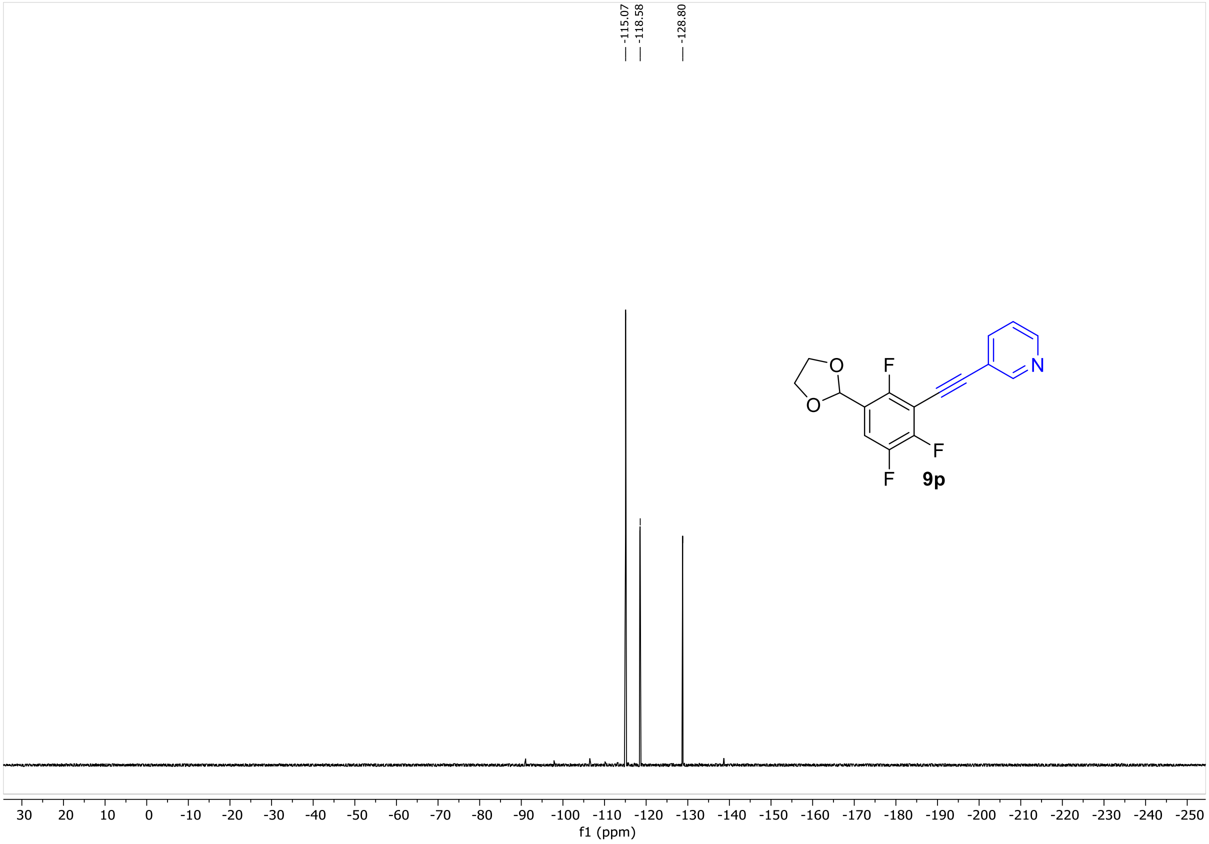
-115.07
-118.58
-128.80
O
O
F
F
F
N
9p
30 20 10 0 -10 -20 -30 -40 -50 -60 -70 -80 -90 -100 -110 -120 -130 -140 -150 -160 -170 -180 -190 -200 -210 -220 -230 -240 -250
f1 (ppm)

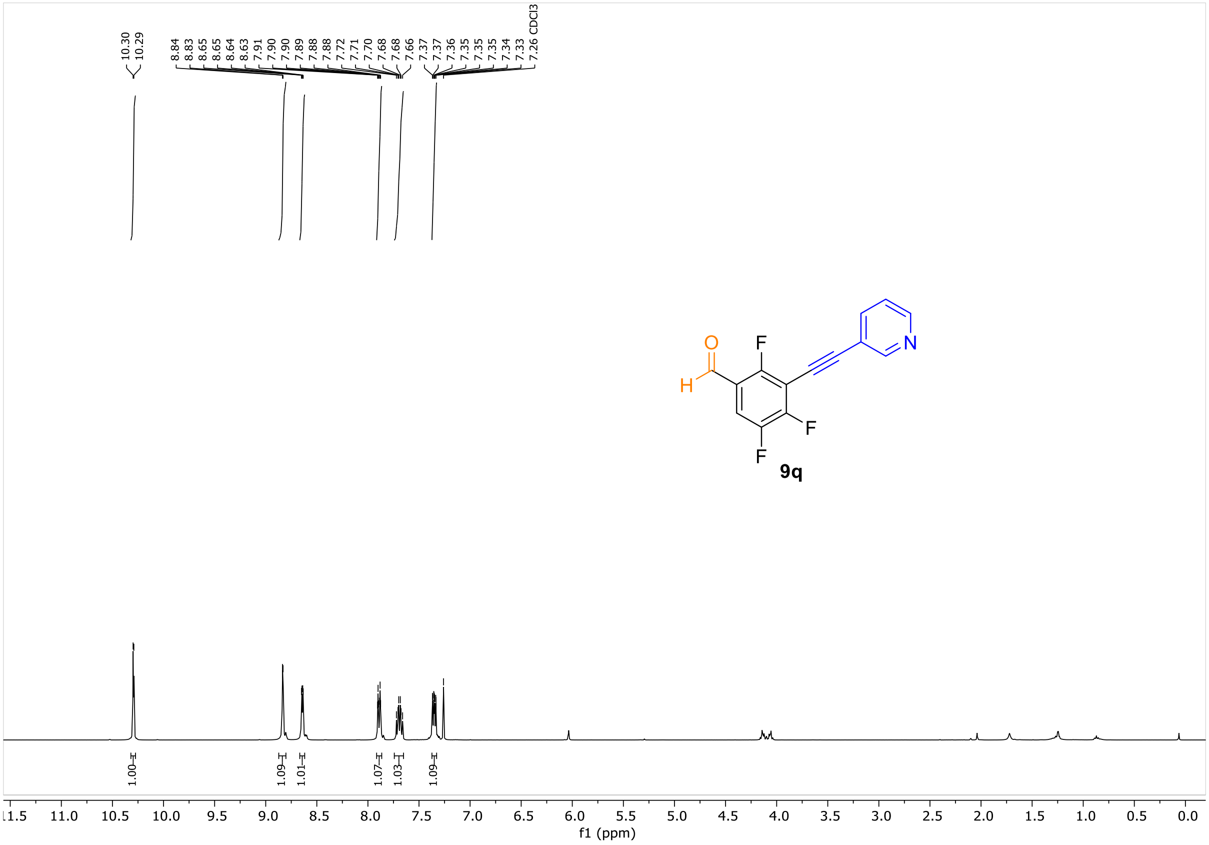
10.30
10.29
8.84
8.83
8.65
8.65
8.64
8.63
7.91
7.90
7.89
7.88
7.88
7.72
7.71
7.70
7.68
7.68
7.66
7.37
7.37
7.36
7.35
7.35
7.35
7.34
7.33
7.26 CDCl3
O
H
F
F
F
N
9q
1.00
1.09
1.01
1.07
1.03
1.09
11.5 11.0 10.5 10.0 9.5 9.0 8.5 8.0 7.5 7.0 6.5 6.0 5.5 5.0 4.5 4.0 3.5 3.0 2.5 2.0 1.5 1.0 0.5 0.0
f1 (ppm)

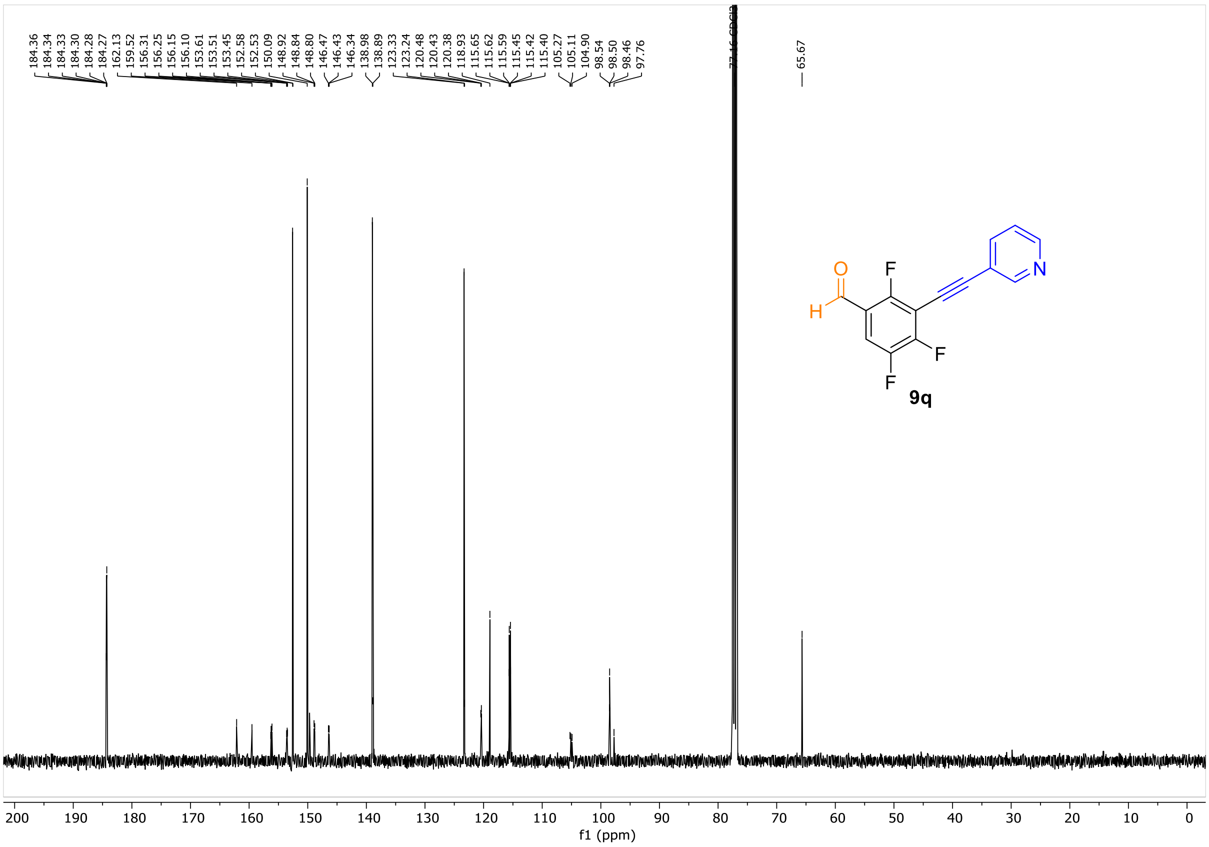
184.36
184.34
184.33
184.30
184.28
184.27
162.13
159.52
156.31
156.25
156.15
156.10
153.61
153.51
153.45
152.58
152.53
150.09
148.92
148.84
148.80
146.47
146.43
146.34
138.98
138.89
123.33
123.24
120.48
120.43
120.38
118.93
115.65
115.62
115.59
115.45
115.42
115.40
105.27
105.11
104.90
98.54
98.50
98.46
97.76
65.67
O
H
F
N
F
F
9q
f1 (ppm)

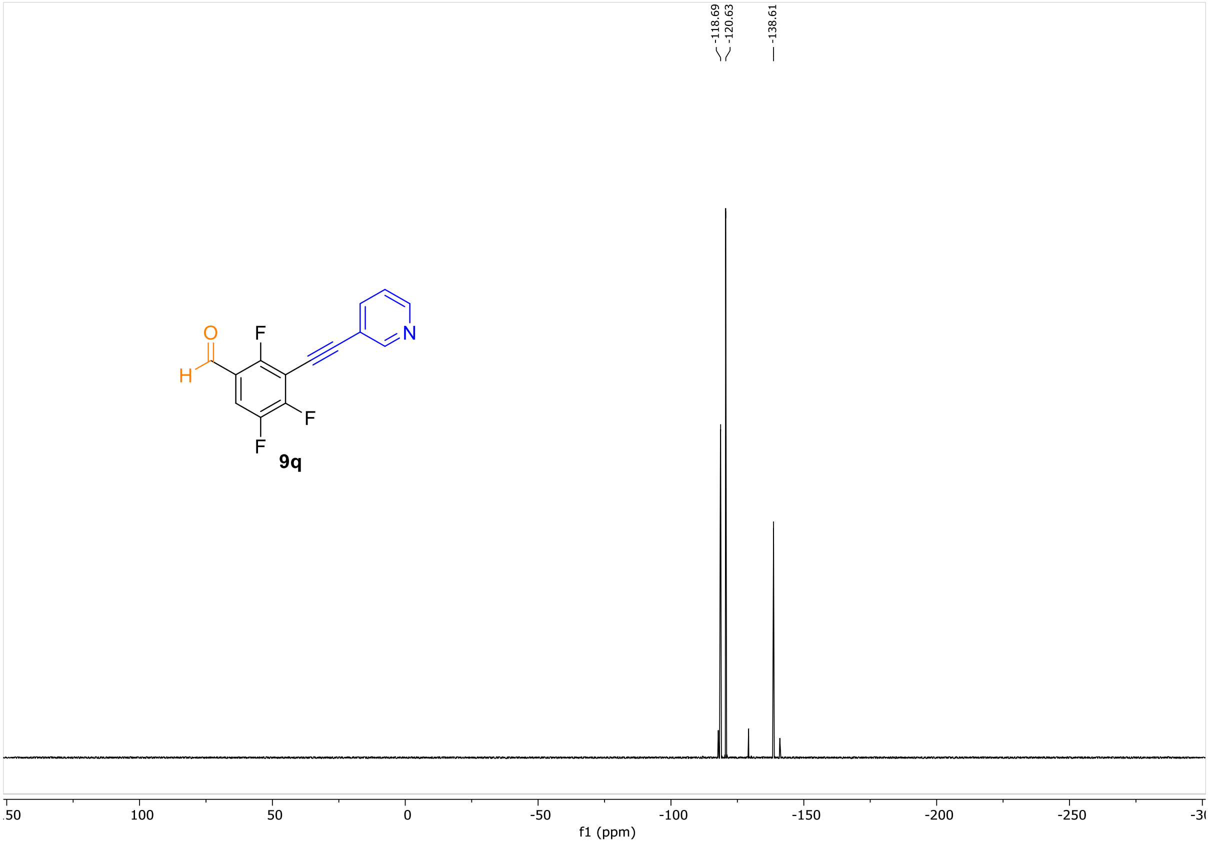
-118.69
-120.63
-138.61
O
H
F
N
F
F
9q
f1 (ppm)

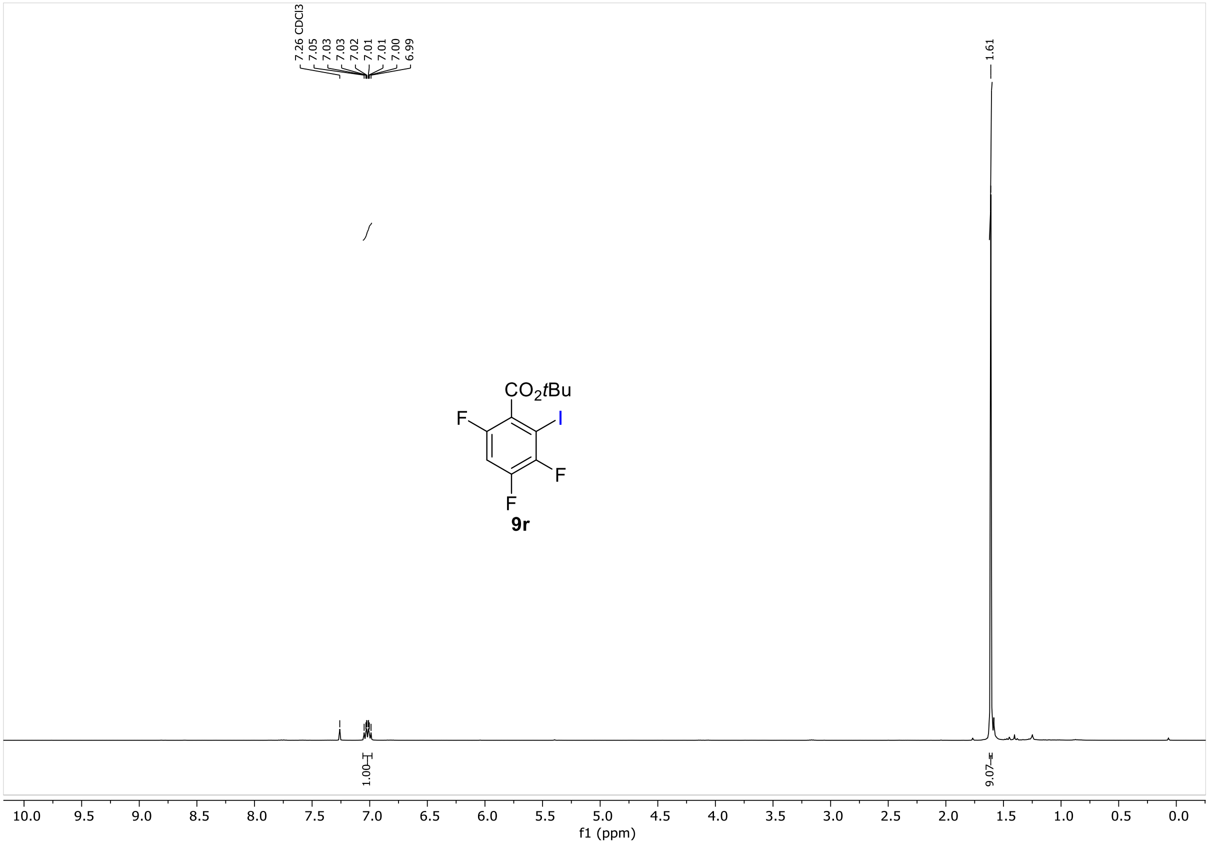
7.26 CDCl3
7.05
7.03
7.03
7.02
7.01
7.01
7.00
6.99
1.61
CO2tBu
F
I
F
F
9r
1.00
9.07
10.0 9.5 9.0 8.5 8.0 7.5 7.0 6.5 6.0 5.5 5.0 4.5 4.0 3.5 3.0 2.5 2.0 1.5 1.0 0.5 0.0
f1 (ppm)

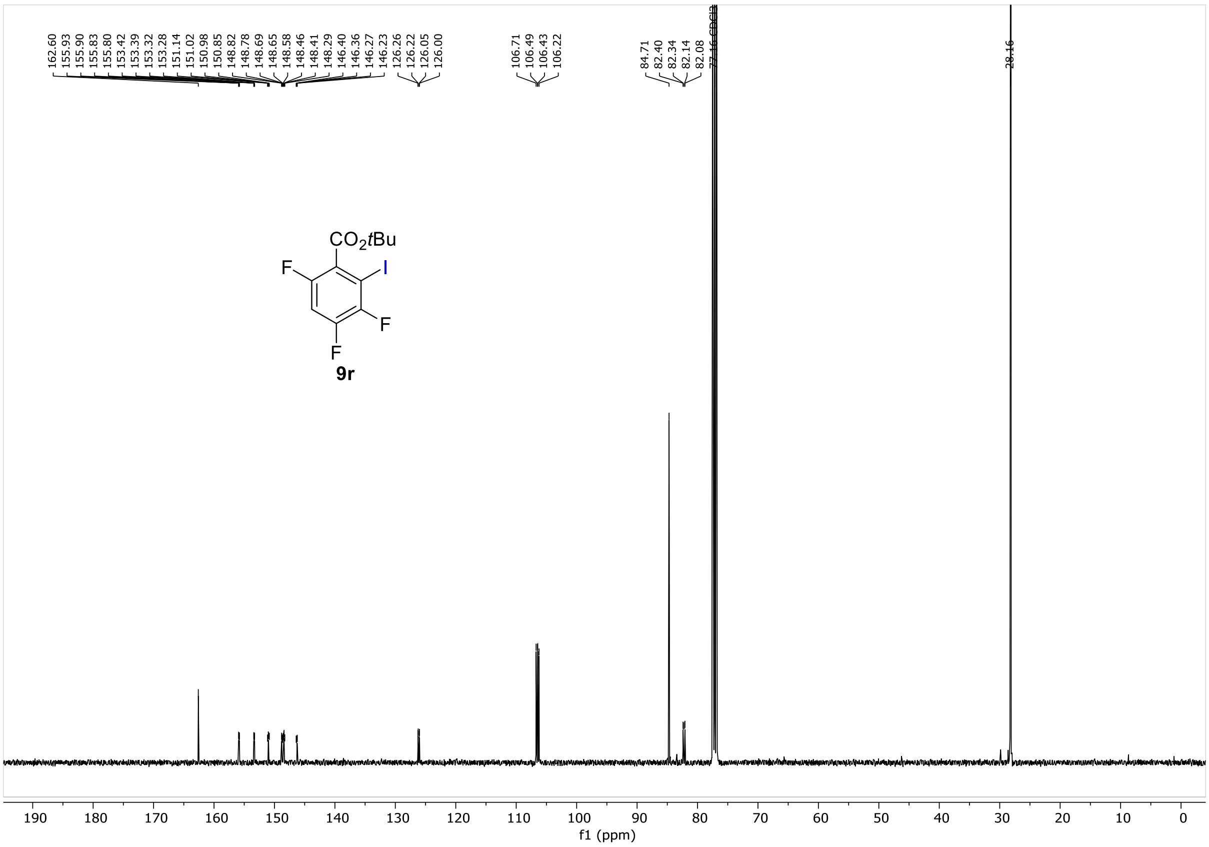
162.60
155.93
155.90
155.83
155.80
153.42
153.39
153.32
153.28
151.14
151.02
150.98
150.85
148.82
148.78
148.69
148.65
148.58
148.46
148.41
148.29
146.40
146.36
146.27
146.23
126.26
126.22
126.05
126.00
106.71
106.49
106.43
106.22
84.71
82.40
82.34
82.14
82.08
77.16 CDCl3
28.16
CO2tBu
F
I
F
F
9r
190 180 170 160 150 140 130 120 110 100 90 80 70 60 50 40 30 20 10 0
f1 (ppm)

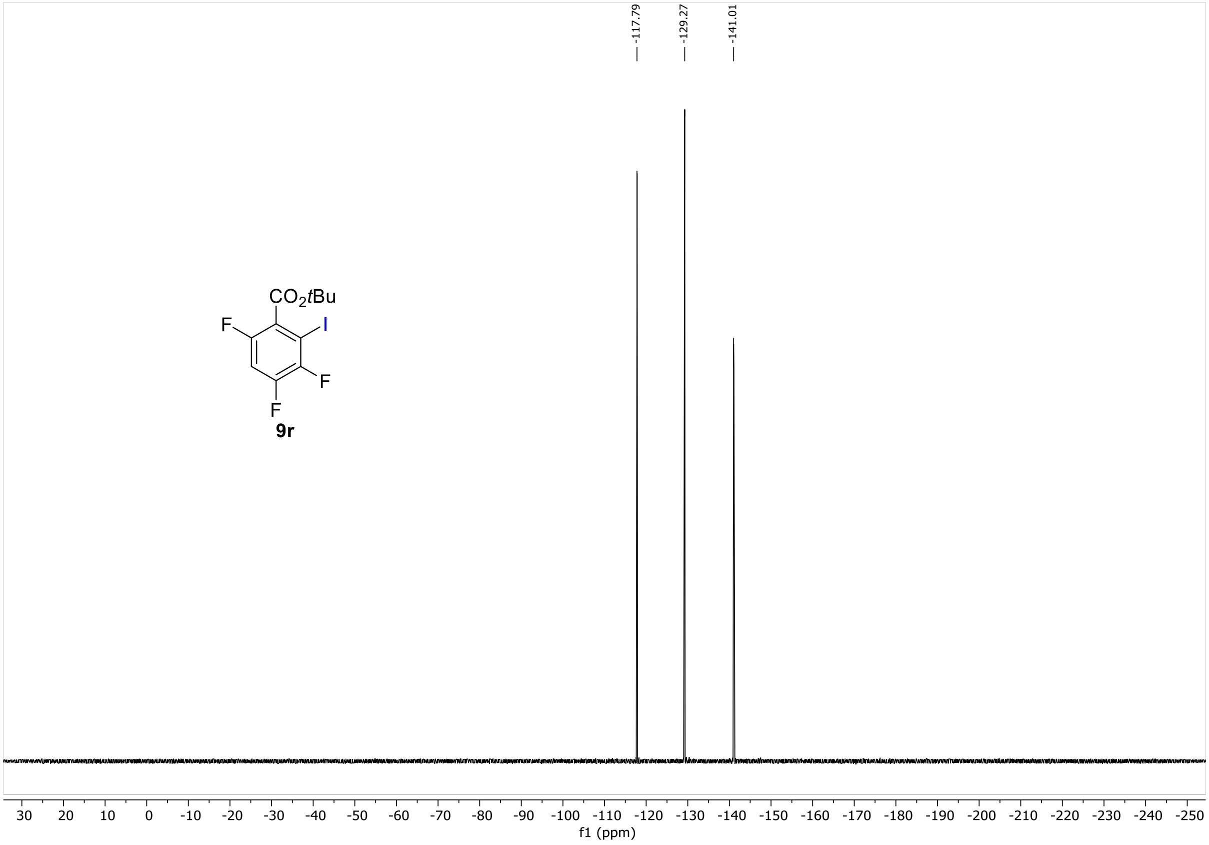
-117.79
-129.27
-141.01
CO_2tBu
F
I
F
F
9r
f1 (ppm)

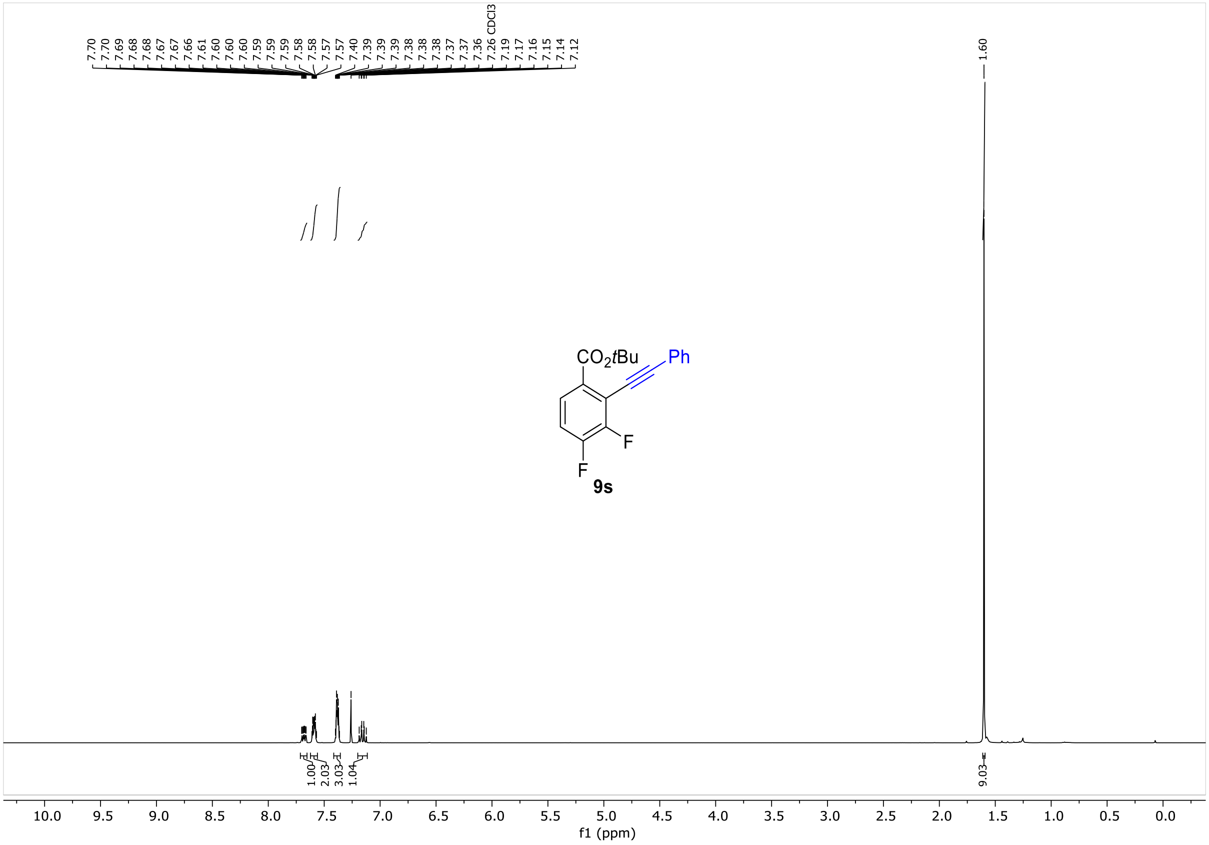
7.70 7.70 7.69 7.68 7.68 7.67 7.67 7.66 7.61 7.60 7.60 7.59 7.59 7.59 7.58 7.58 7.57 7.57 7.40 7.39 7.39 7.39 7.38 7.38 7.37 7.37 7.36 7.26 CDCl3 7.19 7.17 7.16 7.15 7.14 7.12
1.60
CO_2tBu
Ph
F
F
9s
1.00
2.03
3.03
1.04
9.03
f1 (ppm)

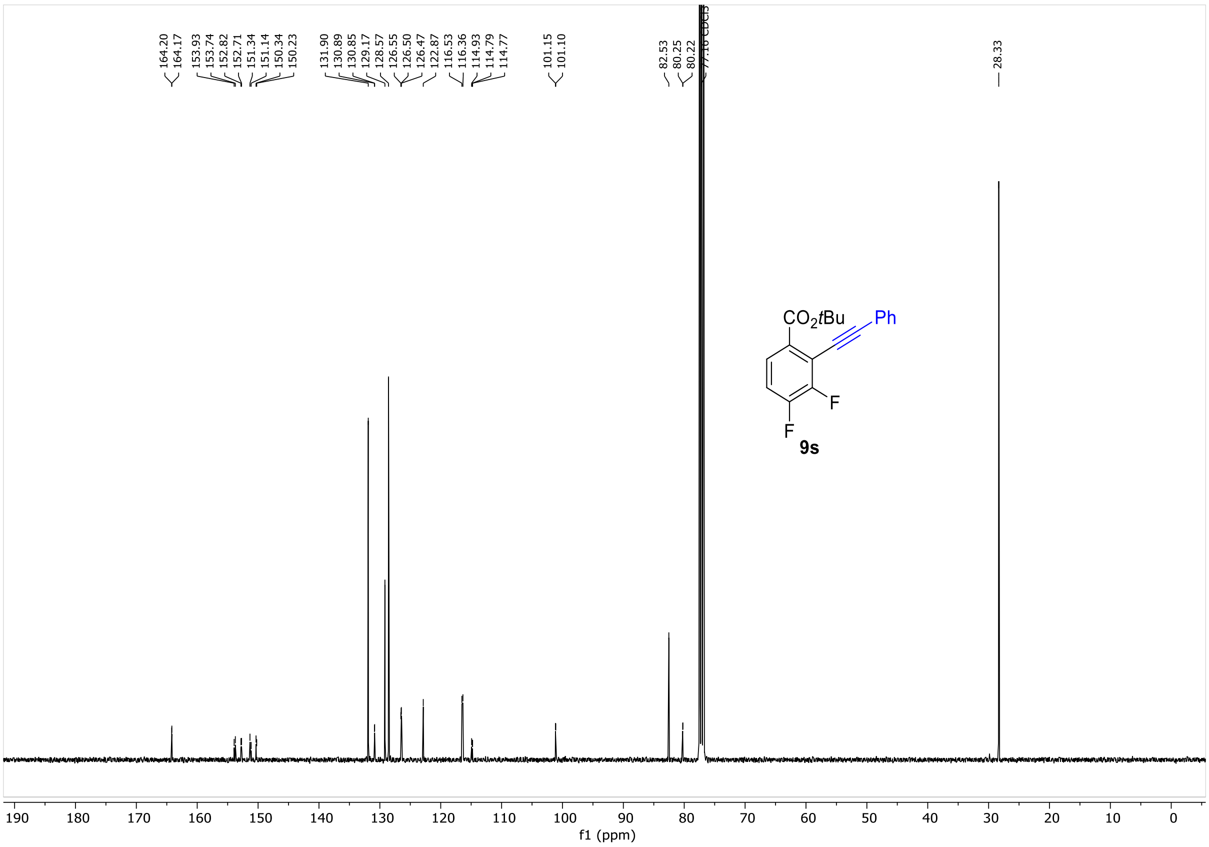
164.20
164.17
153.93
153.74
152.82
152.71
151.34
151.14
150.34
150.23
131.90
130.89
130.85
129.17
128.57
126.55
126.50
126.47
122.87
116.53
116.36
114.93
114.79
114.77
101.15
101.10
82.53
80.25
80.22
77.16 CDCl3
28.33
CO2tBu
Ph
F
F
9s
f1 (ppm)

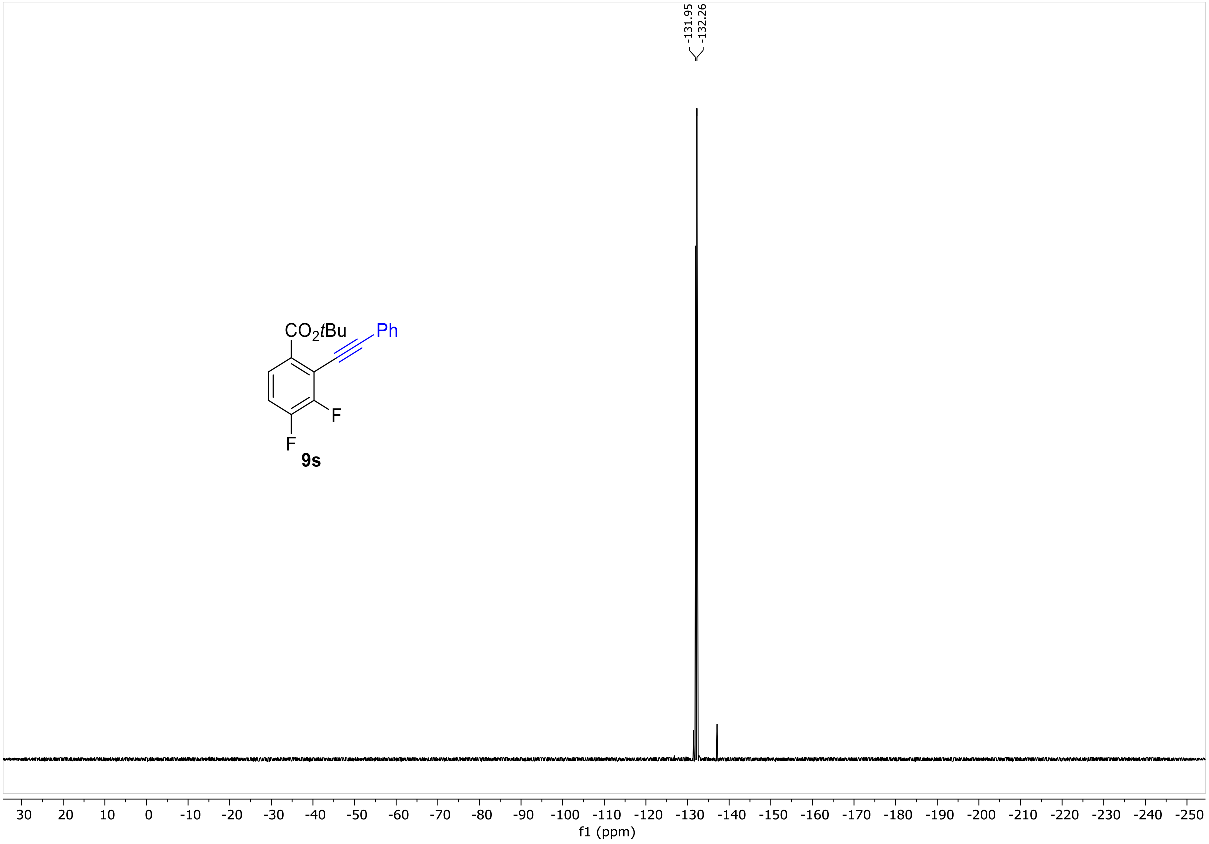
-131.95
-132.26
CO2tBu
Ph
F
F
9s
f1 (ppm)

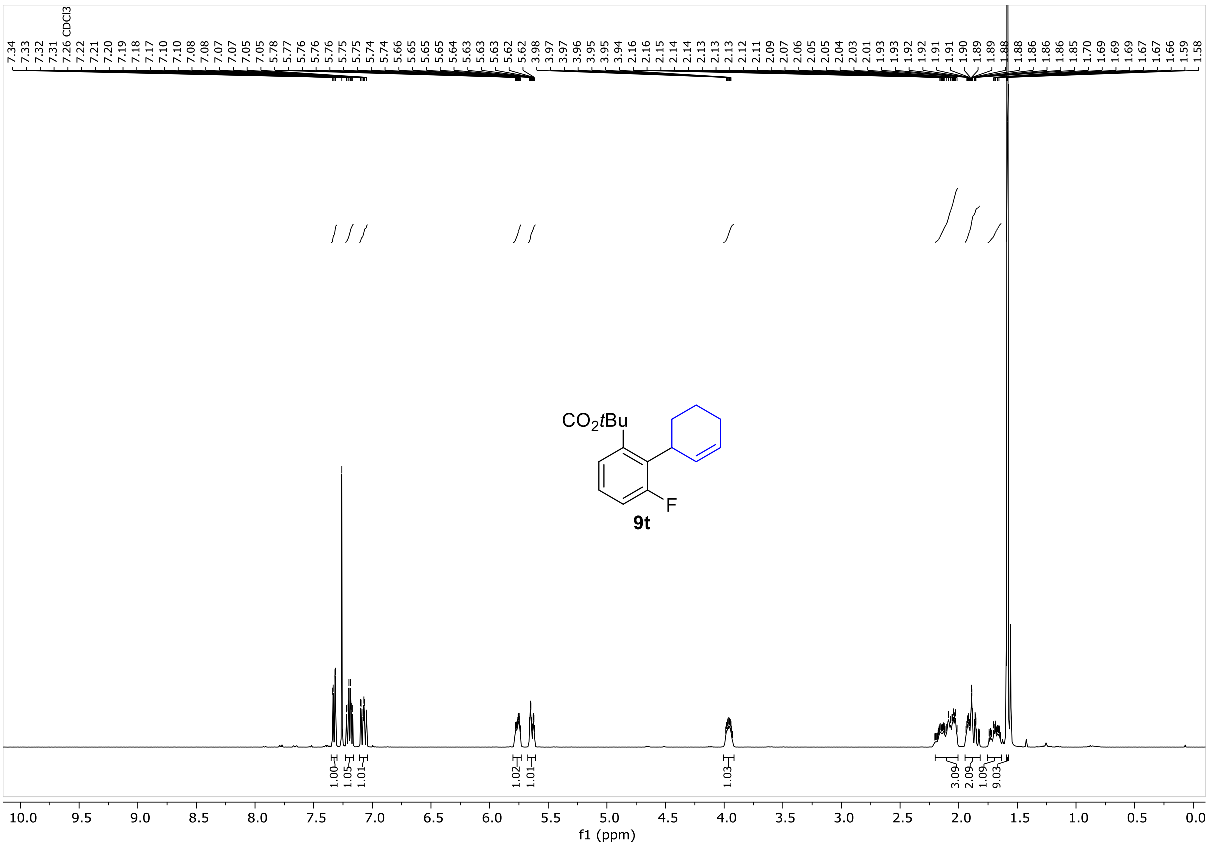
CO2tBu
F
9t
f1 (ppm)

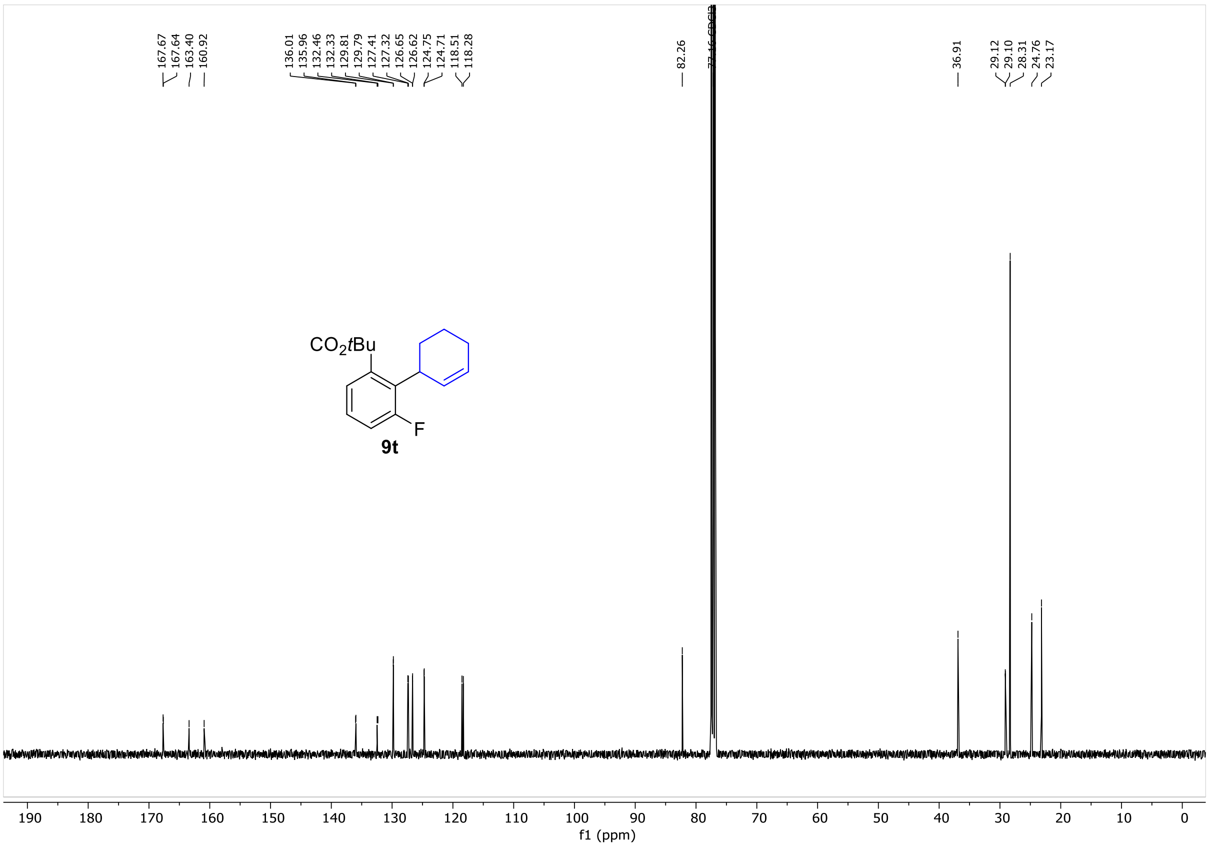
CO2tBu
F
9t
f1 (ppm)

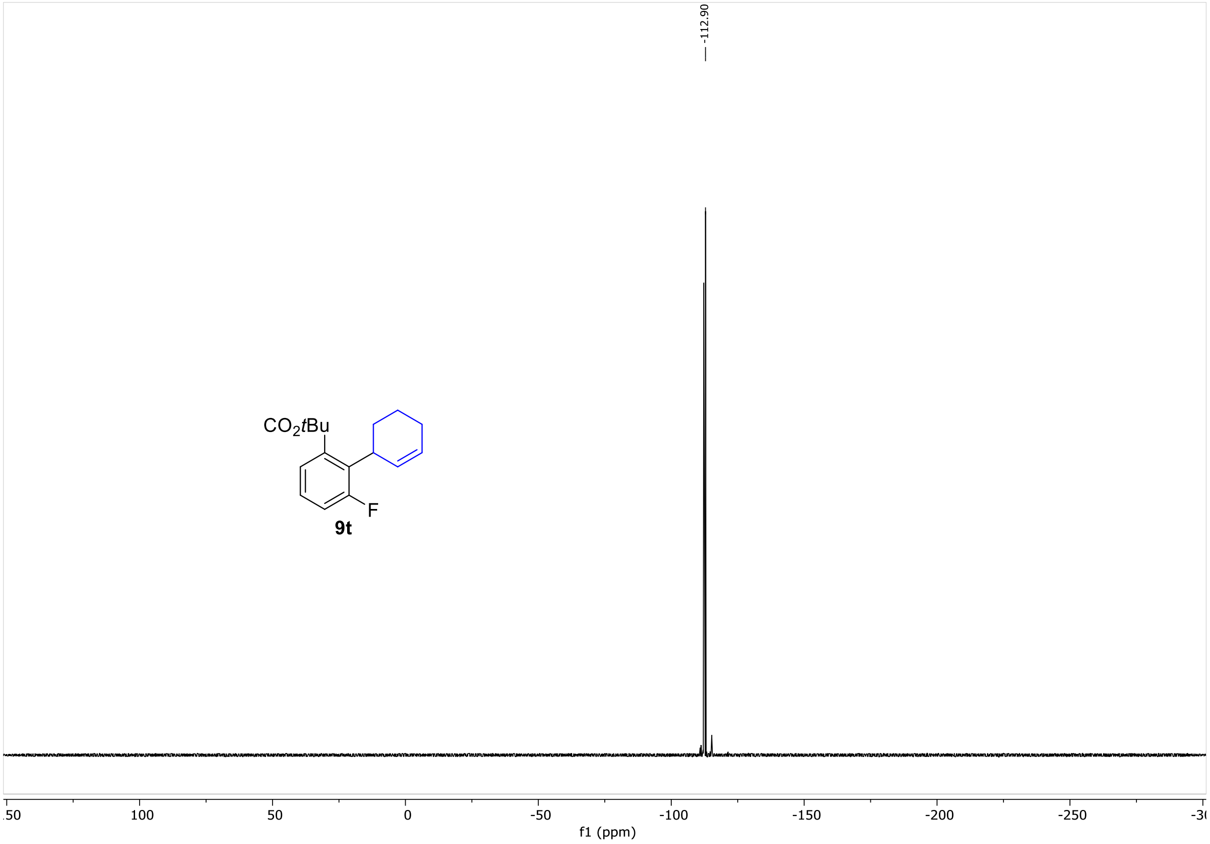
-112.90
CO2tBu
F
9t
f1 (ppm)

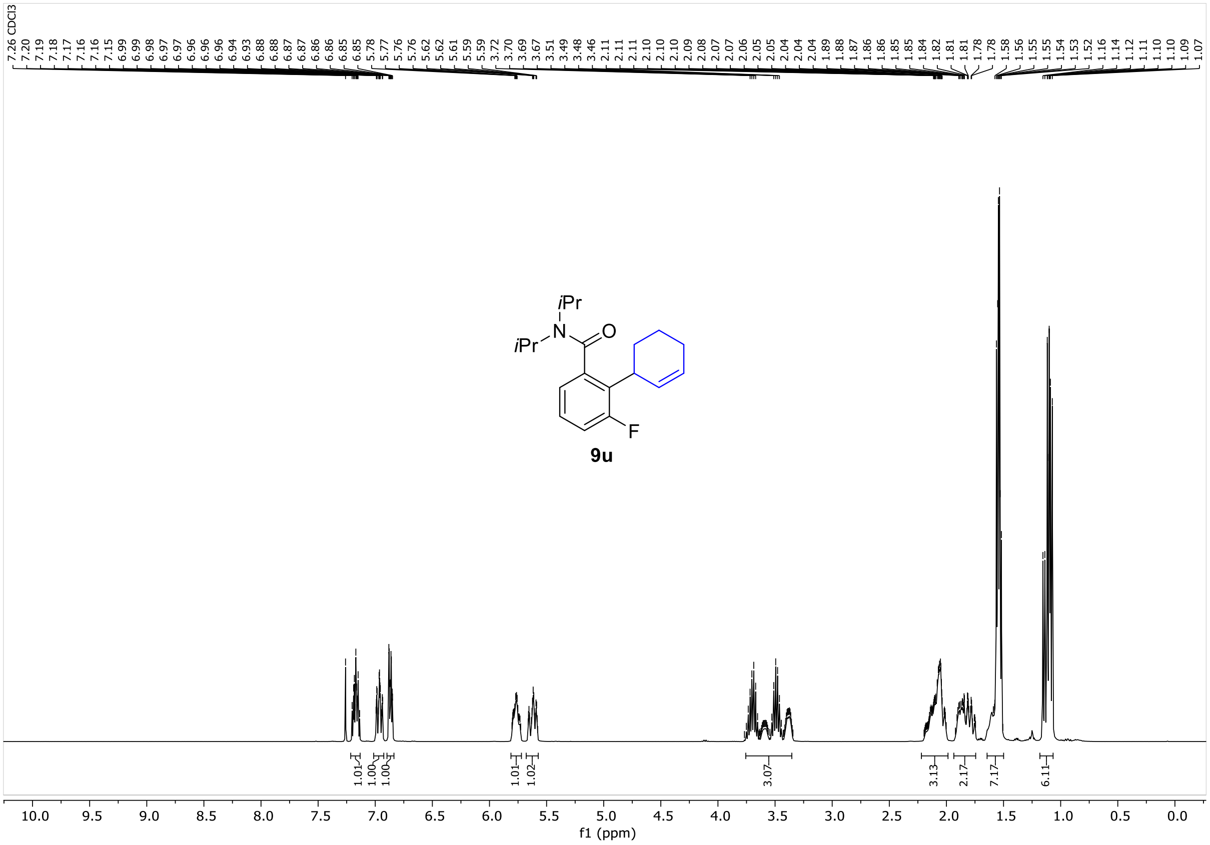
7.26 CDCl3
iPr
N
O
iPr
F
9u
f1 (ppm)

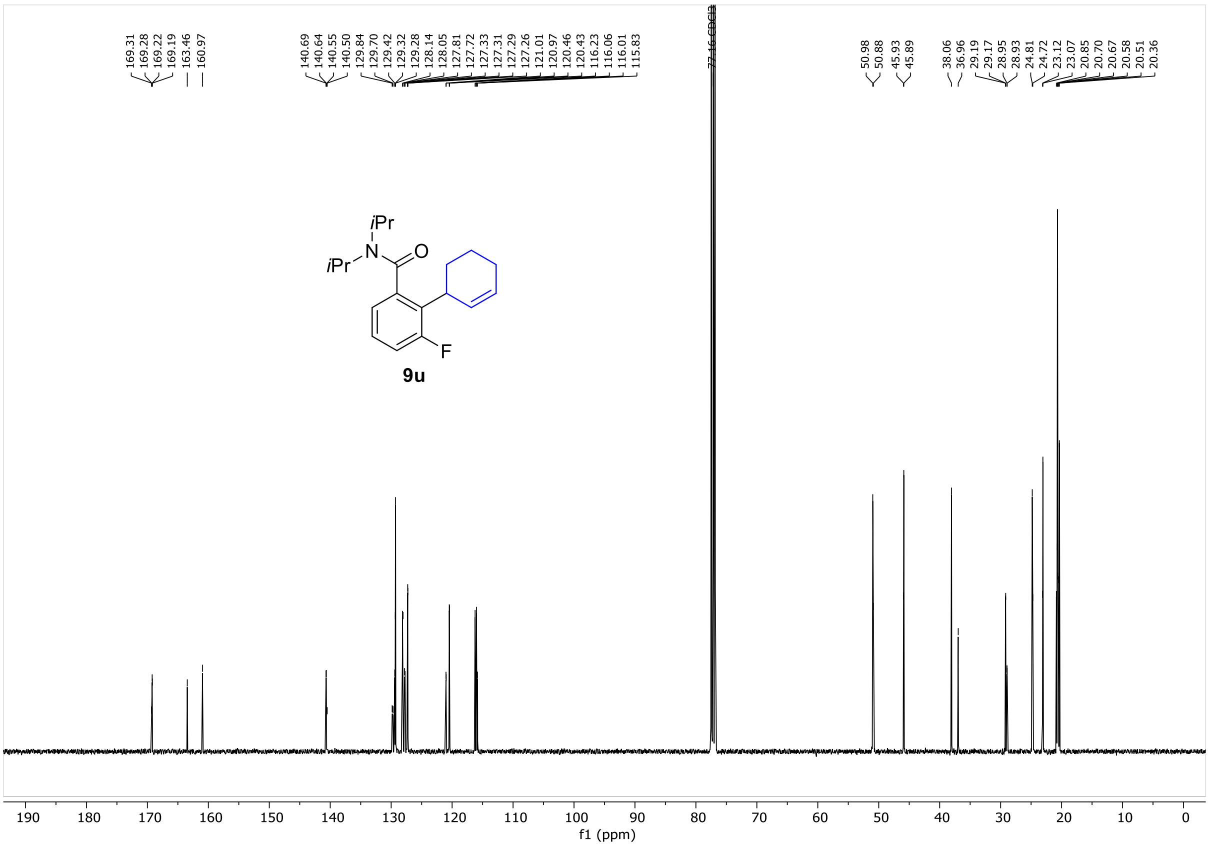
169.31
169.28
169.22
169.19
163.46
160.97
140.69
140.64
140.55
140.50
129.84
129.70
129.42
129.32
129.28
128.14
128.05
127.81
127.72
127.33
127.31
127.29
127.26
121.01
120.97
120.46
120.43
116.23
116.06
116.01
115.83
77.16 CDCl3
50.98
50.88
45.93
45.89
38.06
36.96
29.19
29.17
28.95
28.93
24.81
24.72
23.12
23.07
20.85
20.70
20.67
20.58
20.51
20.36
iPr
N
O
iPr
F
9u
f1 (ppm)

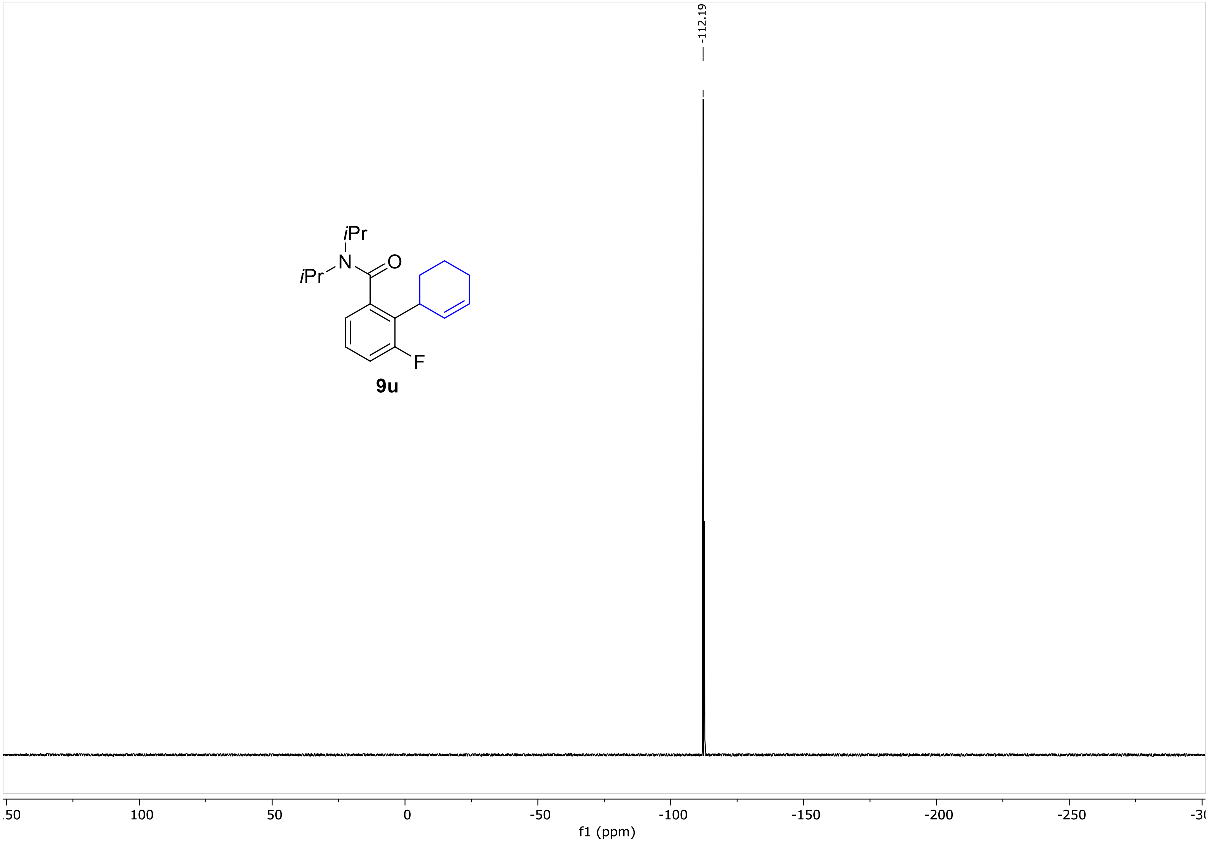
-112.19
iPr
N
O
iPr
F
9u
f1 (ppm)

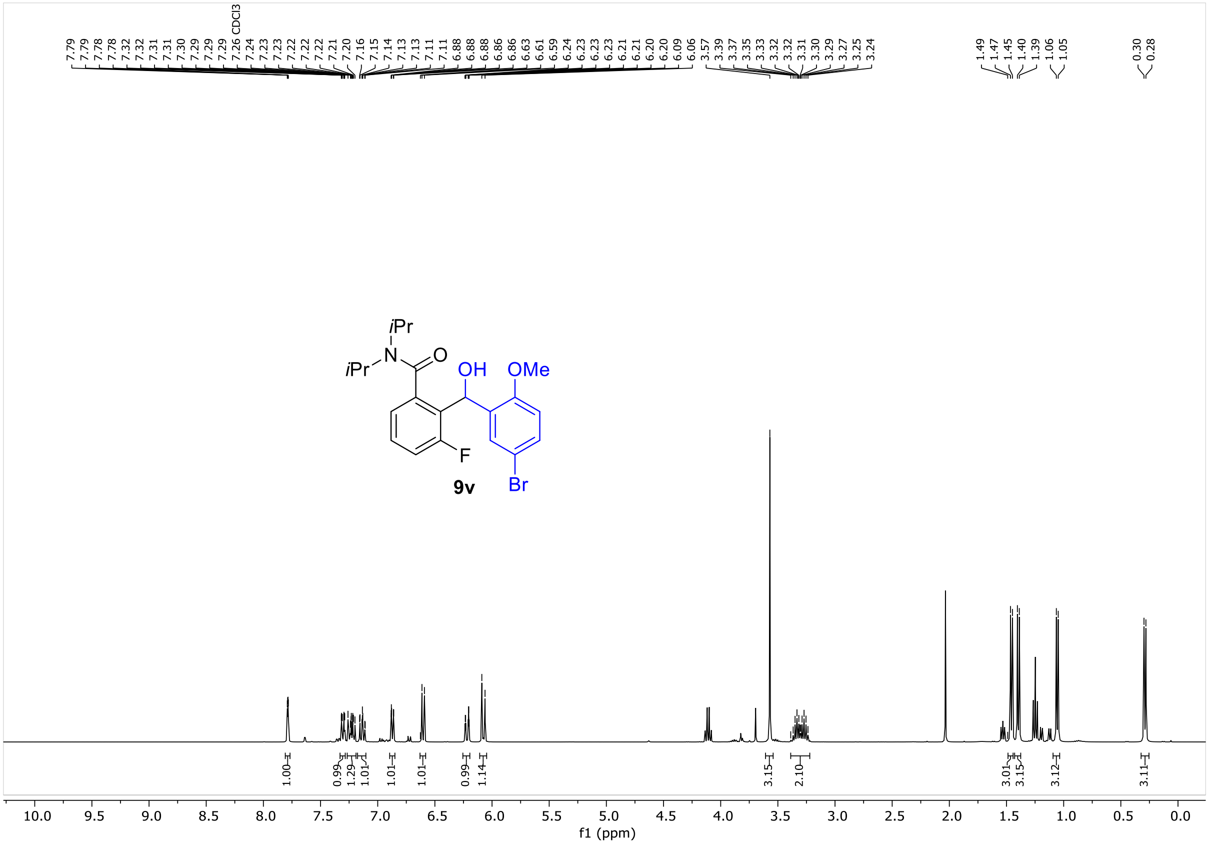
iPr
N
O
iPr
OH
OMe
F
9v
Br
f1 (ppm)

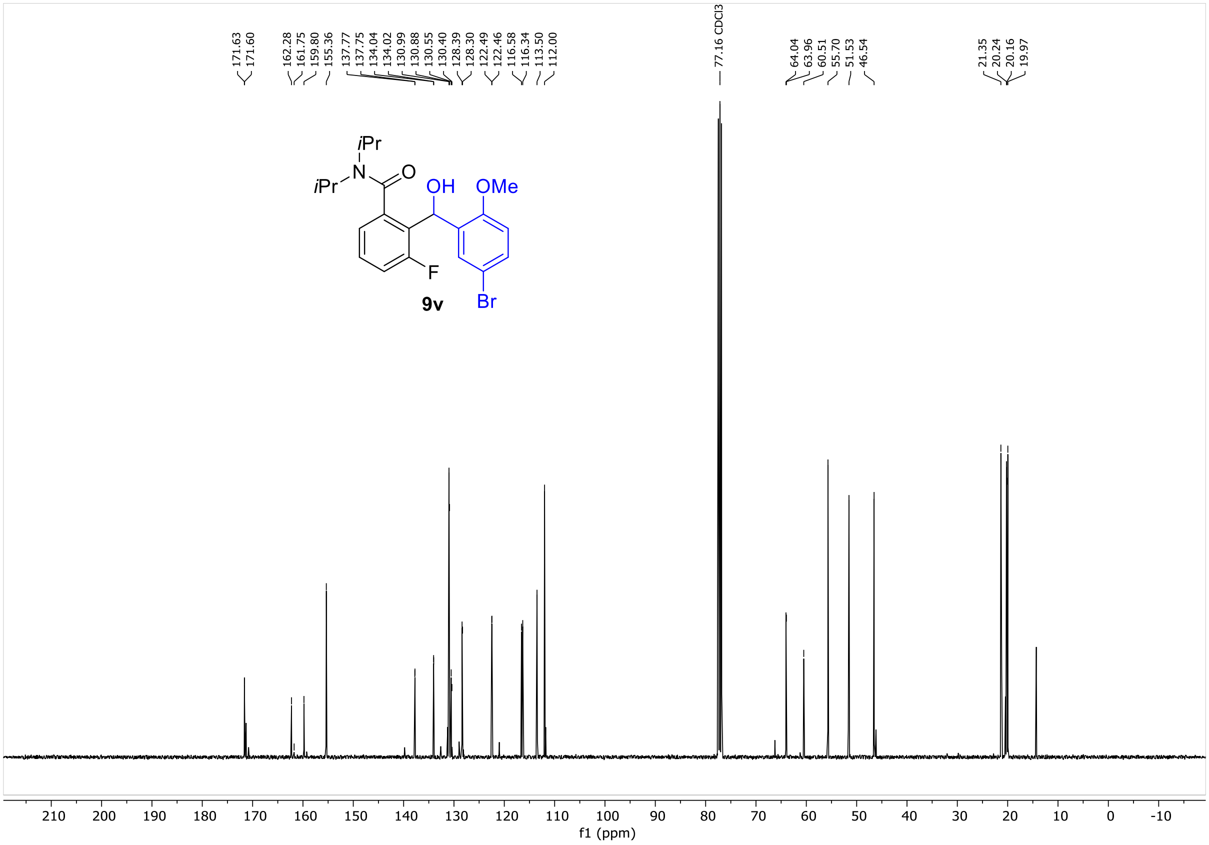
iPr
N
O
iPr
OH
OMe
F
9v
Br
77.16 CDCl3
f1 (ppm)

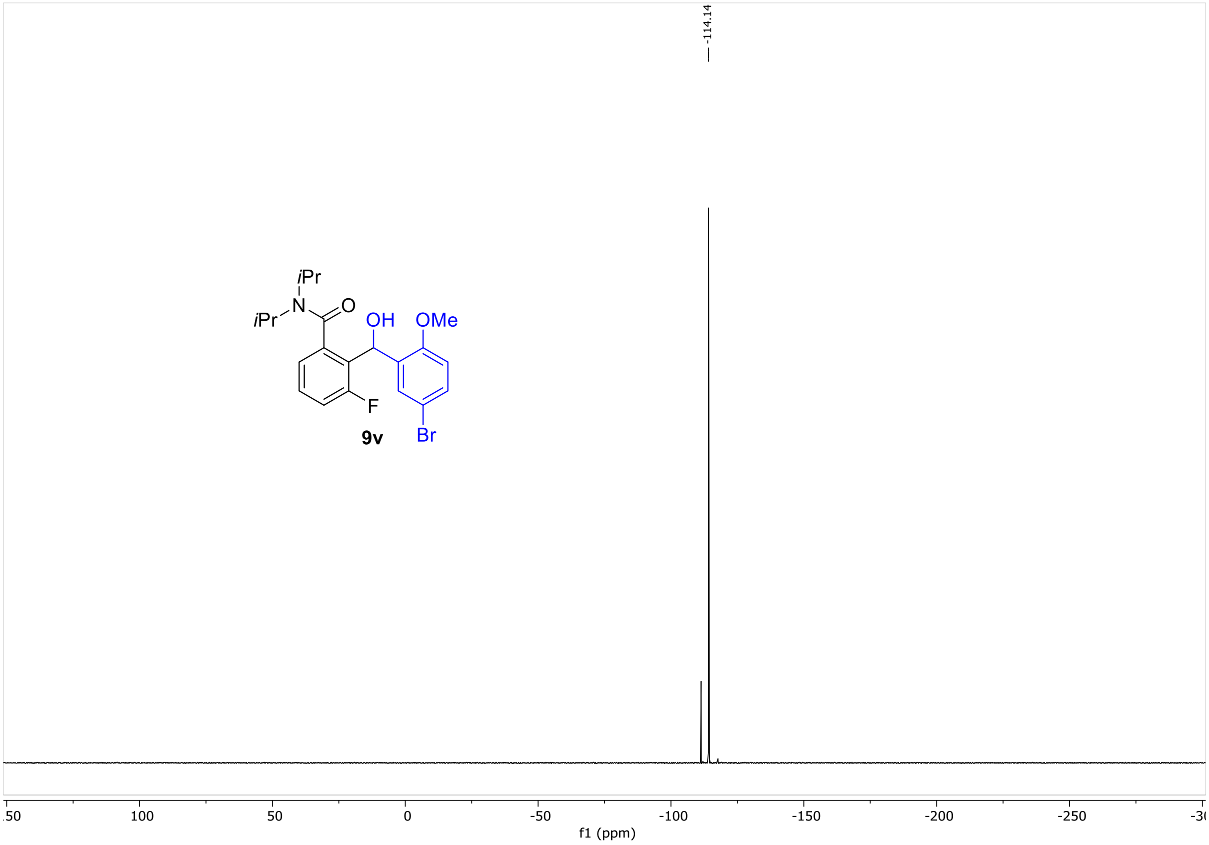
-114.14
iPr
N
O
iPr
OH
OMe
F
Br
9v
f1 (ppm)

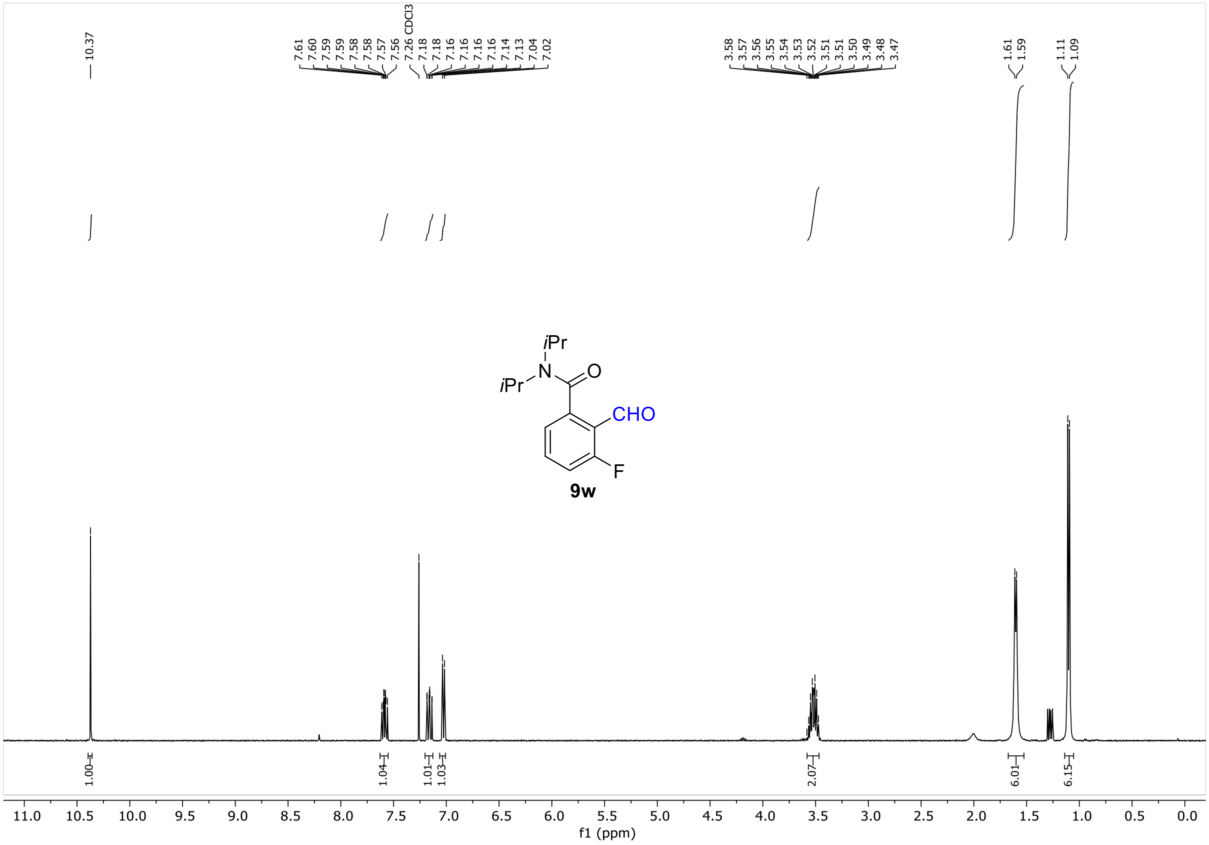
10.37
7.61 7.60 7.59 7.59 7.58 7.58 7.57 7.56 7.26 CDCl3 7.18 7.18 7.16 7.16 7.16 7.16 7.14 7.13 7.04 7.02
3.58 3.57 3.56 3.55 3.54 3.53 3.52 3.51 3.51 3.50 3.49 3.48 3.47
1.61 1.59
1.11 1.09
iPr
N
O
iPr
CHO
F
9w
1.00
1.04
1.01
1.03
2.07
6.01
6.15
f1 (ppm)

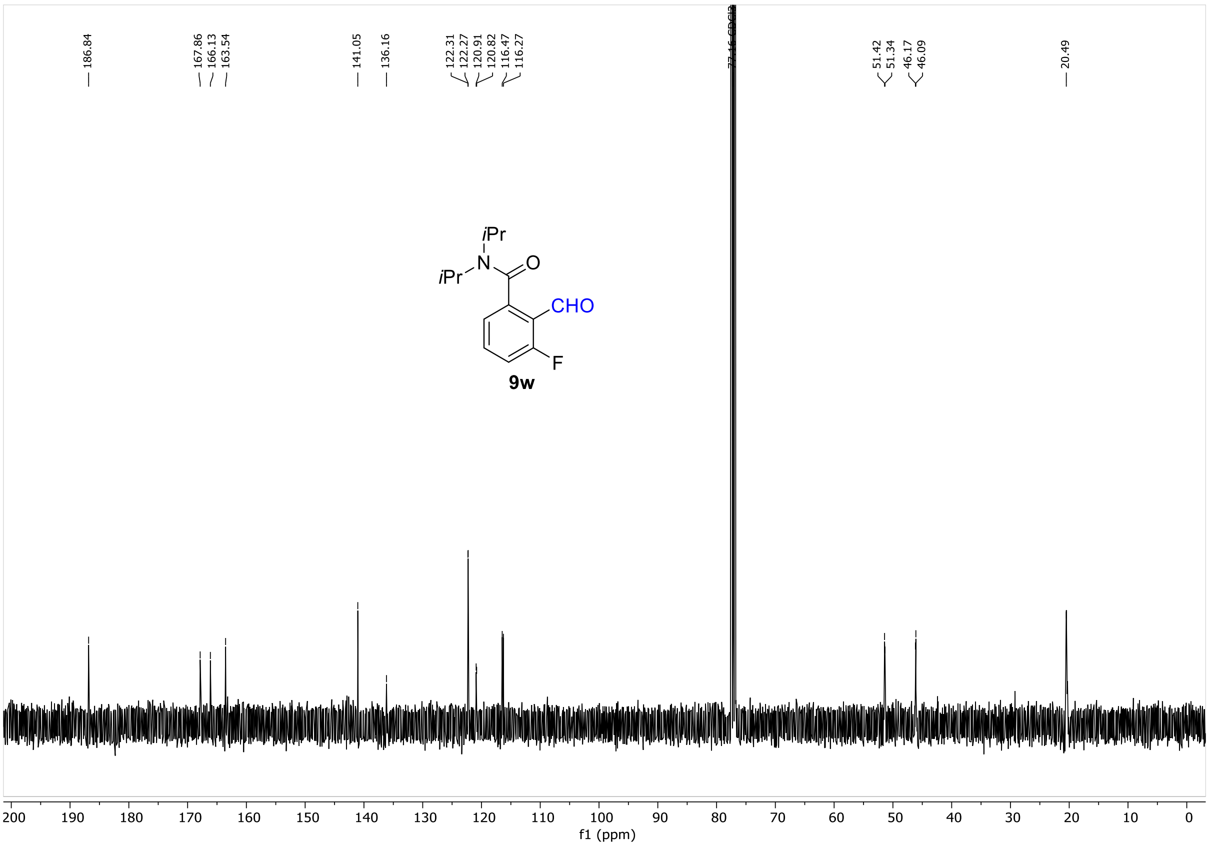
186.84
167.86
166.13
163.54
141.05
136.16
122.31
122.27
120.91
120.82
116.47
116.27
51.42
51.34
46.17
46.09
20.49
iPr
N
O
iPr
CHO
F
9w
200 190 180 170 160 150 140 130 120 110 100 90 80 70 60 50 40 30 20 10 0
f1 (ppm)

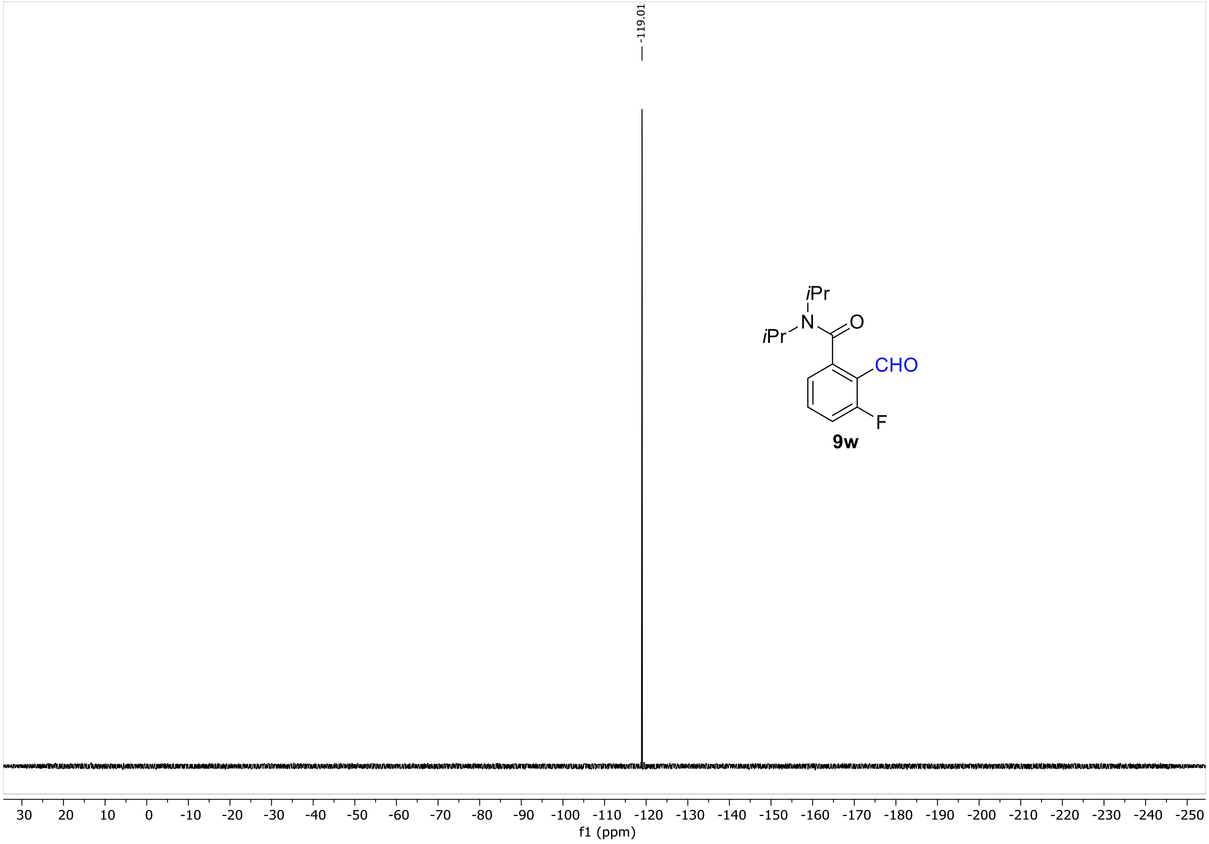
-119.01
iPr
N
O
iPr
CHO
F
9w
30 20 10 0 -10 -20 -30 -40 -50 -60 -70 -80 -90 -100 -110 -120 -130 -140 -150 -160 -170 -180 -190 -200 -210 -220 -230 -240 -250
f1 (ppm)

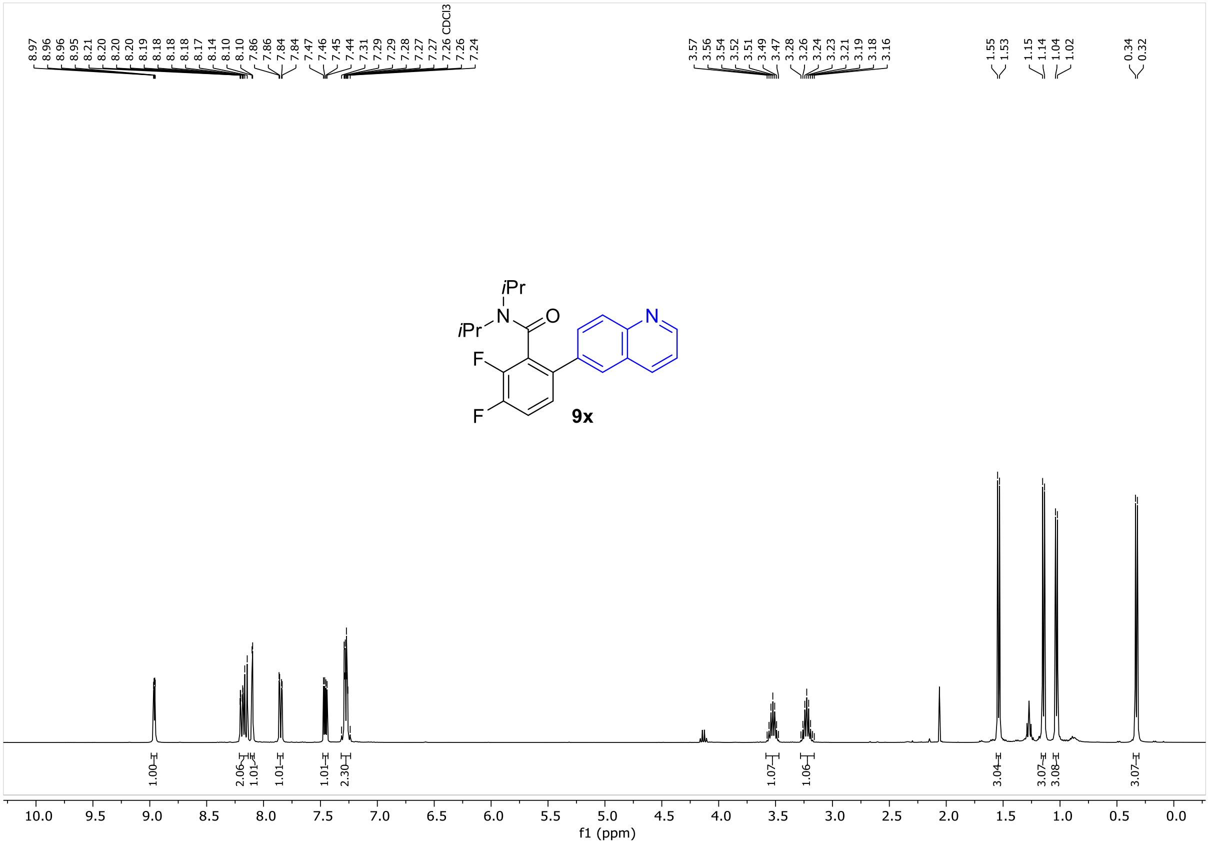
8.97 8.96 8.96 8.95 8.21 8.20 8.20 8.20 8.19 8.18 8.18 8.18 8.17 8.14 8.10 8.10 7.86 7.86 7.84 7.84 7.47 7.46 7.45 7.44 7.31 7.29 7.29 7.28 7.27 7.27 7.26 CDCl3 7.26 7.24
3.57 3.56 3.54 3.52 3.51 3.49 3.47 3.28 3.26 3.24 3.23 3.21 3.19 3.18 3.16
1.55 1.53 1.15 1.14 1.04 1.02 0.34 0.32
iPr
N
O
iPr
F
F
N
9x
1.00 2.06 1.01 1.01 1.01 2.30 1.07 1.06 3.04 3.07 3.08 3.07
10.0 9.5 9.0 8.5 8.0 7.5 7.0 6.5 6.0 5.5 5.0 4.5 4.0 3.5 3.0 2.5 2.0 1.5 1.0 0.5 0.0
f1 (ppm)

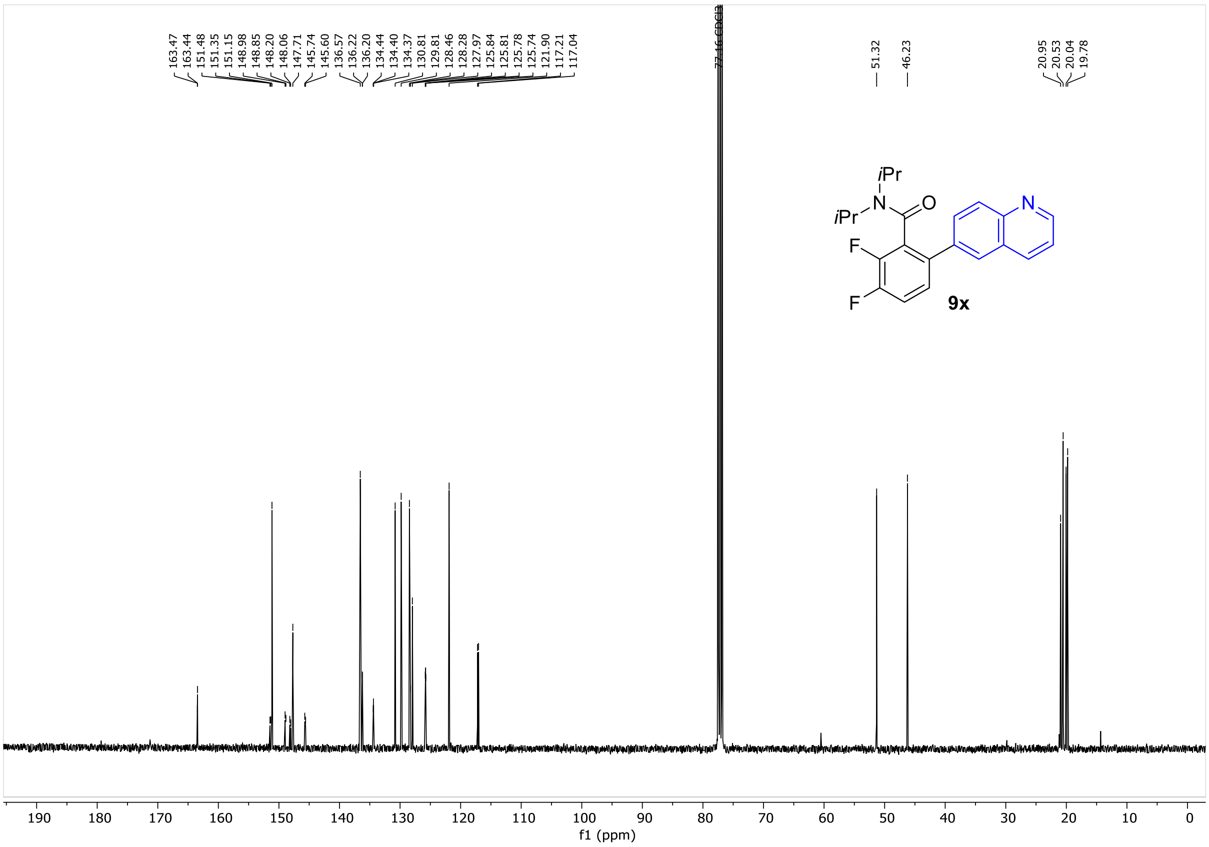
163.47 163.44 151.48 151.35 151.15 148.98 148.85 148.20 148.06 147.71 145.74 145.60 136.57 136.22 136.20 134.44 134.40 134.37 130.81 129.81 128.46 128.28 127.97 125.84 125.81 125.78 125.74 121.90 117.21 117.04
51.32
46.23
20.95 20.53 20.04 19.78
iPr
N
O
iPr
F
F
N
9x
190 180 170 160 150 140 130 120 110 100 90 80 70 60 50 40 30 20 10 0
f1 (ppm)

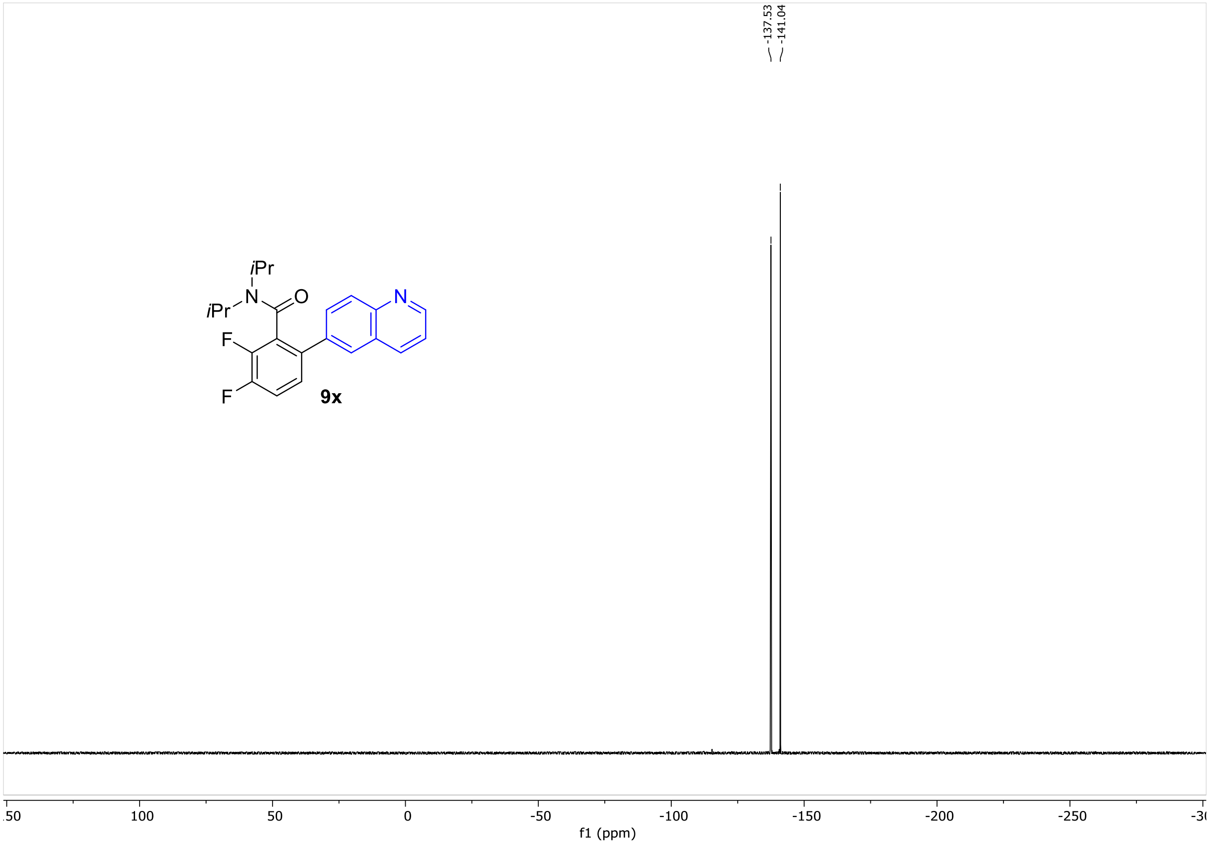
-137.53
-141.04
iPr
N
O
iPr
F
F
N
9x
f1 (ppm)

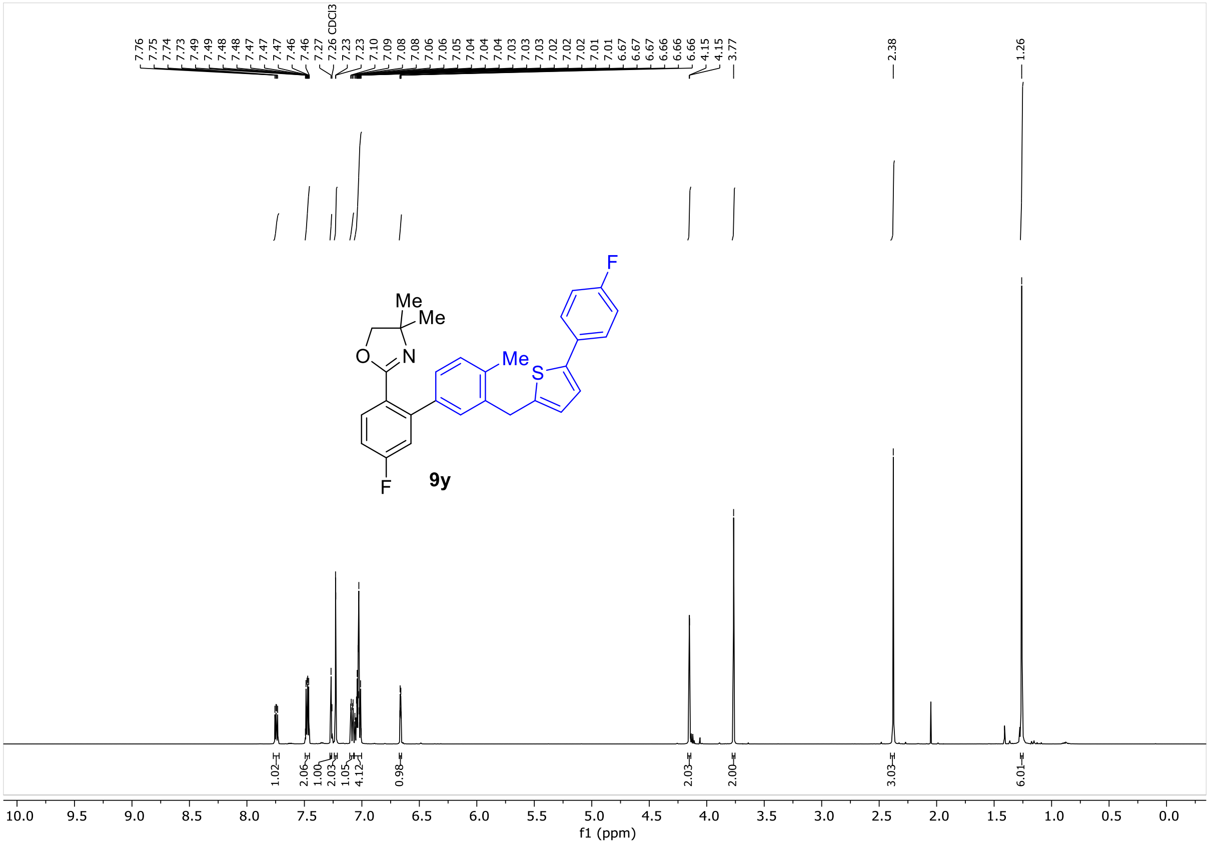
CDCl3
2.38
1.26
Me
Me
O
N
Me
S
F
F
9y
1.02
2.06
1.00
2.03
1.05
4.12
0.98
2.03
2.00
3.03
6.01
f1 (ppm)

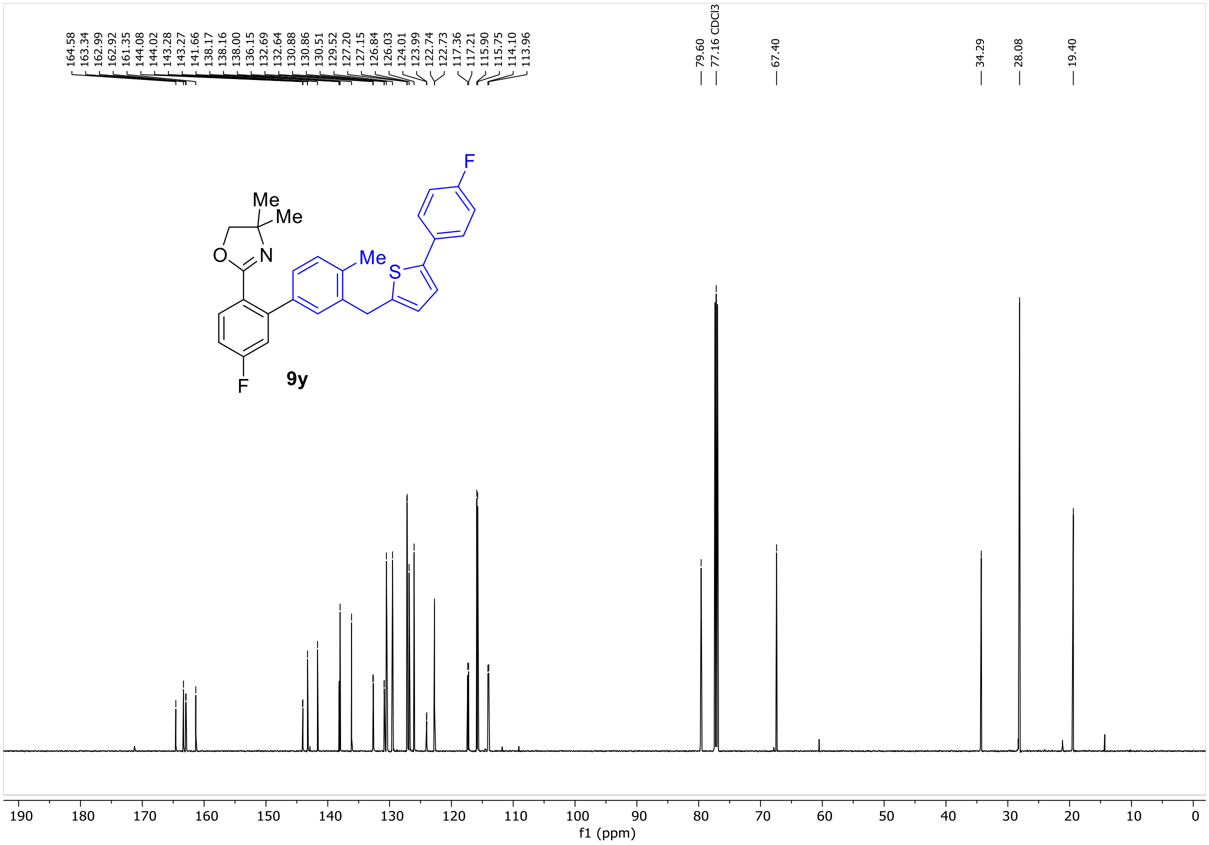
164.58
163.34
162.99
162.92
161.35
144.08
144.02
143.28
143.27
141.66
138.17
138.16
138.00
136.15
132.69
132.64
130.88
130.86
130.51
129.52
127.20
127.15
126.84
126.03
124.01
123.99
122.74
122.73
117.36
117.21
115.90
115.75
114.10
113.96
79.60
77.16 CDCl3
67.40
34.29
28.08
19.40
Me
Me
O
N
Me
S
F
F
9y
190 180 170 160 150 140 130 120 110 100 90 80 70 60 50 40 30 20 10 0
f1 (ppm)

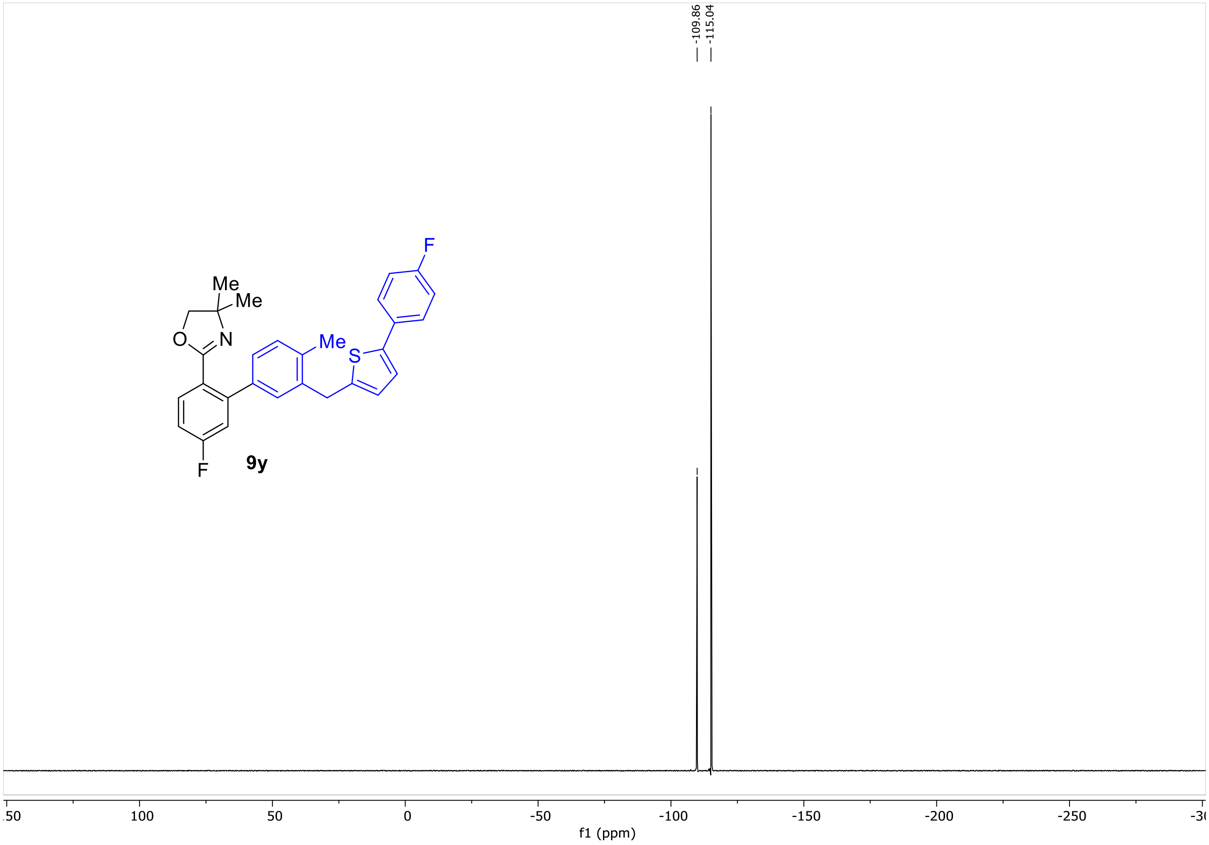
-109.86
-115.04
Me
Me
O
N
Me
S
F
F
9y
150 100 50 0 -50 -100 -150 -200 -250 -300
f1 (ppm)

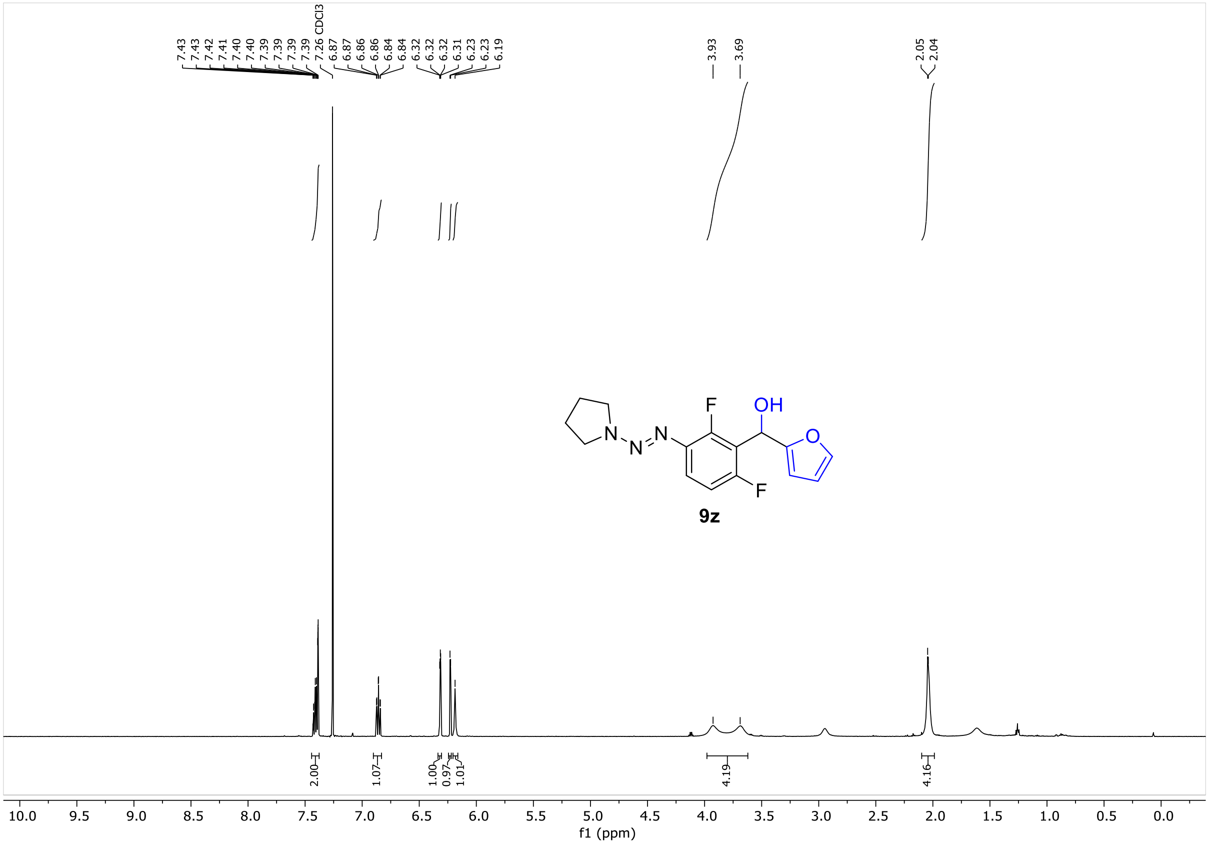
7.43
7.43
7.42
7.41
7.40
7.40
7.39
7.39
7.39
7.39
7.26 CDCl3
6.87
6.87
6.86
6.86
6.84
6.84
6.32
6.32
6.32
6.31
6.23
6.23
6.19
3.93
3.69
2.05
2.04
F
OH
N
N
N
O
F
9z
2.00
1.07
1.00
0.97
1.01
4.19
4.16
10.0 9.5 9.0 8.5 8.0 7.5 7.0 6.5 6.0 5.5 5.0 4.5 4.0 3.5 3.0 2.5 2.0 1.5 1.0 0.5 0.0
f1 (ppm)

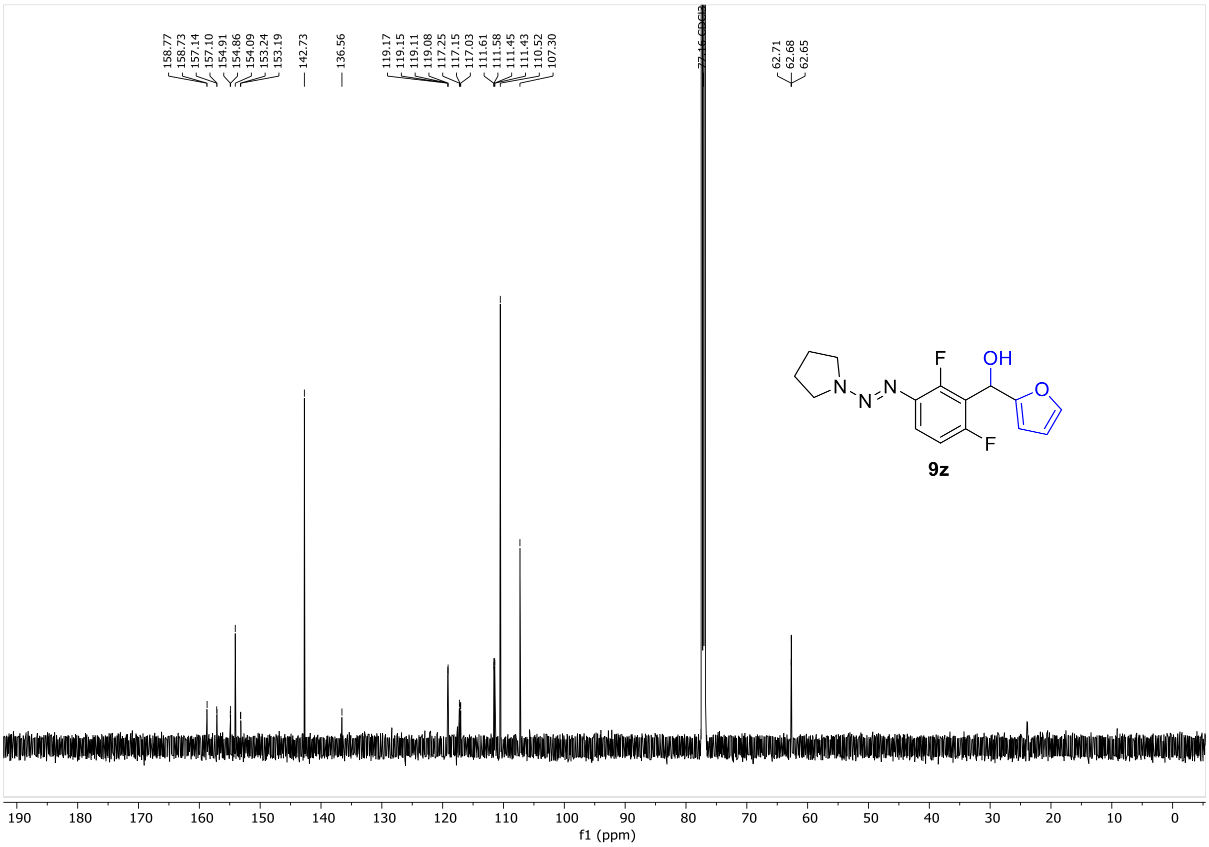
158.77
158.73
157.14
157.10
154.91
154.86
154.09
153.24
153.19
142.73
136.56
119.17
119.15
119.11
119.08
117.25
117.15
117.03
111.61
111.58
111.45
111.43
110.52
107.30
77.16 CDCl3
62.71
62.68
62.65
F
OH
N
N
N
O
F
9z
190 180 170 160 150 140 130 120 110 100 90 80 70 60 50 40 30 20 10 0
f1 (ppm)

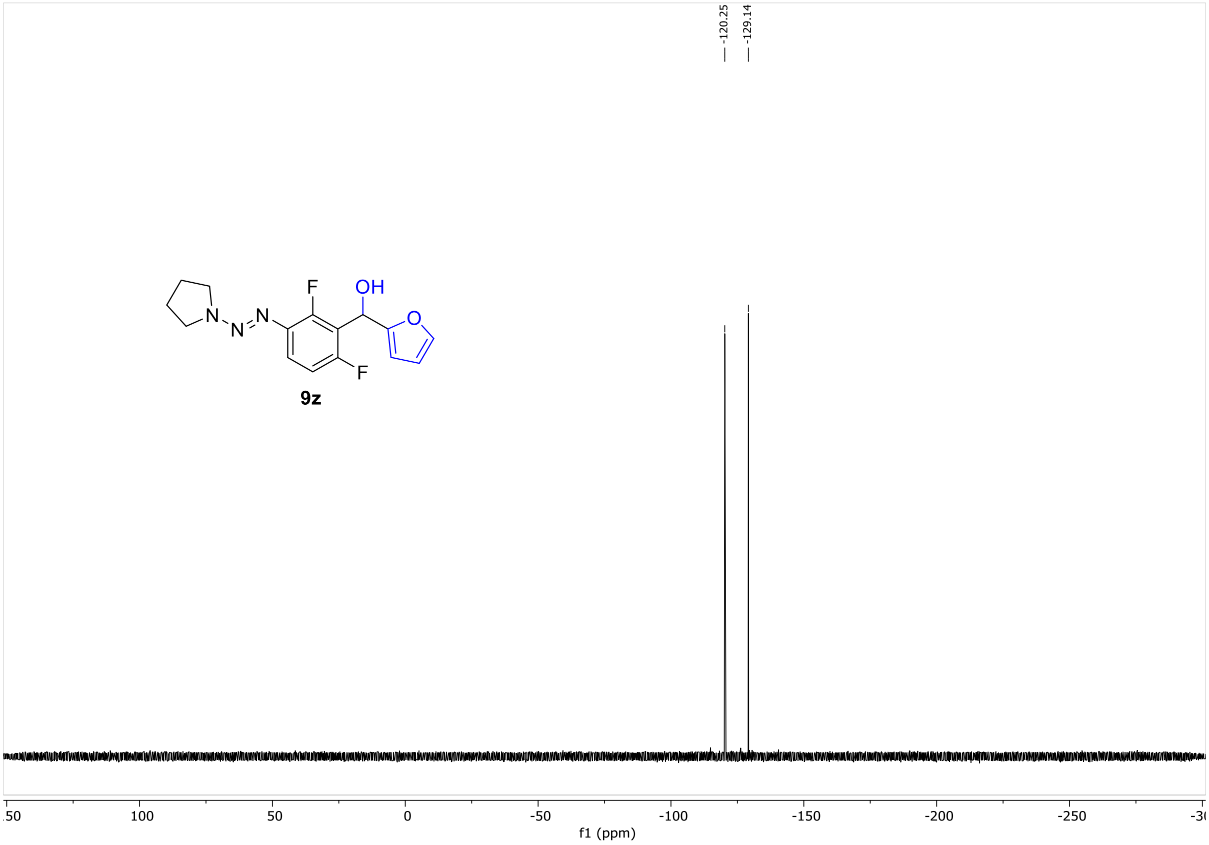

-120.25
-129.14
OH
N
N
N
F
O
F
9z
f1 (ppm)

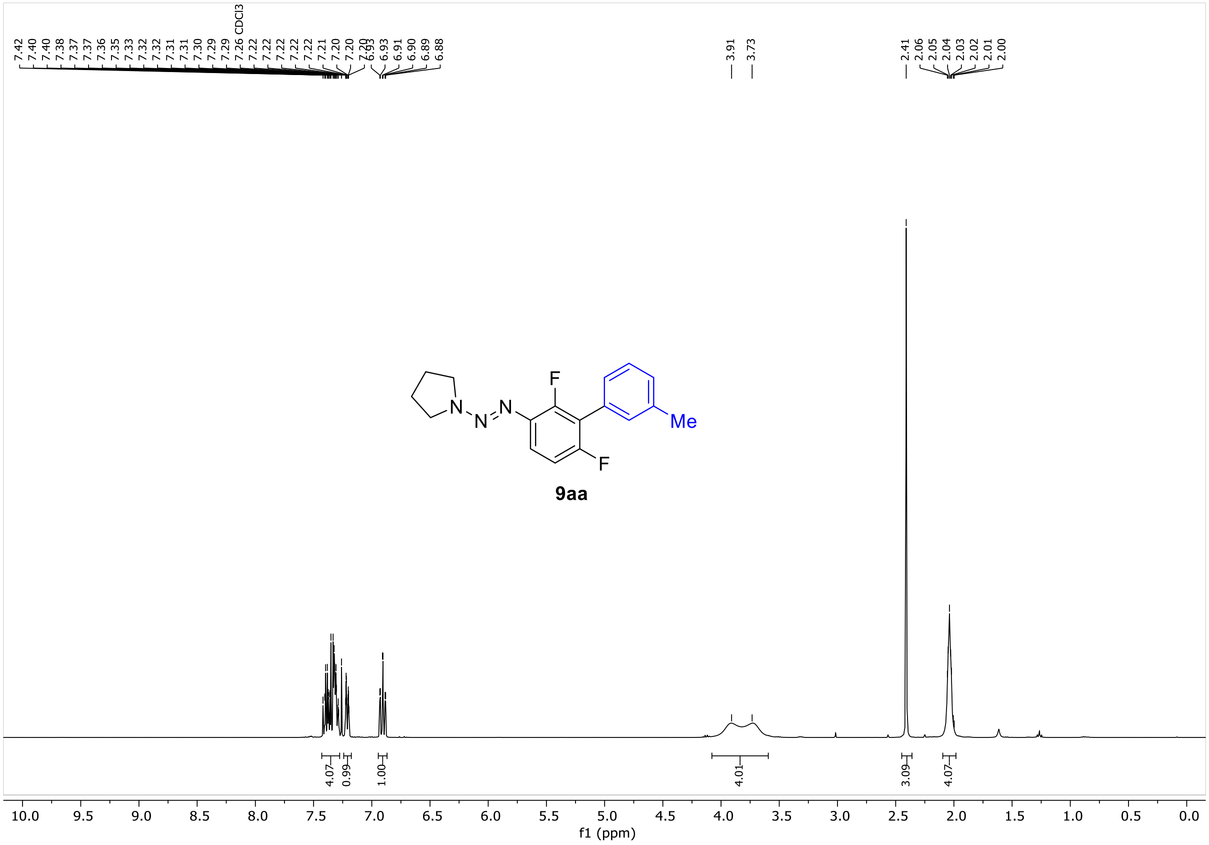

7.42
7.40
7.40
7.38
7.37
7.37
7.36
7.35
7.33
7.32
7.32
7.31
7.31
7.30
7.29
7.29
7.26 CDCl3
7.22
7.22
7.22
7.22
7.22
7.21
7.20
7.20
6.93
6.91
6.90
6.89
6.88
3.91
3.73
2.41
2.06
2.05
2.04
2.03
2.02
2.01
2.00
F
Me
F
9aa
4.07
0.99
1.00
4.01
3.09
4.07
f1 (ppm)

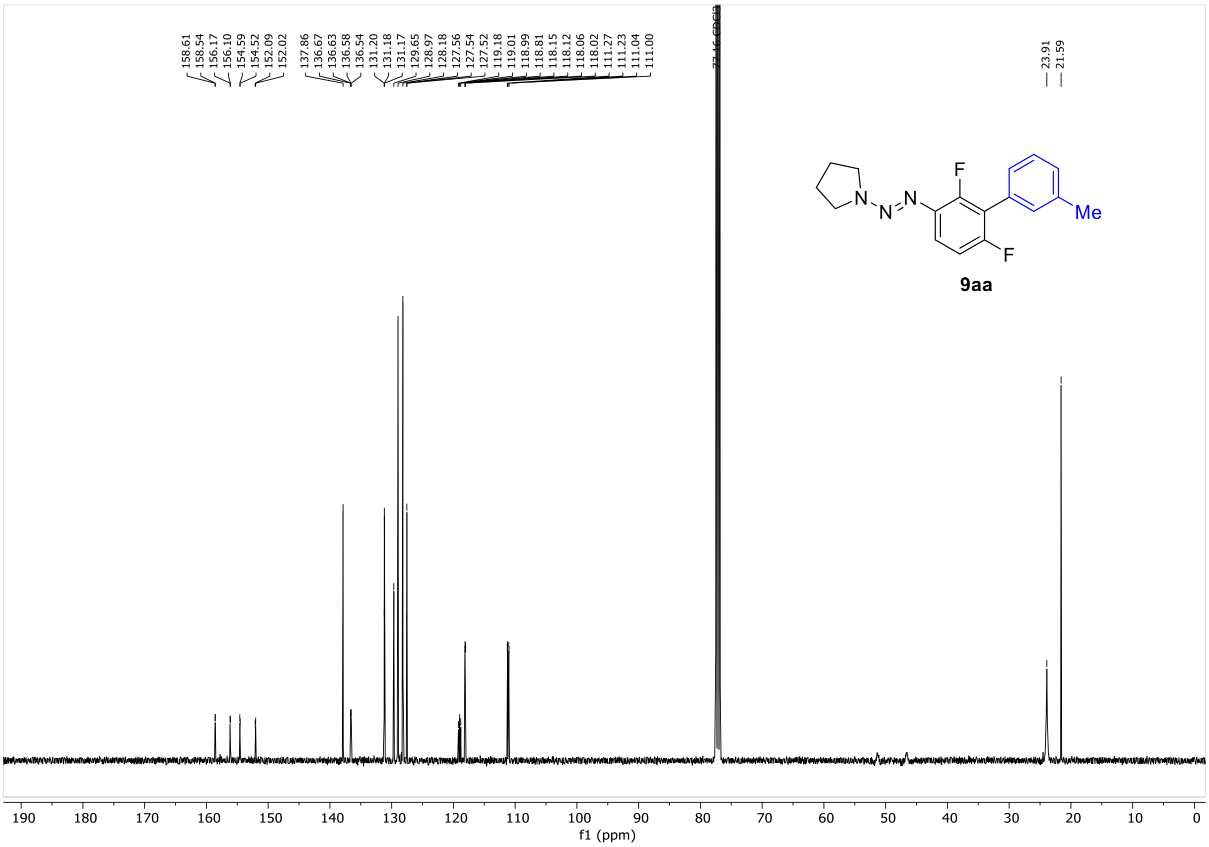
158.61 158.54 156.17 156.10 154.59 154.52 152.09 152.02
137.86 136.67 136.63 136.58 136.54 131.20 131.18 131.17 129.65 128.97 128.18 127.56 127.54 127.52 119.18 119.01 118.99 118.81 118.15 118.12 118.06 118.02 111.27 111.23 111.04 111.00
77.16 CDCl3
23.91
21.59
F
F
Me
9aa
f1 (ppm)

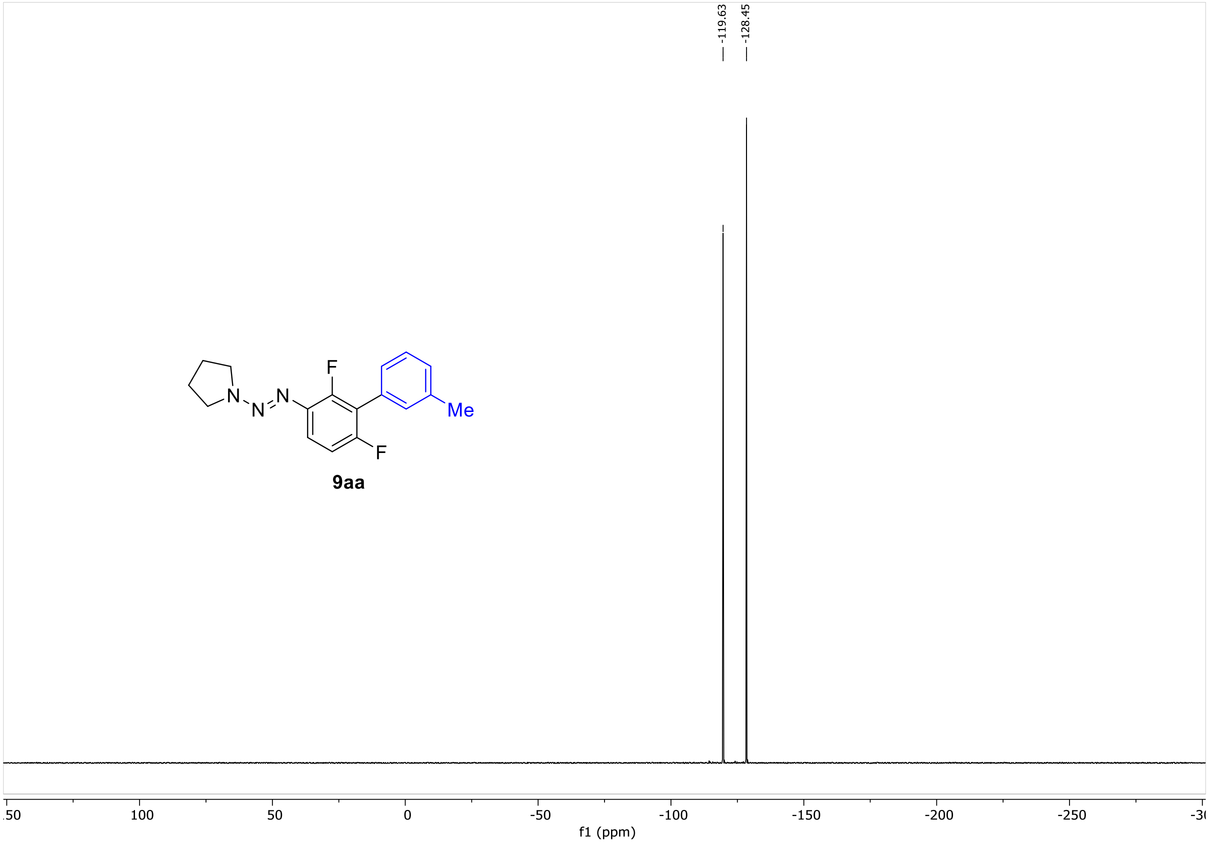
-119.63
-128.45
F
F
Me
9aa
f1 (ppm)

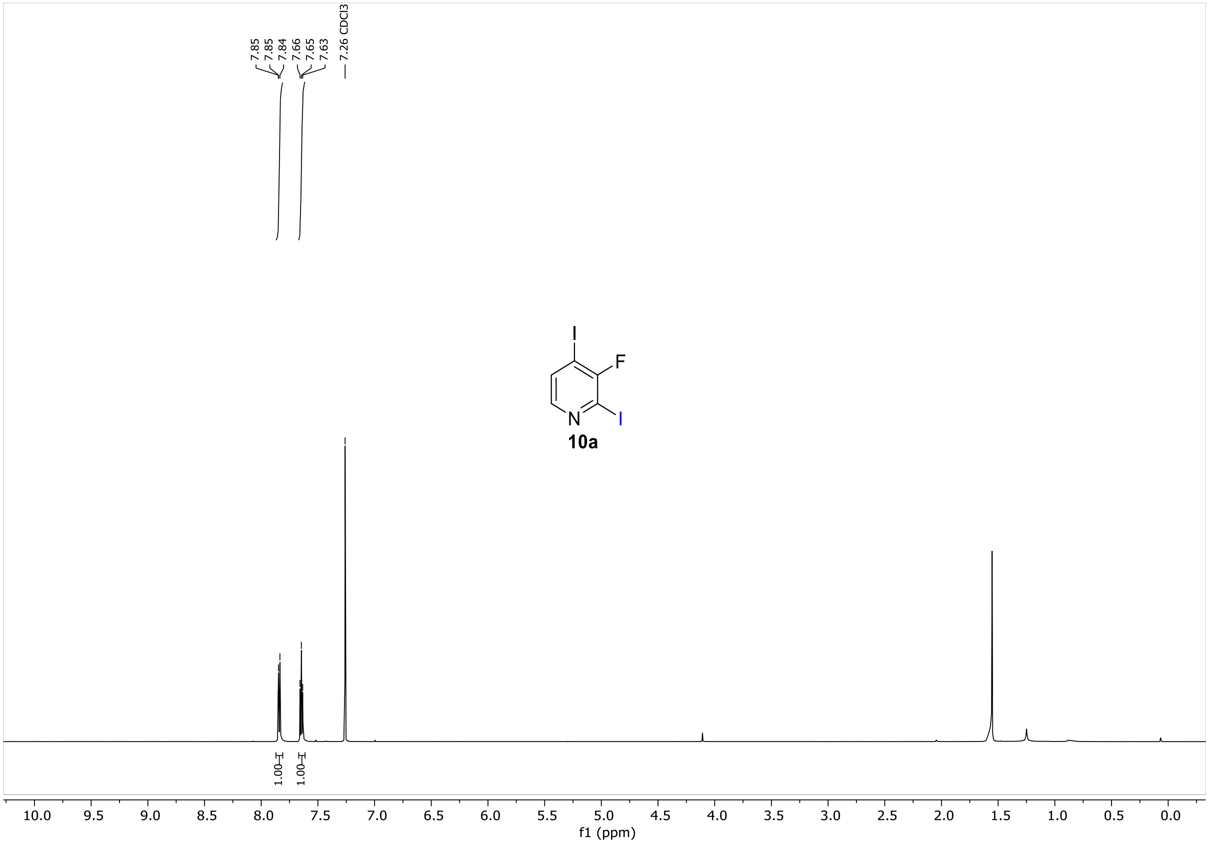
7.85
7.85
7.84
7.66
7.65
7.63
7.26 CDCl3
I
F
N
I
10a
1.00
1.00
10.0 9.5 9.0 8.5 8.0 7.5 7.0 6.5 6.0 5.5 5.0 4.5 4.0 3.5 3.0 2.5 2.0 1.5 1.0 0.5 0.0
f1 (ppm)

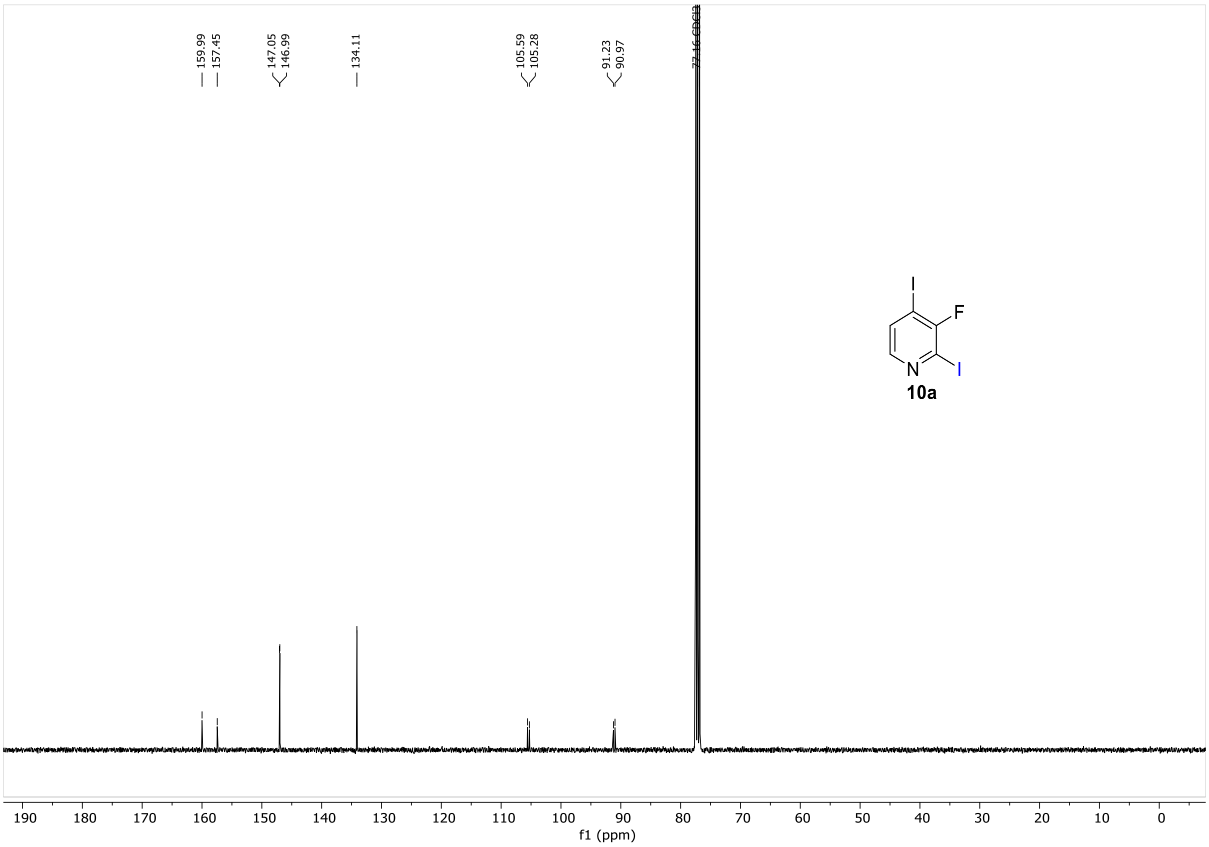
159.99
157.45
147.05
146.99
134.11
105.59
105.28
91.23
90.97
77.16 CDCl3
I
F
N
I
10a
190 180 170 160 150 140 130 120 110 100 90 80 70 60 50 40 30 20 10 0
f1 (ppm)

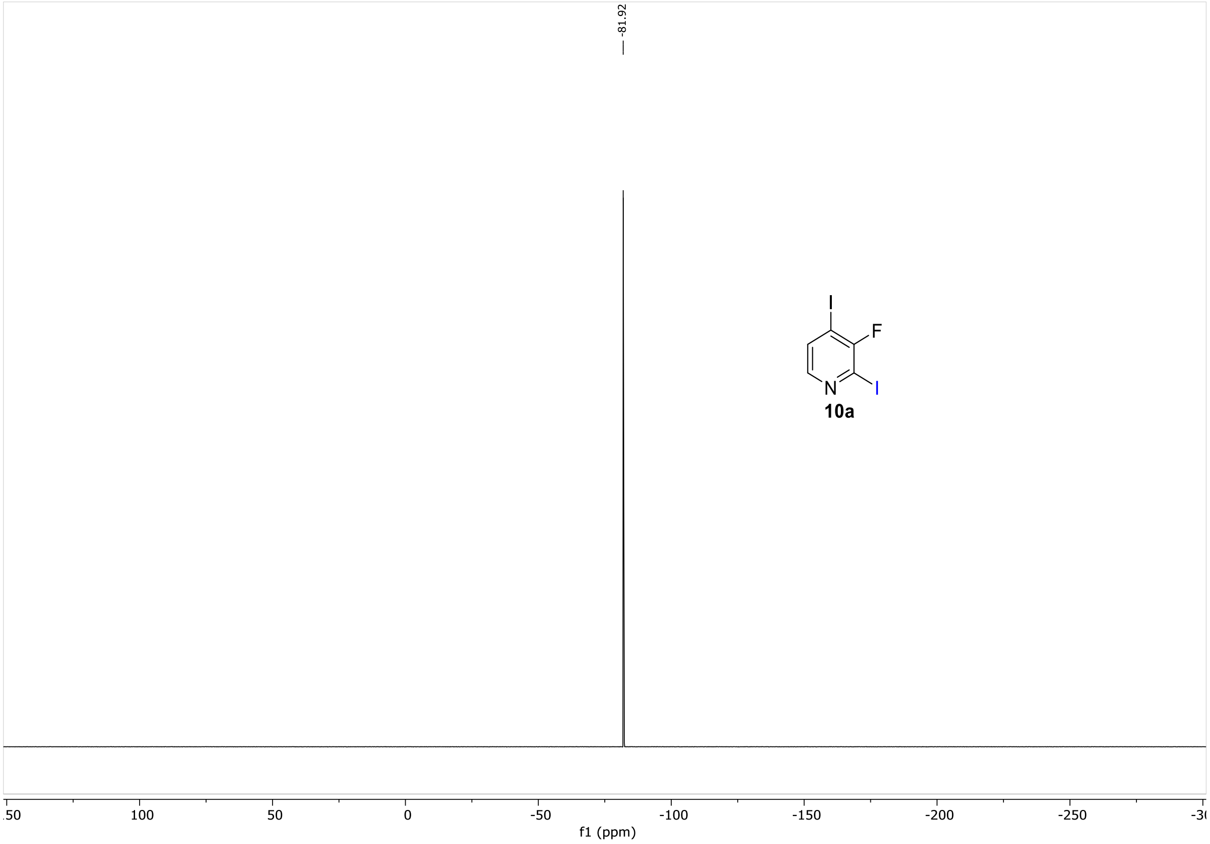
-81.92
I
F
N
I
10a
f1 (ppm)

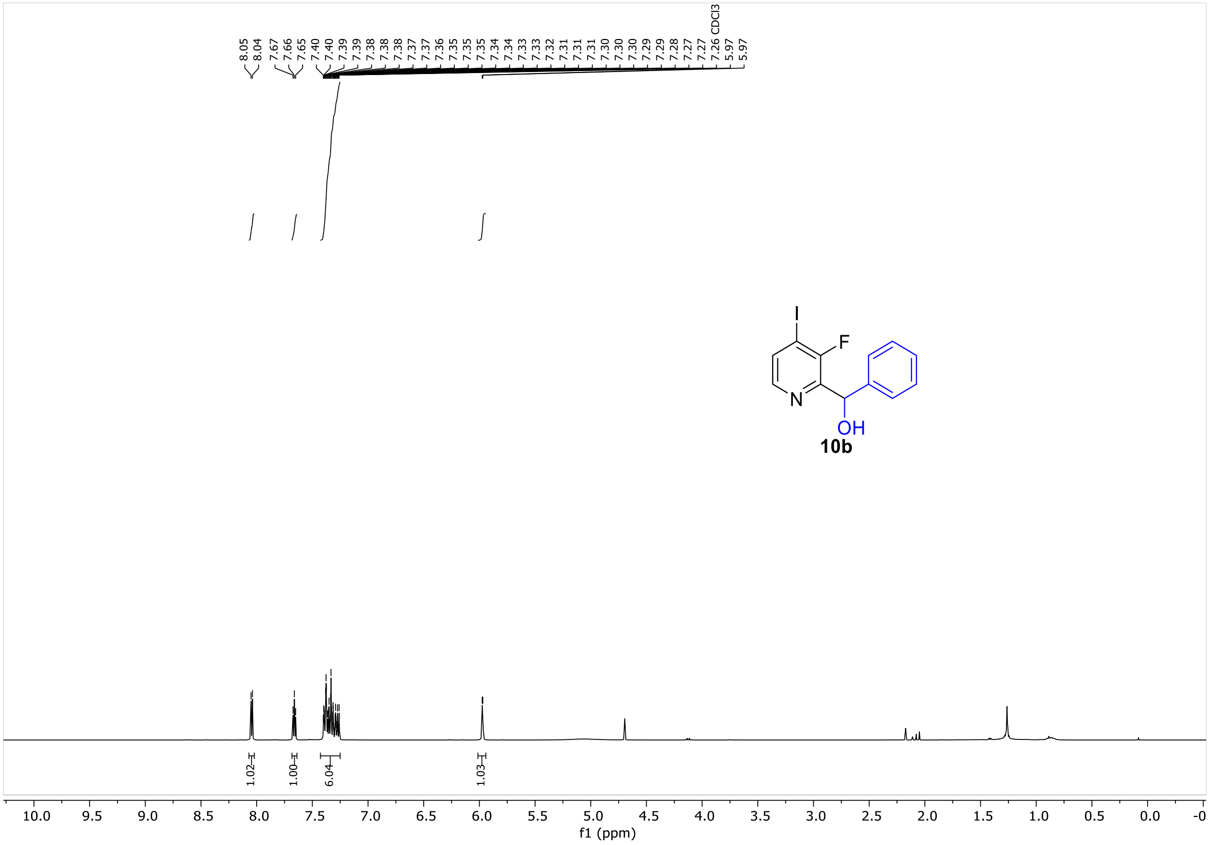
8.05
8.04
7.67
7.66
7.65
7.40
7.26 CDCl3
5.97
5.97
1.02
1.00
6.04
1.03
I
F
N
OH
10b
f1 (ppm)

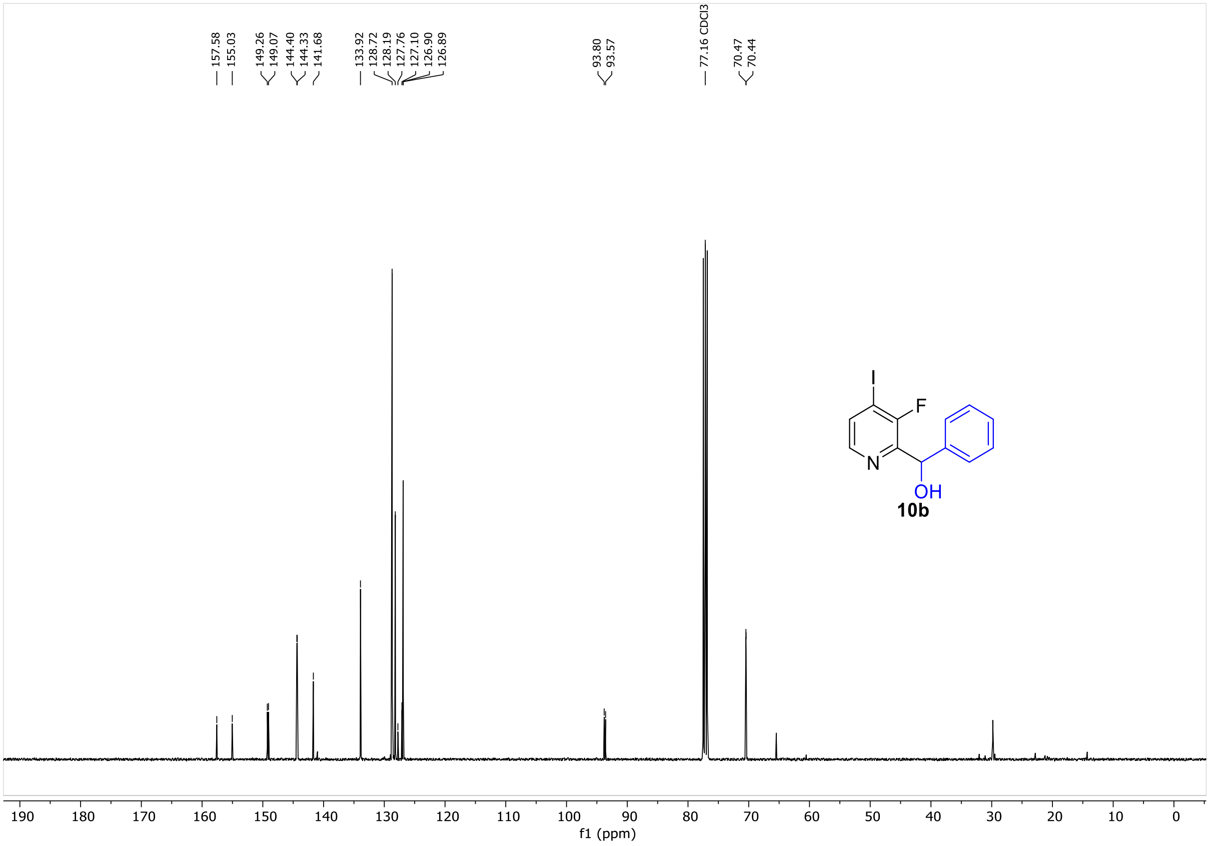
157.58
155.03
149.26
149.07
144.40
144.33
141.68
133.92
128.72
128.19
127.76
127.10
126.90
126.89
93.80
93.57
77.16 CDCl3
70.47
70.44
I
F
N
OH
10b
f1 (ppm)

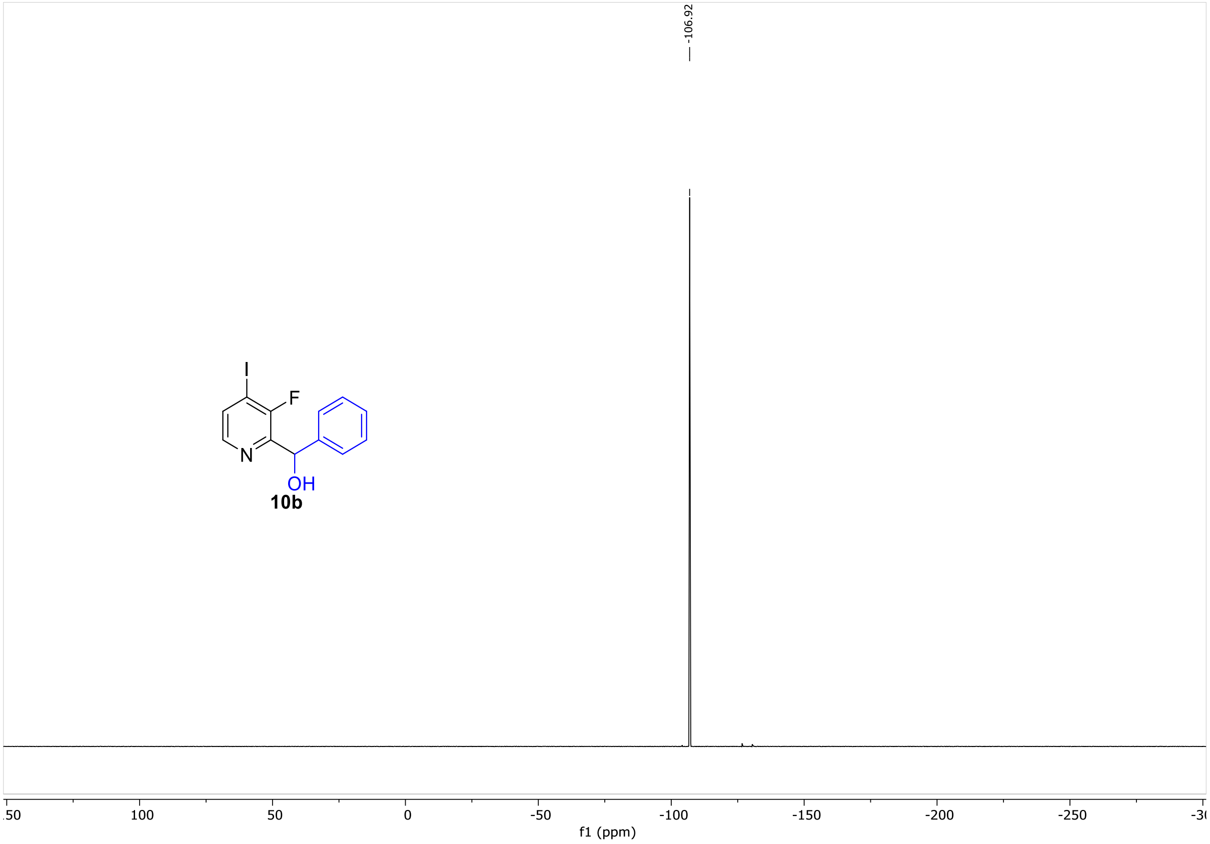
-106.92
I
F
N
OH
10b
f1 (ppm)

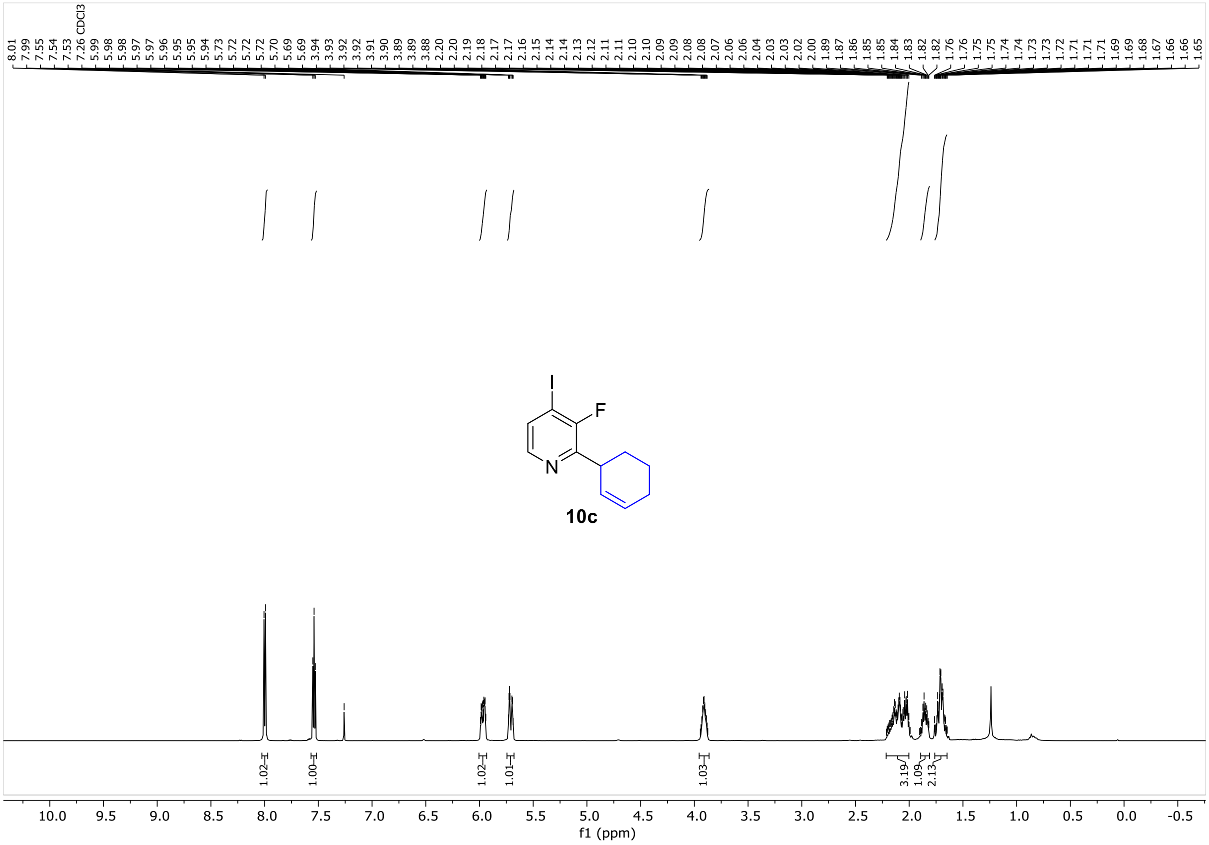
8.01 7.99 7.55 7.54 7.53 7.26 CDCl3 5.99 5.98 5.98 5.97 5.97 5.96 5.95 5.95 5.94 5.73 5.72 5.72 5.72 5.70 5.69 5.69 3.94 3.93 3.92 3.92 3.91 3.90 3.89 3.89 3.88 2.20 2.20 2.19 2.18 2.17 2.17 2.16 2.15 2.14 2.14 2.13 2.13 2.12 2.11 2.11 2.10 2.10 2.09 2.09 2.08 2.08 2.07 2.06 2.06 2.04 2.03 2.03 2.02 2.00 1.89 1.87 1.86 1.85 1.85 1.84 1.83 1.82 1.82 1.76 1.76 1.75 1.75 1.74 1.74 1.73 1.73 1.72 1.71 1.71 1.71 1.69 1.68 1.67 1.66 1.66 1.65
I
F
N
10c
1.02
1.00
1.02
1.01
1.03
3.19
1.09
2.13
10.0 9.5 9.0 8.5 8.0 7.5 7.0 6.5 6.0 5.5 5.0 4.5 4.0 3.5 3.0 2.5 2.0 1.5 1.0 0.5 0.0 -0.5
f1 (ppm)

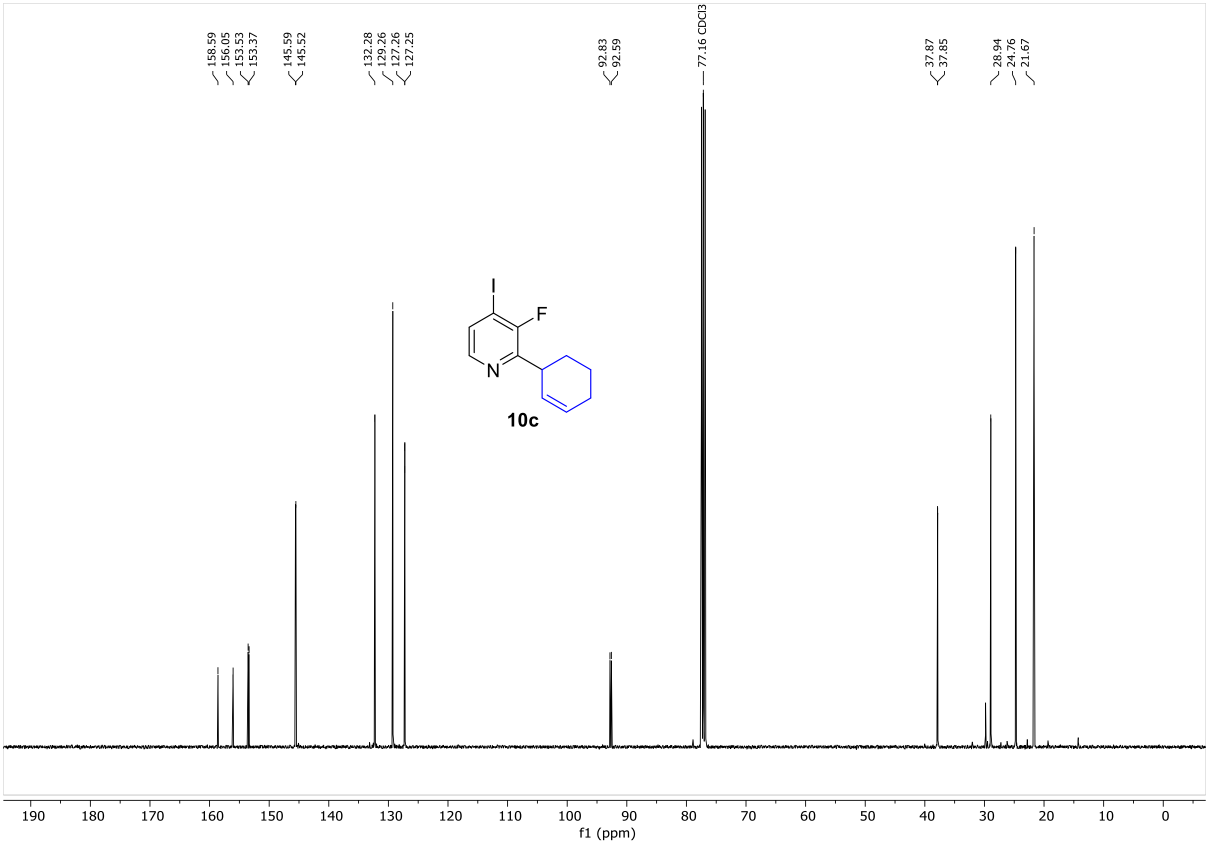
158.59 156.05 153.53 153.37 145.59 145.52 132.28 129.26 127.26 127.25 92.83 92.59 77.16 CDCl3 37.87 37.85 28.94 24.76 21.67
I
F
N
10c
190 180 170 160 150 140 130 120 110 100 90 80 70 60 50 40 30 20 10 0
f1 (ppm)

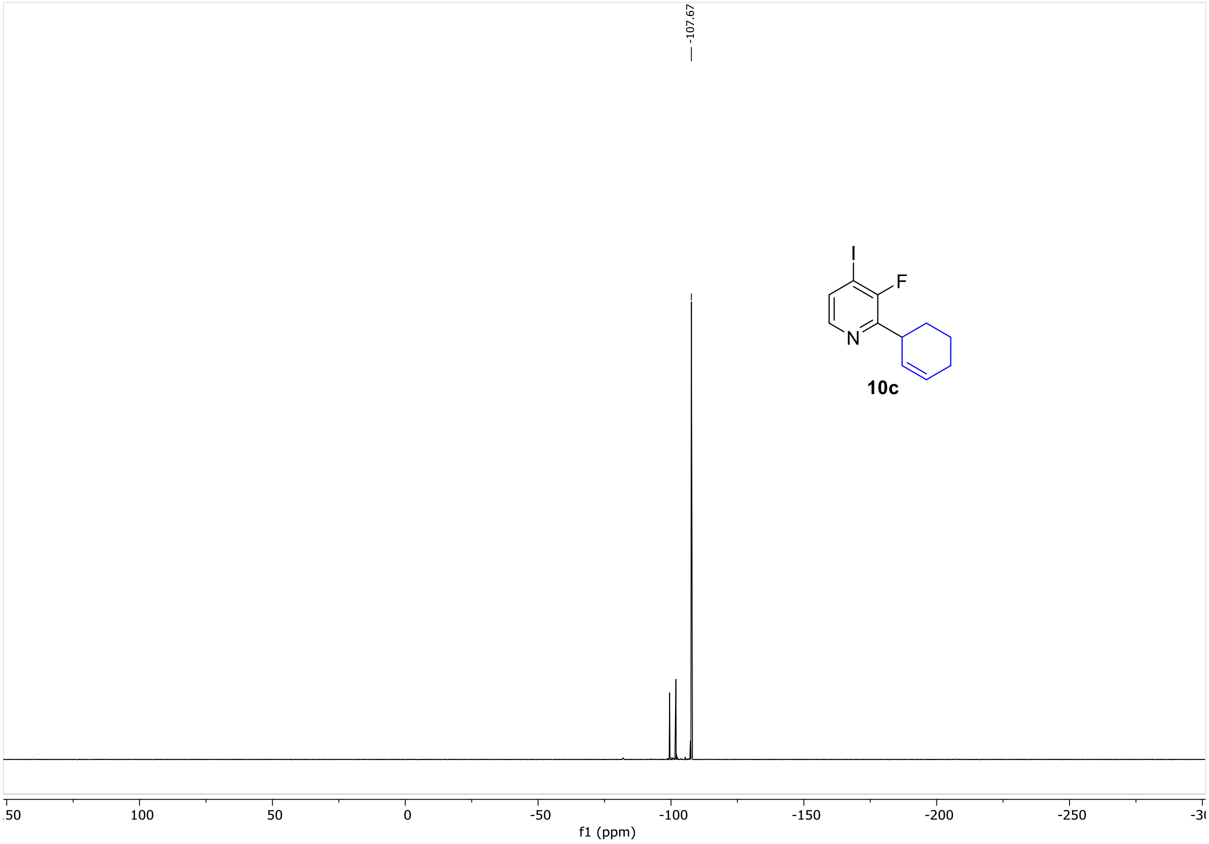
-107.67
I
F
N
150
100
50
0
-50
-100
-150
-200
-250
f1 (ppm)

10c

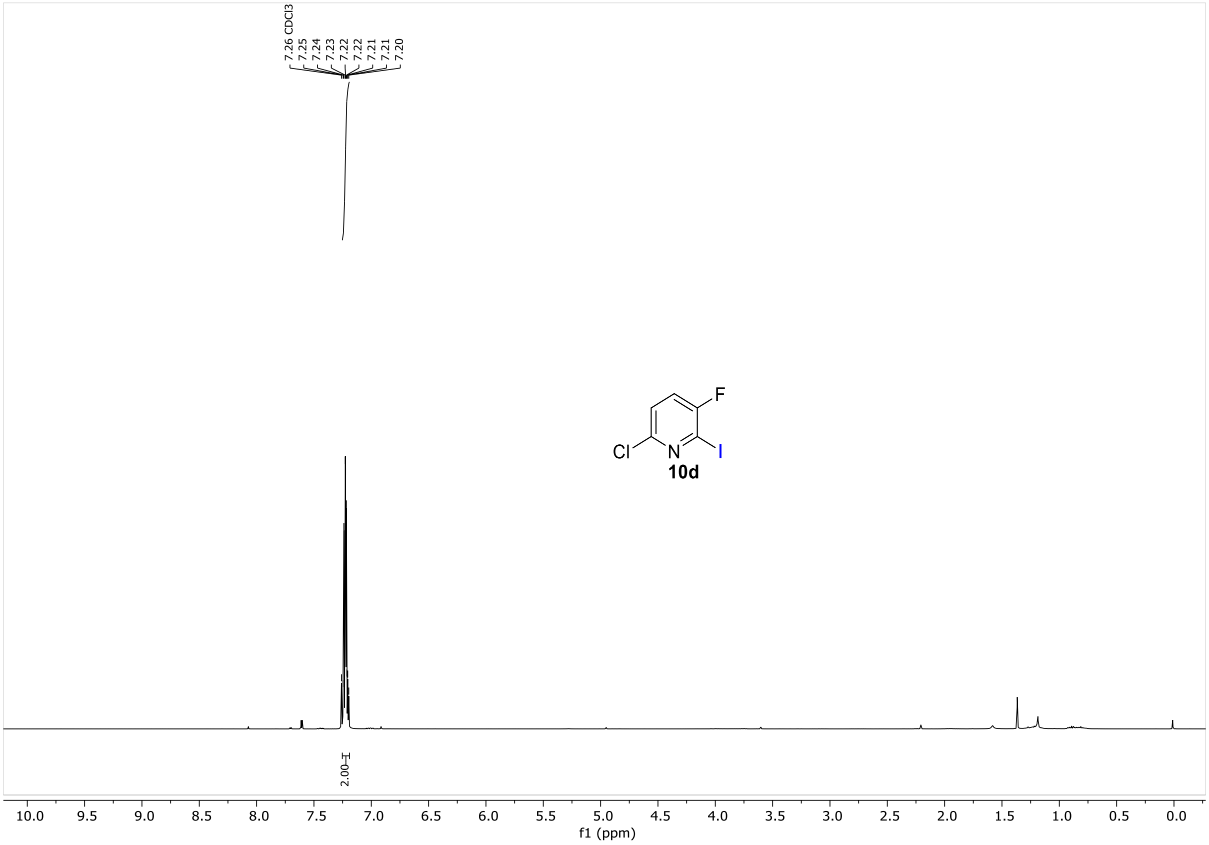
7.26 CDCl3
7.25
7.24
7.23
7.22
7.22
7.21
7.21
7.20
F
Cl
N
I
2.00
10.0
9.5
9.0
8.5
8.0
7.5
7.0
6.5
6.0
5.5
5.0
4.5
4.0
3.5
3.0
2.5
2.0
1.5
1.0
0.5
0.0
f1 (ppm)

10d

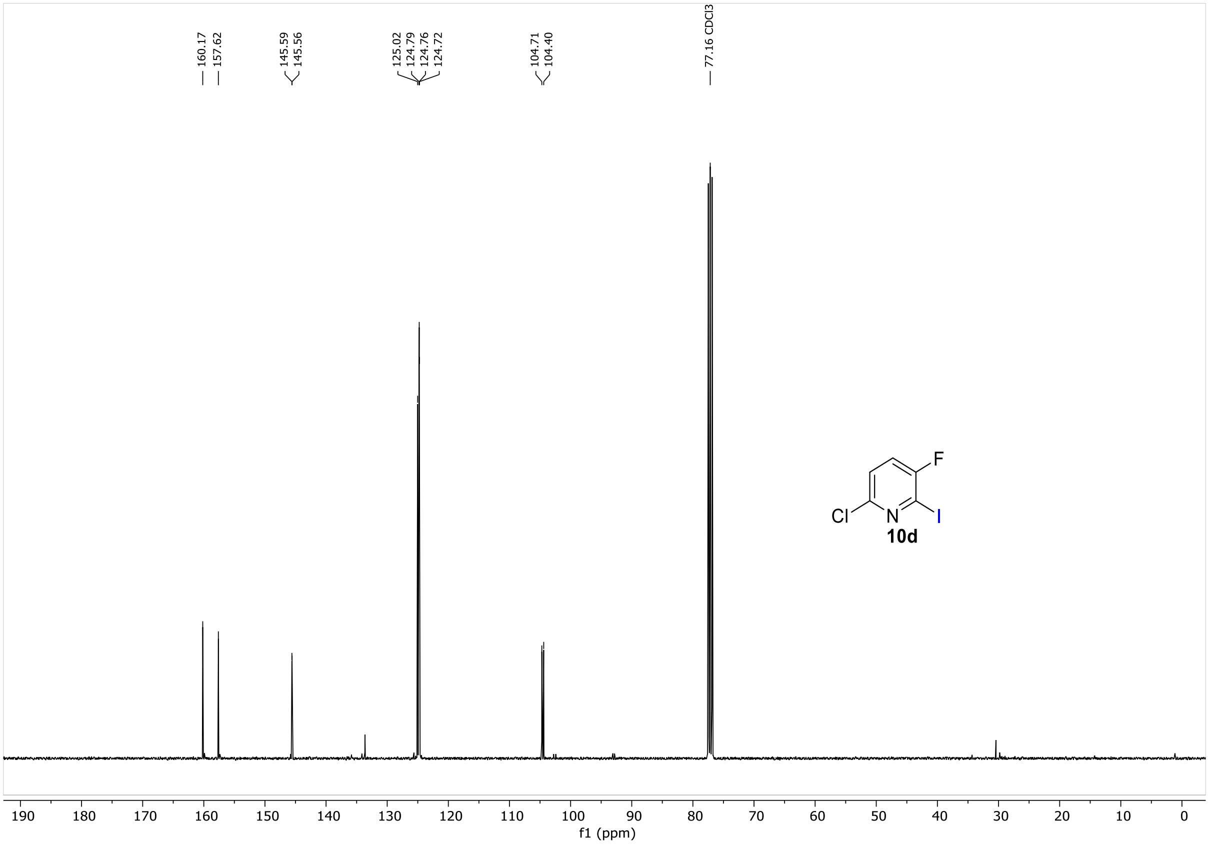
160.17
157.62
145.59
145.56
125.02
124.79
124.76
124.72
104.71
104.40
77.16 CDCl3
F
Cl
N
I
10d
190 180 170 160 150 140 130 120 110 100 90 80 70 60 50 40 30 20 10 0
f1 (ppm)

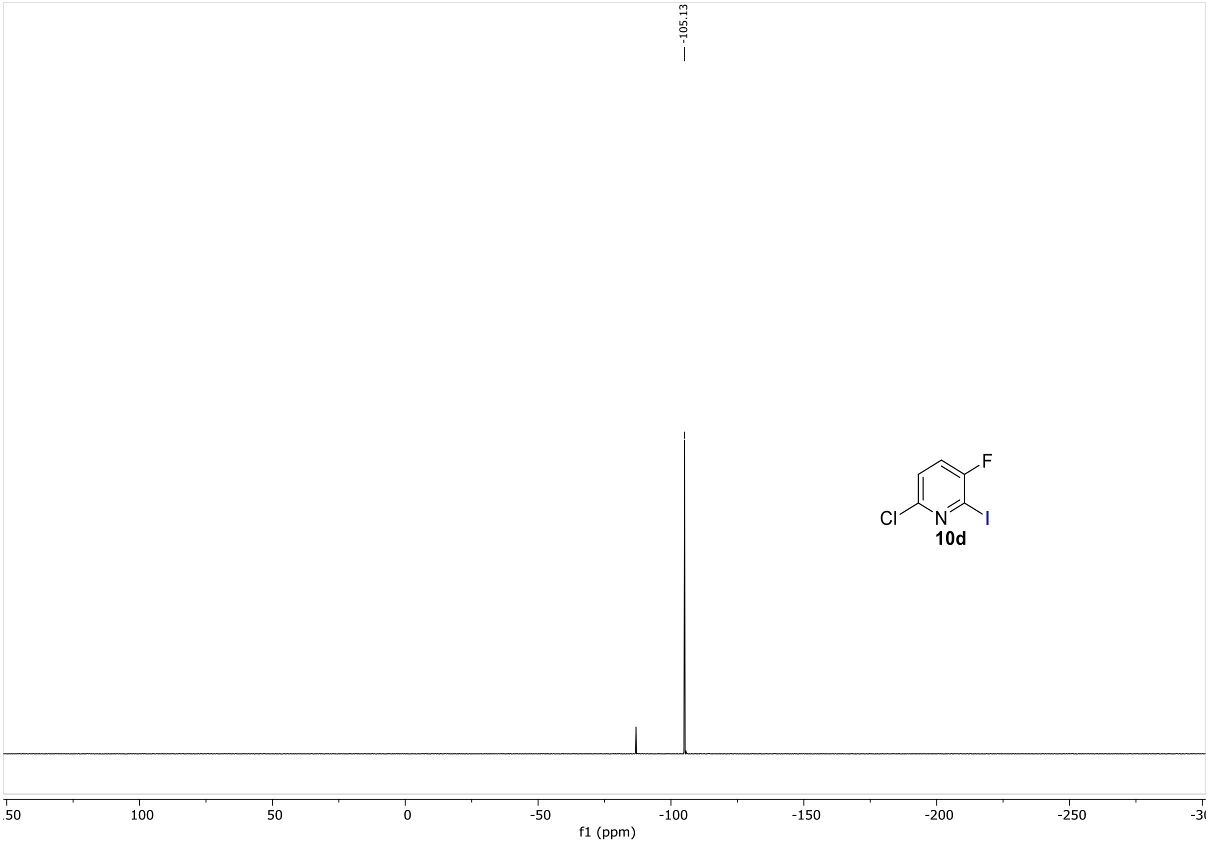
-105.13
F
Cl
N
I
10d
100 50 0 -50 -100 -150 -200 -250
f1 (ppm)

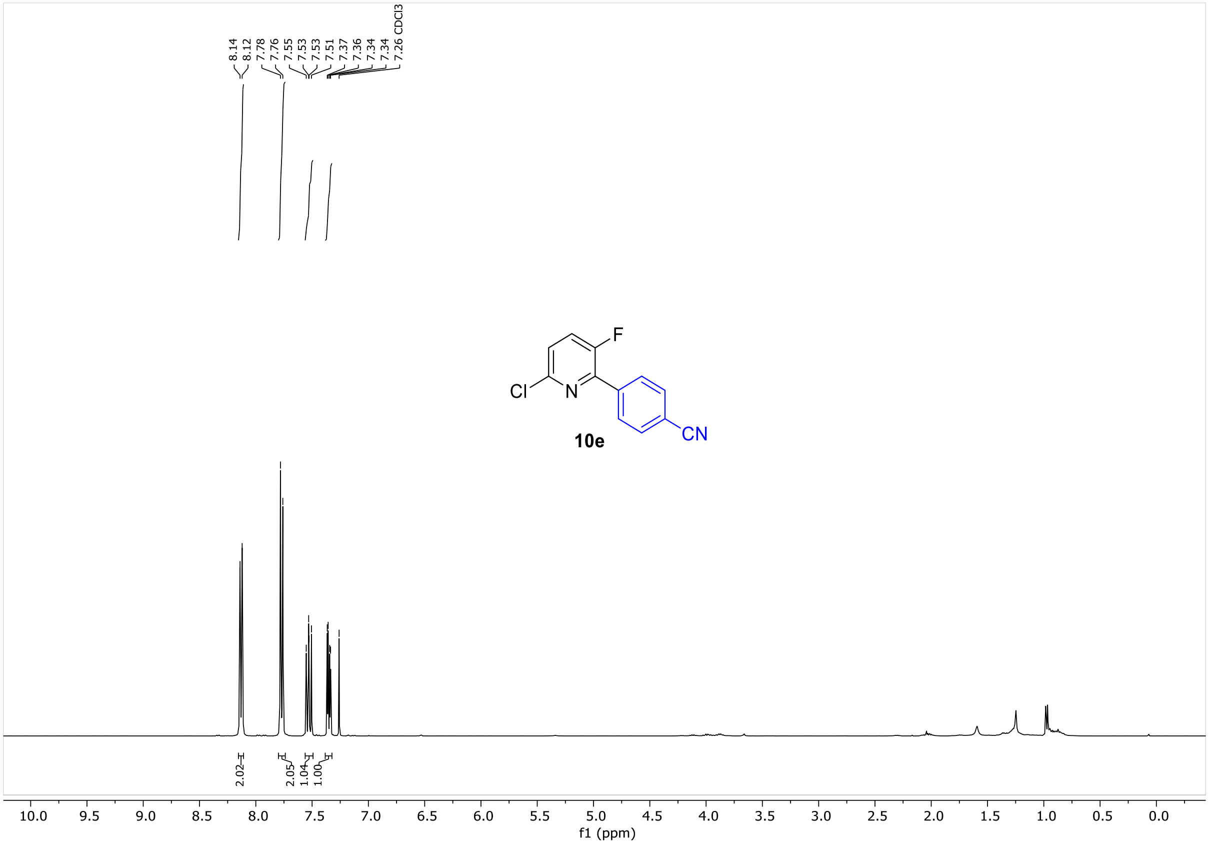
8.14
8.12
7.78
7.76
7.55
7.53
7.53
7.51
7.37
7.36
7.34
7.34
7.26 CDCl3
Cl
N
F
CN
10e
2.02
2.05
1.04
1.00
f1 (ppm)

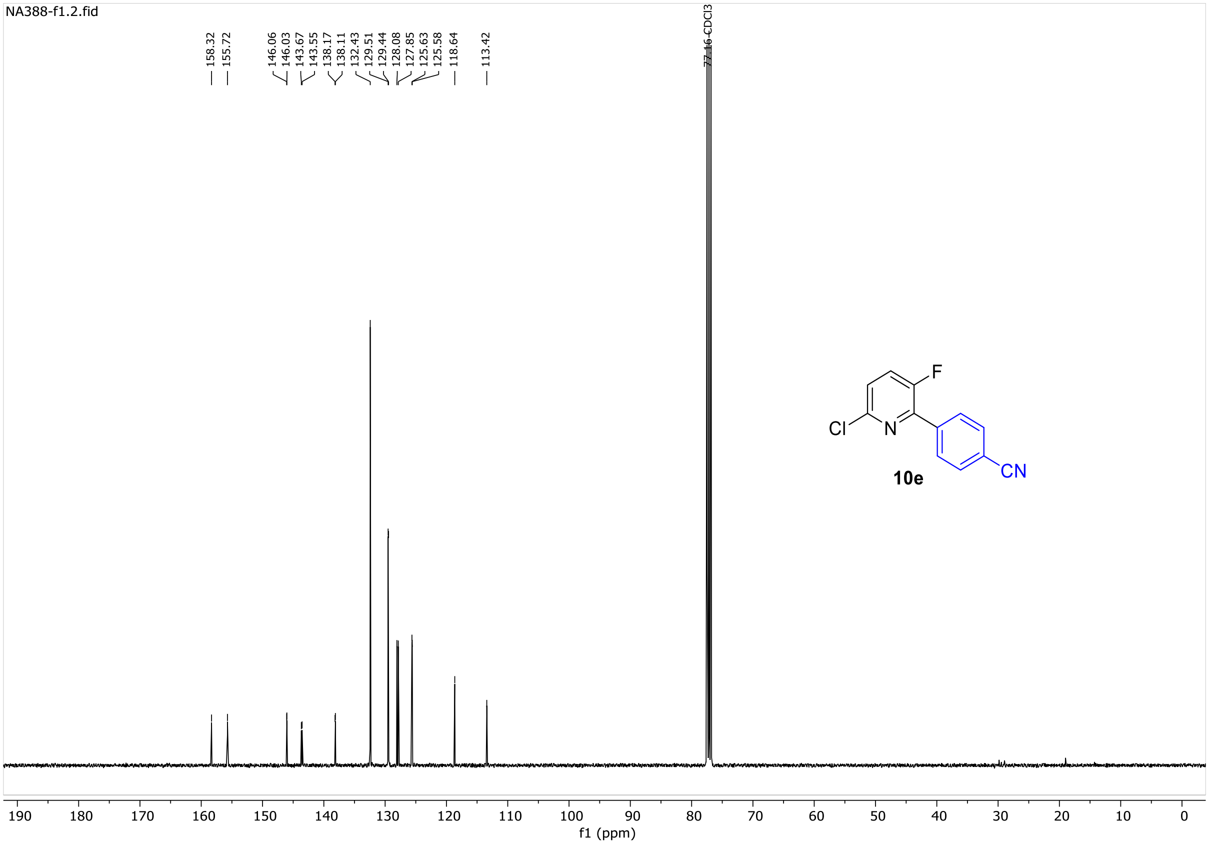
NA388-f1.2.fid
158.32
155.72
146.06
146.03
143.67
143.55
138.17
138.11
132.43
129.51
129.44
128.08
127.85
125.63
125.58
118.64
113.42
77.16 CDCl3
Cl
N
F
CN
10e
f1 (ppm)

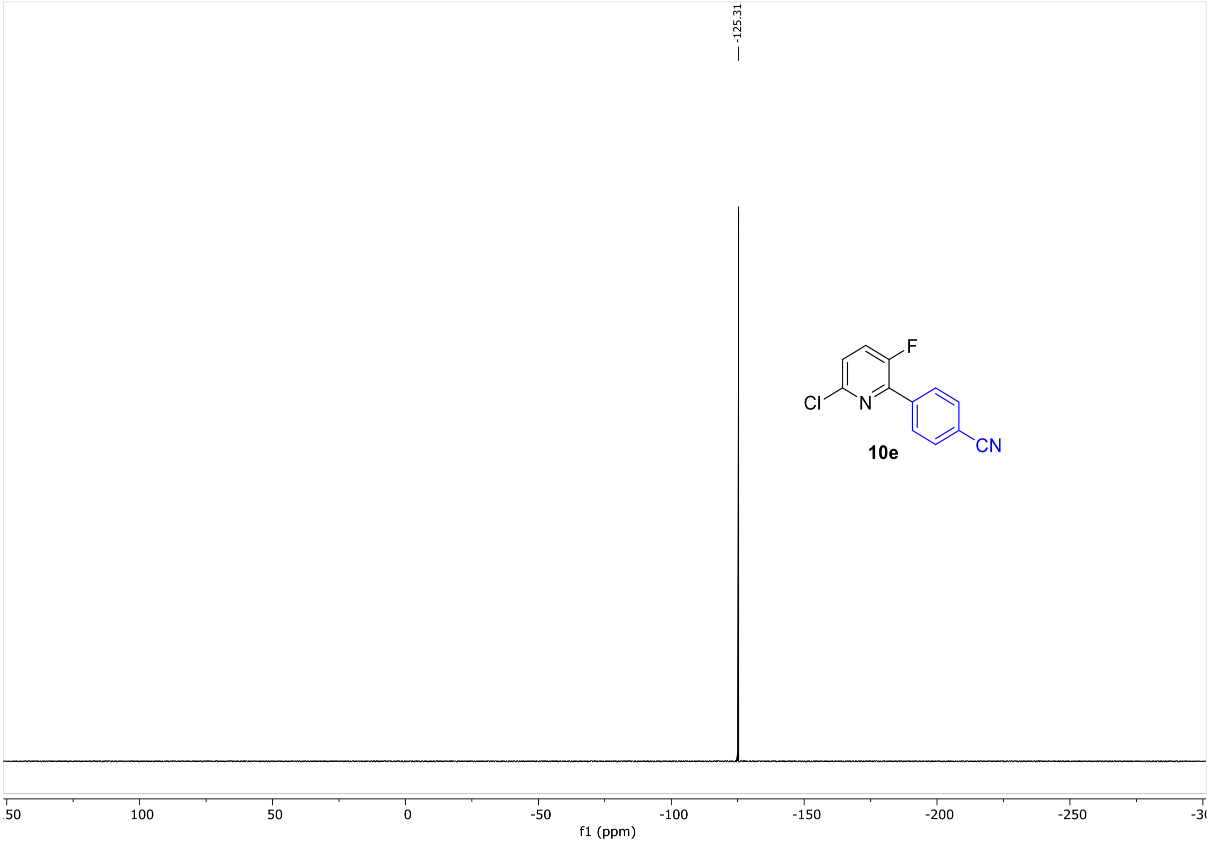
-125.31
F
Cl
N
CN
10e
150
100
50
0
-50
-100
-150
-200
-250
-300
f1 (ppm)

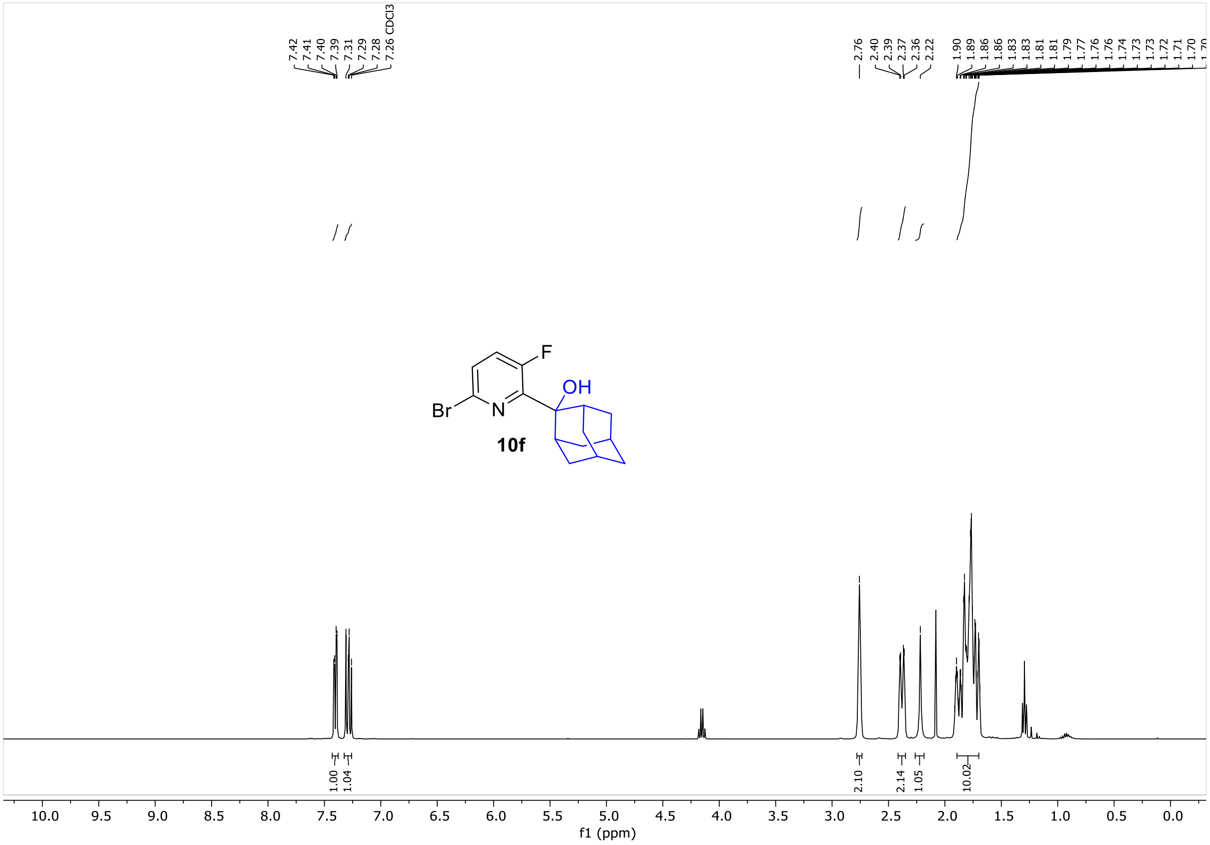
7.42
7.41
7.40
7.39
7.31
7.29
7.28
7.26 CDCl3
2.76
2.40
2.39
2.37
2.36
2.22
1.90
1.89
1.86
1.86
1.83
1.83
1.81
1.81
1.79
1.77
1.76
1.76
1.74
1.73
1.73
1.72
1.71
1.70
F
OH
Br
N
10f
1.00
1.04
2.10
2.14
1.05
10.02
10.0
9.5
9.0
8.5
8.0
7.5
7.0
6.5
6.0
5.5
5.0
4.5
4.0
3.5
3.0
2.5
2.0
1.5
1.0
0.5
0.0
f1 (ppm)

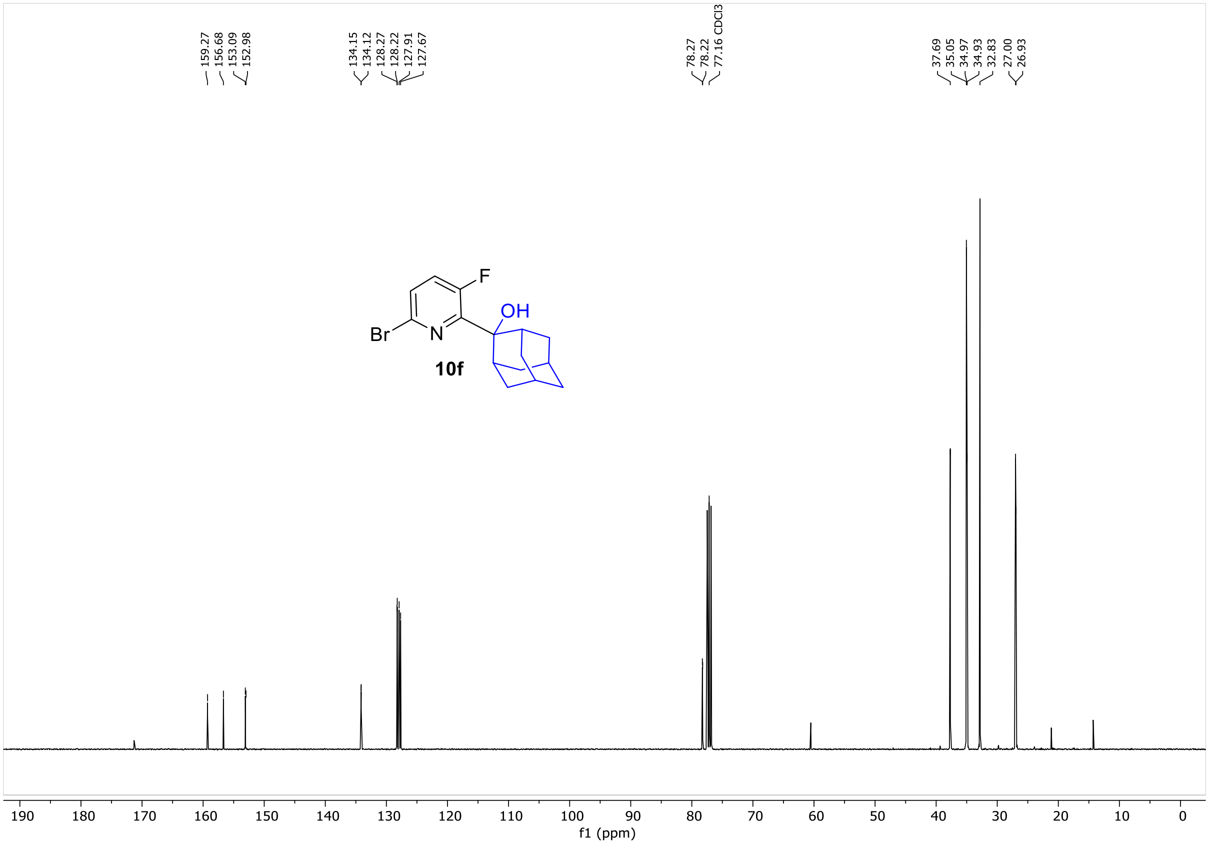

159.27
156.68
153.09
152.98
134.15
134.12
128.27
128.22
127.91
127.67
78.27
78.22
77.16 CDCl3
37.69
35.05
34.97
34.93
32.83
27.00
26.93
F
OH
Br
N
10f
190
180
170
160
150
140
130
120
110
100
90
80
70
60
50
40
30
20
10
0
f1 (ppm)

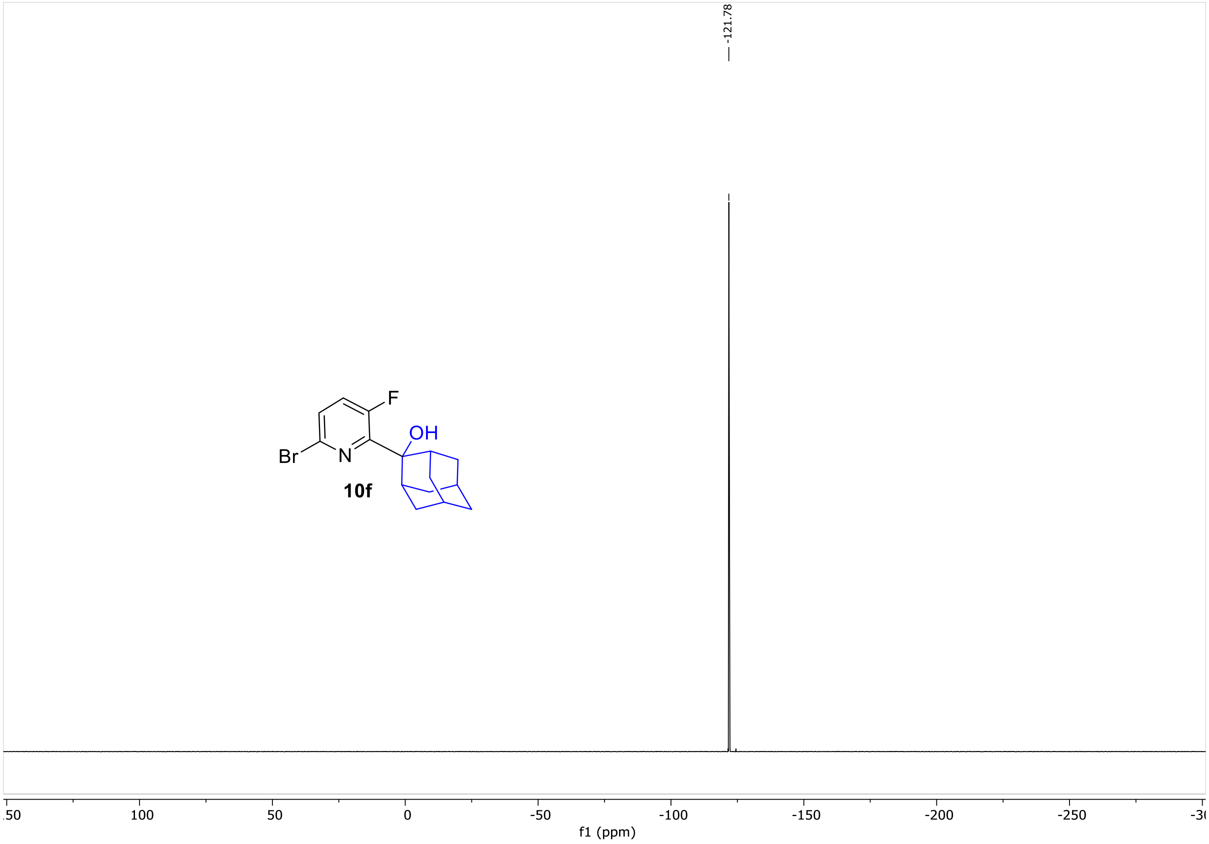

-121.78
F
OH
Br
N
10f
100
50
0
-50
-100
-150
-200
-250
f1 (ppm)

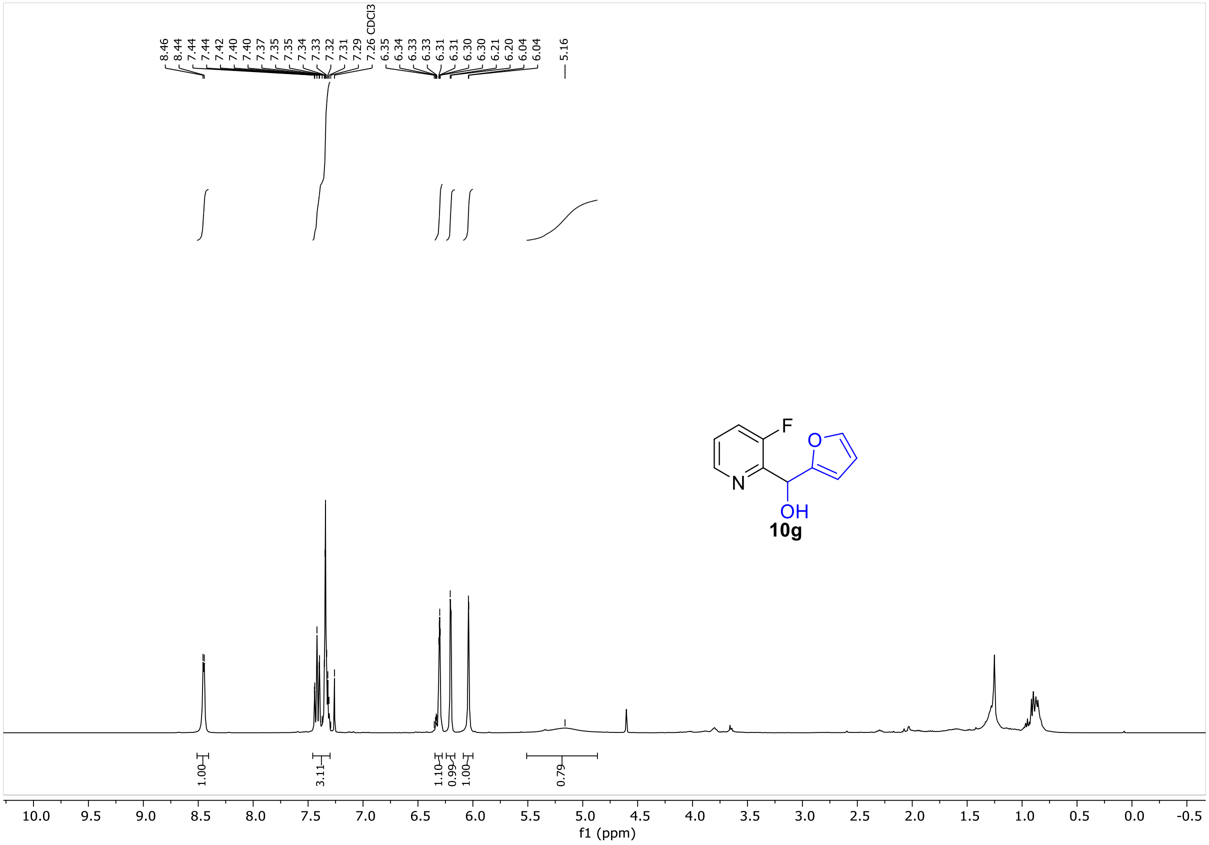
8.46
8.44
7.44
7.42
7.40
7.37
7.35
7.35
7.34
7.33
7.32
7.31
7.29
7.26 CDCl3
6.35
6.34
6.33
6.33
6.31
6.31
6.30
6.30
6.21
6.20
6.04
6.04
5.16
F
O
N
OH
10g
1.00
3.11
1.10
0.99
1.00
0.79
10.0 9.5 9.0 8.5 8.0 7.5 7.0 6.5 6.0 5.5 5.0 4.5 4.0 3.5 3.0 2.5 2.0 1.5 1.0 0.5 0.0 -0.5
f1 (ppm)

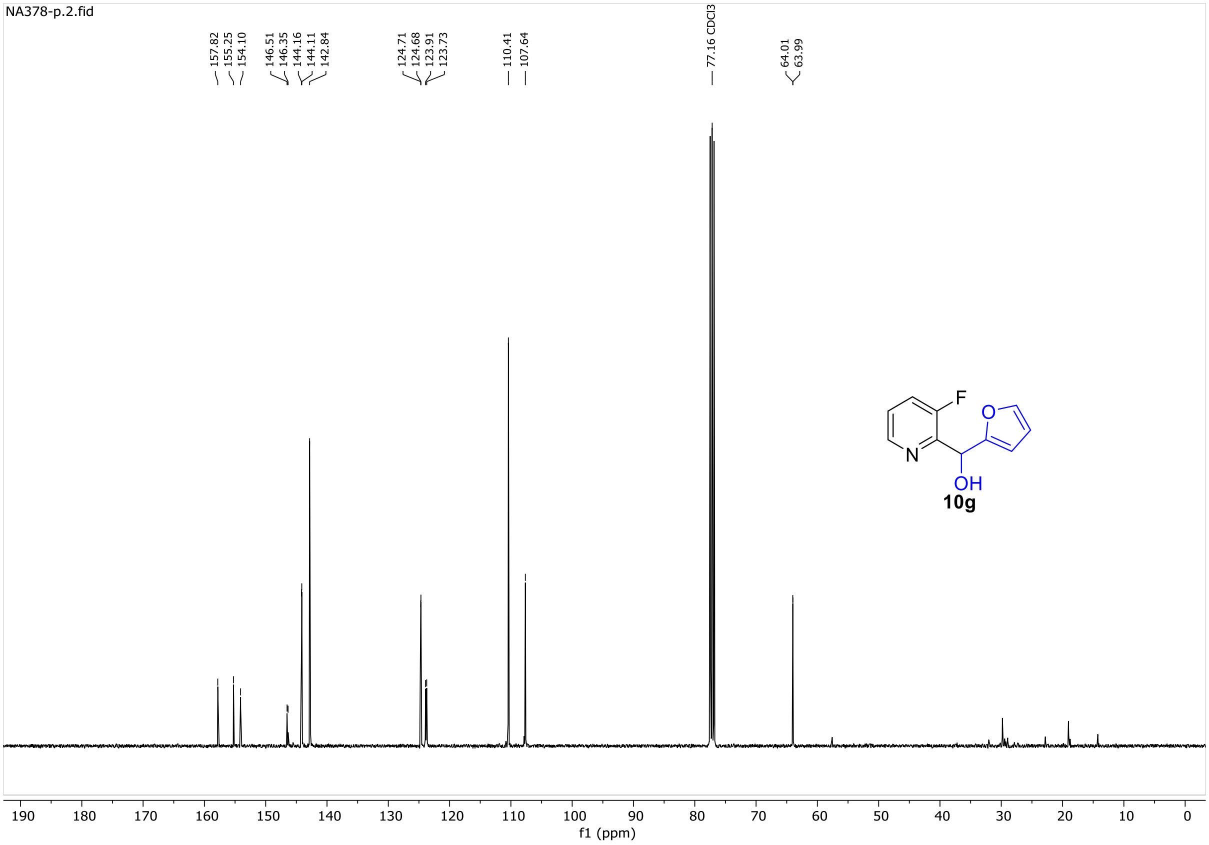
NA378-p.2.fid
157.82
155.25
154.10
146.51
146.35
144.16
144.11
142.84
124.71
124.68
123.91
123.73
110.41
107.64
77.16 CDCl3
64.01
63.99
F
O
N
OH
10g
190 180 170 160 150 140 130 120 110 100 90 80 70 60 50 40 30 20 10 0
f1 (ppm)

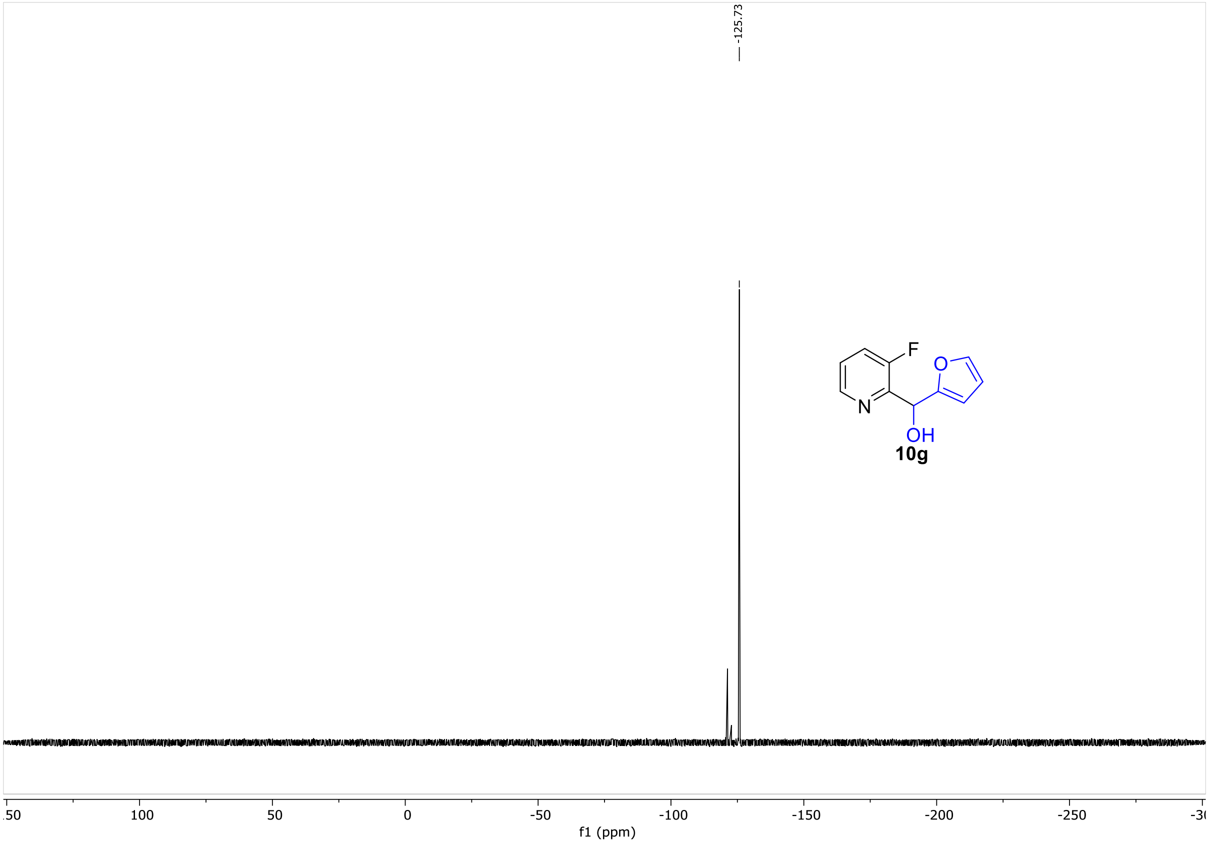
-125.73
F
O
N
OH
10g
150
100
50
0
-50
-100
-150
-200
-250
f1 (ppm)

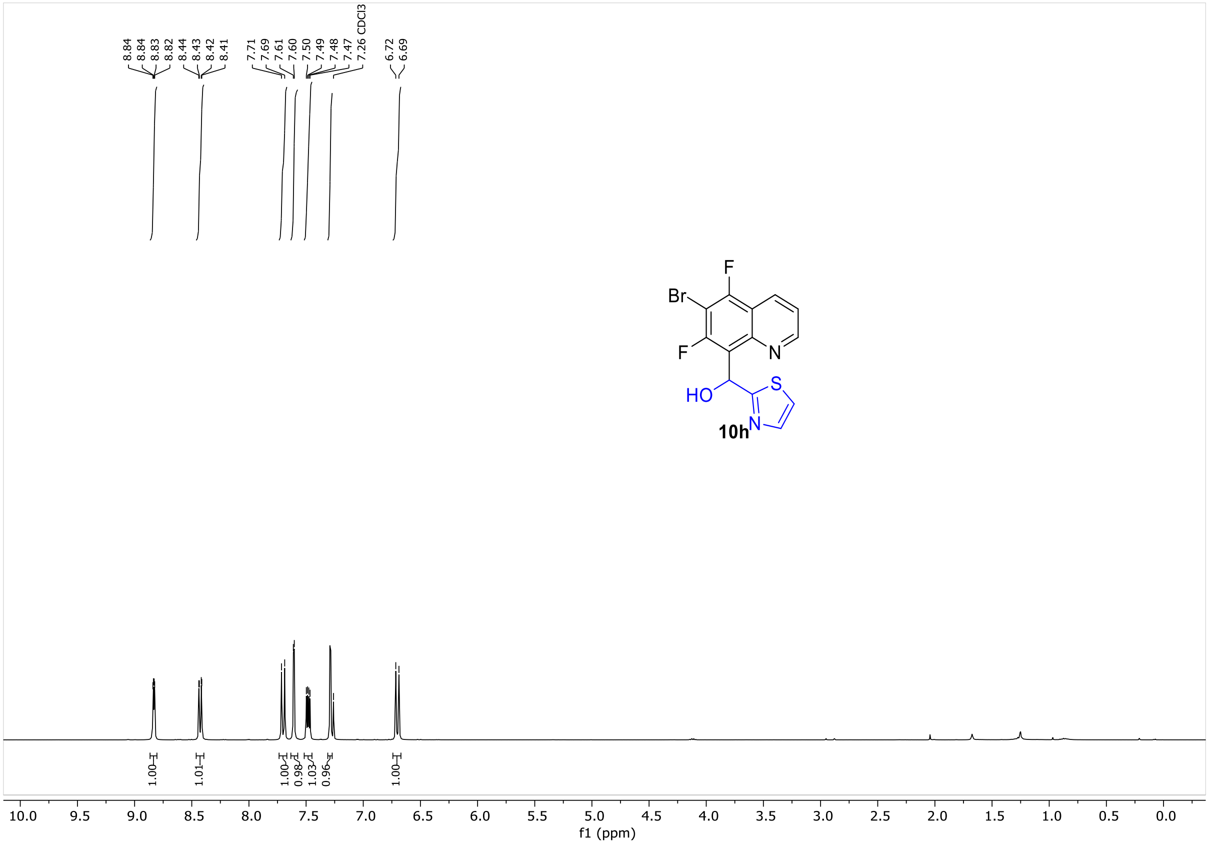
8.84
8.84
8.83
8.82
8.44
8.43
8.42
8.41
7.71
7.69
7.61
7.60
7.50
7.49
7.48
7.47
7.26 CDCl3
6.72
6.69
F
Br
F
N
HO
S
N
10h
1.00
1.01
1.00
0.98
1.03
0.96
1.00
10.0
9.5
9.0
8.5
8.0
7.5
7.0
6.5
6.0
5.5
5.0
4.5
4.0
3.5
3.0
2.5
2.0
1.5
1.0
0.5
0.0
f1 (ppm)

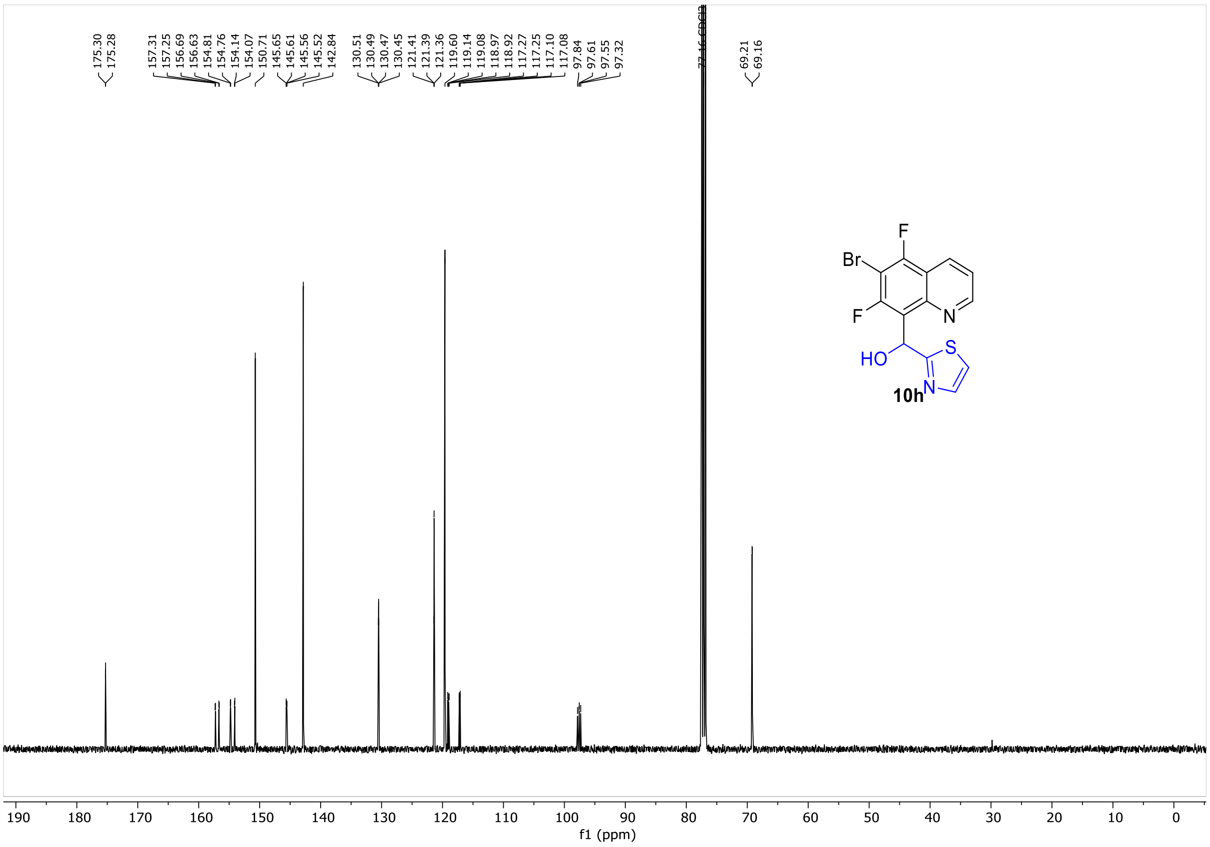
175.30
175.28
157.31
157.25
156.69
156.63
154.81
154.76
154.14
154.07
150.71
145.65
145.61
145.56
145.52
142.84
130.51
130.49
130.47
130.45
121.41
121.39
121.36
119.60
119.14
119.08
118.97
118.92
117.27
117.25
117.10
117.08
97.84
97.61
97.55
97.32
69.21
69.16
Br
F
F
N
HO
S
N
10h
f1 (ppm)

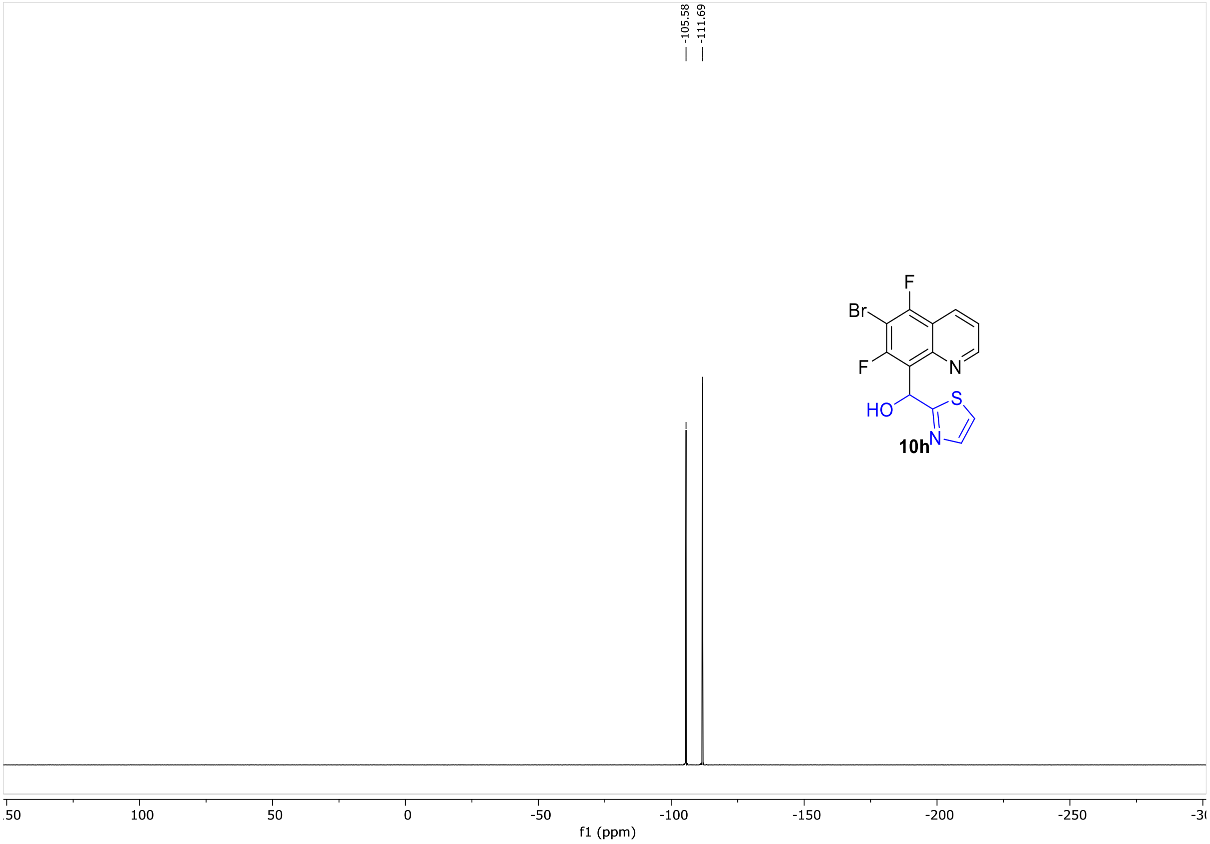
-105.58
-111.69
Br
F
F
N
HO
S
N
10h
f1 (ppm)

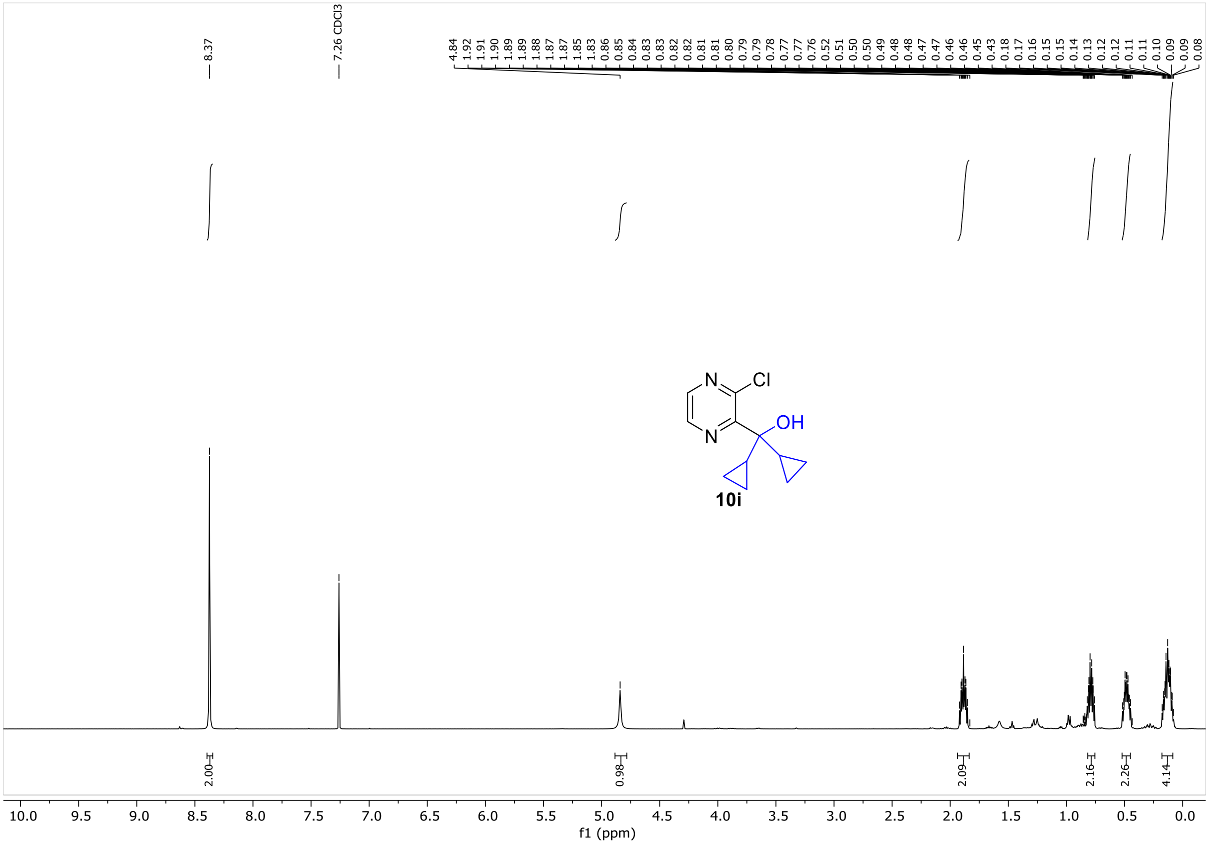
8.37
7.26 CDCl3
4.84 1.92 1.91 1.90 1.89 1.89 1.88 1.87 1.87 1.85 1.83 0.86 0.85 0.84 0.83 0.83 0.82 0.82 0.81 0.81 0.80 0.79 0.79 0.78 0.77 0.77 0.76 0.52 0.51 0.50 0.50 0.49 0.48 0.48 0.47 0.47 0.46 0.46 0.45 0.43 0.18 0.17 0.16 0.15 0.15 0.14 0.13 0.13 0.12 0.12 0.11 0.11 0.10 0.09 0.09 0.08
N
Cl
OH
N
10i
2.00
0.98
2.09
2.16
2.26
4.14
10.0 9.5 9.0 8.5 8.0 7.5 7.0 6.5 6.0 5.5 5.0 4.5 4.0 3.5 3.0 2.5 2.0 1.5 1.0 0.5 0.0
f1 (ppm)

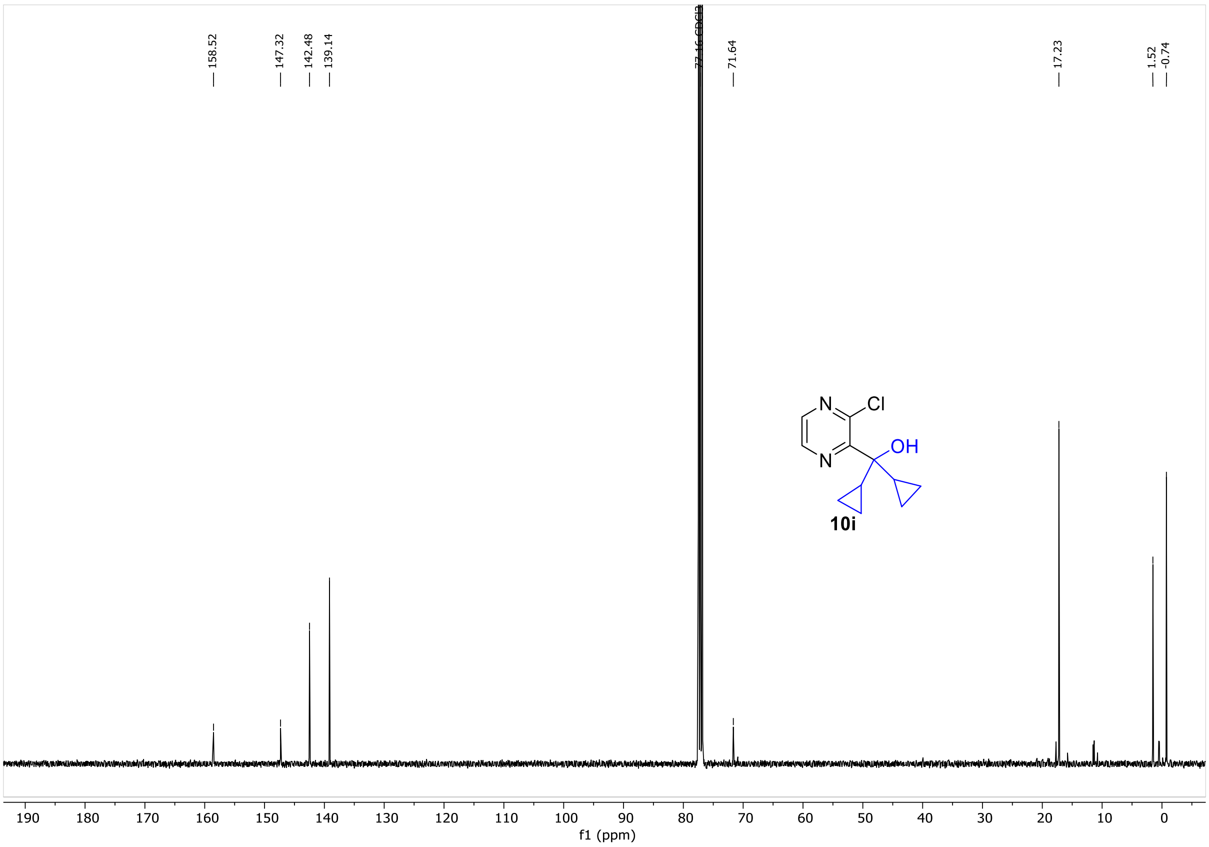
158.52
147.32
142.48
139.14
71.64
17.23
1.52
-0.74
N
Cl
OH
N
10i
190 180 170 160 150 140 130 120 110 100 90 80 70 60 50 40 30 20 10 0
f1 (ppm)

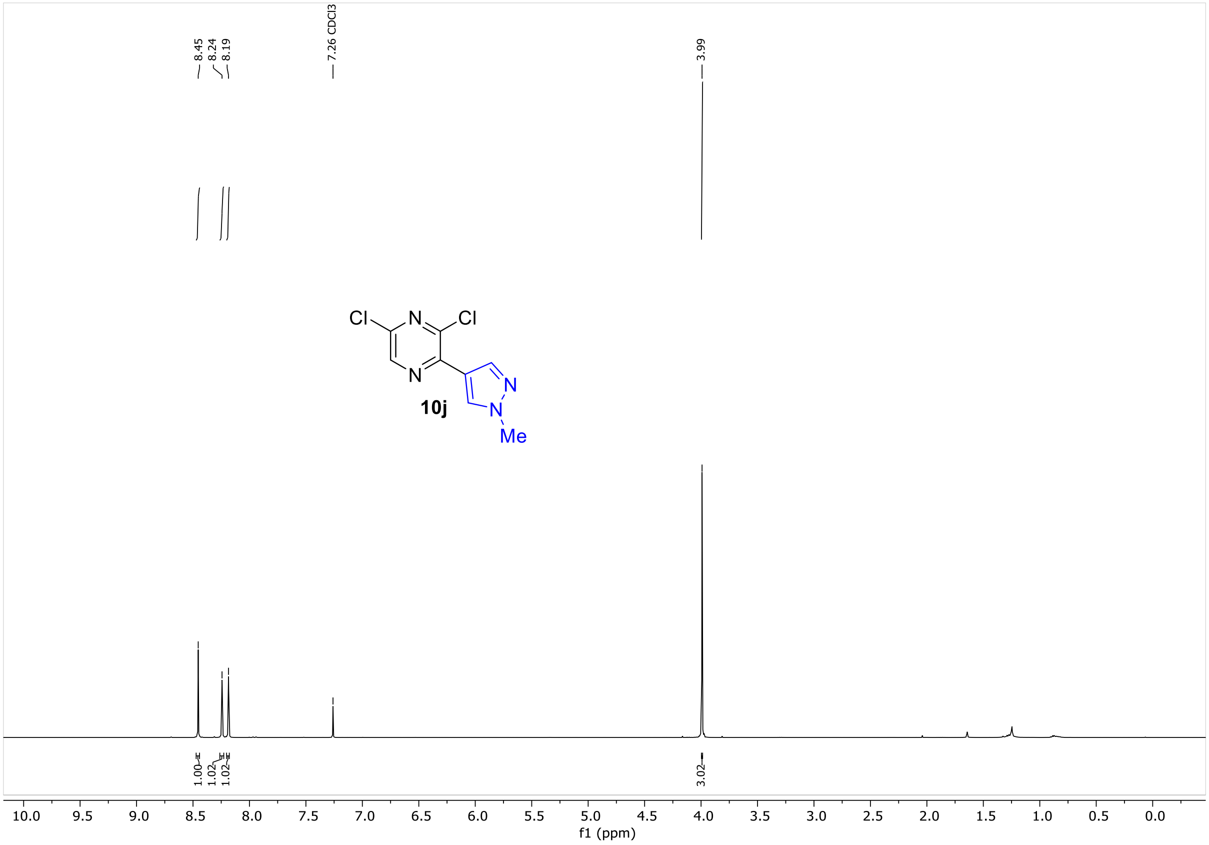
8.45
8.24
8.19
7.26 CDCl3
3.99
Cl
N
Cl
N
N
N
Me
10j
1.00
1.02
1.02
3.02
10.0 9.5 9.0 8.5 8.0 7.5 7.0 6.5 6.0 5.5 5.0 4.5 4.0 3.5 3.0 2.5 2.0 1.5 1.0 0.5 0.0
f1 (ppm)

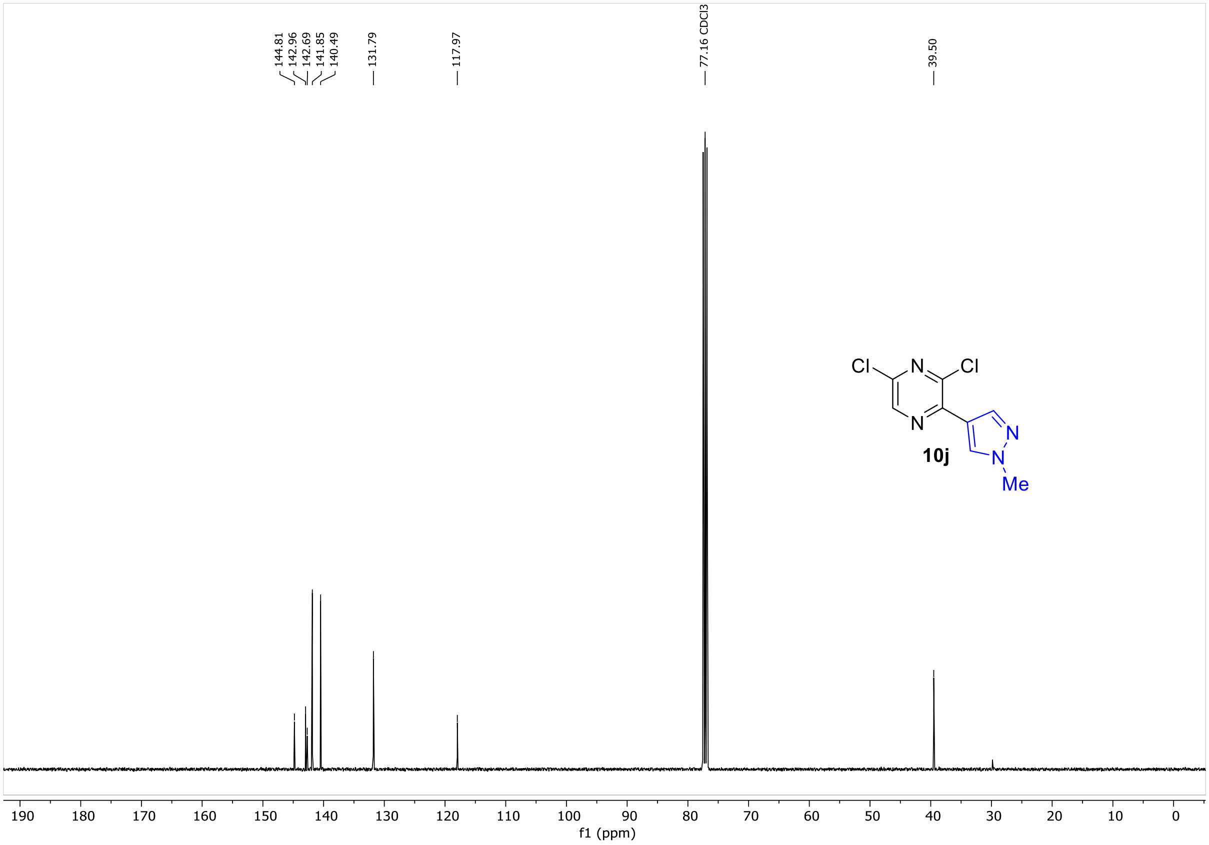
144.81
142.96
142.69
141.85
140.49
131.79
117.97
77.16 CDCl3
39.50
Cl
N
Cl
N
N
N
Me
10j
190 180 170 160 150 140 130 120 110 100 90 80 70 60 50 40 30 20 10 0
f1 (ppm)

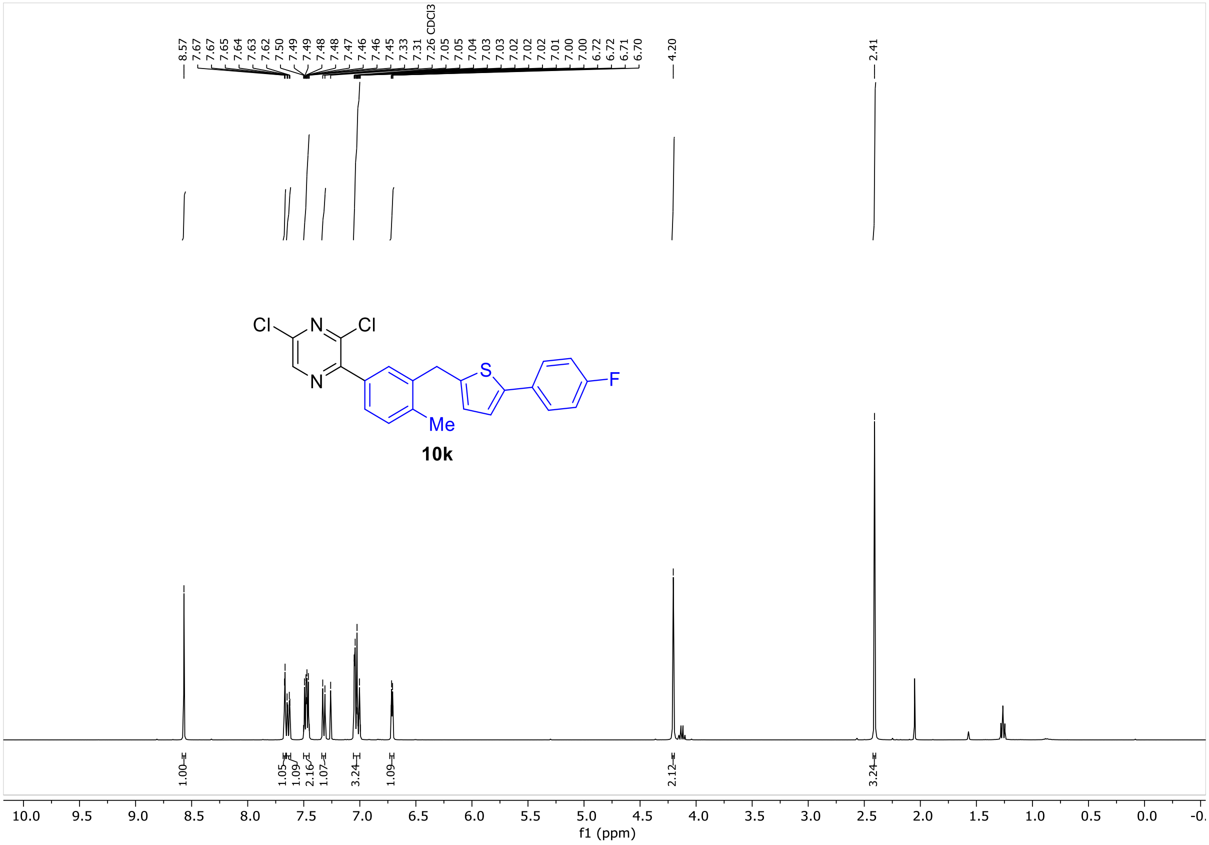
8.57 7.67 7.65 7.64 7.63 7.62 7.50 7.49 7.49 7.48 7.48 7.47 7.46 7.46 7.45 7.33 7.31 7.26 CDCl3 7.05 7.05 7.04 7.03 7.03 7.02 7.02 7.02 7.01 7.00 7.00 6.72 6.72 6.71 6.70
4.20
2.41
Cl N Cl N S F Me
10k
1.00 1.05 1.09 2.16 1.07 3.24 1.09 2.12 3.24
10.0 9.5 9.0 8.5 8.0 7.5 7.0 6.5 6.0 5.5 5.0 4.5 4.0 3.5 3.0 2.5 2.0 1.5 1.0 0.5 0.0
f1 (ppm)

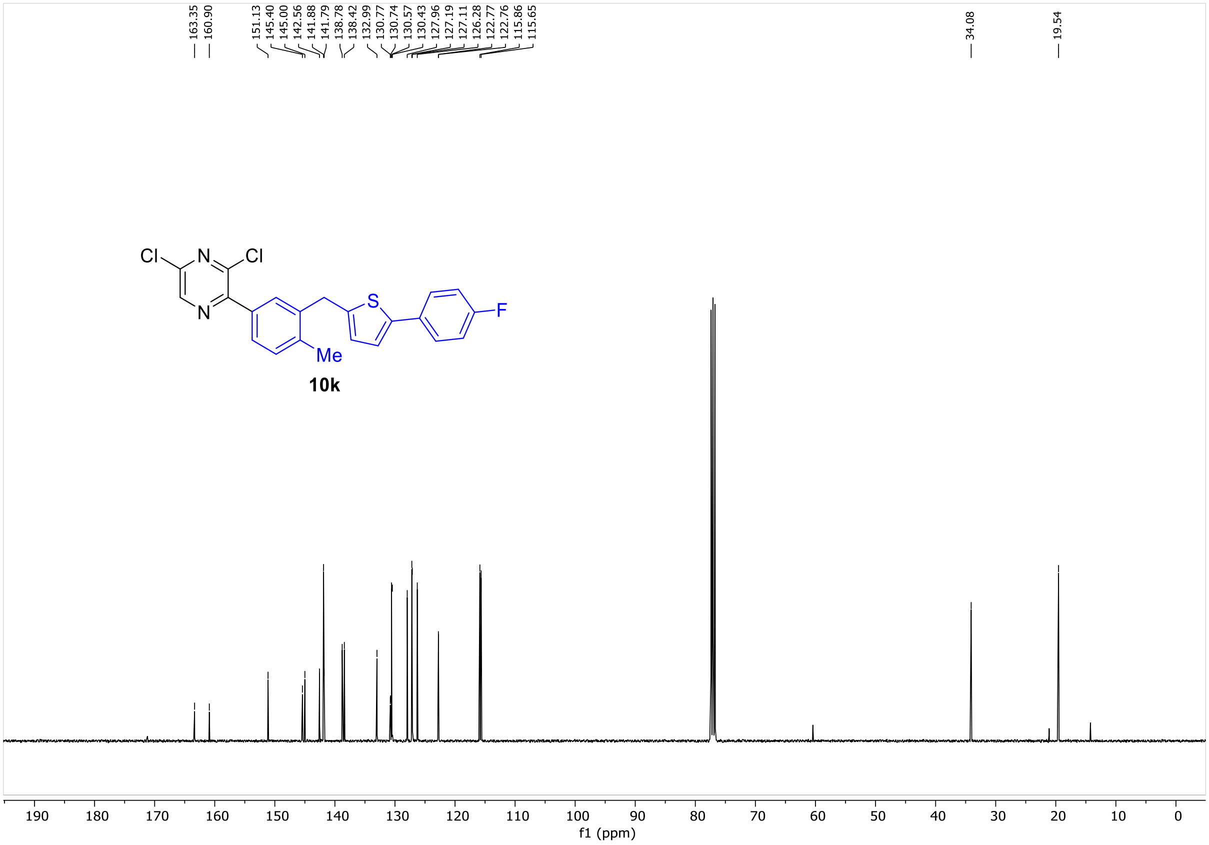
163.35 160.90 151.13 145.40 145.00 142.56 141.88 141.79 138.78 138.42 132.99 130.77 130.74 130.57 130.43 127.96 127.19 127.11 126.28 122.77 122.76 115.86 115.65
34.08
19.54
Cl N Cl N S F Me
10k
190 180 170 160 150 140 130 120 110 100 90 80 70 60 50 40 30 20 10 0
f1 (ppm)

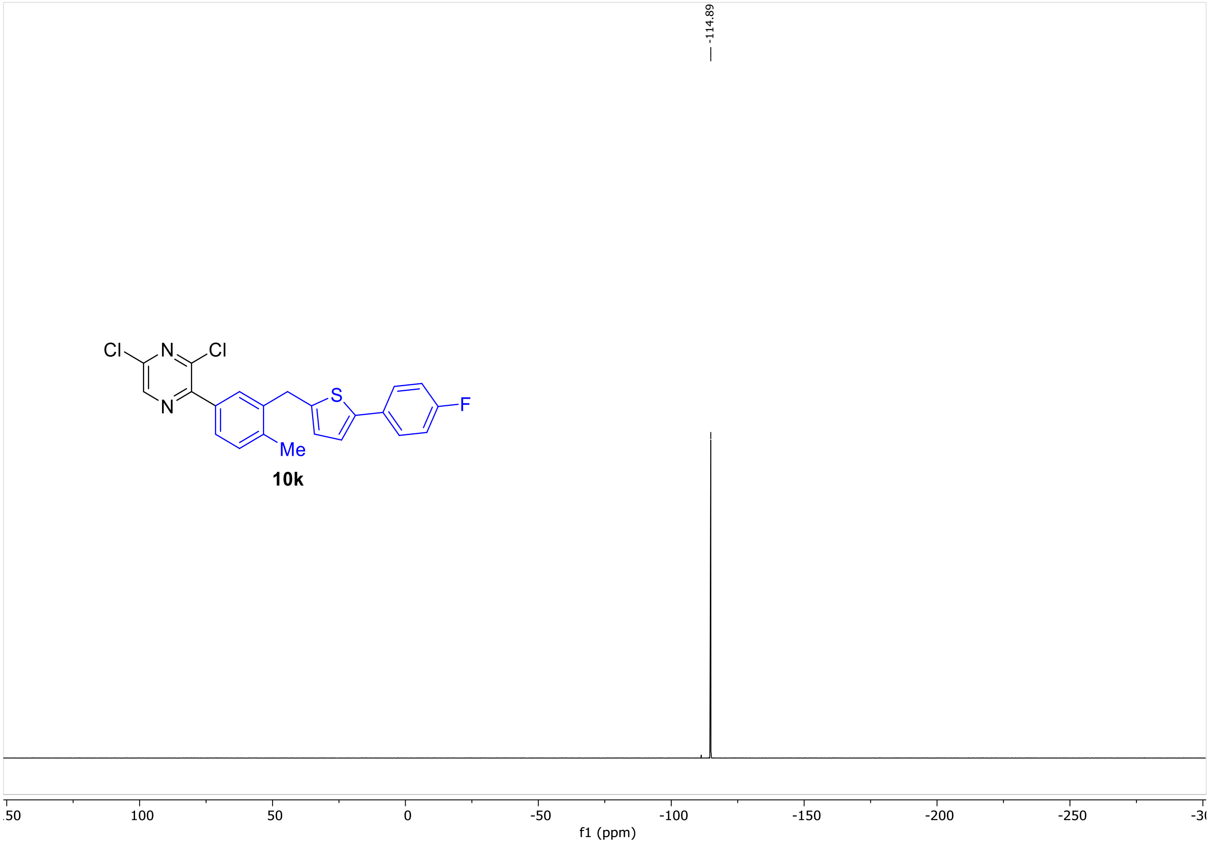
-114.89
Cl
N
Cl
N
S
F
Me
10k
f1 (ppm)

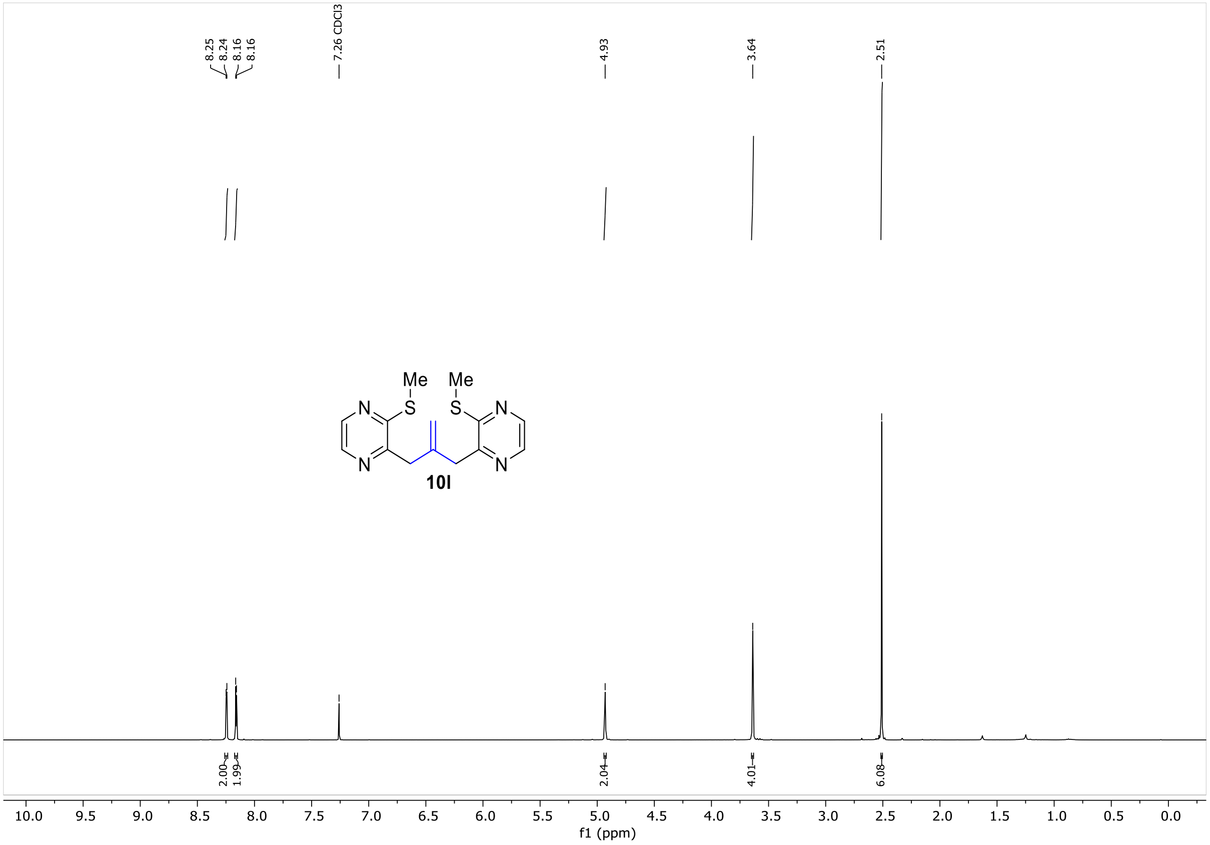
8.25
8.24
8.16
8.16
7.26 CDCl3
4.93
3.64
2.51
Me
Me
S
S
N
N
N
N
10l
2.00
1.99
2.04
4.01
6.08
f1 (ppm)

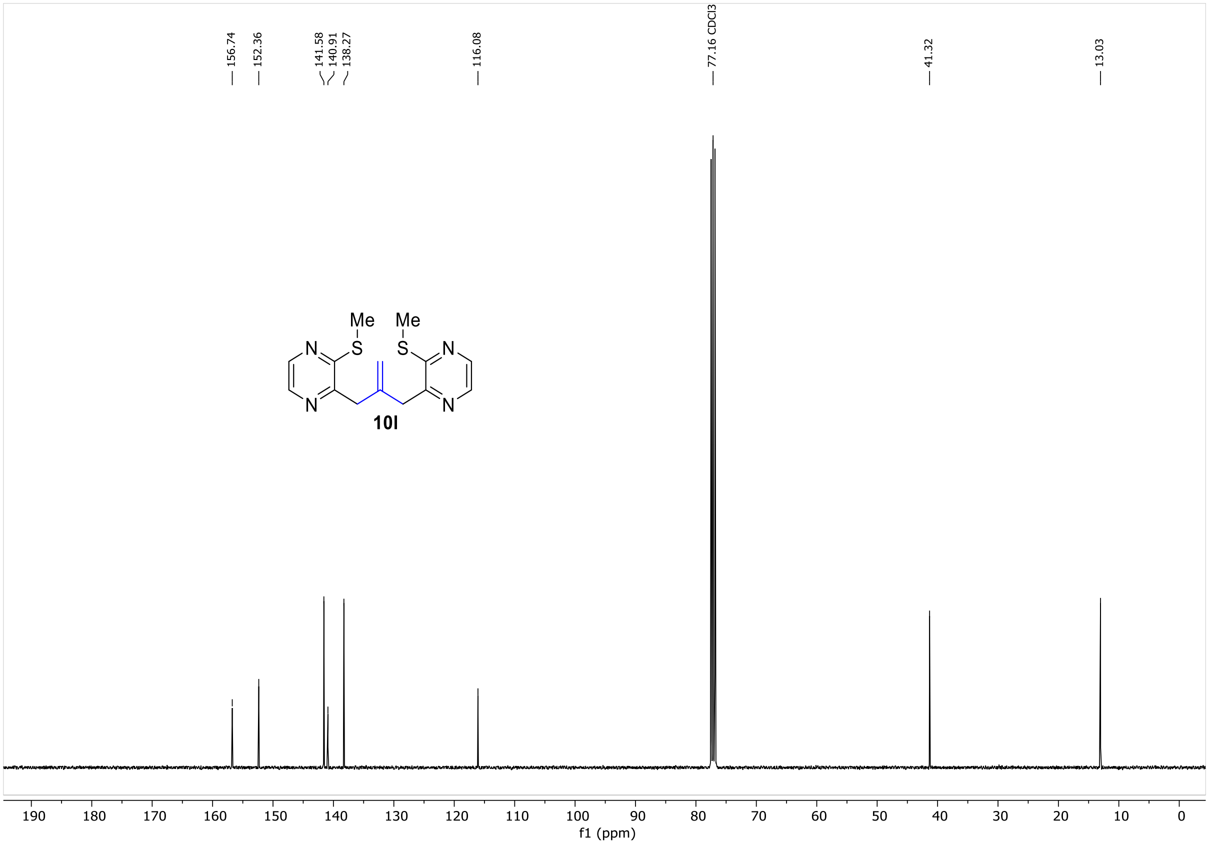
156.74
152.36
141.58
140.91
138.27
116.08
77.16 CDCl3
41.32
13.03
Me
Me
S
S
N
N
N
N
10l
190 180 170 160 150 140 130 120 110 100 90 80 70 60 50 40 30 20 10 0
f1 (ppm)

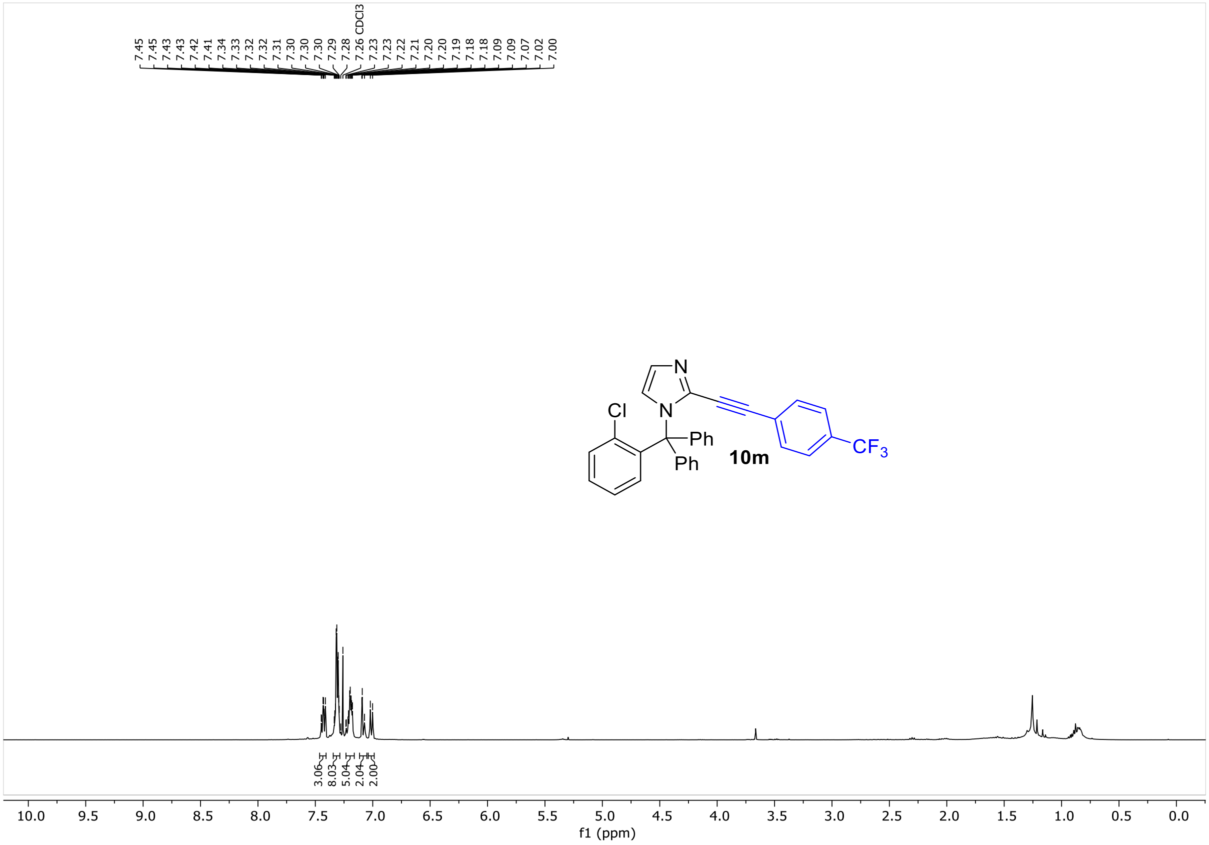
7.45 7.45 7.43 7.43 7.42 7.41 7.34 7.33 7.32 7.32 7.31 7.30 7.30 7.30 7.29 7.28 7.26 CDCl3 7.23 7.23 7.22 7.21 7.20 7.20 7.19 7.18 7.09 7.09 7.07 7.02 7.00
N
N
Cl
Ph
Ph
10m
CF3
3.06
8.03
5.04
2.04
2.00
10.0 9.5 9.0 8.5 8.0 7.5 7.0 6.5 6.0 5.5 5.0 4.5 4.0 3.5 3.0 2.5 2.0 1.5 1.0 0.5 0.0
f1 (ppm)

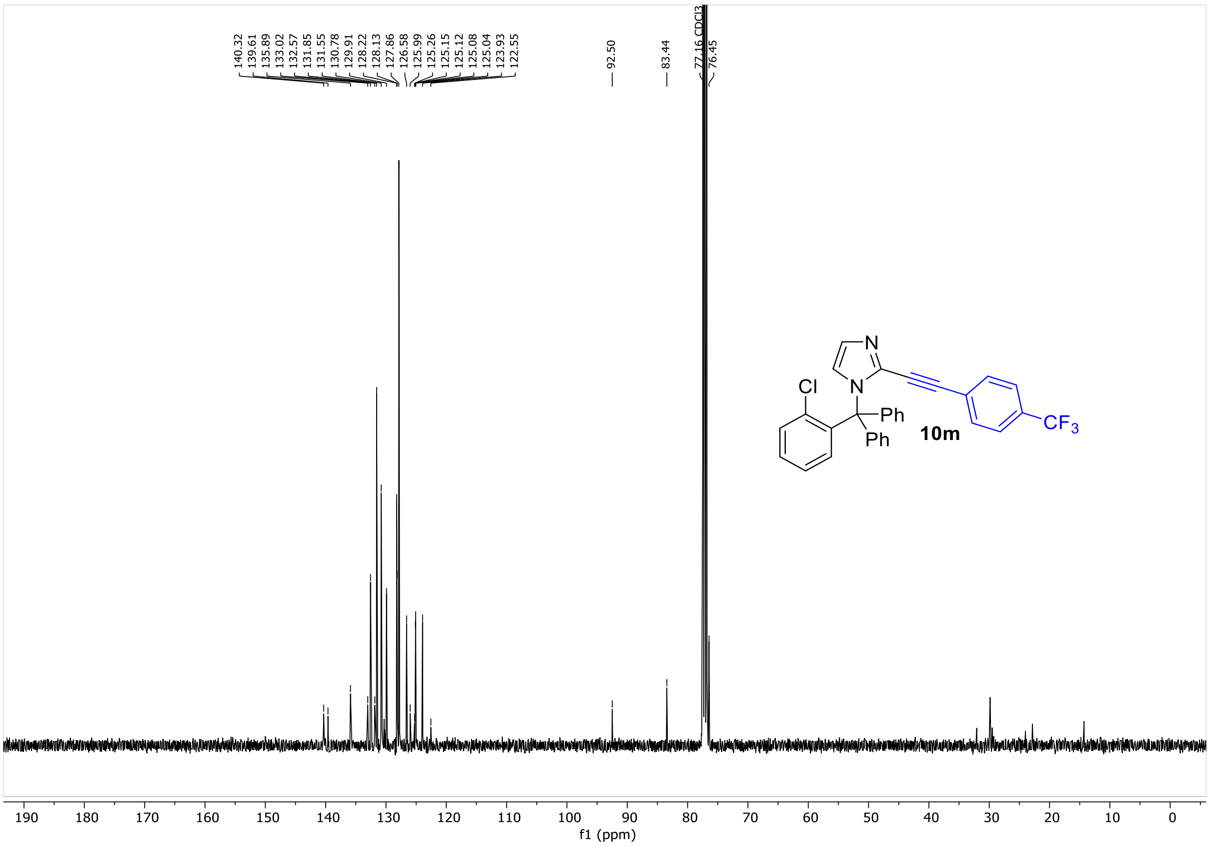
140.32
139.61
135.89
133.02
132.57
131.85
131.55
130.78
129.91
128.22
128.13
127.86
126.58
125.99
125.26
125.15
125.12
125.08
125.04
123.93
122.55
92.50
83.44
77.16 CDCl3
76.45
Cl
N
N
Ph
Ph
10m
CF3
f1 (ppm)

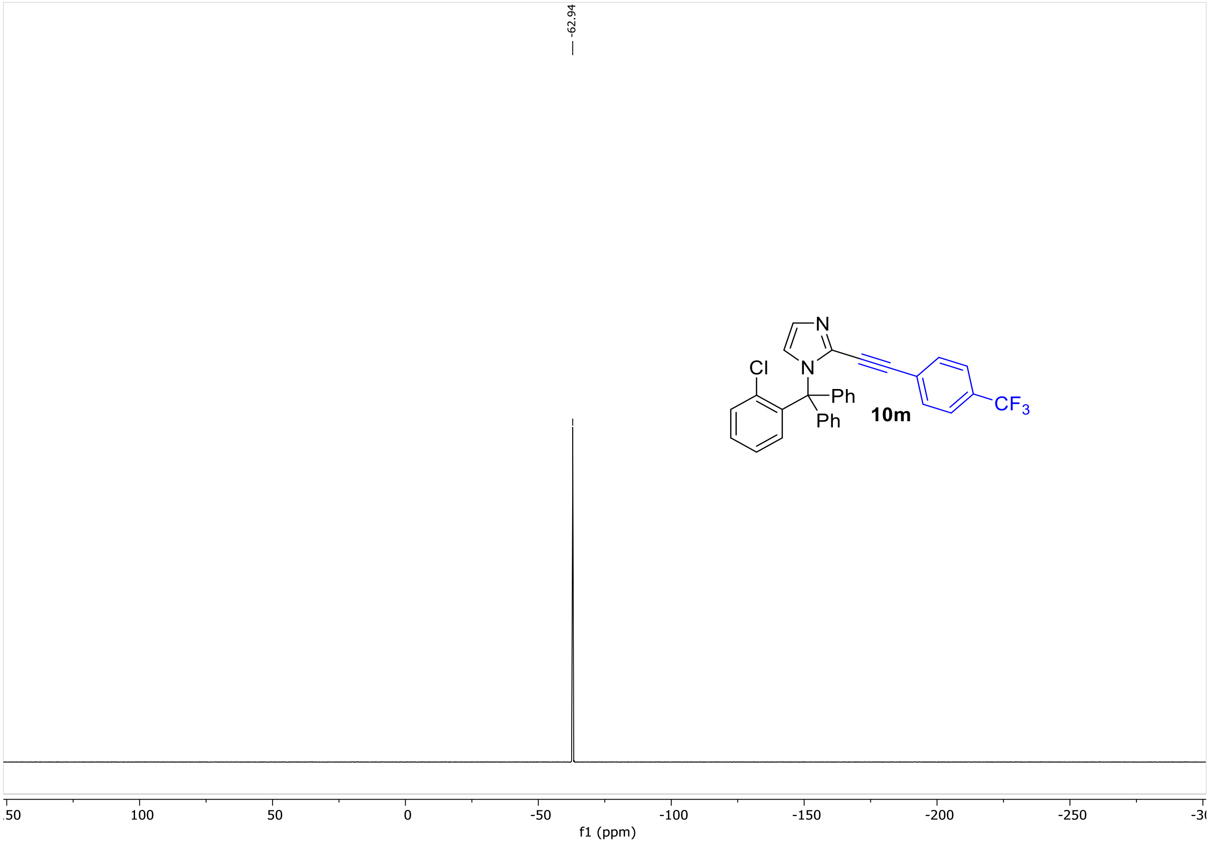
-62.94
Cl
N
N
Ph
Ph
10m
CF3
f1 (ppm)

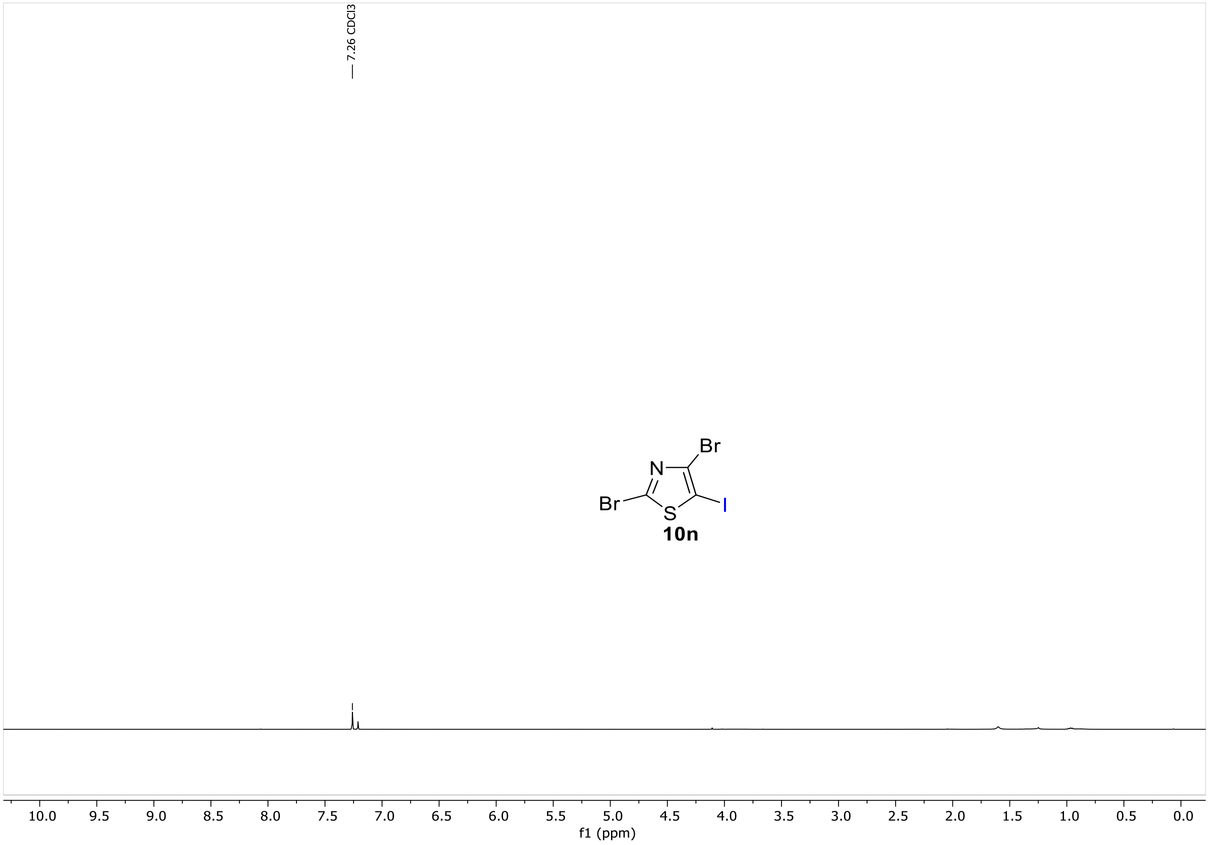
7.26 CDCl3
Br
N
Br
S
I
10n
10.0
9.5
9.0
8.5
8.0
7.5
7.0
6.5
6.0
5.5
5.0
4.5
4.0
3.5
3.0
2.5
2.0
1.5
1.0
0.5
0.0
f1 (ppm)

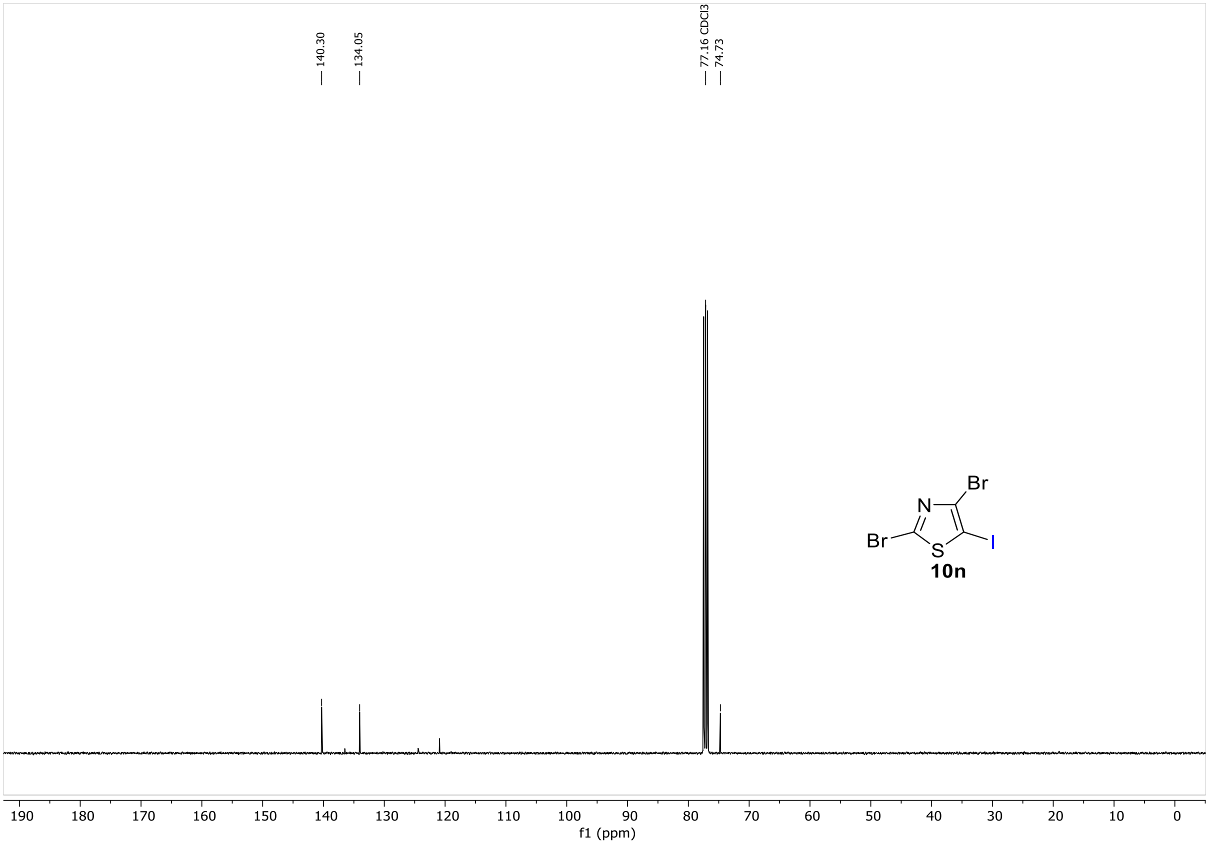
140.30
134.05
77.16 CDCl3
74.73
Br
N
Br
S
I
10n
190
180
170
160
150
140
130
120
110
100
90
80
70
60
50
40
30
20
10
0
f1 (ppm)

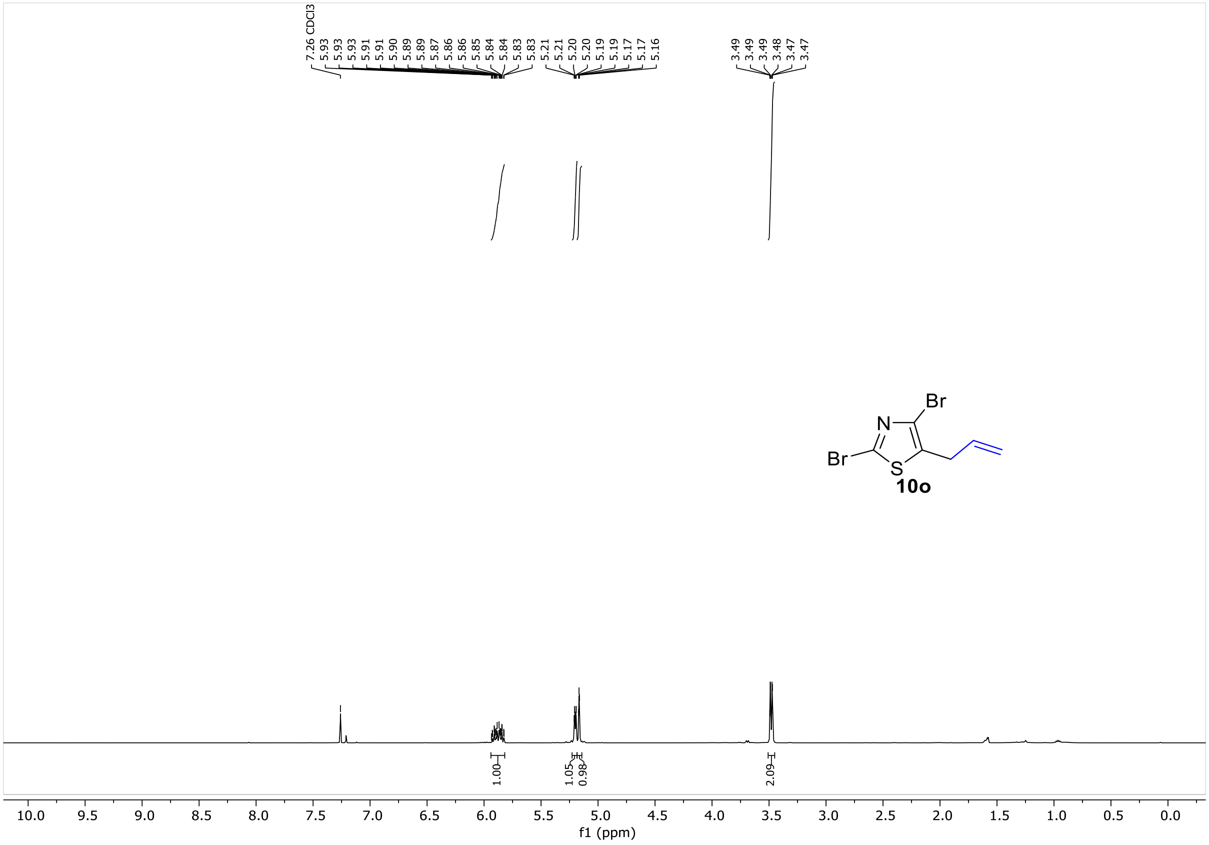
7.26 CDCl3
5.93
5.93
5.93
5.91
5.91
5.90
5.89
5.89
5.87
5.86
5.86
5.85
5.84
5.84
5.83
5.83
5.21
5.21
5.20
5.20
5.19
5.19
5.17
5.17
5.16
3.49
3.49
3.49
3.48
3.47
3.47
Br
N
Br
S
10o
1.00
1.05
0.98
2.09
10.0
9.5
9.0
8.5
8.0
7.5
7.0
6.5
6.0
5.5
5.0
4.5
4.0
3.5
3.0
2.5
2.0
1.5
1.0
0.5
0.0
f1 (ppm)

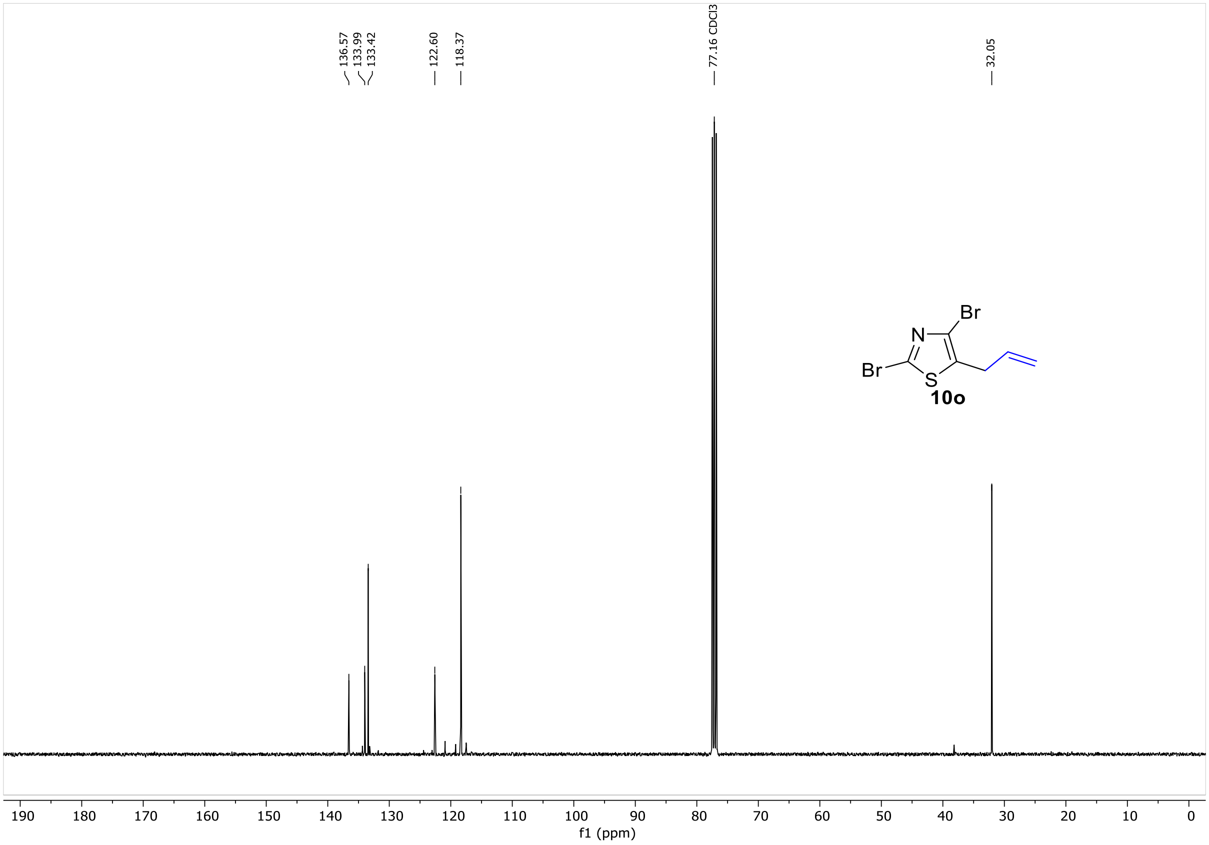
136.57
133.99
133.42
122.60
118.37
77.16 CDCl3
32.05
Br
N
Br
S
10o
190
180
170
160
150
140
130
120
110
100
90
80
70
60
50
40
30
20
10
0
f1 (ppm)

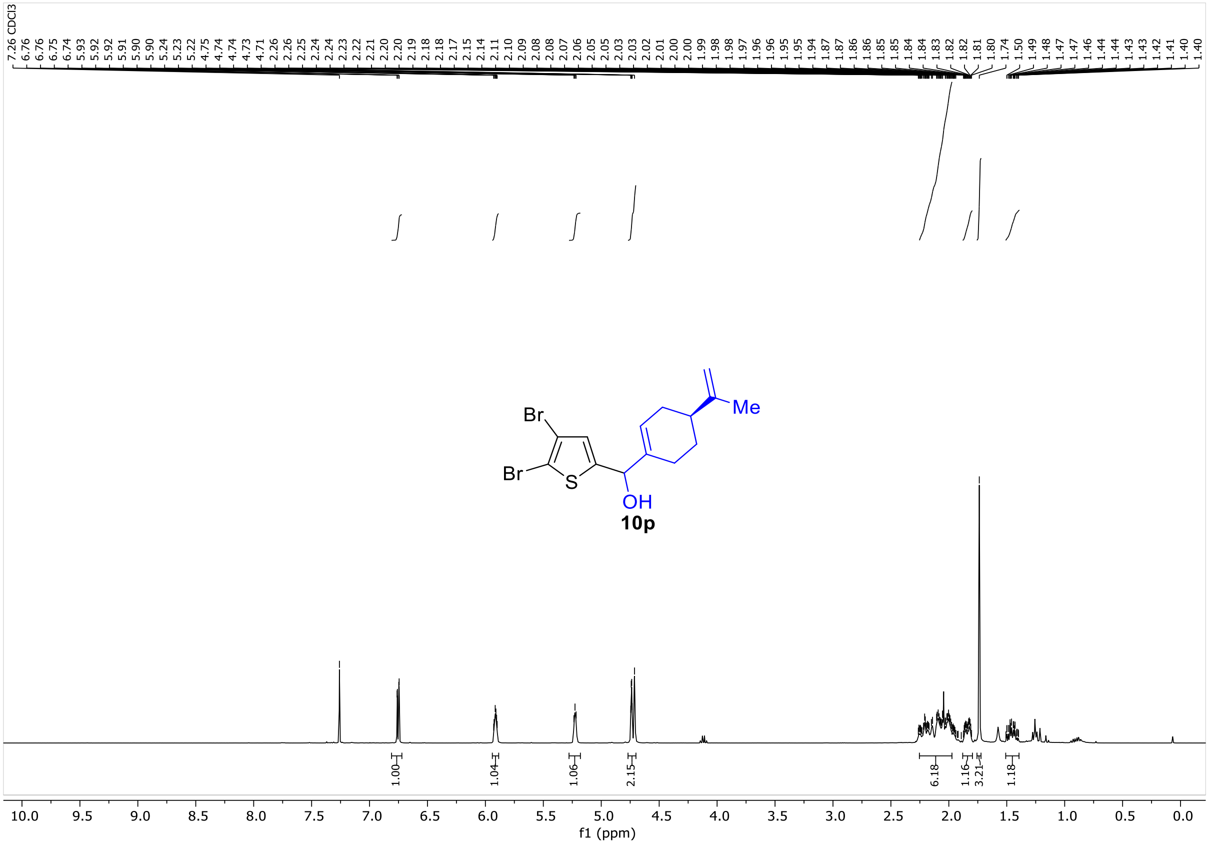
Br
Br
S
OH
Me
10p
f1 (ppm)

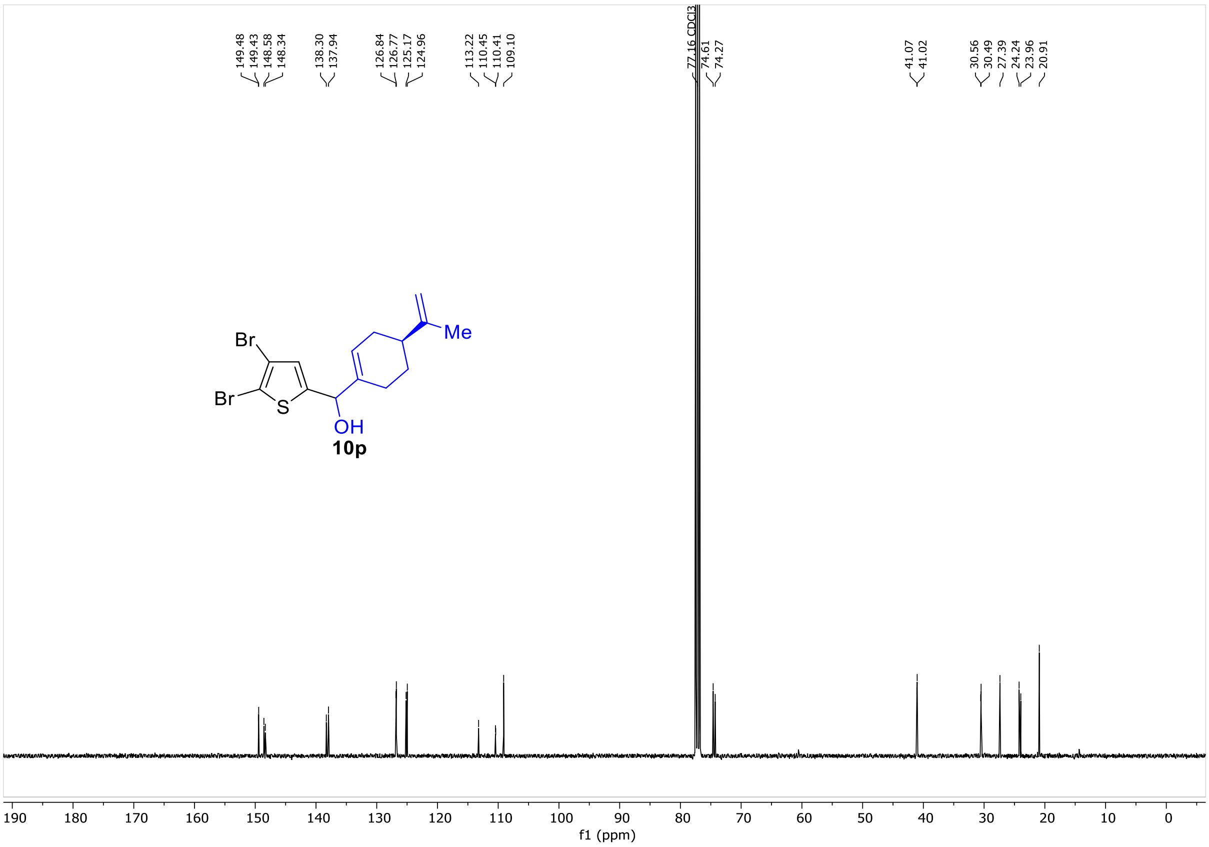
Br
Br
S
OH
Me
10p
f1 (ppm)

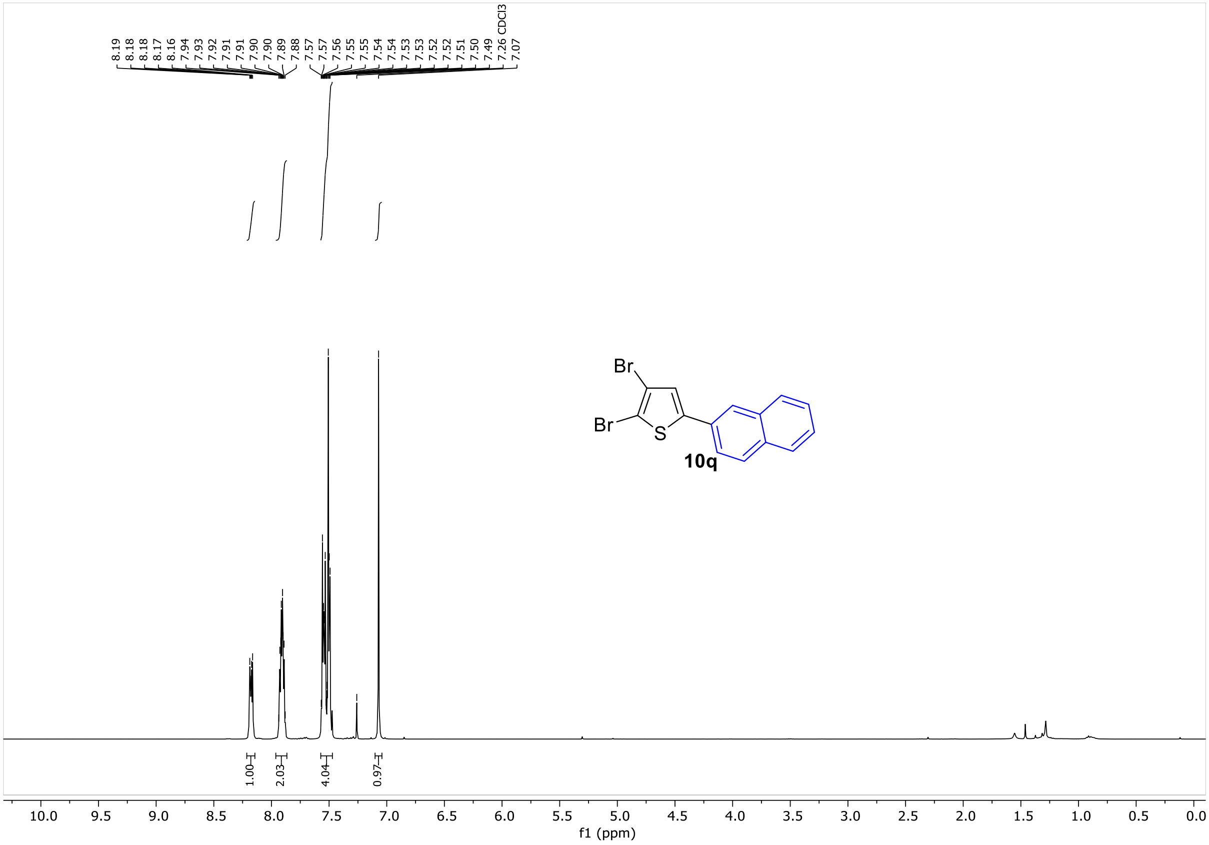
8.19
8.18
8.18
8.17
8.16
7.94
7.93
7.92
7.92
7.91
7.91
7.90
7.90
7.89
7.88
7.57
7.57
7.56
7.55
7.55
7.54
7.54
7.53
7.53
7.52
7.52
7.51
7.50
7.49
7.26 CDCl3
7.07
Br
Br
S
10q
1.00
2.03
4.04
0.97
10.0 9.5 9.0 8.5 8.0 7.5 7.0 6.5 6.0 5.5 5.0 4.5 4.0 3.5 3.0 2.5 2.0 1.5 1.0 0.5 0.0
f1 (ppm)

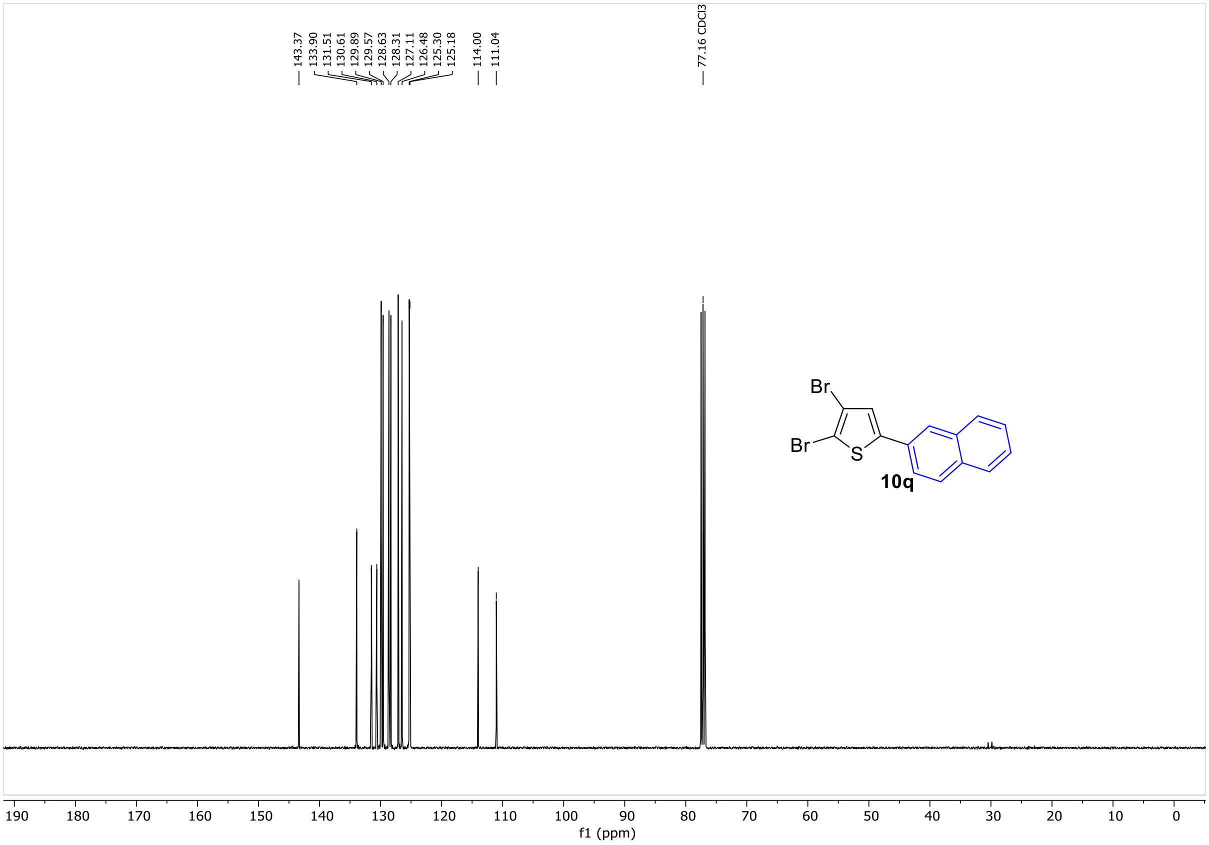
143.37
133.90
131.51
130.61
129.89
129.57
128.63
128.31
127.11
126.48
125.30
125.18
114.00
111.04
77.16 CDCl3
Br
Br
S
10q
190 180 170 160 150 140 130 120 110 100 90 80 70 60 50 40 30 20 10 0
f1 (ppm)

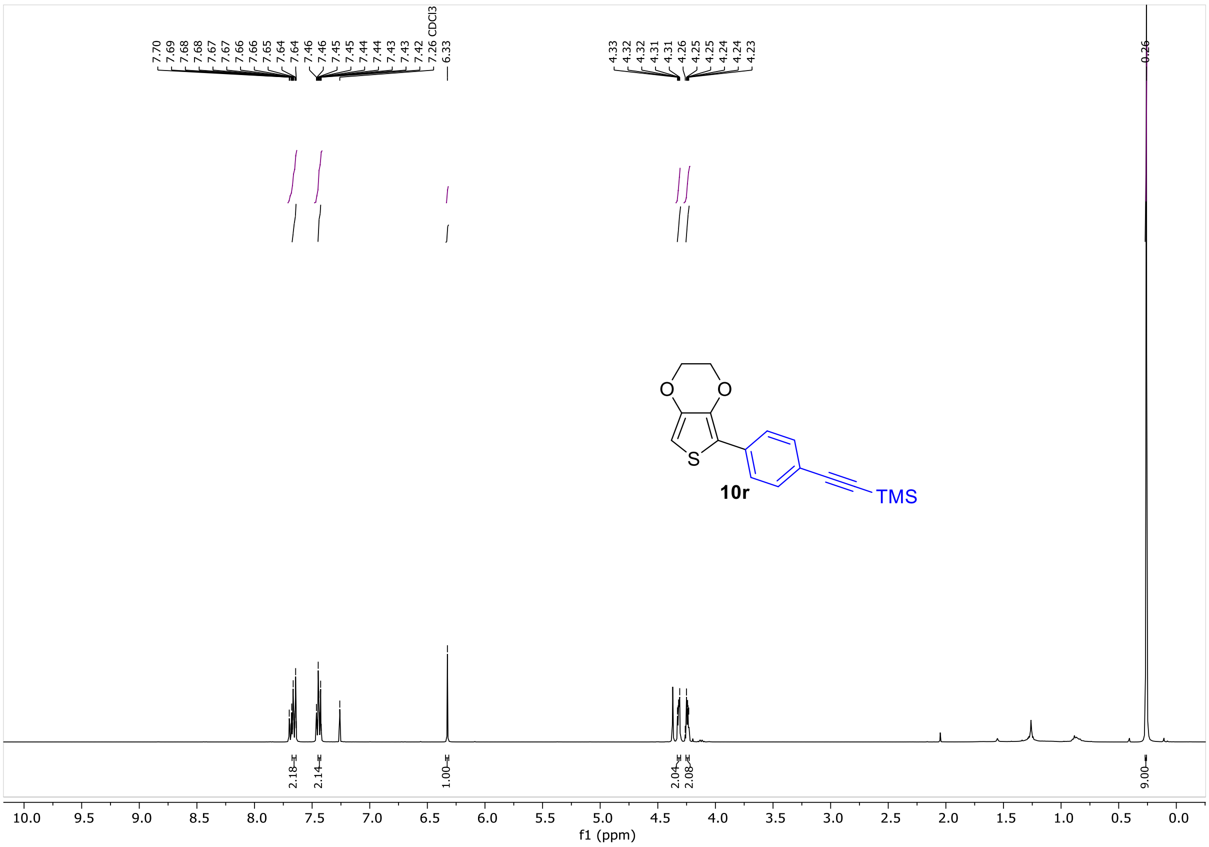
7.70 7.69 7.68 7.68 7.67 7.67 7.66 7.66 7.65 7.64 7.64 7.46 7.46 7.45 7.45 7.44 7.44 7.43 7.43 7.42 7.26 CDCl3 6.33
4.33 4.32 4.32 4.31 4.31 4.26 4.25 4.25 4.24 4.24 4.23
0.26
O
O
S
10r
TMS
2.18 2.14 1.00 2.04 2.08 9.00
f1 (ppm)

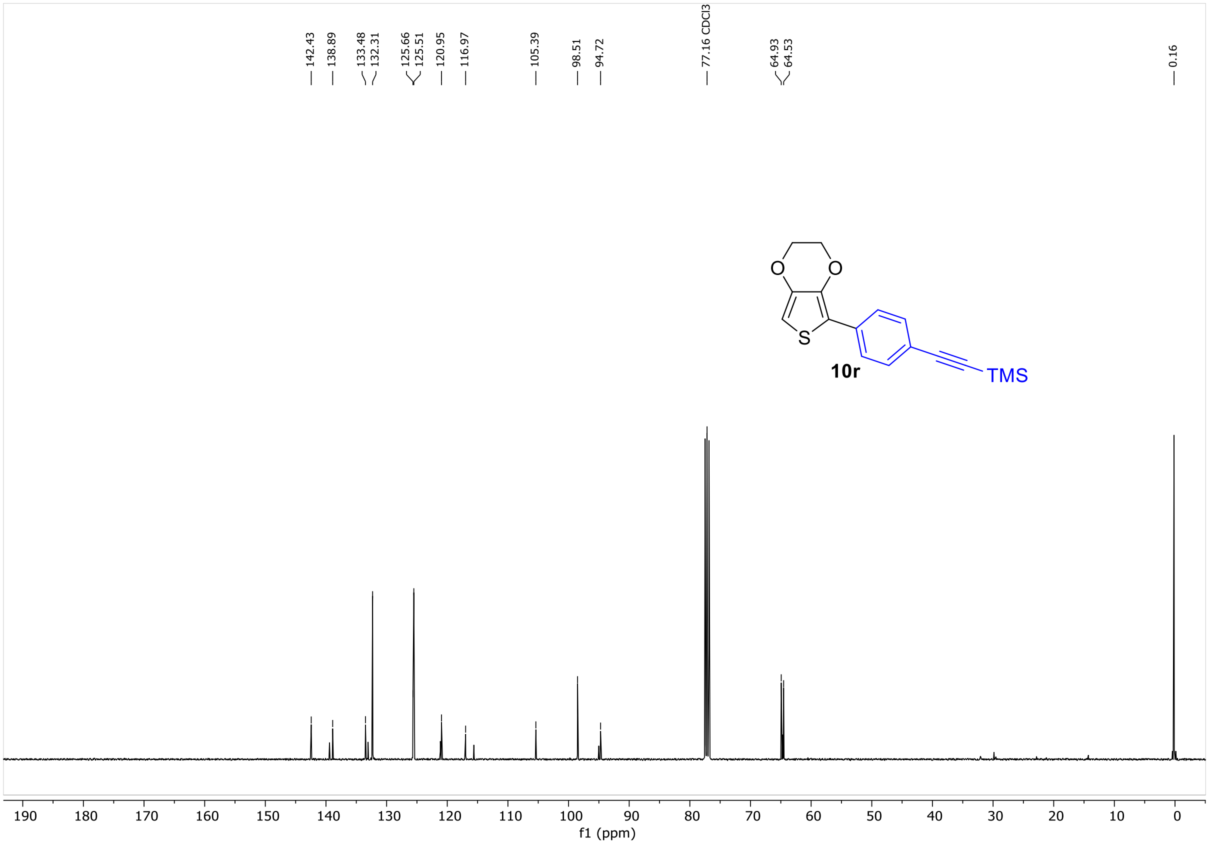
142.43 138.89 133.48 132.31 125.66 125.51 120.95 116.97 105.39 98.51 94.72 77.16 CDCl3 64.93 64.53 0.16
O
O
S
10r
TMS
f1 (ppm)

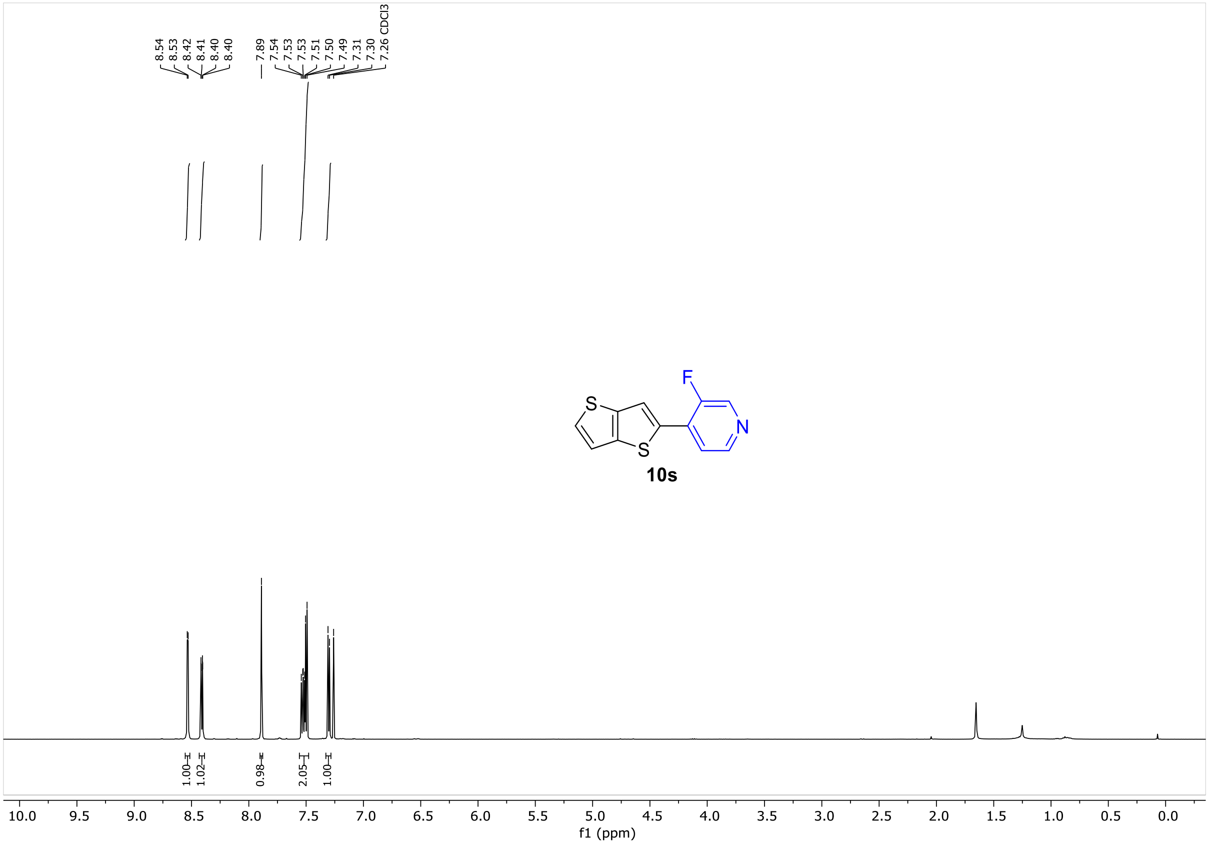
8.54
8.53
8.42
8.41
8.40
8.40
7.89
7.54
7.53
7.53
7.51
7.50
7.49
7.31
7.30
7.26 CDCl3
1.00
1.02
0.98
2.05
1.00
10s
f1 (ppm)

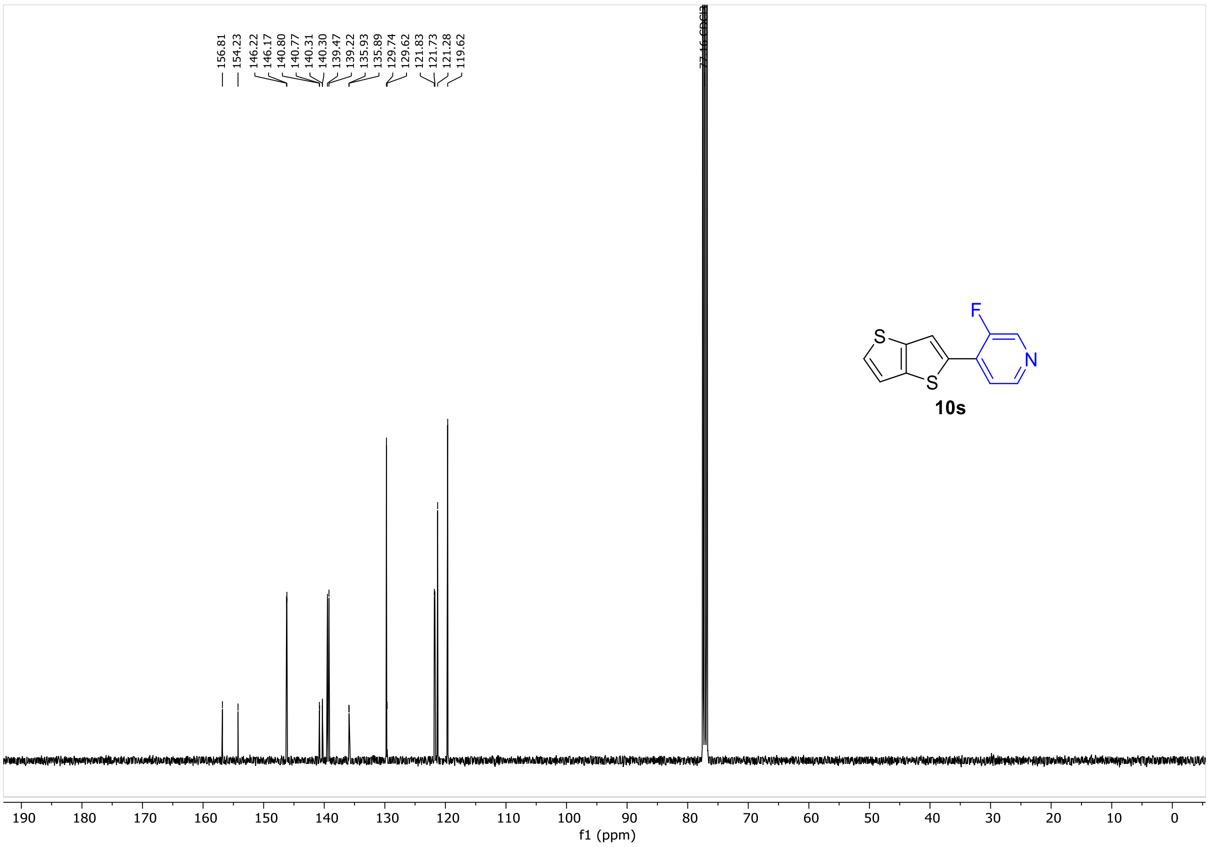
156.81
154.23
146.22
146.17
140.80
140.77
140.31
140.30
139.47
139.22
135.93
135.89
129.74
129.62
121.83
121.73
121.28
119.62
10s
f1 (ppm)

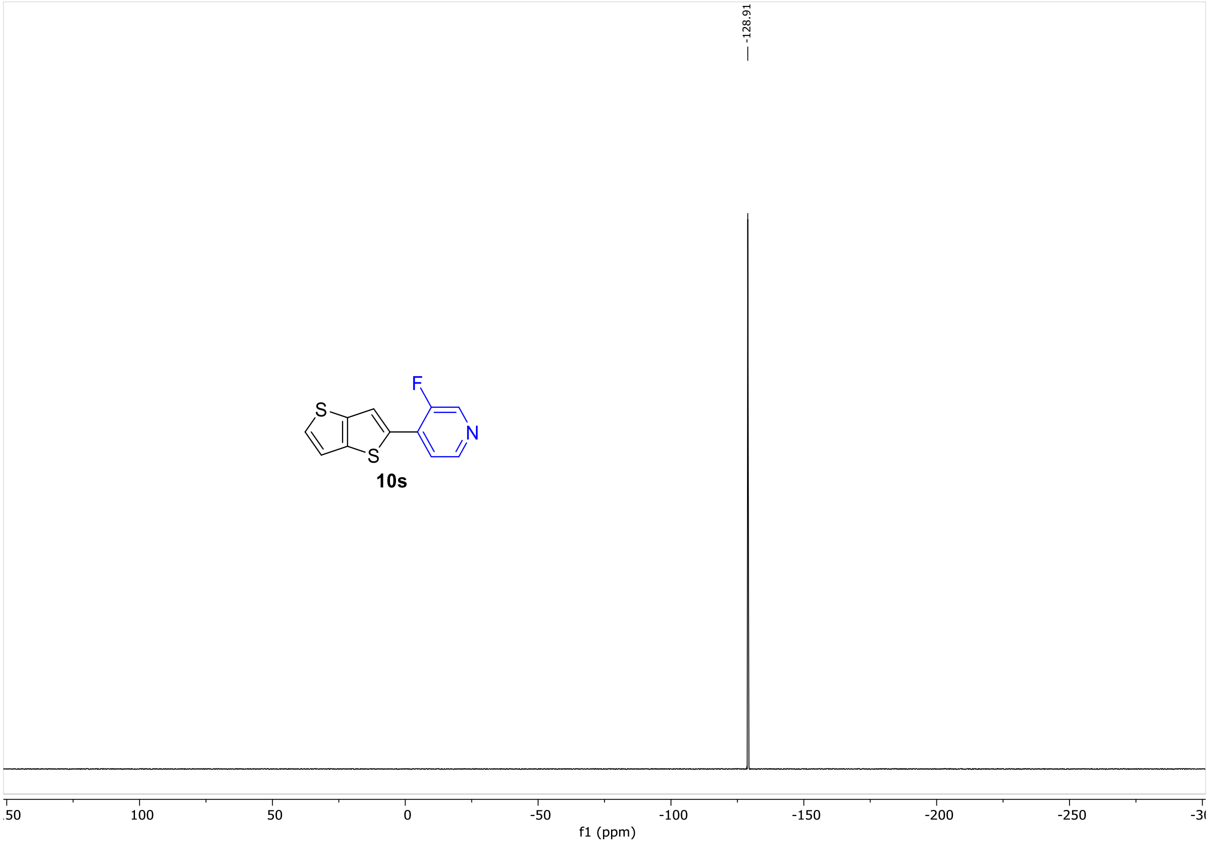

-128.91
10s
150
100
50
0
-50
-100
-150
-200
-250
f1 (ppm)

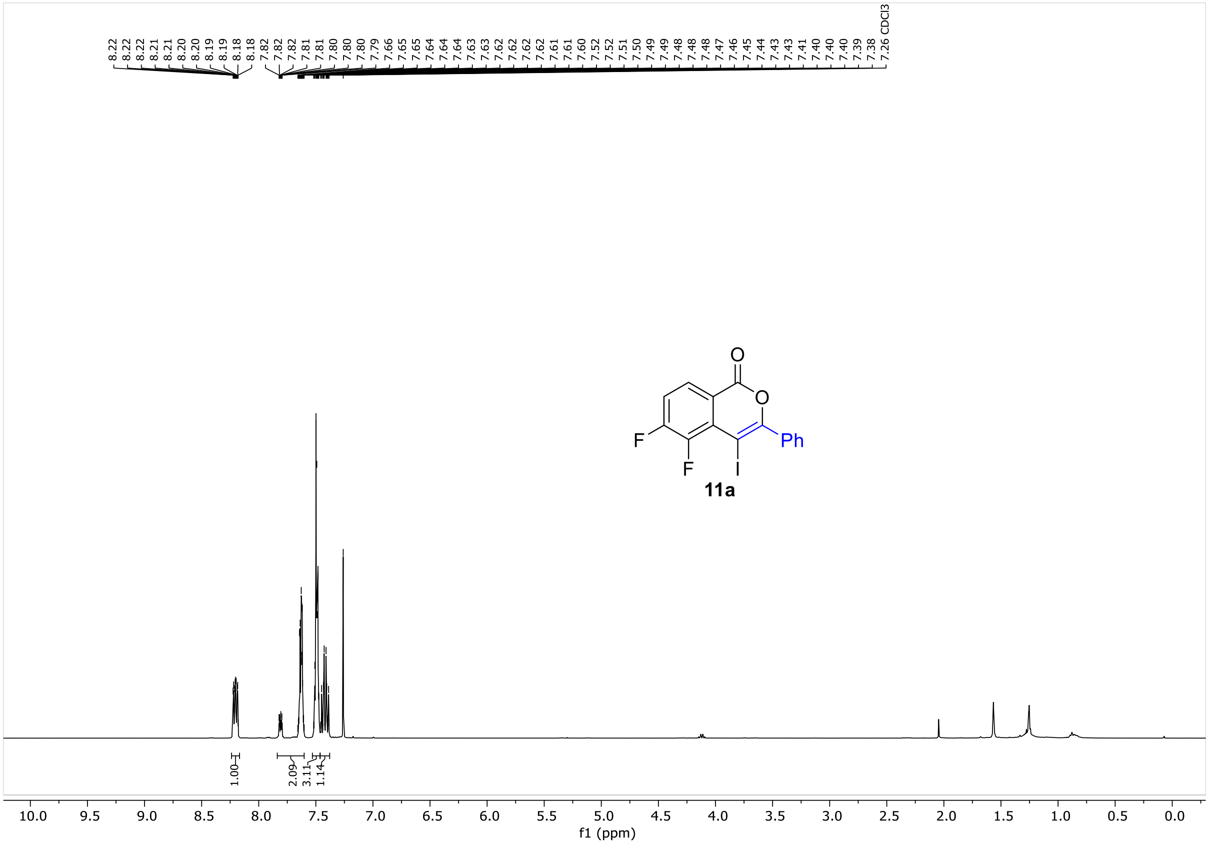

8.22
8.22
8.22
8.21
8.21
8.20
8.20
8.19
8.19
8.18
8.18
7.82
7.82
7.82
7.81
7.81
7.80
7.80
7.80
7.79
7.66
7.65
7.65
7.64
7.64
7.64
7.63
7.63
7.62
7.62
7.62
7.62
7.61
7.61
7.60
7.52
7.52
7.51
7.50
7.49
7.49
7.48
7.48
7.48
7.47
7.46
7.45
7.44
7.43
7.43
7.41
7.40
7.40
7.40
7.39
7.38
7.26 CDCl3
11a
1.00
2.09
3.11
1.14
10.0
9.5
9.0
8.5
8.0
7.5
7.0
6.5
6.0
5.5
5.0
4.5
4.0
3.5
3.0
2.5
2.0
1.5
1.0
0.5
0.0
f1 (ppm)

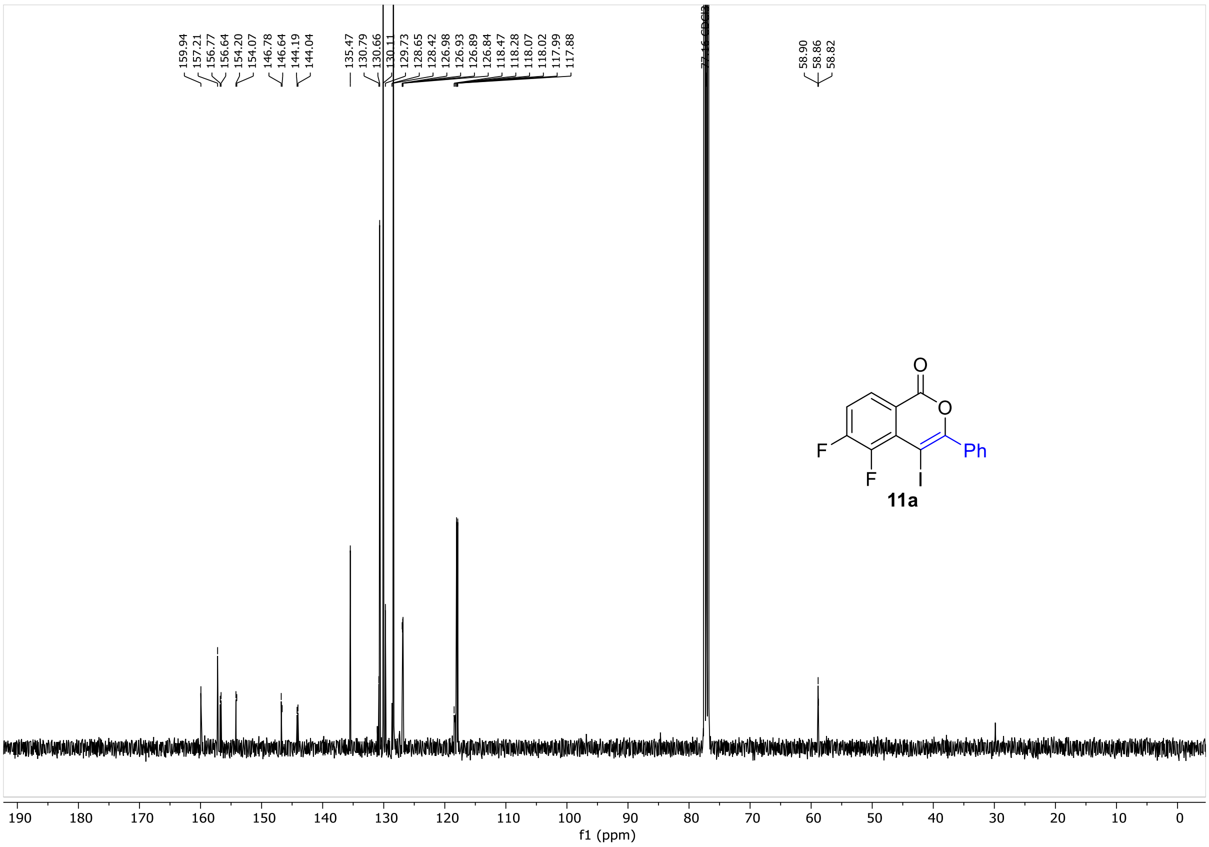
159.94
157.21
156.77
156.64
154.20
154.07
146.78
146.64
144.19
144.04
135.47
130.79
130.66
130.11
129.73
128.65
128.42
126.98
126.93
126.89
126.84
118.47
118.28
118.07
118.02
117.99
117.88
58.90
58.86
58.82
O
O
F
F
I
Ph
11a
190 180 170 160 150 140 130 120 110 100 90 80 70 60 50 40 30 20 10 0
f1 (ppm)

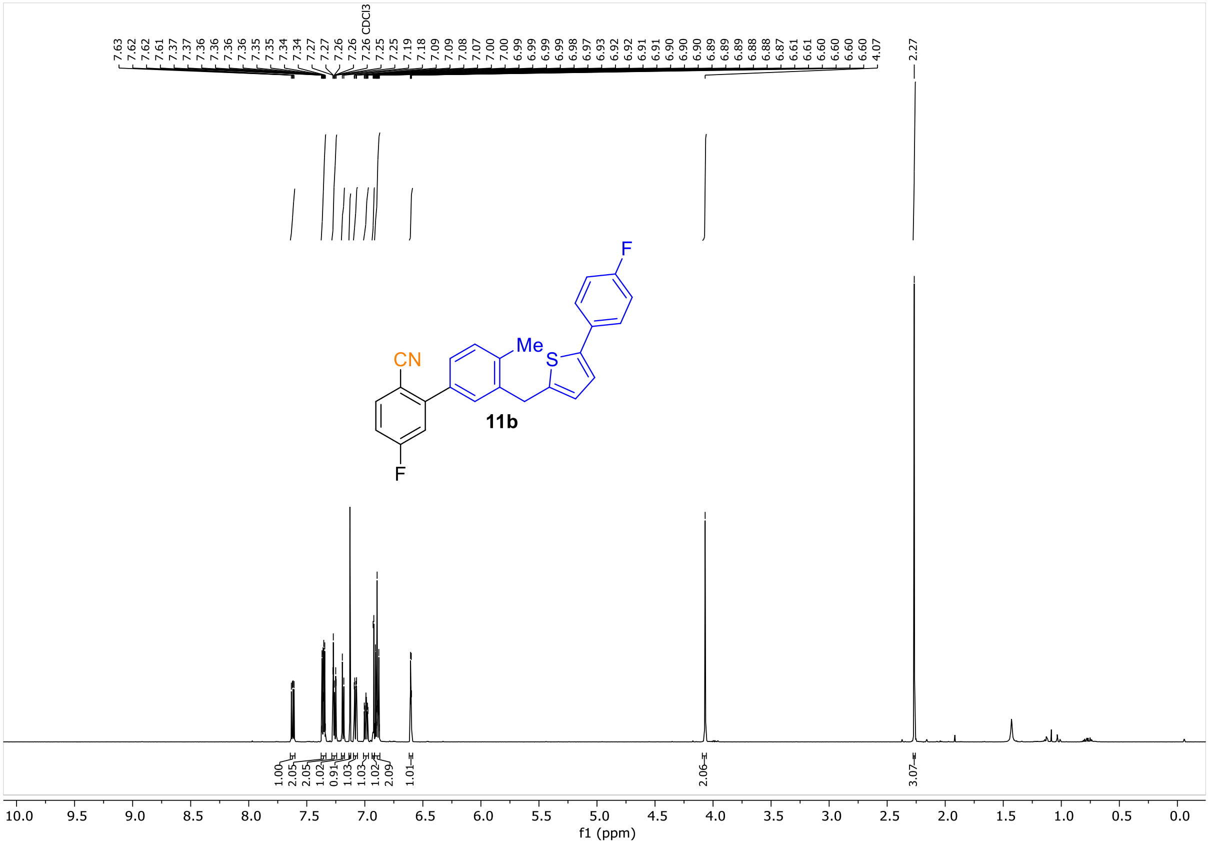
7.63
7.62
7.61
7.37
7.37
7.36
7.36
7.36
7.35
7.35
7.34
7.34
7.27
7.27
7.26
7.26 CDCl3
7.26
7.25
7.19
7.18
7.09
7.09
7.08
7.07
7.00
7.00
6.99
6.99
6.99
6.99
6.98
6.97
6.93
6.92
6.92
6.91
6.91
6.90
6.90
6.90
6.89
6.89
6.88
6.88
6.87
6.61
6.61
6.60
6.60
6.60
6.60
4.07
2.27
CN
Me
S
F
F
11b
1.00
2.05
2.05
1.02
0.91
1.02
1.03
1.03
1.02
2.09
1.01
2.06
3.07
10.0 9.5 9.0 8.5 8.0 7.5 7.0 6.5 6.0 5.5 5.0 4.5 4.0 3.5 3.0 2.5 2.0 1.5 1.0 0.5 0.0
f1 (ppm)

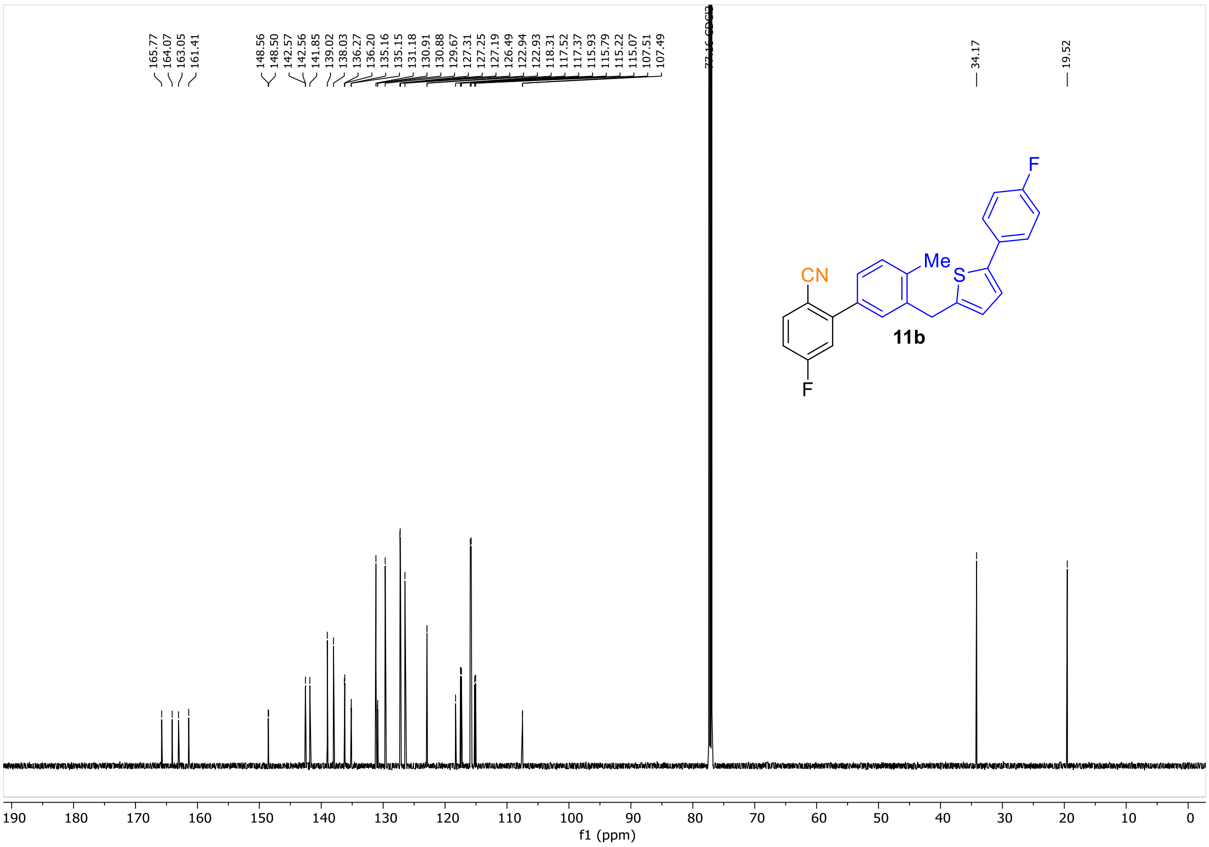
165.77
164.07
163.05
161.41
148.56
148.50
142.57
142.56
141.85
139.02
138.03
136.27
136.20
135.16
135.15
131.18
130.91
130.88
129.67
127.31
127.25
127.19
126.49
122.94
122.93
118.31
117.52
117.37
115.93
115.79
115.22
115.07
107.51
107.49
77.16 CDCl3
34.17
19.52
CN
Me
S
F
11b
F
f1 (ppm)

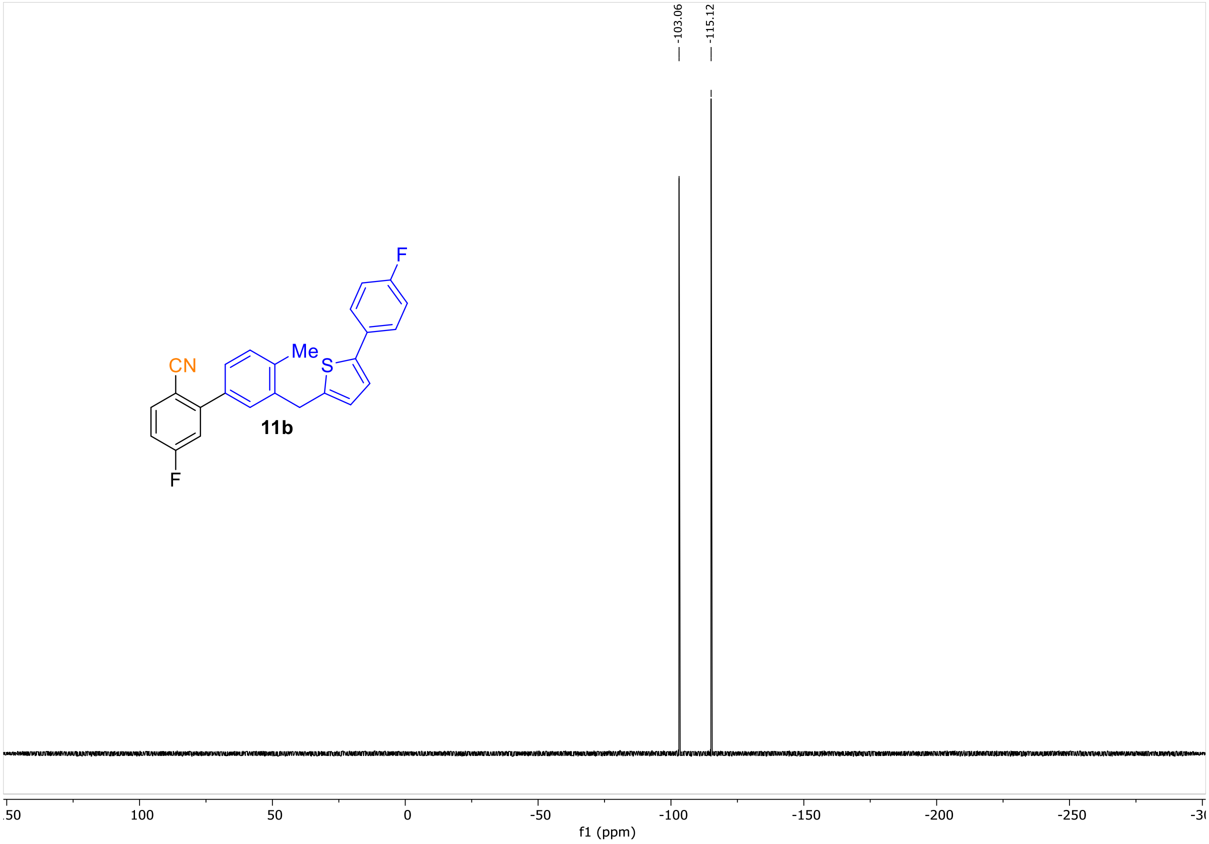
-103.06
-115.12
CN
Me
S
F
11b
F
f1 (ppm)

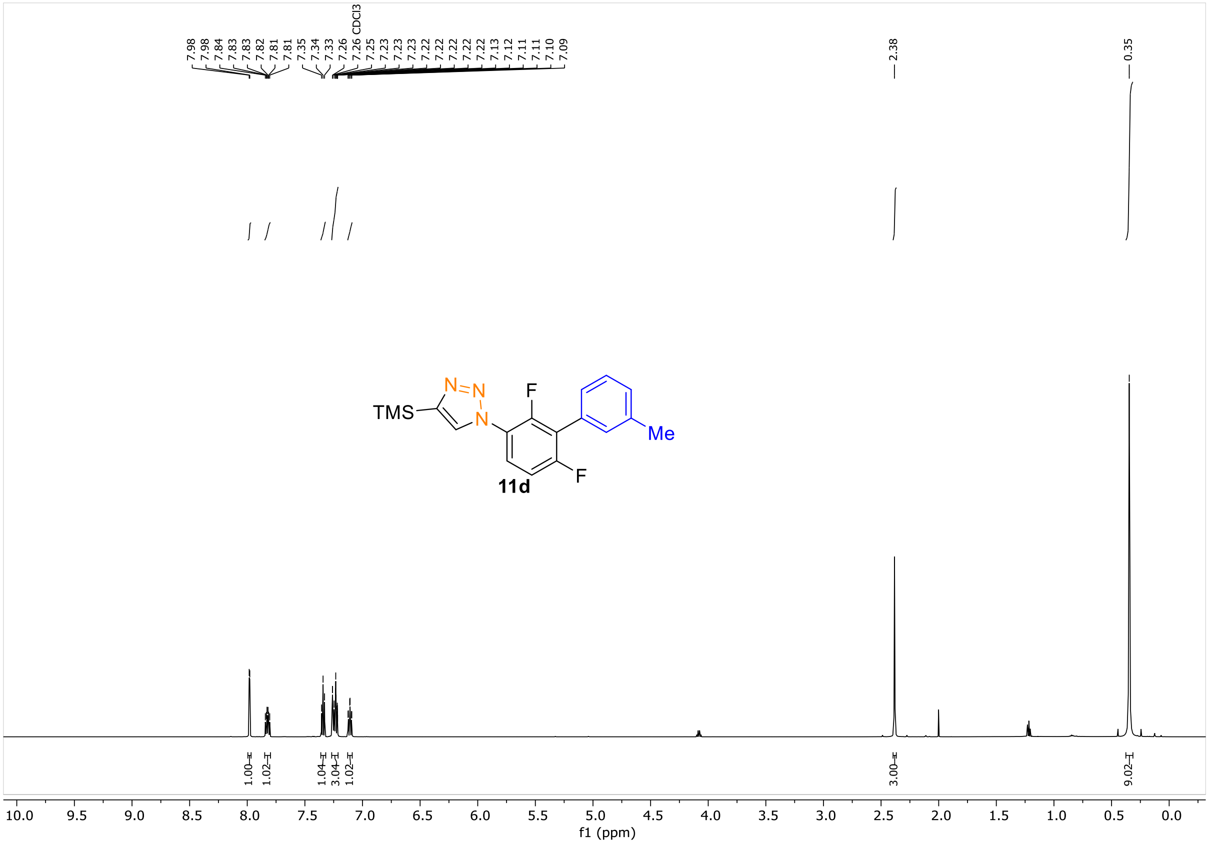
TMS
F
Me
F
11d
f1 (ppm)

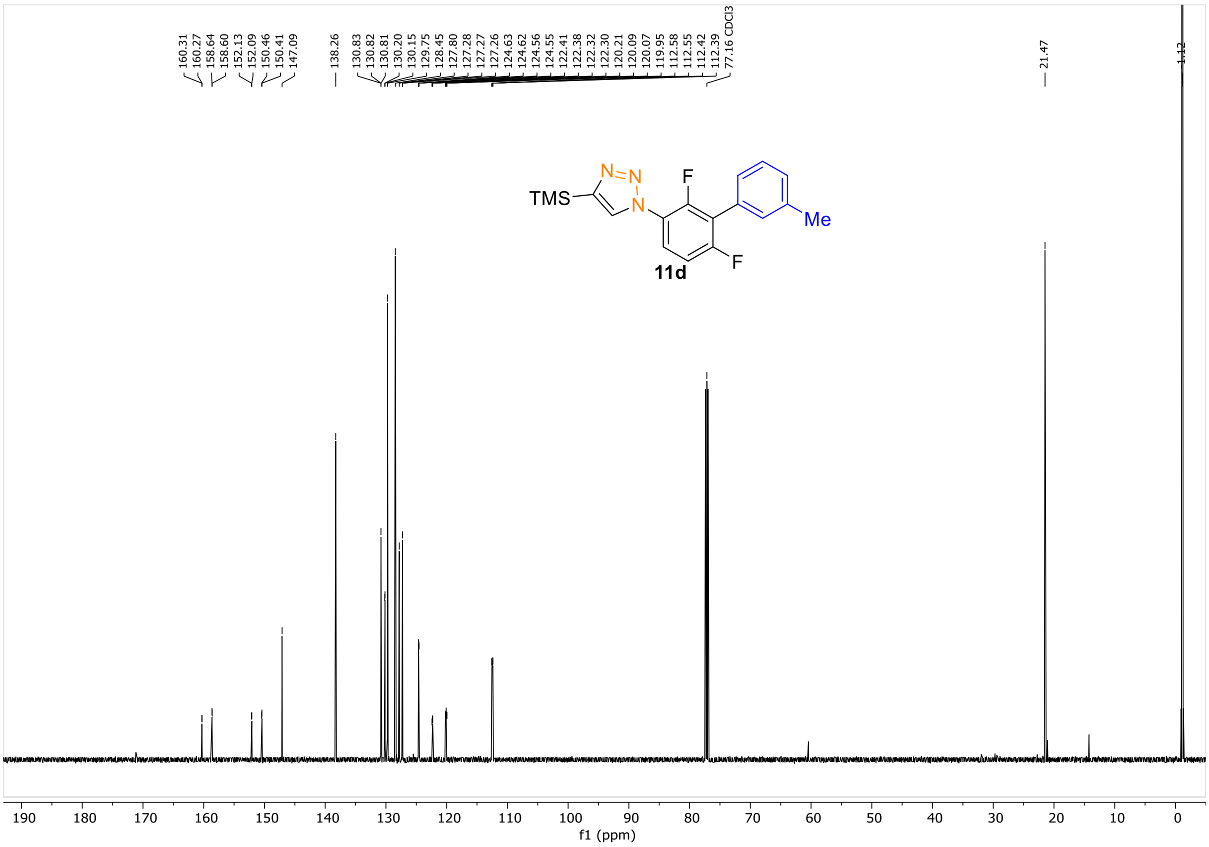
TMS
F
Me
F
11d
f1 (ppm)

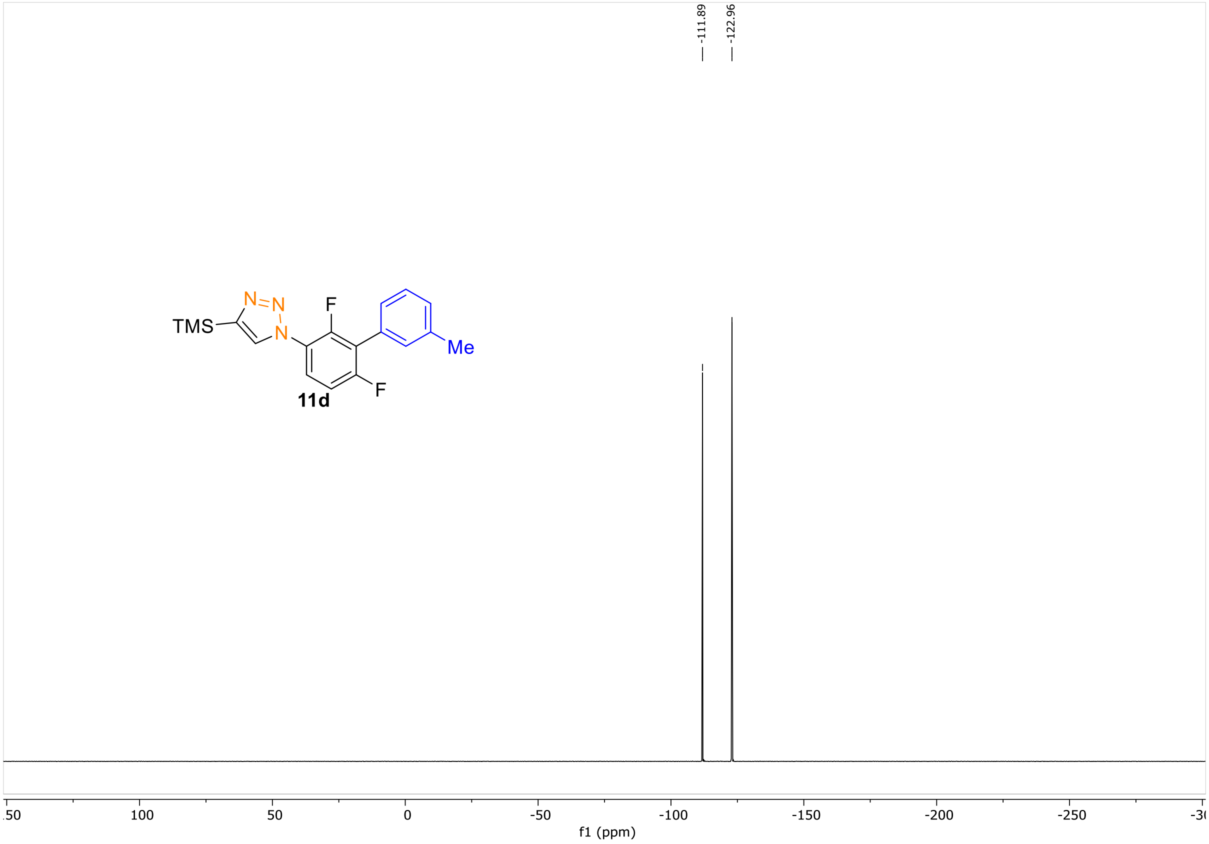
-111.89
-122.96
TMS
N
N
N
F
F
Me
11d
f1 (ppm)

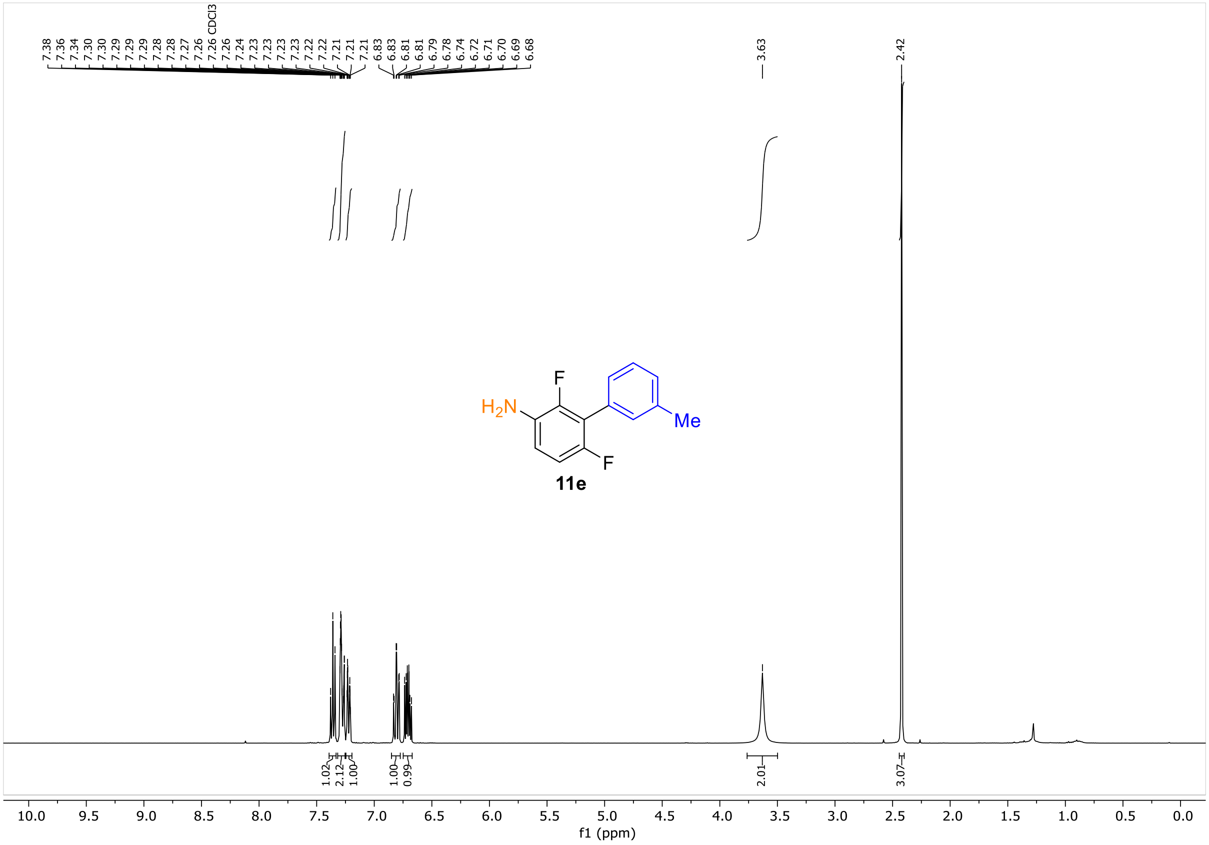
CDCl3
3.63
2.42
H2N
F
F
Me
11e
f1 (ppm)

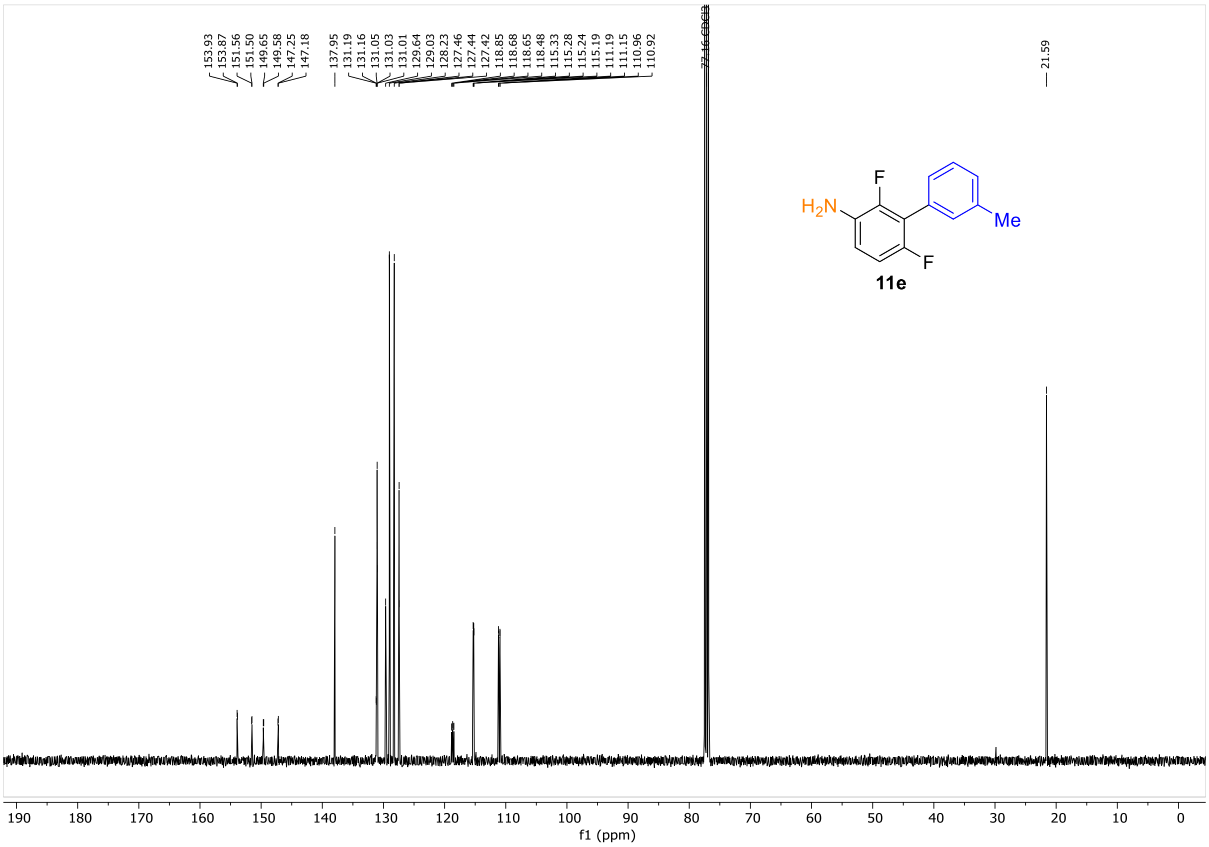
153.93
153.87
151.56
151.50
149.65
149.58
147.25
147.18
137.95
131.19
131.16
131.05
131.03
131.01
129.64
129.03
128.23
127.46
127.44
127.42
118.85
118.68
118.65
118.48
115.33
115.28
115.24
115.19
111.19
111.15
110.96
110.92
77.16 CDCl3
21.59
H2N
F
F
Me
11e
f1 (ppm)

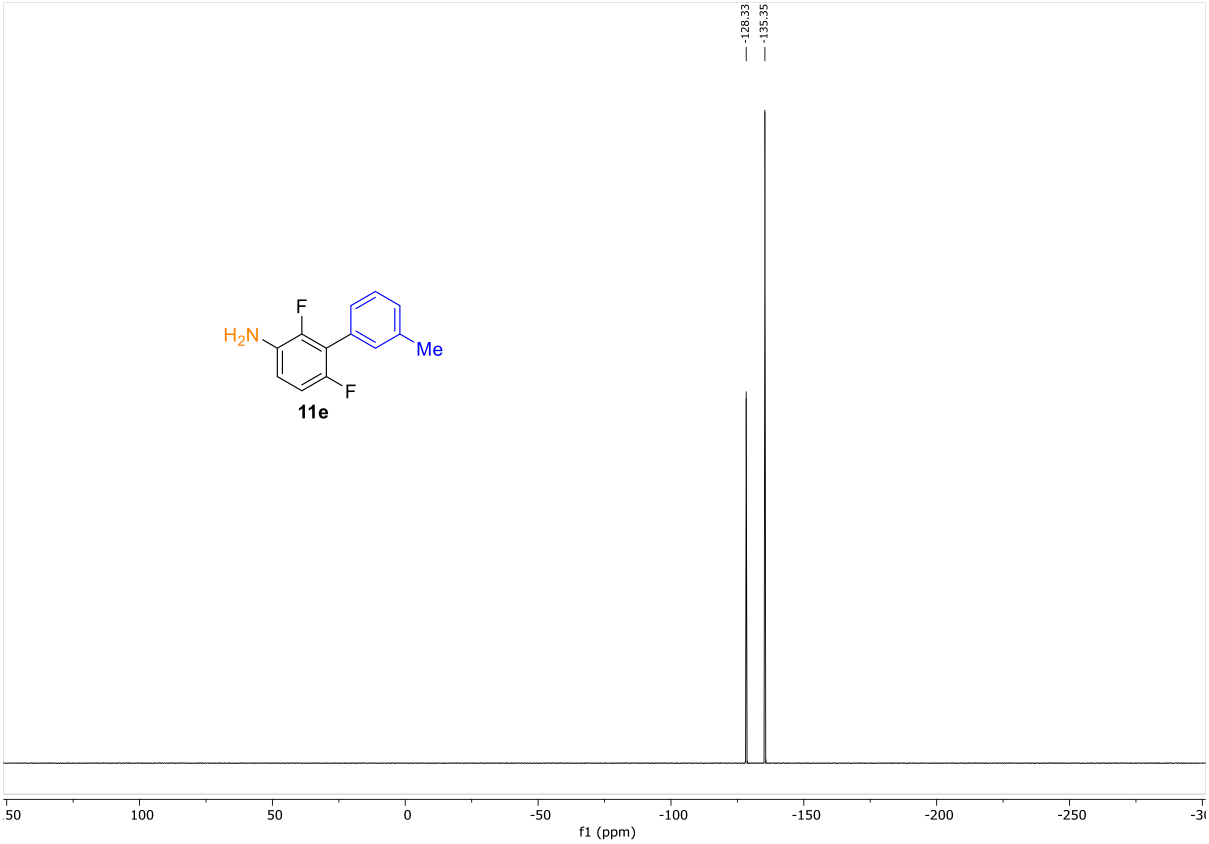
-128.33
-135.35
H2N
F
F
Me
11e
f1 (ppm)
